# Key Features of Digital Phenotyping for Monitoring Mental Disorders: Systematic Review

**DOI:** 10.2196/77331

**Published:** 2025-11-05

**Authors:** Hyun Woo Jung, Do Yeon Kim, Ilju Lee, Ok Kim, Seungjin Lee, Sujin Lee, Un Sun Chung, Jae-Hyun Kim, Sehwan Kim, Jung Won Kim, Ah Lahm Shin, Jung Jae Lee

**Affiliations:** 1 Department of Psychiatry, College of Medicine, Dankook University, Cheonan-si, Republic of Korea; 2 Digital Mental Health Innovation Center, Dankook University, Dongnam-gu, 201 Manghyang-ro, Cheonan-si, 31116, Republic of Korea, 82 415506390; 3 Department of Psychotherapy, College of Health and Welfare Sciences, Dankook University, Cheonan-si, Republic of Korea; 4 Department of Psychology, Graduate School, Dankook University, Cheonan-si, Republic of Korea; 5 Department of Medical Laser, Graduate School of Medicine, Dankook University, Cheonan-si, Republic of Korea; 6 Department of Psychiatry, School of Medicine, Kyungpook National University, Daegu, Republic of Korea; 7 Department of Health Administration, College of Health Science, Dankook University, Cheonan-si, Republic of Korea; 8 Department of Biomedical Engineering, College of Medicine, Dankook University, Cheonan-si, Republic of Korea; 9 Department of Psychiatry and Behavioral Sciences, Boston Children's Hospital, Boston, MA, United States; 10 Department of Psychiatry, Harvard Medical School, Harvard University, Boston, United States; 11 McLean Hospital, Belmont, MA, United States; 12 Department of Psychiatry, Dankook University Hospital, Cheonan-si, Republic of Korea

**Keywords:** depression, anxiety, digital phenotyping, mobile sensing, wearable devices, PRISMA, Preferred Reporting Items for Systematic Reviews and Meta-Analyses

## Abstract

**Background:**

The COVID-19 pandemic has intensified mental health issues globally, highlighting the urgent need for remote mental health monitoring. Digital phenotyping using smart devices has emerged as a promising approach, but it remains unclear which features are essential for predicting depression and anxiety.

**Objective:**

This study aimed to identify the types of features collected through smart packages—integrated systems combining smartphones with wearable devices such as Actiwatches, smart bands, and smartwatches—and to determine which features should be considered essential for mental health monitoring based on the type of device used.

**Methods:**

A systematic review was conducted. Searches were performed across Web of Science, PubMed, and Scopus on February 5, 2025. Inclusion criteria comprised quantitative studies involving adults (≥19 years) using smart devices to predict depression or anxiety based on passive data collection. Studies focusing solely on smartphones or qualitative designs were excluded. Risk of bias was assessed using the Mixed Methods Appraisal Tool and the Quality Criteria Checklist. Data were synthesized descriptively, and the relative contribution of each feature was further assessed by calculating coverage (proportion of studies using a feature) and importance among used (proportion identifying it as important when used). These metrics were visualized in quadrant-based scatter plots to identify consistently important features across devices.

**Results:**

From 1382 records, 22 studies across 11 countries were included. The overall synthesis identified a core feature package—accelerometer, steps, heart rate (HR), and sleep. Device-specific analyses revealed further nuances: in Actiwatch studies, accelerometer and activity were consistently important, but sleep features were rarely examined. In smart band studies, HR, steps, sleep, and phone usage were essential, while GPS, electrodermal activity (EDA), and skin temperature showed high importance when used, suggesting opportunities for broader adoption. In smartwatch studies, sleep and HR emerged as core features, whereas steps and accelerometer were widely used but often not identified as important.

**Conclusions:**

This systematic review identified a core feature package comprising accelerometer, steps, HR, and sleep that consistently contributes to mood disorder prediction across devices. At the same time, device-specific differences were observed: Actiwatch studies mainly emphasized accelerometer and activity but underused sleep features; smart bands highlighted HR, steps, sleep, and phone usage, with EDA, skin temperature, and GPS showing additional promise; and smartwatches most reliably leveraged sleep and HR, while steps and accelerometer were widely used yet less effective. These findings suggest that while a shared core set of features exists, optimizing digital phenotyping requires tailoring feature selection to the characteristics of each device type. To advance this field, improving data accessibility, particularly in smartwatch ecosystems, and adopting standardized reporting frameworks will be essential to enhance comparability, reproducibility, and future meta-analytic integration.

## Introduction 

### Background

The COVID-19 pandemic has intensified mental health issues and restricted access to health care services worldwide [1]. The World Health Organization (WHO) reported that the prevalence of major depressive disorder and anxiety increased by approximately 25% across the global population due to COVID-19 [1]. However, the pandemic also accelerated changes in health care systems [1,2]. Due to the infectious nature of COVID-19, many people experienced limitations in accessing essential health care services [3]. These experiences directly underscored the need for remote health care devices and systems, leading to a rapid increase in research on remote patient monitoring in the post-COVID era [4]. Among remote monitoring tools, smart packages—defined as a combination of a smartphone (serving as the primary data server) and a wearable device such as an Actiwatch, smart band, or smartwatch—have received particular attention. In this study, the term “wearable device” specifically refers to these 3 types of devices unless otherwise stated. This is largely due to their high portability, widespread adoption, compatibility with various platforms, and the development of sensors and applications capable of collecting diverse personal data [5].

Meanwhile, wearable devices offer much greater advantages in managing mental health conditions compared to other diseases. Physical illnesses, once diagnosed, generally follow a relatively predictable pathological progression, allowing periodic checkups at longer intervals, such as once a week or even less frequently [6]. In contrast, mental illnesses—particularly mood disorders such as depression and anxiety—are highly sensitive to real-world influences, including social, economic, and environmental factors [7,8]. As a result, they exhibit high variability even over short periods, making real-time monitoring essential and prediction difficult [9]. The challenge is that due to the high variability of mental health states, it is impossible to predict whether and when these fluctuations may escalate into extreme outcomes, including suicide.

This uncertainty has led to a growing demand for real-time monitoring. Traditional methods for diagnosing depression and anxiety rely on self-reports through interviews or questionnaires during clinical visits, but these approaches are limited by memory biases and the subjectivity of patients’ evaluations. Furthermore, they are unable to capture information about environmental and contextual factors influencing the onset of depressive symptoms in real time [10,11]. In addition, the clinical setting itself may influence patients’ behaviors and responses, making it difficult to accurately reflect their actual mental health status during in-person assessments [12]. As a result, due to the intrinsic variability of mental health conditions and the impossibility of direct real-world observation, clear physical and laboratory indicators, and biomarkers for conditions such as depression and anxiety remain elusive and largely hidden within a “black box.” 

As an alternative to addressing the lack of real-time longitudinal data, digital phenotyping using smart packages has emerged as a promising approach, driven by the need for continuous monitoring. This approach uses data collected from individuals’ daily lives to continuously monitor mental health status and enables more precise and refined assessments through advanced technological methodologies [13]. The growth of digital phenotyping has been largely fueled by the continuous global increase in smartphone users over the past several years [14], along with rapid advances in wearable devices such as smartwatches and smart bands, which now incorporate sensors that enable continuous data collection through their widespread adoption and compatibility.

The advancement of smartphones has enabled researchers to passively collect detailed information about users’ fine-grained activities, such as voice patterns, touchscreen interactions, and phone usage behaviors, as well as external activities such as movement and location (through GPS) and environmental data such as ambient light [1]. Furthermore, wearable devices, including Actiwatch, smart bands, and smartwatches, have recently expanded these capabilities by enabling the measurement of vital signals such as blood pressure, heart rate (HR), skin temperature, respiratory rate, and oxygen saturation [15,16]. Since users constantly carry these devices, they provide an advantage in tracking dynamic changes within individuals, particularly the highly fluctuating nature of mental states [17].

However, it remains unclear which features should be considered essential when designing digital phenotyping systems using smart packages. Each type of wearable device—Actiwatch, smart bands, and smartwatches—has distinct purposes, functionalities, and levels of data accessibility. Actiwatches are used mainly in research, smart bands focus on health tracking, and smartwatches serve as multifunctional consumer devices. These differences influence which features can realistically be collected and analyzed. Incorporating too many sensors and functions into a single device can lead to issues such as functional conflicts and increased battery consumption. In this regard, it is necessary to develop strategies that optimize predictive accuracy for mood disorders while minimizing the number of required sensors and features for each type of wearable device.

Recent systematic reviews have primarily focused on smartphone-based sensors and features [18,19], examined whether digital phenotypes can predict the worsening of psychiatric symptoms [1], or provided general overviews of the current state of research in this field [20]. However, these reviews have largely overlooked the specific roles of wearable devices such as Actiwatches, smart bands, and smartwatches in mental health prediction. Our review uniquely contributes by systematically identifying which features or sensors derived from these wearable devices are most effective for predicting depression and anxiety.

### Objectives

Therefore, this study aims to systematically identify features collected through smart packages—integrated systems of smartphones with wearable devices such as Actiwatches, smart bands, or smartwatches—and to determine which features are essential for mental health monitoring, depending on the device type.

## Methods

### Study Design and Search Strategy

#### Overview

This study used a systematic review approach to answer the research question. The databases searched were Web of Science, PubMed, and Scopus. The review was conducted in compliance with the PRISMA (Preferred Reporting Items for Systematic Reviews and Meta-Analyses) guidelines [21]. The completed PRISMA checklists are provided in Checklists1  2. In addition, the study protocol was guided by and registered on the Open Science Framework [22]. All records were managed in RefWorks (ProQuest).

#### Definition of Keywords

The study referred to the most relevant existing systematic review studies [1,23] to define the specific keywords. We used the following keywords: depressive mood, depressive symptoms, depression, mental health, affective disorder, anxiety, anxious mood, generalized anxiety disorder, digital phenotyping, digital phenotype, digital biomarker, digital footprint, smartwatch, smart band, mobile sensing, and passive sensing.

#### Search Query

We constructed database-specific search strings based on these keywords (Table 1) and searched all databases on February 5, 2025. To increase precision and efficiency, we used field tags and Boolean operators (AND, OR) to limit searches to titles, abstracts, and keywords. At this stage, only original studies were included: review studies, conference papers, book chapters, and editorials were excluded.

**Table 1.** Search strings used in academic databases.

Academic database	Search string
Web of Science	TS=(“depressive mood” OR “depressive symptoms” OR “depression” OR “mental health” OR “affective disorder” OR “anxiety” OR “anxious mood” OR “generalized anxiety disorder”) AND TS=(“digital phenotyping” OR “digital phenotype” OR “digital biomarker” OR “digital footprint” OR “smartwatch” OR “smartband” OR “mobile sensing” OR “passive sensing”)
PubMed	(“depressive mood” [Title/Abstract] OR “depressive symptoms” [Title/Abstract] OR “depression” [MeSH Terms] OR “depression” [Title/Abstract] OR “mental health” [MeSH Terms] OR “mental health” [Title/Abstract] OR “affective disorder” [Title/Abstract] OR “anxiety” [MeSH Terms] OR “anxiety” [Title/Abstract] OR “anxious mood” [Title/Abstract] OR “generalized anxiety disorder” [MeSH Terms] OR “generalized anxiety disorder” [Title/Abstract]) AND (“digital phenotyping” [Title/Abstract] OR “digital phenotype” [Title/Abstract] OR “digital biomarker” [Title/Abstract] OR “digital footprint” [Title/Abstract] OR “smartwatch” [Title/Abstract] OR “smartband” [Title/Abstract] OR “mobile sensing” [Title/Abstract] OR “passive sensing” [Title/Abstract])
Scopus	(TITLE-ABS-KEY(“depressive mood”) OR TITLE-ABS-KEY(“depressive symptoms”) OR TITLE-ABS-KEY(“depression”) OR TITLE-ABS-KEY(“mental health”) OR TITLE-ABS-KEY(“affective disorder”) OR TITLE-ABS-KEY(“anxiety”) OR TITLE-ABS-KEY(“anxious mood”) OR TITLE-ABS-KEY(“generalized anxiety disorder”)) AND (TITLE-ABS-KEY(“digital phenotyping”) OR TITLE-ABS-KEY(“digital phenotype”) OR TITLE-ABS-KEY(“digital biomarker”) OR TITLE-ABS-KEY(“digital footprint”) OR TITLE-ABS-KEY(“smartwatch”) OR TITLE-ABS-KEY(“smart band”) OR TITLE-ABS-KEY(“mobile sensing”) OR TITLE-ABS-KEY(“passive sensing”))

#### Definition of Population, Intervention, Comparison, Outcomes, and Study

Defining the population, intervention, comparison, outcomes, and study (PICOS) design is an essential strategy in systematic reviews to ensure a structured and focused approach for achieving specific findings. PICOS structures research questions across these 5 parameters. In this study, we limited the target population to adults aged 19 years or older because adolescents and children differ from adults in symptoms, daily schedules, meal routines, and overall living environments.

For the intervention, we included studies using smartphones, Actiwatch devices, smartwatches, and smart bands. For comparisons, we did not impose restrictions on the presence of a comparison group but excluded theoretical studies. Regarding outcomes, we included only studies on mental health, primarily focusing on depression and anxiety (Table 2). Studies unrelated to either were excluded. We also included only quantitative studies. Furthermore, gray literature sources and conference proceedings were not considered. Only peer-reviewed journal studies published in English were eligible for inclusion.

**Table 2.** Population, intervention, comparison, outcomes, and study (PICOS) design of this study.

Parameter	Inclusion criteria	Exclusion criteria
Participants	Adults aged ≥19 years	Adolescents <19 years
Interventions	Smartphone, Actiwatch, smartwatch, and smart band	Smartphone-exclusive sensing studies
Comparisons	Comparison not required	Theoretical studies
Outcomes	Mental health (depression and anxiety)	Not related to depression or anxiety
Study design	Quantitative studies	Qualitative studies, all types of review papers, and meta-analyses

#### Additional Eligibility Criteria

This study excluded studies using smartphone-exclusive sensing. This decision was made for 3 reasons. First, this review aimed to examine smart packages—integrated systems combining smartphones with wearable devices—which better reflect the core concept of digital phenotyping by leveraging both behavioral and physiological signals. Second, smartphones alone lack the capability to capture key biological signals (eg, HR, skin temperature, and EDA) due to their nonwearable nature and lack of direct contact with the body, limiting their relevance to physiological monitoring. Including such studies would have introduced heterogeneity and reduced comparability with wearable-integrated studies, potentially weakening the focus and reliability of our synthesis.

#### Screening Based on Title and Abstract

Three authors, Seungjin Lee, DYK, and Sujin Lee, independently screened all literature retrieved from academic databases by title and abstract, following the predetermined PICOS criteria. After completing the independent screening, the authors compared their selection lists. At this stage, the other authors (HWJ, OK, IL, and JJL) reviewed the lists using RefWorks’ sharing function and discussed whether to include or exclude studies from the mismatched selections. HWJ and JJL acted as the primary arbiters in cases of disagreement.

#### Two Rounds of Full-Text Screening

Three authors, Seungjin Lee, DYK, and Sujin Lee, conducted the full-text screening of studies that passed the title and abstract review stage. Given the large number of studies and the limitations of the PICOS criteria in precisely selecting studies aligned with the aim of this research, the authors proceeded as follows: We conducted 2 rounds of full-text screening. Both rounds focused on studies that used passively collected smartphone data, such as GPS location tracking, communication app usage, and detection of nearby Bluetooth devices, obtained from Android (Google) or iOS (Apple) devices. Studies that combined smartphone-based passive data with other sources—particularly data from smartwatches and smart bands—were included. The selected studies collected passive data continuously during participants’ daily routines or in laboratory settings. In addition, studies examining the relationship between passively collected smartphone data and depressive symptoms or clinically diagnosed depressive disorders were considered. All the authors jointly discussed and finalized study selection, with HWJ and JJL serving as primary arbiters.

### Data Extraction 

For each included study, we extracted detailed information on the types of features collected via smart devices (eg, accelerometer, HR, and peripheral capillary oxygen saturation [SpO₂]), the devices used, statistical analysis methods, and whether each feature was explicitly identified as important for predicting depression or anxiety. Three reviewers (Seungjin Lee, DYK, and Sujin Lee) independently extracted data, and discrepancies were resolved through discussion with a fourth reviewer (HWJ). In cases where the original study did not provide a full variable list, only those features explicitly described in the text, tables, or figures were included.

To facilitate data synthesis, we classified each study based on its method for determining feature importance—such as predictive performance metrics, statistical significance tests, or the authors’ narrative emphasis. This approach enabled cross-study comparisons of how features were selected and highlighted for depression or anxiety prediction. To address potential heterogeneity across studies, we descriptively stratified the data based on device type (eg, smartphone, Actiwatch, and smart band), population characteristics (eg, clinical vs community), and analytic approaches (eg, traditional statistics vs deep learning). Studies were grouped accordingly during data extraction, and the synthesized feature importance was compared across these strata. The first series in the “List of Feature Categories in the Selected Studies” subsection was constructed to visually support these comparisons.

### Synthesizing Methods

To quantify and compare the relative importance of features across studies, we synthesized the extracted data from the first series: synthesized features of Actiwatch (SFA), synthesized features of smart band (SFB), synthesized features of smartwatch (SFW), and total synthesized features (TSFs). For each device type, we calculated (1) coverage, defined as the proportion of studies using a given feature among all included studies using that device type, and (2) importance among used, defined as the proportion of studies identifying the feature as important among those that used it. To facilitate interpretation across studies, we mapped these indices onto quadrant-based scatter plots, generated for all devices combined and for each device type separately. This visualization helped identify features that were both frequently used and consistently important, thereby providing a structured basis for determining core features across devices. Plots were generated using Python (version 3.13; Python Software Foundation) with Matplotlib.

### Ethical Considerations

This systematic review analyzed data that are publicly available and, therefore, did not require ethics approval. The results of this review will be shared through various platforms, including peer-reviewed journals, webinars, stakeholder meetings, digital media, and conferences. The review was preregistered with the Open Science Framework (OSF) under the registration number “nz7k8.”

## Results

### Flow Diagram

**Figure 1.**
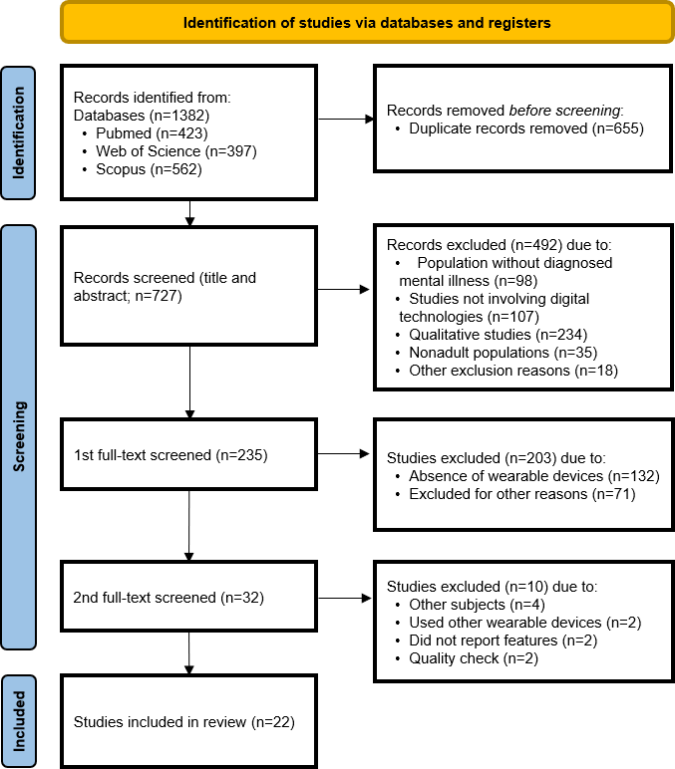
Flow diagram of study selection process based on the PRISMA (Preferred Reporting Items for Systematic Reviews and Meta-Analyses) guidelines. A total of 1382 records were identified: PubMed (n=423), Web of Science (n=397), and Scopus (n=562).

Figure 1 presents the screening and selection process of this systematic review. A total of 1382 records were retrieved through 3 databases: PubMed (n=423), Web of Science (n=397), and Scopus (n=562). After removing 655 duplicates, 727 unique records remained. Three reviewers independently screened the titles and abstracts. As a result, 492 studies were excluded: population without diagnosed mental illness (n=98), studies not involving digital technologies (n=107), and qualitative studies (n=234). The remaining 235 studies underwent a 2-stage full-text screening, also conducted independently by the 3 reviewers. In the first round, 203 studies were excluded due to the absence of wearable devices (n=132) or other reasons (n=71). In the second round, 10 studies were excluded: unrelated subjects (n=4), other types of wearable devices (n=2), no reported features (n=2), and final quality check (n=2). After completing the screening process, 22 studies were included in the final review.

### Quality Assessment of the Selected Studies

During the final round of full-text review, all authors participated in a discussion meeting to assess the quality of the selected studies, and the results are summarized in Table 3. Although the Joanna Briggs Institute critical appraisal tools are widely used in systematic reviews, they provide design-specific checklists. For instance, Joanna Briggs Institute tools are designed to assess and compare studies within the same design type, such as randomized controlled trials, cohort studies, or case-control studies. Since the aim of this review was not to compare effect estimates within a single study design, and the selected studies included a variety of designs, we used the Mixed Methods Appraisal Tool and Quality Criteria Checklist—both of which are suitable for comprehensive evaluations of studies with qualitative and quantitative elements (MultimediaAppendices 1  2).

**Table 3.** Results of quality assessment using Mixed Methods Appraisal Tool (MMAT) and Quality Criteria Checklist (QCC).

Number	Reference	Year	MMAT	QCC	Final decision
1	Jacobson et al [24]	2019	Pass	Positive (+)	Included
2	Jacobson et al [25]	2019	Pass	Positive (+)	Included
3	Price et al [26]	2022	Pass	Positive (+)	Included
4	Aledavood et al [27]	2025	Pass	Positive (+)	Included
5	Anmella et al [28]	2023	Pass	Neutral (0)	Included
6	Zou et al [29]	2023	Pass	Positive (+)	Included
7	Pedrelli et al [30]	2020	Pass	Positive (+)	Included
8	Wang et al [31]	2018	Pass	Positive (+)	Included
9	Sano et al [32]	2018	Pass	Positive (+)	Included
10	Hong et al [33]	2024	Pass	Positive (+)	Included
11	Ahmed et al [34]	2022	Pass	Neutral (0)	Included
12	Price et al [35]	2023	Pass	Positive (+)	Included
13	Bai et al [36]	2021	Pass	Positive (+)	Included
14	Mahendran et al [37]	2019	Pass	Positive (+)	Included
15	Cho et al [38]	2020	Pass	Positive (+)	Included
16	Cho et al [39]	2019	Pass	Positive (+)	Included
17	Tazawa et al [40]	2020	Pass	Positive (+)	Included
18	Čermák et al [5]	2023	Pass	Positive (+)	Included
19	Zhang et al [41]	2025	Pass	Positive (+)	Included
20	Song et al [42]	2024	Pass	Positive (+)	Included
21	Narziev et al [43]	2020	Pass	Positive (+)	Included
22	Horwitz et al [44]	2023	Pass	Positive (+)	Included
23	Jacobson et al [45]	2021	Fail	Negative (–)	Excluded
24	Maruani et al [46]	2024	Fail	Negative (–)	Excluded

It is important to note that the primary purpose of these tools is not to exclude studies based solely on quality, but rather to provide qualitative insight into methodological rigor while retaining studies for synthesis. However, even under these inclusive criteria, 2 studies were excluded. Although Jacobson contributed several studies to the literature (Table 3), 1 study (Jacobson et al [45]) did not meet the quality criteria applied in this review. This should not be interpreted as an indication of poor quality overall, but rather as a reflection of misalignment with the review’s objectives. The main reason for exclusion was the lack of a clear feature list or explanation. The other excluded study was Maruani et al [46], which was removed because its dependent variable was based solely on clinician judgment, rather than using validated measurement scales.

### Summary of Selected Studies

A total of 22 studies were included in the final review. Table 4 summarizes the included studies, organized by device type: Actiwatch, smart band, and smartwatch. These studies were conducted across a wide range of countries, including Brazil, Norway, Finland, the United States, China, South Korea, India, Japan, the Czech Republic, the United Kingdom, and several multinational collaborations. The United States and South Korea were the most frequently represented countries, with 5 studies each. Notably, countries using Actiwatch devices did not overlap with those using smart bands or smartwatches.

In particular, neither the United States nor South Korea—in spite of their high publication counts—had any studies that used Actiwatch devices. This pattern may reflect variations in research infrastructure, funding availability, or longstanding preferences for specific device types. For instance, Actiwatch has traditionally been favored in European sleep research [25,27], while countries such as the United States and South Korea may lean toward smart bands or smartwatches due to broader commercial availability and stronger integration with consumer technologies. These regional trends suggest that national contexts influence device selection and, by extension, the types of features prioritized in mental health prediction research. Recognizing these patterns may guide future multinational collaborations and the development of context-sensitive standardized protocols.

In addition, notable differences were observed in the characteristics of the target populations depending on the device type. All studies using Actiwatch devices primarily targeted patients with clinically diagnosed psychiatric disorders. In contrast, studies involving smart bands or smartwatches recruited more diverse populations, including patients with mental health conditions, college students, community-dwelling individuals, the general population, older adults, caregivers, and even medical interns. This may reflect that Actiwatch devices are designed for clinical and research purposes rather than consumer use and are therefore typically used in controlled settings with patient populations. Smart bands and smartwatches, on the other hand, are commercially available, user-friendly, and widely adopted by the general public, making them suitable for naturalistic studies across a range of populations.

The statistical methodologies used in the included studies were predominantly artificial intelligence (AI)–based, with 16 studies using machine learning and 2 using deep learning approaches, while 5 studies relied on traditional statistical techniques. Notably, recent work such as Song et al [42] and Aledavood et al [27] used mixed models and multilevel analysis. Thus, the predominance of AI-based methods should not be interpreted as evidence of a temporal shift in analytic approaches.

Although differences were observed in target populations and analytic approaches across device types, stratified analysis indicated that these variations did not affect the lists of important features or reveal systematic tendencies across groups (Multimedia Appendix 3).

**Table 4.** Summary of studies using wearable devices for digital phenotyping of mood disorders.

Study	Device type	Nation	Authors	Target population (sample size)	Statistical methodology	Data collection period	Device
1	Actiwatch	Brazil	Jacobson et al [24]	Patients with MDD^a^ (n=15)	Machine learning	1 week	Actiwatch-L
2	Actiwatch	Norway	Jacobson et al [25]	Patients with mood disorders (bipolar, depression; n=23) and healthy controls (n=32)	Machine learning	Up to 2 weeks (minimum 19,299 minutes)	Actiwatch
3	Actiwatch	Norway	Price et al [26]	Patients with schizophrenia (n=22), with major depression (n=23), and healthy controls (n=32)	Machine learning	2 weeks (1 week of data used)	Actiwatch
4	Actiwatch	Finland	Aledavood et al [27]	Patients with bipolar (n=21), depression (n=76), borderline personality disorder (n=24), and healthy controls (n=30)	Traditional statistics (Kaplan-Meier survivor test, log-rank test, and linear mixed models)	2 weeks (active phase) + up to 1 year (passive phase)	Actiwatch (Philips Actiwatch 2)
5	Smart band	Spain	Anmella et al [28]	Patients with mood disorders (bipolar, depression; n=12) and healthy controls (n=7)	Deep learning model	Up to 3 sessions per person × 48 hours =~1512 hours total	Smart band (Empatica E4)
6	Smart band	China	Zou et al [29]	Patients with MDD (n=245)	Deep learning model	2 weeks passive sensing + 12-week follow-up	Smart band
7	Smart band	United States	Pedrelli et al [30]	Patients with MDD (n=31)	Machine learning	9 weeks	Smart band (Empatica E4)
8	Smart band	United States	Wang et al [31]	College students (n=83)	Traditional statistics and machine learning	9 weeks	Smart band (Microsoft Band 2)
9	Smart band	United States	Sano et al [32]	College students (n=201)	Machine learning	1 month	Actiwatch (Motion Logger) and smart band (Q Sensor Affectiva)
10	Smart band	South Korea	Hong et al [33]	Inpatients diagnosed with schizophrenia or mood disorders (n=191)	Machine learning	~21 days	Smart band (URBAN HR)^b^
11	Smart band	Multicountry (China dataset)	Ahmed et al [34]	Community-dwelling Chinese patients with clinical depression (n=87)	Machine learning	5 days (9 AM-11 PM daily)	Smart band (Psychorus band)
12	Smart band	United States	Price et al [35]	General population in the United States (n=939)	Machine learning	1 year	Smart band (Fitbit)
13	Smart band	China	Bai et al [36]	Outpatients with MDD (n=334)	Machine learning	12 weeks	Smart band (Mi Band 2)
14	Smart band	India	Mahendran et al [37]	General population who reported mood-related symptoms (n=450)	Machine learning	7 days	Smart band (Mi Band 2)
15	Smart band	South Korea	Cho et al [39]	Patients with MDD (n=18), with bipolar 1 (n=18), and bipolar 2 (n=19)	Machine learning	2 years (used 2003 days of data for analysis)	Smart band (Fitbit Charge HR or 2)
16	Smart band	South Korea	Cho et al [38]	Patients with mood disorder (using CRM^c^ 10 and not using CRM 33; n=43)	Traditional statistics (generalized linear model)	1 year	Smart band (Fitbit Charge HR)
17	Smart band	Japan	Tazawa et al [40]	Patients with depression (n=45) and healthy controls (n=41)	Machine learning	Up to 9 days (mean 4.2 days)	Smart band (Silmee W20)
18	Smartwatch	Czech Republic	Čermák et al [5]	Patients affected by MDD and treated with trazodone OAD^d^ monotherapy (n=10)	Descriptive analysis	8 weeks	Smartwatch (Withings Move ECG)
19	Smartwatch	United Kingdom	Zhang et al [41]	United Kingdom–based general population (n=10,129)	Machine learning	2 weeks	Smartwatch (Fitbit)
20	Smartwatch	South Korea	Song et al [42]	Adults (n=25) older than 65 years, and their community caregivers from community center	Multilevel analysis	6 weeks	Smartwatch (Fitbit)
21	Smartwatch	South Korea	Narziev et al [43]	College students (n=20)	Machine learning	4 weeks	Smartwatch (Galaxy S3)
22	Smartwatch	United States	Horwitz et al [44]	First-year medical interns from over 300 residency hospitals (n=2459)	Machine learning	13 weeks	Smartwatch (Fitbit Charge 4)

a MDD: major depressive disorder.

b HR: heart rate.

c CRM: circadian rhythm metric.

d OAD: overall activity density.

Although the data collection periods varied widely across studies—from a minimum of 1 week to up to 2 years—some studies reported that the duration of data used for analysis did not fully align with the total period of data collection [25,27]. While not all studies explicitly mentioned this, it is reasonable to assume that most digital phenotyping studies based on wearable or smartphone devices involve discrepancies between the overall data collection period and the time frame included in the final analysis.

This is largely due to the inherent characteristics of digital data collection, which is susceptible to device shutdowns, communication failures, server issues, improper use, and noncompliance. Furthermore, because these devices collect data continuously during everyday life, the volume of data can be overwhelming, and researchers often need to extract optimized segments for analysis. Consequently, such methodological considerations likely account for the differences observed between collection and analysis periods in many studies.

### List of Feature Categories in the Selected Studies

The key findings of this study are summarized in Tables5^7^, which present the features used in studies using smart packages, defined as combinations of smartphones and either Actiwatch devices, smart bands, or smartwatches. Each row corresponds to a distinct feature, and each column represents a study categorized by device type. The numbers in the columns correspond to those in Table 4, which identify specific studies.

The final column for each device category presents a synthesized feature summary: SFA, SFB, and SFW. In addition, a TSF column in Table 7 is included to represent the combined results across all devices. Each value in these synthesized columns indicates the ratio of studies in which the corresponding feature was not only used but also identified as important. For example, a value of 1/2 indicates that the feature was used in 2 studies, but only 1 of them identified it as an important predictor.

In the table, an open circle (○) indicates that the corresponding feature was used, while a solid circle (●) denotes that the feature was identified as an important predictor of mental health outcomes such as depression or anxiety, based on feature importance metrics reported in the respective studies. For studies that applied AI or machine learning techniques and reported feature importance, important predictors were identified based on those metrics. In contrast, for studies that either used traditional statistical methods or applied machine learning but did not report feature importance, features were considered important if the authors emphasized them through tables, figures, or narrative interpretation of the results. A more detailed explanation of data extraction methods and feature importance determination criteria is provided in Multimedia Appendix 4.

Although all included studies focused on digital phenotyping, the specific features extracted varied considerably—even within the same categories. To enhance the table’s readability and interpretability, we therefore grouped similar features into broader feature classes. Through the review of the selected studies, the extracted features were classified into the following groups: blood volume pulse (BVP), HR, interbeat intervals, SpO₂, accelerometer, caloric consumption, sedentary minutes, activity, steps, motion magnitude, electrodermal activity (EDA), skin temperature, communication-related features (call log, SMS text messaging, phone usage, and app usage), sleep, light exposure, and GPS. Among them, HR, interbeat intervals, and SpO₂ are derived from BVP signals, while caloric consumption, sedentary minutes, activity, steps, and motion magnitude are derived from accelerometer data.

A more detailed summary of the studies and description of features is provided in Multimedia Appendix 5.

**Table 5.** Features used across studies on wearable-based mood monitoring by device type.

Feature list		Actiwatch				
		1	2	3	4	SFA^a^
BVP^b^						0/0
HR^c^						0/0
Interbeat intervals						0/0
SpO₂^d^						0/0
Accelerometer		●		●	●	3/3
Caloric consumption						0/0
Sedentary minutes						0/0
Activity			●		○	1/2
Steps						0/0
Motion magnitude						0/0
EDA^e^						0/0
Skin temperature						0/0
Sleep					○	0/1
Call logs					●	1/1
SMS					○	0/1
Phone usage					○	0/1
App usage					○	0/1
Light exposure		●				1/1
GPS					○	0/1
Feature importance extraction method		PR^f^	AE^g^	AE	*P* ^h^	—^i^

a SFA: synthesized feature summary for Actiwatch.

b BVP: blood volume pulse.

c HR: heart rate.

d SpO₂: peripheral capillary oxygen saturation.

e EDA: electrodermal activity.

f PR: predictive performance.

g AE: author emphasis.

h *P*: *P* value from traditional statistics.

i Not available.

**Table 6.** Features used across studies on wearable-based mood monitoring by device type (continued).

Feature list		Smart band													
		5	6	7	8	9	10	11	12	13	14	15	16	17	SFB^a^
BVP^b^		○													0/1
HR^c^		●		●	○		●	●		●	●	●	○	○	7/10
Interbeat intervals		○		●											1/2
SpO₂^d^											○				0/1
Accelerometer		●		○		●	○	●			●				4/6
Caloric consumption							○				○			●	1/3
Sedentary minutes															0/0
Activity					●				○			○	○	●	2/5
Steps			○				●		●	●		●	○	●	5/7
Motion magnitude				○											0/1
EDA^e^		●		●		●		●							4/4
Skin temperature		●				●								○	2/3
Sleep			○	○	●	●	○		●	●		○	○	●	5/10
Call logs			○	○		●				●					2/4
SMS				○		○									0/2
Phone usage			●	●	●	●				○					4/5
App usage			●	○						○					1/3
Light exposure												●	○	○	1/3
GPS				●			●								2/2
Feature importance extraction method		PR^f^	AE^g^	PR	P^h^	PR	PR	PR	PR	AE	PR	P	PR	PR	—^i^

a SFB: synthesized feature summary for smart bands.

b BVP: blood volume pulse

c HR: heart rate

d SpO₂: peripheral capillary oxygen saturation.

e EDA: electrodermal activity.

f PR: predictive performance.

g AE: author emphasis.

h P indicates *P* value from traditional statistics.

i Not available.

**Table 7.** Features used across studies on wearable-based mood monitoring by device type (continued).

Feature list		Smartwatch						Total
		18	19	20	21	22	SFW^a^	TSF^b^
BVP^c^							0/0	0/1
HR^d^			●	○	●	○	2/4	9/14
Interbeat intervals							0/0	1/2
SpO₂^e^							0/0	0/1
Accelerometer		●		○	○		1/3	8/12
Caloric consumption		●	○				1/2	2/5
Sedentary minutes			○				0/1	0/1
Activity		○	○			○	0/3	3/10
Steps		○	●	○	●	○	2/5	7/12
Motion magnitude					●		1/1	1/2
EDA^f^							0/0	4/4
Skin temperature							0/0	2/3
Sleep		●	●	●	●	○	4/5	9/15
Call logs					○		0/1	2/5
SMS							0/0	0/3
Phone usage							0/0	4/6
App usage					○		0/1	1/5
Light exposure							0/0	2/4
GPS							0/0	2/3
Feature importance extraction method		AE^g^	PR^h^	*P* ^i^	AE	—^j^	—	—

a SFW: synthesized feature of smartwatch.

b TSF: total synthesized feature across all devices.

c BVP: blood volume pulse.

d HR: heart rate.

e SpO₂: peripheral capillary oxygen saturation.

f EDA: electrodermal activity.

g AE: author emphasis.

h PR: predictive performance.

i P indicates *P* value from traditional statistics.

j Not available.

### Synthesized Findings

**Figure 2.**
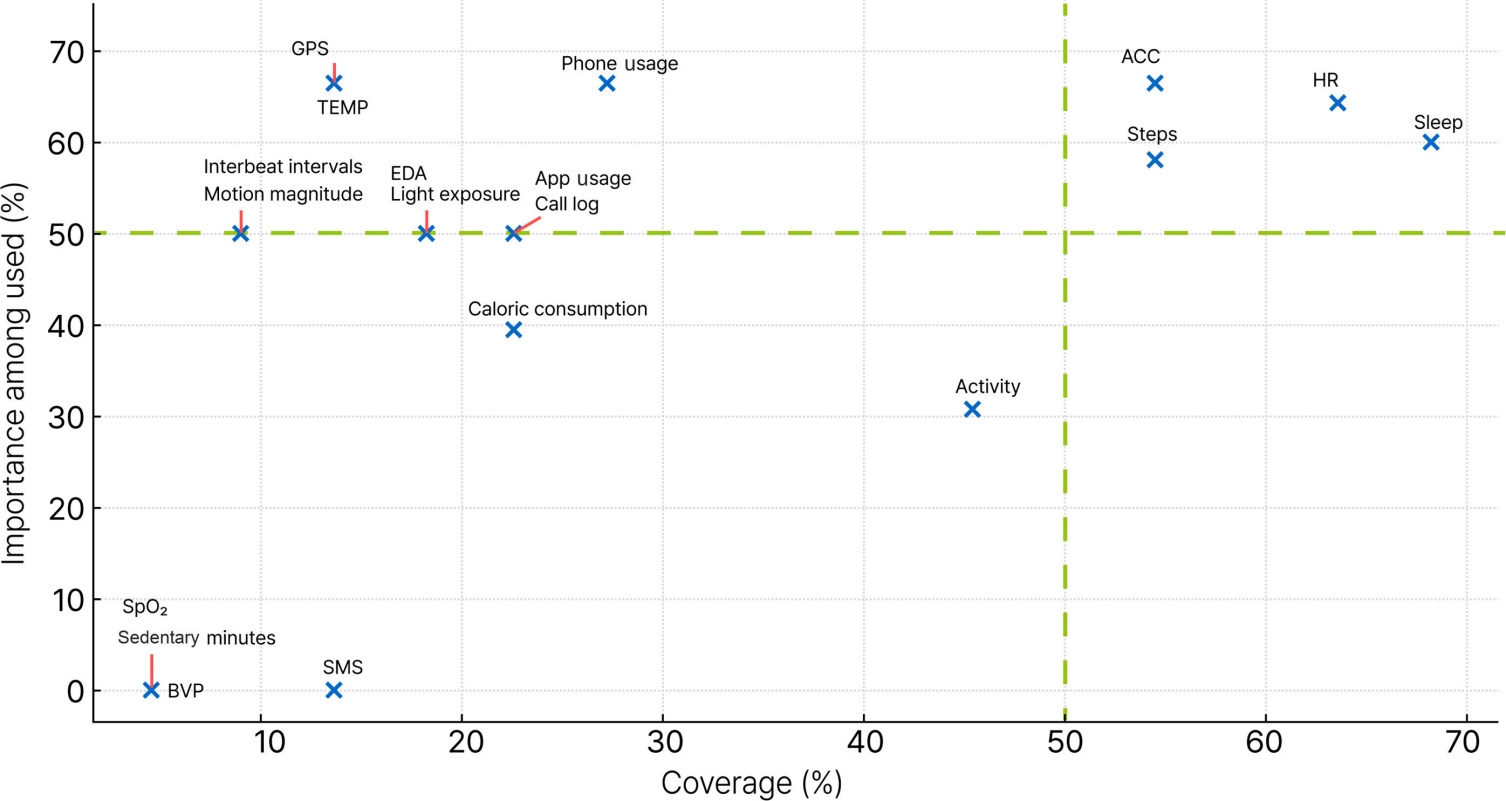
Feature coverage and importance of all devices. Coverage is expressed as the percentage of studies using each feature, and importance among those indicates the proportion of studies that identified the feature as important among those that used it. The graph was generated using Python (Matplotlib). ACC: accelerometer; BVP: blood volume pulse; EDA: electrodermal activity; HR: heart rate; SpO₂: peripheral capillary oxygen saturation; TEMP: skin temperature.

**Figure 3.**
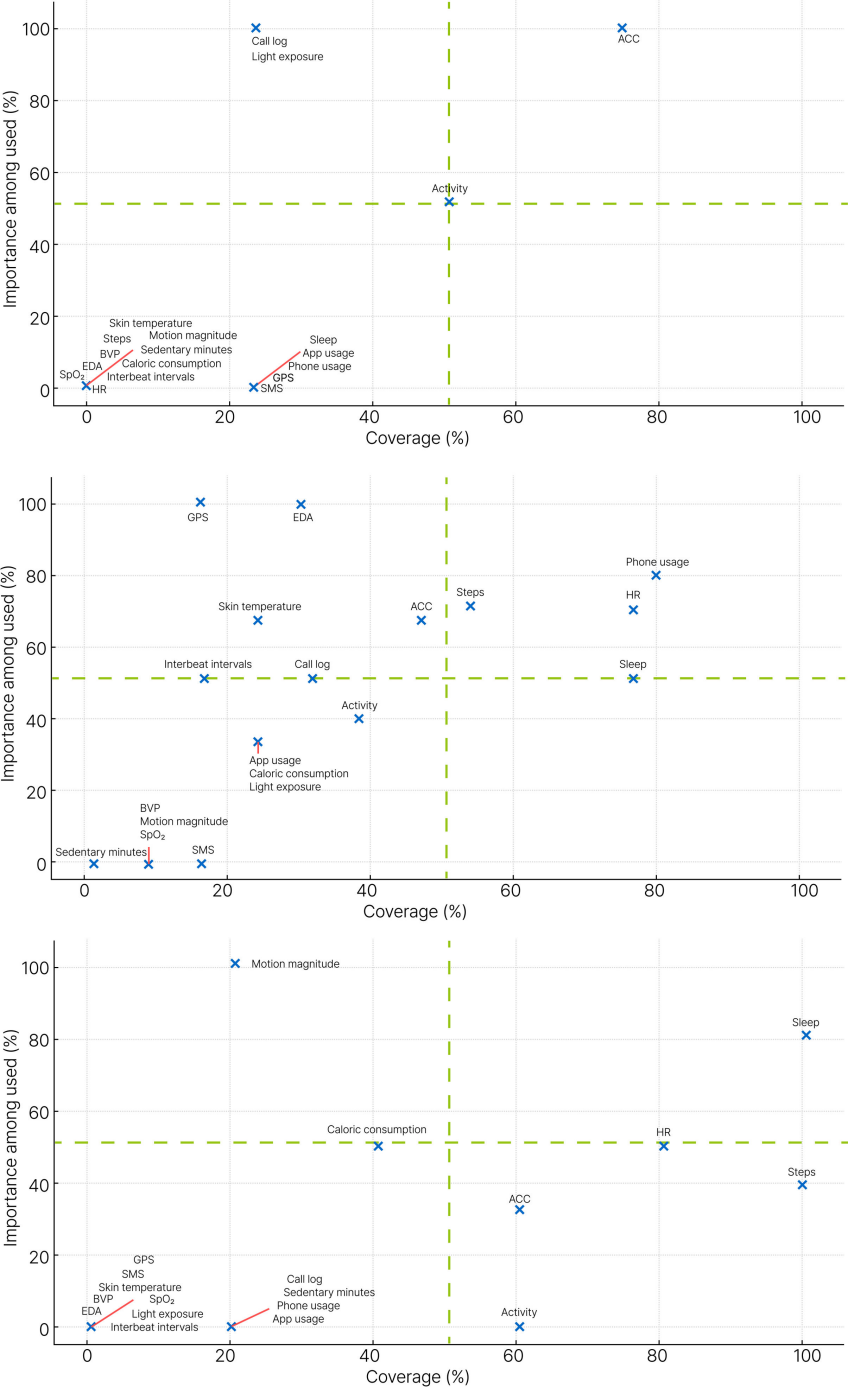
Feature coverage and importance by device type. The upper graph presents the feature coverage and importance of the Actiwatch, the middle graph shows those of the smart band, and the bottom graph represents those of the smartwatch. Coverage is expressed as the percentage of studies using each feature, and importance among those used indicates the proportion of studies that identified the feature as important among those that used it. The graph was generated using Python (Matplotlib). ACC: accelerometer; BVP: blood volume pulse; EDA: electrodermal activity; HR: heart rate; SpO₂: peripheral capillary oxygen saturation.

The synthesized feature summaries from Tables5^7^ (SFA, SFB, SFW, and TSF) were used to calculate coverage and importance among the used, as previously defined. The calculation results are provided in Multimedia Appendix 6. Visualizations of the relationship between coverage and importance used for each device are shown in Figures2  3. Figure 2 presents the results for all devices, and Figure 3 shows the results for each device type separately.

In Figure 2, Accelerometer, HR, steps, and sleep were located in the first quadrant (50% or higher for both coverage and importance among the used), indicating that these features were both frequently used and identified as important in previous studies. The second quadrant includes features with low coverage but high importance when used, such as GPS, skin temperature, phone usage, motion magnitude, light exposure, app usage, interbeat intervals, EDA, and call logs. The third quadrant represents features with both low coverage and low importance, including caloric consumption, activity, SMS text messaging, BVP, sedentary minutes, and SpO₂. No features were positioned in the fourth quadrant (high coverage but low importance).

However, Figure 2 does not account for differences by device type. Figure 3 provides device-specific coverage–importance plots. For Actiwatch, accelerometer and activity were in the first quadrant, showing high use and high importance, while call logs and light exposure were in the second quadrant, indicating lower usage but consistently high importance when used. The remaining features were all in the third quadrant. For smart bands, phone usage, HR, steps, and sleep were in the first quadrant, while GPS, EDA, skin temperature, accelerometer, interbeat interval, and call logs were in the second quadrant. The remaining features were positioned in the third quadrant. For smartwatches, only sleep and HR were in the first quadrant, while motion magnitude and caloric consumption were in the second quadrant. Notably, Steps, accelerometer, and activity appeared in the fourth quadrant, meaning they were widely used but had a relatively low proportion of studies identifying them as important.

While categorizing features into these 4 quadrants can facilitate intuitive interpretation, a strictly dichotomous reading should be avoided. For example, although smart bands and smartwatches are classified quite differently from the 4-quadrant perspective, their accelerometer, HR, sleep, and steps features exhibit broadly similar distributions.

## Discussion

### Principal Findings

This systematic review identified the most commonly reported and potentially important features in digital phenotyping studies using smartphone-wearable device packages, revealing that the key features varied depending on the type of wearable device used. These differences likely reflect the distinct sensor configurations and primary functions of each device type. The primary results were derived from Figures2  3.

The systematic classification and visualization of features revealed distinct patterns of feature usage in the literature. The all-devices synthesis further identified a core feature package that digital phenotyping studies should prioritize: accelerometer, HR, steps, and sleep, which cluster in the first quadrant of Figure 2. This comprehensive approach is particularly valuable when a device type has relatively few studies, such as the Actiwatch. For example, although existing Actiwatch research highlights only accelerometer and derived activity features as major predictors (Figure 3), this should not be taken as the definitive scope for future research. Figure 2 suggests that Actiwatch-based studies should consider incorporating additional features that have proven important in other device contexts, such as HR, steps, and sleep, which have not yet been examined in Actiwatch research.

The device-specific plots and the quadrant framework help investigators quickly identify features likely to contribute to depression or anxiety prediction, while also providing justification for pruning less informative features (Figure 3). Device-level inspection is necessary because sensor suites, embedded feature-generation algorithms, and data-access policies differ across device types. In this scheme, features in quadrants 1 and 2 are judged important when used and therefore warrant inclusion in future models when feasible. In contrast, features accumulating in quadrants 3 and 4 tend not to be important when they are used and can be considered candidates for exclusion, subject to study aims and context.

By device, patterns diverged. In studies using Actiwatch devices, accelerometer and derived activity features most often fell into the high-use, high-importance region (Figure 3). However, sleep was seldom analyzed even though it can be estimated from time-stamped accelerometer and light data using existing sleep scoring algorithms. Incorporating screen-use features to refine sleep windows may further improve inference within smart-package designs. Together, these observations support more systematic sleep computation in future Actiwatch work.

Smart bands were the most widely used device type and generally showed good performance. The results also indicate clear headroom for further progress. In Figure 3, smart bands have more features than other devices in quadrant 2, meaning several features performed well when used but are not yet widely adopted. On this basis, skin temperature, accelerometer, interbeat interval, call log, and especially GPS and EDA are recommended for consideration in future smart band studies.

In smartwatch-based studies, the number of papers was similar to that for Actiwatch, but the feature mix was more diverse, though narrower than for smart bands. Even so, many added features were not identified as important (Figure 3). For example, in Song et al [42], only sleep among 4 tested features was important, and in Horwitz et al [44], none of the 4 features were important (Table 7). Notably, steps were used in all smartwatch studies (coverage=100%), yet only 40% of those studies identified steps as an important predictor. This pattern is consistent with platform and usage differences that can affect data fidelity and downstream importance estimates.

### Usage Contexts of Each Feature

Figures2  3 have the advantage of allowing an intuitive understanding of the usage status and outcomes of each feature; however, since they use the proportion-based metrics of coverage and importance among those used, it is difficult to determine the exact number of studies in which each feature was used, in which it was identified as important, and the specific contexts in which it was used. This section, therefore, draws on the synthesized feature summary for each feature in Tables5^7^ (particularly TSF) to provide these details. The explanation follows the order of the feature list presented in Table 5. 

Among the sensor-derived features, BVP, which originates from photoplethysmography sensors, was rarely used as a standalone variable (TSF=0/1; Table 7). Instead, it was most commonly used in the form of its derived variable, HR (TSF=9/14). Across studies, HR was further elaborated into a variety of subfeatures, including HRV, circadian rhythm amplitude, acrophase, first-last heartbeat interval, as well as the mean, median, and SD of heartbeat [30,33,39]. Similarly, interbeat intervals can be regarded as derivatives of HR. However, because 2 studies (studies 5 and 7; [28,30]) explicitly distinguished and used interbeat intervals as separate analytical features (TSF=1/2), they are presented as independent items in Tables5^7^. BVP and its related features were frequently used in studies involving smart bands and smartwatches, but they have not been examined in studies using Actiwatch devices due to the absence of a photoplethysmography sensor [47].

Meanwhile, although SpO₂ is also derived from photoplethysmography sensors [48], it differs in its analytical role. SpO₂ was used in only 1 study (TSF=0/1), and even in that case, it was not identified as an important predictor. While BVP was frequently used as a basis for generating HR and other physiological features, SpO₂ typically did not lead to the development of additional derived variables. The limited use of SpO₂ compared to BVP in digital phenotyping studies likely stems from the restricted variation and contextual relevance of oxygen saturation levels in everyday conditions, combined with the lower measurement accuracy of wrist-worn devices [49]. In most nonclinical settings, SpO₂ values tend to remain within a narrow and relatively uninformative range, making them less useful for behavioral or mental health predictions.

On the other hand, an accelerometer represents raw sensor data from which various features, such as caloric consumption, sedentary minutes, activity levels, steps, and motion magnitude, are derived. In some studies, accelerometer signals also contributed to the calculation of sleep-related features [50]. Compared to BVP or SpO₂, the accelerometer was frequently used as a standalone feature, with its raw signal or simplified metrics directly applied in analysis (TSF=8/12). Among accelerometer-derived variables, activity (TSF=3/10) and step count (TSF=7/12) were the most frequently used across studies. When either of these 2 features was included, the raw accelerometer signal was typically not used independently, suggesting that they often serve as substitutes for the raw data.

Interestingly, while accelerometer-derived features were commonly used across all device types, they were more likely to be identified as important in studies using Actiwatch and smart bands than in those using smartwatches. The accelerometer of SFW in the smartwatch category (Table 7) showed use in 1 of 3 studies. Its derived features—caloric consumption, sedentary minutes, activity, steps, and motion magnitude—were reported in 1 of 2 studies, 0 of 1 studies, 0 of 3 studies, 2 of 5 studies, and 1 of 1 study, respectively. Although Figure 3 shows 100% importance for caloric consumption and 50% for motion magnitude, these values are based on a few studies and may be overestimated. This difference may reflect variations in device usage context and data collection fidelity. Actiwatches and smart bands are primarily designed for passive, continuous monitoring with minimal user interaction. In contrast, smartwatches are multifunctional and affected by user behaviors—such as screen interaction, app use, and wearing time—which can introduce variability and noise into sensor signal quality.

Meanwhile, although EDA and skin temperature were used exclusively in studies involving smart bands, they demonstrated notable performance, with TSF values reported in 4 of 4 studies and 2 of 3 studies, respectively. Despite their demonstrated importance, these features were still not widely used in smart band studies, as they are positioned in the second quadrant of Figure 3 and were never used in any studies using Actiwatch or smartwatches. This absence can be attributed to different types of limitations associated with each device type. In the case of Actiwatch, the limitation is primarily due to hardware constraints, as most models do not include sensors for EDA or skin temperature measurement [24-26]. On the other hand, smartwatches, although they often include these sensors, present challenges in terms of data accessibility. Many commercially available smartwatch platforms restrict access to raw physiological data or apply proprietary preprocessing, making it difficult for researchers to extract and use detailed sensor-level information for analysis.

Sleep-related features were among the most frequently used (TSF=9/15), often in combination with HR, and demonstrated substantial contributions to predicting mood disorders, as indicated by their placement in the first quadrant of Figure 2. The process of sleep data collection and computation varied widely depending on the study, device, and available sensors, making it inherently complex. Sleep was typically inferred using accelerometer data to detect periods of minimal movement, often supplemented by ambient light levels and, in some cases, HR patterns [30,31]. The specific algorithms and sensor combinations differed across studies, reflecting variations in data availability and device capabilities. Like other feature categories, sleep features encompassed a wide range of variables, such as the sleep regularity index, mean, median, and SD of bedtime, sleep duration, and sleep efficiency [32].

Additional variables included binary indicators for sleep deprivation (eg, pulling an all-nighter), presleep use of internet-based or electronic media (eg, email, phone calls, SMS text messaging, Skype, chat, and online games), and pre-sleep personal interaction [32]. This diversity may partly explain why sleep features are often identified as important. Beyond frequency of use, their strong conceptual link to mental health also supports their value as predictive indicators. None of the studies using Actiwatch included sleep features, despite the fact that sleep can be inferred from time-stamped data such as light exposure, accelerometer activity, and phone usage—all of which can be collected via a smart package using Actiwatch. Considering this, future studies using Actiwatch devices should actively incorporate sleep-related features. These features can be derived from accelerometer-based activity data and ambient light exposure, analyzed with established sleep-wake scoring algorithms (eg, Cole-Kripke or Oakley). In addition, combining these data with complementary smartphone indicators, such as screen-on or off status or app usage patterns, may further improve the accuracy of sleep inference in smart package frameworks.

However, certain features, such as SMS text messaging (TSF=0/3) and app usage (TSF=1/5), showed relatively low contributions to mental health prediction, as reflected in their placement in the third quadrant of Figure 2. In the case of SMS text messaging, this may be explained by the shift in communication habits toward dedicated messaging applications such as Facebook Messenger, WhatsApp, Snapchat, and Instagram, which likely reduces both its usage frequency and relevance as a predictive feature. App usage was identified as important in only 1 study, conducted by Zou et al [29] in China. However, since this study examined very few other features, the observed importance of app usage may have been overestimated by the limited range of features considered.

Call log features yielded a moderate contribution (TSF=2/5), with highly diverse variables extracted. For instance, Pedrelli et al [30] derived more than 150 call-related features, including counts and durations of missed, dismissed, and responded incoming calls, as well as reached and unanswered outgoing calls, calculated over various time intervals such as night (midnight-6 AM), morning (6 AM-noon), afternoon (noon-6 PM), and evening (6 PM-midnight), along with corresponding statistical metrics such as mean, median, and SD. In comparison, phone usage, which typically refers to screen-on behavior, showed stronger performance (SFB=4/6). It was measured using variables such as screen-on duration, number of screen activations, and the mean and SD of screen-on intervals. However, both call log and phone usage features were used in only a limited number of studies involving Actiwatch or smartwatches.

Other smartphone-based features, such as light exposure (TSF=2/4) and GPS (TSF=2/3), were not widely used but demonstrated reasonable contributions when included. These features are often used in conjunction with sleep- or activity-related features [30,33]. Light exposure tended to be identified as an important feature in Actiwatch-based studies, while GPS showed similar importance in smart band–based studies (second quadrant, 100% importance among used). However, the small number of studies limits the strength of this evidence; therefore, further research is warranted.

### Implications and Future Research

Although all included studies involved smartphones, many did not incorporate smartphone-derived features, and even when such features were included, they were generally limited in scope and rarely emphasized as important predictors of mental health outcomes. Our sensitivity analysis showed that including or excluding smartphone-exclusive sensing studies did not substantially change the findings (Multimedia Appendix 7).

Notably, smartwatch-based studies did not use smartphone-derived features (call log, SMS text messaging, phone usage, app usage, light exposure, and GPS). This discrepancy may be attributable to differences in research designs and device ecosystems. Smart band–based studies often paired the device with smartphones, treating the smartphone as the primary platform for collecting behavioral data such as call logs and screen usage. In contrast, smartwatch-based studies tended to focus more on the wearable device itself, to assess its standalone capabilities. Many commercial smartwatches, particularly those using closed platforms such as Apple’s, restrict access to smartphone-linked usage data or manage such data within proprietary systems that limit external extraction. Our sensitivity analysis also revealed that all smartphone-exclusive sensing studies used Android phones and none used iOS devices (Multimedia Appendix 7).

As a result, smartphone-derived features are rarely incorporated into smartwatch-focused studies, even when the participant is concurrently using a smartphone. To address this limitation, future smartwatch-based research could consider using open-source devices (eg, devices running on the Android Open Source Project or custom firmware such as AsteroidOS), which allow direct access to raw sensor and smartphone-linked data. When open-source hardware is not feasible, researchers may instead leverage official or third-party application programming interfaces to access selected behavioral and physiological data—such as screen-on time, step count, or HR—while still adhering to privacy and platform constraints.

Finally, it is important to note that the composition of features varied across studies; therefore, the proposed results should not be overgeneralized. The feature selection process and data context in each study significantly influenced the observed predictive contributions. Therefore, this study does not argue that features with low predictive power should be excluded from data collection.

The essence of AI analyses, including machine learning and deep learning, lies in developing predictive algorithms that encompass a wide range of features. As will be further discussed in the “Limitations” section, the substantial heterogeneity in research designs and the types of features included across studies may introduce bias or distortion in the interpretation of results. Currently, there is no standardized framework for categorizing included features or detailed subfeatures, and thus, the reported importance of features should be interpreted with caution. Nevertheless, identifying the types of features that consistently emerge as important can provide valuable reference points for future research.

To enhance methodological transparency, reproducibility, and the overall robustness of future digital phenotyping studies, researchers must develop and adopt a standardized, domain-specific reporting framework. Such a framework, akin to the proposed Digital Assessment and Performance Reporting (DAPPER) checklist, would provide much-needed guidance, ensuring comprehensive reporting of study design, data collection, feature extraction, and analysis. This would not only improve the quality of individual studies but also facilitate more meaningful comparisons and syntheses across the rapidly evolving landscape of digital phenotyping research.

In addition, for future interventional applications of AI-based digital phenotyping tools, the CONSORT-AI (Consolidated Standards of Reporting Trials–Artificial Intelligence) extension offers a complementary reporting guideline. It addresses the unique challenges of reporting randomized clinical trials involving AI interventions. It emphasizes detailed descriptions of AI models, their integration into clinical workflows, and the management of input-output processes and error analyses. Incorporating such guidelines can help ensure transparency, reproducibility, and critical appraisal of AI-driven interventions in real-world settings [51]. Overall, adopting standardized reporting frameworks and ensuring access to key data sources will be essential to advancing the field of digital phenotyping for mental health.

### Limitations

This review was based on the features explicitly described in each study. In cases where a complete list of variables was not clearly reported, only those mentioned in the main text were considered included features. This approach may particularly affect raw sensor data, which are often processed into derived variables that are then highlighted as key features. For instance, light exposure may be used to infer sleep, but only the sleep feature may be mentioned in the results, while the contributing light exposure data may not be explicitly acknowledged. As a result, there is potential for reporting bias, particularly in studies that do not provide full transparency regarding feature extraction and selection. This limitation highlights the need for future studies to provide comprehensive documentation of both raw and derived variables, along with clear descriptions of their processing pipelines.

In addition, each study used different active mental health assessment tools, such as the Montgomery–Åsberg Depression Rating Scale, Patient Health Questionnaire-9, Hamilton Depression Rating Scale, and Beck Depression Inventory-II, either as predictive features or as outcome variables. However, due to the scope and limitations of this study, these active assessment tools were not included in our analysis. The use of different assessment tools may also introduce variability in the measurement of depression or anxiety severity, as each scale has unique scoring thresholds, symptom emphasis, and time frames. This heterogeneity could have influenced the strength or direction of associations between features and outcomes across studies.

In addition, the binary indicator (●) used to represent feature importance in this review was synthesized across heterogeneous analytical approaches, including machine learning, deep learning, and traditional statistical methods. While we applied consistent coding criteria to identify features emphasized by the authors or ranked highly in reported importance metrics, this harmonization inevitably simplifies the wide variation in feature selection and reporting strategies across studies. Future reviews may benefit from using more standardized quantitative strategies—such as vote-counting, feature ranking thresholds, or model-weighted scoring systems—to enhance comparability and reduce potential bias in cross-study interpretation of feature importance.

This study did not conduct a meta-analysis due to substantial heterogeneity in study designs, outcome measures, and feature selection methods, which limited the availability of comparable effect size metrics. Although we descriptively stratified studies by device type, target population, and analytic method, meta-regression analyses were not feasible for similar reasons. As digital phenotyping research evolves and reporting becomes more standardized, future reviews may be better positioned to conduct meta-analyses. Furthermore, incorporating gray literature and unpublished data may help reduce potential publication bias.

In addition, although key features such as accelerometer and HR were frequently identified, this review did not examine how these features were processed or integrated into predictive models. Many of the included studies addressed preprocessing techniques and modeling approaches in depth (eg, Price et al [26]). However, we did not explore these aspects in detail due to the considerable variation across studies. A thorough synthesis of such diverse methods would require an entirely separate review. Future studies should investigate modeling strategies—including feature engineering, data transformation, and temporal aggregation—to enhance both clinical applicability and technical understanding.

### Conclusions

This systematic review synthesized findings from 22 studies to identify the most relevant and frequently used features in digital phenotyping research involving combinations of smartphones with Actiwatch devices, smart bands, or smartwatches. The analysis revealed that accelerometer data and its derived features, such as step count and activity levels, were consistently used, especially in studies using Actiwatch and smart bands. HR-related features were also frequently used, whereas other physiological signals, such as SpO₂ and BVP, were rarely used, largely due to sensor limitations or restricted access. Sleep-related features demonstrated strong predictive potential but were notably absent in Actiwatch-based studies, despite the availability of supporting indicators such as light exposure and activity data. This represents a missed opportunity for more comprehensive usage of the feature. Smartphone-derived behavioral features (eg, call logs, screen time, and app usage) were underused in smartwatch-based studies, likely due to closed platforms and limited access to smartphone-linked data. These findings offer practical guidance for selecting features based on device type and highlight the need for improved data accessibility and reporting consistency. To promote standardization, we recommend the development of a reporting framework that explicitly documents both raw sensor inputs and derived features. Establishing a minimum feature set, such as accelerometer-based activity, HR, and sleep indicators, would enhance comparability across studies. In addition, prioritizing interoperable or open-access tools and transparently reporting preprocessing pipelines can facilitate reproducibility and enable future meta-analyses.

## Supplementary material

10.2196/77331 Multimedia Appendix 1 Quality assessment using the Mixed Methods Appraisal Tool.

10.2196/77331 Multimedia Appendix 2 Quality assessment using the Quality Criteria Checklist.

10.2196/77331 Multimedia Appendix 3 Stratified analyses.

10.2196/77331 Multimedia Appendix 4 Classification of data extraction methods and feature importance determination criteria.

10.2196/77331 Multimedia Appendix 5 Summary of studies and description of features.

10.2196/77331 Multimedia Appendix 6 Feature importance percentage.

10.2196/77331 Multimedia Appendix 7 Sensitivity analysis.

10.2196/77331 Checklist 1 PRISMA abstract checklist.

10.2196/77331 Checklist 2 PRISMA checklist.

## Abbreviations

AI: artificial intelligence
BVP: blood volume pulse
CONSORT-AI: Consolidated Standards of Reporting Trials–Artificial Intelligence
DAPPER: Digital Assessment and Performance Reporting
EDA: electrodermal activity
HR: heart rate
PICOS: population, intervention, comparison, outcomes, and study
PRISMA: Preferred Reporting Items for Systematic Reviews and Meta-Analyses
SFA: synthesized features of Actiwatch
SFB: synthesized features of smart band
SFW: synthesized features of smartwatch
SpO₂: peripheral capillary oxygen saturation
TSF: total synthesized feature
WHO: World Health Organization
