# Supplementary material for: Key Features of Digital Phenotyping for Monitoring Mental Disorders: Systematic Review
Source: J Med Internet Res. 2025 Nov 5;27:e77331. doi: 10.2196/77331 (PMC12588392; doi:10.2196/77331)
Supplement: Multimedia Appendix 1 [file jmir-v27-e77331-s001.docx]

**Part I: Mixed Methods Appraisal Tool (MMAT), version 2018**

1. **Exploring Digital Biomarkers of Illness Activity in Mood Episodes: Hypotheses Generating and Model Development Study**

| **Category of study designs** | **Methodological quality criteria** | **Responses** | | | |
| --- | --- | --- | --- | --- | --- |
|  |  | Yes | No | Can’t tell | Comments |
| Screening questions (for all types) | S1. Are there clear research questions? | O |  |  |  |
|  | S2. Do the collected data allow to address the research questions? | O |  |  |  |
|  | *Further appraisal may not be feasible or appropriate when the answer is ‘No’ or ‘Can’t tell’ to one or both screening questions.* | | | | |
| 1. Qualitative | 1.1. Is the qualitative approach appropriate to answer the research question? |  |  |  |  |
|  | 1.2. Are the qualitative data collection methods adequate to address the research question? |  |  |  |  |
|  | 1.3. Are the findings adequately derived from the data? |  |  |  |  |
|  | 1.4. Is the interpretation of results sufficiently substantiated by data? |  |  |  |  |
|  | 1.5. Is there coherence between qualitative data sources, collection, analysis and interpretation? |  |  |  |  |
| 2. Quantitative randomized controlled trials | 2.1. Is randomization appropriately performed? |  |  |  |  |
|  | 2.2. Are the groups comparable at baseline? |  |  |  |  |
|  | 2.3. Are there complete outcome data? |  |  |  |  |
|  | 2.4. Are outcome assessors blinded to the intervention provided? |  |  |  |  |
|  | 2.5 Did the participants adhere to the assigned intervention? |  |  |  |  |
| 3. Quantitative non- randomized | 3.1. Are the participants representative of the target population? |  | O |  |  |
|  | 3.2. Are measurements appropriate regarding both the outcome and intervention (or exposure)? | O |  |  |  |
|  | 3.3. Are there complete outcome data? | O |  |  |  |
|  | 3.4. Are the confounders accounted for in the design and analysis? |  | O |  |  |
|  | 3.5. During the study period, is the intervention administered (or exposure occurred) as intended? | O |  |  |  |
| 4. Quantitative descriptive | 4.1. Is the sampling strategy relevant to address the research question? |  |  |  |  |
|  | 4.2. Is the sample representative of the target population? |  |  |  |  |
|  | 4.3. Are the measurements appropriate? |  |  |  |  |
|  | 4.4. Is the risk of nonresponse bias low? |  |  |  |  |
|  | 4.5. Is the statistical analysis appropriate to answer the research question? |  |  |  |  |
| 5. Mixed methods | 5.1. Is there an adequate rationale for using a mixed methods design to address the research question? |  |  |  |  |
|  | 5.2. Are the different components of the study effectively integrated to answer the research question? |  |  |  |  |
|  | 5.3. Are the outputs of the integration of qualitative and quantitative components adequately interpreted? |  |  |  |  |
|  | 5.4. Are divergences and inconsistencies between quantitative and qualitative results adequately addressed? |  |  |  |  |
|  | 5.5. Do the different components of the study adhere to the quality criteria of each tradition of the methods involved? |  |  |  |  |

**Part II: Explanations**

| **3. Quantitative non-randomized studies** | **Methodological quality criteria** |
| --- | --- |
| Non-randomized studies are defined as any quantitative studies estimating the effectiveness of an intervention or studying other exposures that do not use randomization to allocate units to comparison groups (Higgins and Green, 2008).  Common designs include (this list is not exhaustive):  **Non-randomized controlled trials**  The intervention is assigned by researchers, but there is no randomization, e.g., a pseudo-randomization. A non- random method of allocation is not reliable in producing alone similar groups.  **Cohort study**  Subsets of a defined population are assessed as exposed, not exposed, or exposed at different degrees to factors of interest. Participants are followed over time to determine if an outcome occurs (prospective longitudinal).  **Case-control study**  Cases, e.g., patients, associated with a certain outcome are selected, alongside a corresponding group of controls.  Data is collected on whether cases and controls were exposed to the factor under study (retrospective).  **Cross-sectional analytic study**  At one particular time, the relationship between health- related characteristics (outcome) and other factors (intervention/exposure) is examined. E.g., the frequency of outcomes is compared in different population subgroups according to the presence/absence (or level) of the intervention/exposure. | 3.1. Are the participants representative of the target population?  - No  - The sample was small (n=19), clinician-selected, and not randomly drawn, which limits representativeness of the broader BD/MDD population. |
|  | 3.2. Are measurements appropriate regarding both the outcome and intervention (or exposure)?  - Yes  - Outcome measures (HDRS, YMRS) are validated clinical tools, and exposure (wearable data) was collected using a research-grade device (Empatica E4). |
|  | 3.3. Are there complete outcome data?  **-** Yes  - All participants were accounted for. Although ~11% of data were excluded post–quality control, the withdrawal rate was within acceptable range and transparently reported. |
|  | 3.4. Are the confounders accounted for in the design and analysis?  - No  - Confounders such as age, sex, medication use, and comorbidities were not controlled for in the study design or analysis. |
|  | 3.5 During the study period, is the intervention administered (or exposure occurred) as intended?  - Yes  - Exposure via wearable monitoring was applied consistently (~48h per session), and the data were collected as intended without evidence of contamination or deviation. |

**Part I: Mixed Methods Appraisal Tool (MMAT), version 2018**

1. **Sequence Modeling of Passive Sensing Data for Treatment Response Prediction in Major Depressive Disorder**

| **Category of study designs** | **Methodological quality criteria** | **Responses** | | | |
| --- | --- | --- | --- | --- | --- |
|  |  | Yes | No | Can’t tell | Comments |
| Screening questions (for all types) | S1. Are there clear research questions? | O |  |  |  |
|  | S2. Do the collected data allow to address the research questions? | O |  |  |  |
|  | *Further appraisal may not be feasible or appropriate when the answer is ‘No’ or ‘Can’t tell’ to one or both screening questions.* | | | | |
| 1. Qualitative | 1.1. Is the qualitative approach appropriate to answer the research question? |  |  |  |  |
|  | 1.2. Are the qualitative data collection methods adequate to address the research question? |  |  |  |  |
|  | 1.3. Are the findings adequately derived from the data? |  |  |  |  |
|  | 1.4. Is the interpretation of results sufficiently substantiated by data? |  |  |  |  |
|  | 1.5. Is there coherence between qualitative data sources, collection, analysis and interpretation? |  |  |  |  |
| 2. Quantitative randomized controlled trials | 2.1. Is randomization appropriately performed? |  |  |  |  |
|  | 2.2. Are the groups comparable at baseline? |  |  |  |  |
|  | 2.3. Are there complete outcome data? |  |  |  |  |
|  | 2.4. Are outcome assessors blinded to the intervention provided? |  |  |  |  |
|  | 2.5 Did the participants adhere to the assigned intervention? |  |  |  |  |
| 3. Quantitative non- randomized | 3.1. Are the participants representative of the target population? |  | O |  |  |
|  | 3.2. Are measurements appropriate regarding both the outcome and intervention (or exposure)? | O |  |  |  |
|  | 3.3. Are there complete outcome data? | O |  |  |  |
|  | 3.4. Are the confounders accounted for in the design and analysis? |  | O |  |  |
|  | 3.5. During the study period, is the intervention administered (or exposure occurred) as intended? | O |  |  |  |
| 4. Quantitative descriptive | 4.1. Is the sampling strategy relevant to address the research question? |  |  |  |  |
|  | 4.2. Is the sample representative of the target population? |  |  |  |  |
|  | 4.3. Are the measurements appropriate? |  |  |  |  |
|  | 4.4. Is the risk of nonresponse bias low? |  |  |  |  |
|  | 4.5. Is the statistical analysis appropriate to answer the research question? |  |  |  |  |
| 5. Mixed methods | 5.1. Is there an adequate rationale for using a mixed methods design to address the research question? |  |  |  |  |
|  | 5.2. Are the different components of the study effectively integrated to answer the research question? |  |  |  |  |
|  | 5.3. Are the outputs of the integration of qualitative and quantitative components adequately interpreted? |  |  |  |  |
|  | 5.4. Are divergences and inconsistencies between quantitative and qualitative results adequately addressed? |  |  |  |  |
|  | 5.5. Do the different components of the study adhere to the quality criteria of each tradition of the methods involved? |  |  |  |  |

**Part II: Explanations**

| **3. Quantitative non-randomized studies** | **Methodological quality criteria** |
| --- | --- |
| Non-randomized studies are defined as any quantitative studies estimating the effectiveness of an intervention or studying other exposures that do not use randomization to allocate units to comparison groups (Higgins and Green, 2008).  Common designs include (this list is not exhaustive):  **Non-randomized controlled trials**  The intervention is assigned by researchers, but there is no randomization, e.g., a pseudo-randomization. A non- random method of allocation is not reliable in producing alone similar groups.  **Cohort study**  Subsets of a defined population are assessed as exposed, not exposed, or exposed at different degrees to factors of interest. Participants are followed over time to determine if an outcome occurs (prospective longitudinal).  **Case-control study**  Cases, e.g., patients, associated with a certain outcome are selected, alongside a corresponding group of controls.  Data is collected on whether cases and controls were exposed to the factor under study (retrospective).  **Cross-sectional analytic study**  At one particular time, the relationship between health- related characteristics (outcome) and other factors (intervention/exposure) is examined. E.g., the frequency of outcomes is compared in different population subgroups according to the presence/absence (or level) of the intervention/exposure. | 3.1. Are the participants representative of the target population?  - No  - Small, clinician-selected sample limits generalizability. |
|  | 3.2. Are measurements appropriate regarding both the outcome and intervention (or exposure)?  - Yes  - Validated tools (PHQ-8, passive sensing) used appropriately. |
|  | 3.3. Are there complete outcome data?  - Yes  - Dropout and missing data reported; acceptable completeness. |
|  | 3.4. Are the confounders accounted for in the design and analysis?  - No  - No statistical adjustment for age, sex, or treatment. |
|  | 3.5 During the study period, is the intervention administered (or exposure occurred) as intended?  - Yes  - Passive data collection followed as planned (8-week period). |

**Part I: Mixed Methods Appraisal Tool (MMAT), version 2018**

1. **Using Digital Phenotyping to Accurately Detect Depression Severity**

| **Category of study designs** | **Methodological quality criteria** | **Responses** | | | |
| --- | --- | --- | --- | --- | --- |
|  |  | Yes | No | Can’t tell | Comments |
| Screening questions (for all types) | S1. Are there clear research questions? | O |  |  |  |
|  | S2. Do the collected data allow to address the research questions? | O |  |  |  |
|  | *Further appraisal may not be feasible or appropriate when the answer is ‘No’ or ‘Can’t tell’ to one or both screening questions.* | | | | |
| 1. Qualitative | 1.1. Is the qualitative approach appropriate to answer the research question? |  |  |  |  |
|  | 1.2. Are the qualitative data collection methods adequate to address the research question? |  |  |  |  |
|  | 1.3. Are the findings adequately derived from the data? |  |  |  |  |
|  | 1.4. Is the interpretation of results sufficiently substantiated by data? |  |  |  |  |
|  | 1.5. Is there coherence between qualitative data sources, collection, analysis and interpretation? |  |  |  |  |
| 2. Quantitative randomized controlled trials | 2.1. Is randomization appropriately performed? |  |  |  |  |
|  | 2.2. Are the groups comparable at baseline? |  |  |  |  |
|  | 2.3. Are there complete outcome data? |  |  |  |  |
|  | 2.4. Are outcome assessors blinded to the intervention provided? |  |  |  |  |
|  | 2.5 Did the participants adhere to the assigned intervention? |  |  |  |  |
| 3. Quantitative non- randomized | 3.1. Are the participants representative of the target population? |  | O |  | Small, female-skewed sample |
|  | 3.2. Are measurements appropriate regarding both the outcome and intervention (or exposure)? | O |  |  | Valid tools and objective digital data |
|  | 3.3. Are there complete outcome data? |  | O |  | Data from 5 participants were excluded with no explanation. |
|  | 3.4. Are the confounders accounted for in the design and analysis? | O |  |  | Age and sex were statistically controlled. |
|  | 3.5. During the study period, is the intervention administered (or exposure occurred) as intended? |  |  | O | Adherence to wearable use was not reported. |
| 4. Quantitative descriptive | 4.1. Is the sampling strategy relevant to address the research question? |  |  |  |  |
|  | 4.2. Is the sample representative of the target population? |  |  |  |  |
|  | 4.3. Are the measurements appropriate? |  |  |  |  |
|  | 4.4. Is the risk of nonresponse bias low? |  |  |  |  |
|  | 4.5. Is the statistical analysis appropriate to answer the research question? |  |  |  |  |
| 5. Mixed methods | 5.1. Is there an adequate rationale for using a mixed methods design to address the research question? |  |  |  |  |
|  | 5.2. Are the different components of the study effectively integrated to answer the research question? |  |  |  |  |
|  | 5.3. Are the outputs of the integration of qualitative and quantitative components adequately interpreted? |  |  |  |  |
|  | 5.4. Are divergences and inconsistencies between quantitative and qualitative results adequately addressed? |  |  |  |  |
|  | 5.5. Do the different components of the study adhere to the quality criteria of each tradition of the methods involved? |  |  |  |  |

**Part II: Explanations**

| **3. Quantitative non-randomized studies** | **Methodological quality criteria** |
| --- | --- |
| Non-randomized studies are defined as any quantitative studies estimating the effectiveness of an intervention or studying other exposures that do not use randomization to allocate units to comparison groups (Higgins and Green, 2008). Common designs include (this list is not exhaustive):  **Non-randomized controlled trials**  The intervention is assigned by researchers, but there is no randomization, e.g., a pseudo-randomization. A non- random method of allocation is not reliable in producing alone similar groups.  **Cohort study**  Subsets of a defined population are assessed as exposed, not exposed, or exposed at different degrees to factors of interest. Participants are followed over time to determine if an outcome occurs (prospective longitudinal).  **Case-control study**  Cases, e.g., patients, associated with a certain outcome are selected, alongside a corresponding group of controls.  Data is collected on whether cases and controls were exposed to the factor under study (retrospective).  **Cross-sectional analytic study**  At one particular time, the relationship between health- related characteristics (outcome) and other factors (intervention/exposure) is examined. E.g., the frequency of outcomes is compared in different population subgroups according to the presence/absence (or level) of the intervention/exposure | 3.1. Are the participants representative of the target population?  - No  - Small, mostly female sample limits generalizability. |
|  | 3.2. Are measurements appropriate regarding both the outcome and intervention (or exposure)?  - Yes  - Valid clinical scales and objective sensor data were used. |
|  | 3.3. Are there complete outcome data?  - No  - 5 participants excluded without explanation (original dataset = 20). |
|  | 3.4. Are the confounders accounted for in the design and analysis?  - Yes  - Age and sex were controlled statistically. |
|  | 3.5 During the study period, is the intervention administered (or exposure occurred) as intended?  - Can’t tell  - Adherence to wearable use was not reported. |

**Part I: Mixed Methods Appraisal Tool (MMAT), version 2018**

1. **An unsupervised machine learning approach using passive movement data to understand depression and schizophrenia**

| **Category of study designs** | **Methodological quality criteria** | **Responses** | | | |
| --- | --- | --- | --- | --- | --- |
|  |  | Yes | No | Can’t tell | Comments |
| Screening questions (for all types) | S1. Are there clear research questions? | O |  |  |  |
|  | S2. Do the collected data allow to address the research questions? | O |  |  |  |
|  | *Further appraisal may not be feasible or appropriate when the answer is ‘No’ or ‘Can’t tell’ to one or both screening questions.* | | | | |
| 1. Qualitative | 1.1. Is the qualitative approach appropriate to answer the research question? |  |  |  |  |
|  | 1.2. Are the qualitative data collection methods adequate to address the research question? |  |  |  |  |
|  | 1.3. Are the findings adequately derived from the data? |  |  |  |  |
|  | 1.4. Is the interpretation of results sufficiently substantiated by data? |  |  |  |  |
|  | 1.5. Is there coherence between qualitative data sources, collection, analysis and interpretation? |  |  |  |  |
| 2. Quantitative randomized controlled trials | 2.1. Is randomization appropriately performed? |  |  |  |  |
|  | 2.2. Are the groups comparable at baseline? |  |  |  |  |
|  | 2.3. Are there complete outcome data? |  |  |  |  |
|  | 2.4. Are outcome assessors blinded to the intervention provided? |  |  |  |  |
|  | 2.5 Did the participants adhere to the assigned intervention? |  |  |  |  |
| 3. Quantitative non- randomized | 3.1. Are the participants representative of the target population? |  | O |  | Small sample, specific clinical setting |
|  | 3.2. Are measurements appropriate regarding both the outcome and intervention (or exposure)? | O |  |  |  |
|  | 3.3. Are there complete outcome data? | O |  |  | No drop out |
|  | 3.4. Are the confounders accounted for in the design and analysis? |  | O |  | Does not needed. |
|  | 3.5. During the study period, is the intervention administered (or exposure occurred) as intended? | O |  |  |  |
| 4. Quantitative descriptive | 4.1. Is the sampling strategy relevant to address the research question? |  |  |  |  |
|  | 4.2. Is the sample representative of the target population? |  |  |  |  |
|  | 4.3. Are the measurements appropriate? |  |  |  |  |
|  | 4.4. Is the risk of nonresponse bias low? |  |  |  |  |
|  | 4.5. Is the statistical analysis appropriate to answer the research question? |  |  |  |  |
| 5. Mixed methods | 5.1. Is there an adequate rationale for using a mixed methods design to address the research question? |  |  |  |  |
|  | 5.2. Are the different components of the study effectively integrated to answer the research question? |  |  |  |  |
|  | 5.3. Are the outputs of the integration of qualitative and quantitative components adequately interpreted? |  |  |  |  |
|  | 5.4. Are divergences and inconsistencies between quantitative and qualitative results adequately addressed? |  |  |  |  |
|  | 5.5. Do the different components of the study adhere to the quality criteria of each tradition of the methods involved? |  |  |  |  |

**Part II: Explanations**

| **3. Quantitative non-randomized studies** | **Methodological quality criteria** |
| --- | --- |
| Non-randomized studies are defined as any quantitative studies estimating the effectiveness of an intervention or studying other exposures that do not use randomization to allocate units to comparison groups (Higgins and Green, 2008).  Common designs include (this list is not exhaustive):  **Non-randomized controlled trials**  The intervention is assigned by researchers, but there is no randomization, e.g., a pseudo-randomization. A non- random method of allocation is not reliable in producing alone similar groups.  **Cohort study**  Subsets of a defined population are assessed as exposed, not exposed, or exposed at different degrees to factors of interest. Participants are followed over time to determine if an outcome occurs (prospective longitudinal).  **Case-control study**  Cases, e.g., patients, associated with a certain outcome are selected, alongside a corresponding group of controls.  Data is collected on whether cases and controls were exposed to the factor under study (retrospective).  **Cross-sectional analytic study**  At one particular time, the relationship between health- related characteristics (outcome) and other factors (intervention/exposure) is examined. E.g., the frequency of outcomes is compared in different population subgroups according to the presence/absence (or level) of the intervention/exposure. | 3.1. Are the participants representative of the target population?  - No  - The sample was relatively small and recruited from a clinical setting, which may limit generalizability. |
|  | 3.2. Are measurements appropriate regarding both the outcome and intervention (or exposure)?  - Yes  - Objective and validated tools (e.g., actigraphy) were used to measure exposure; methods were appropriate for the research question. |
|  | 3.3. Are there complete outcome data?  - Yes  - No missing data were reported; all enrolled participants were included in the final analysis. |
|  | 3.4. Are the confounders accounted for in the design and analysis?  - No  - No adjustments were made for potential confounding variables such as age, sex, or medication use.   \|  \| \| --- \| |
|  | 3.5 During the study period, is the intervention administered (or exposure occurred) as intended?  - Yes  - Passive movement data were collected as planned over a one-week period with full compliance. |

**Part I: Mixed Methods Appraisal Tool (MMAT), version 2018**

1. **An Observational Pilot Study using a Digital Phenotyping Approach in Patients with Major Depressive Disorder Treated with Trazodone**

| **Category of study designs** | **Methodological quality criteria** | **Responses** | | | |
| --- | --- | --- | --- | --- | --- |
|  |  | Yes | No | Can’t tell | Comments |
| Screening questions (for all types) | S1. Are there clear research questions? | O |  |  |  |
|  | S2. Do the collected data allow to address the research questions? | O |  |  |  |
|  | *Further appraisal may not be feasible or appropriate when the answer is ‘No’ or ‘Can’t tell’ to one or both screening questions.* | | | | |
| 1. Qualitative | 1.1. Is the qualitative approach appropriate to answer the research question? |  |  |  |  |
|  | 1.2. Are the qualitative data collection methods adequate to address the research question? |  |  |  |  |
|  | 1.3. Are the findings adequately derived from the data? |  |  |  |  |
|  | 1.4. Is the interpretation of results sufficiently substantiated by data? |  |  |  |  |
|  | 1.5. Is there coherence between qualitative data sources, collection, analysis and interpretation? |  |  |  |  |
| 2. Quantitative randomized controlled trials | 2.1. Is randomization appropriately performed? |  |  |  |  |
|  | 2.2. Are the groups comparable at baseline? |  |  |  |  |
|  | 2.3. Are there complete outcome data? |  |  |  |  |
|  | 2.4. Are outcome assessors blinded to the intervention provided? |  |  |  |  |
|  | 2.5 Did the participants adhere to the assigned intervention? |  |  |  |  |
| 3. Quantitative non- randomized | 3.1. Are the participants representative of the target population? |  |  |  |  |
|  | 3.2. Are measurements appropriate regarding both the outcome and intervention (or exposure)? |  |  |  |  |
|  | 3.3. Are there complete outcome data? |  |  |  |  |
|  | 3.4. Are the confounders accounted for in the design and analysis? |  |  |  |  |
|  | 3.5. During the study period, is the intervention administered (or exposure occurred) as intended? |  |  |  |  |
| 4. Quantitative descriptive | 4.1. Is the sampling strategy relevant to address the research question? | O |  |  |  |
|  | 4.2. Is the sample representative of the target population? |  | O |  | Small sample size and selected from a clinical setting |
|  | 4.3. Are the measurements appropriate? | O |  |  |  |
|  | 4.4. Is the risk of nonresponse bias low? | O |  |  |  |
|  | 4.5. Is the statistical analysis appropriate to answer the research question? | O |  |  |  |
| 5. Mixed methods | 5.1. Is there an adequate rationale for using a mixed methods design to address the research question? |  |  |  |  |
|  | 5.2. Are the different components of the study effectively integrated to answer the research question? |  |  |  |  |
|  | 5.3. Are the outputs of the integration of qualitative and quantitative components adequately interpreted? |  |  |  |  |
|  | 5.4. Are divergences and inconsistencies between quantitative and qualitative results adequately addressed? |  |  |  |  |
|  | 5.5. Do the different components of the study adhere to the quality criteria of each tradition of the methods involved? |  |  |  |  |

**Part II: Explanations**

| **4. Quantitative descriptive studies** | **Methodological quality criteria** |
| --- | --- |
| Quantitative descriptive studies are “concerned with and designed only to describe the existing distribution of variables without much regard to causal relationships or other hypotheses” (Porta et al., 2014, p. 72). They are used to monitoring the population, planning, and generating hypothesis (Grimes and Schulz, 2002).  Common designs include the following single-group studies (this list is not exhaustive):  **Incidence or prevalence study without comparison group**  In a defined population at one particular time, what is happening in a population, e.g., frequencies of factors (importance of problems), is described (portrayed).  **Survey**  “Research method by which information is gathered by asking people questions on a specific topic and the data collection procedure is standardized and well defined.” (Bennett et al., 2011, p. 3).  **Case series**  A collection of individuals with similar characteristics are used to describe an outcome.  **Case report**  An individual or a group with a unique/unusual outcome is described in detail.  Key references: Critical Appraisal Skills Programme (2017); Draugalis et al. (2008) | 4.1. Is the sampling strategy relevant to address the research question?  - Yes  - The sample consisted of patients with MDD receiving trazodone, which is relevant to the research aim. Although convenience sampling was used, the sample frame was appropriate for a pilot study. |
|  | 4.2. Is the sample representative of the target population?  - No  - The sample was small (n = 10) and drawn from a single clinical setting, limiting generalizability to the broader MDD population. |
|  | 4.3. Are the measurements appropriate?  - Yes  - Validated tools such as MADRS, PHQ-9, and actigraphy-based digital phenotyping were used, making the measurements appropriate and reliable. |
|  | 4.4. Is the risk of nonresponse bias low?  - Yes  - All enrolled participants contributed data to the final analysis; no dropouts or missing data were reported. |
|  | 4.5. Is the statistical analysis appropriate to answer the research question?  - Yes  - Descriptive statistics and Pearson correlation were appropriate for the exploratory and observational nature of the study. |

**Part I: Mixed Methods Appraisal Tool (MMAT), version 2018**

1. **Large-scale digital phenotyping: Identifying depression and anxiety indicators in a general UK population with over 10,000 participants**

| **Category of study designs** | **Methodological quality criteria** | **Responses** | | | |
| --- | --- | --- | --- | --- | --- |
|  |  | Yes | No | Can’t tell | Comments |
| Screening questions (for all types) | S1. Are there clear research questions? | O |  |  |  |
|  | S2. Do the collected data allow to address the research questions? | O |  |  |  |
|  | *Further appraisal may not be feasible or appropriate when the answer is ‘No’ or ‘Can’t tell’ to one or both screening questions.* | | | | |
| 1. Qualitative | 1.1. Is the qualitative approach appropriate to answer the research question? |  |  |  |  |
|  | 1.2. Are the qualitative data collection methods adequate to address the research question? |  |  |  |  |
|  | 1.3. Are the findings adequately derived from the data? |  |  |  |  |
|  | 1.4. Is the interpretation of results sufficiently substantiated by data? |  |  |  |  |
|  | 1.5. Is there coherence between qualitative data sources, collection, analysis and interpretation? |  |  |  |  |
| 2. Quantitative randomized controlled trials | 2.1. Is randomization appropriately performed? |  |  |  |  |
|  | 2.2. Are the groups comparable at baseline? |  |  |  |  |
|  | 2.3. Are there complete outcome data? |  |  |  |  |
|  | 2.4. Are outcome assessors blinded to the intervention provided? |  |  |  |  |
|  | 2.5 Did the participants adhere to the assigned intervention? |  |  |  |  |
| 3. Quantitative non- randomized | 3.1. Are the participants representative of the target population? |  | O |  |  |
|  | 3.2. Are measurements appropriate regarding both the outcome and intervention (or exposure)? | O |  |  |  |
|  | 3.3. Are there complete outcome data? | O |  |  |  |
|  | 3.4. Are the confounders accounted for in the design and analysis? |  |  | O | Does not needed |
|  | 3.5. During the study period, is the intervention administered (or exposure occurred) as intended? | O |  |  |  |
| 4. Quantitative descriptive | 4.1. Is the sampling strategy relevant to address the research question? |  |  |  |  |
|  | 4.2. Is the sample representative of the target population? |  |  |  |  |
|  | 4.3. Are the measurements appropriate? |  |  |  |  |
|  | 4.4. Is the risk of nonresponse bias low? |  |  |  |  |
|  | 4.5. Is the statistical analysis appropriate to answer the research question? |  |  |  |  |
| 5. Mixed methods | 5.1. Is there an adequate rationale for using a mixed methods design to address the research question? |  |  |  |  |
|  | 5.2. Are the different components of the study effectively integrated to answer the research question? |  |  |  |  |
|  | 5.3. Are the outputs of the integration of qualitative and quantitative components adequately interpreted? |  |  |  |  |
|  | 5.4. Are divergences and inconsistencies between quantitative and qualitative results adequately addressed? |  |  |  |  |
|  | 5.5. Do the different components of the study adhere to the quality criteria of each tradition of the methods involved? |  |  |  |  |

**Part II: Explanations**

| **3. Quantitative non-randomized studies** | **Methodological quality criteria** |
| --- | --- |
| Non-randomized studies are defined as any quantitative studies estimating the effectiveness of an intervention or studying other exposures that do not use randomization to allocate units to comparison groups (Higgins and Green, 2008).  **Non-randomized controlled trials**  The intervention is assigned by researchers, but there is no randomization, e.g., a pseudo-randomization. A non- random method of allocation is not reliable in producing alone similar groups.  **Cohort study**  Subsets of a defined population are assessed as exposed, not exposed, or exposed at different degrees to factors of interest. Participants are followed over time to determine if an outcome occurs (prospective longitudinal).  **Case-control study**  Cases, e.g., patients, associated with a certain outcome are selected, alongside a corresponding group of controls.  Data is collected on whether cases and controls were exposed to the factor under study (retrospective).  **Cross-sectional analytic study**  At one particular time, the relationship between health- related characteristics (outcome) and other factors (intervention/exposure) is examined. E.g., the frequency of outcomes is compared in different population subgroups according to the presence/absence (or level) of the intervention/exposure. | 3.1. Are the participants representative of the target population?  - No  - Although the sample is large, participants with complete wearable data may not fully represent the general UK population. |
|  | 3.2. Are measurements appropriate regarding both the outcome and intervention (or exposure)?  - Yes  - Validated tools (PHQ-8, GAD-7) and objectively collected wearable data were used appropriately. |
|  | 3.3. Are there complete outcome data?  - Yes  - Only participants with sufficient data quality and complete outcome measures were included in the analysis |
|  | 3.4. Are the confounders accounted for in the design and analysis?  - N/A  - The aim was prediction using machine learning, not causal inference. Confounder control was not required. |
|  | 3.5 During the study period, is the intervention administered (or exposure occurred) as intended?  - Yes  - Passive data collection occurred as planned using consistent protocols and devices. |

**Part I: Mixed Methods Appraisal Tool (MMAT), version 2018**

1. **Digital Phenotyping of Geriatric Depression Using a Community-Based Digital Mental Health Monitoring Platform for Socially Vulnerable Older Adults and Their Community Caregivers: 6-Week Living Lab Single-Arm Pilot Study**

| **Category of study designs** | **Methodological quality criteria** | **Responses** | | | |
| --- | --- | --- | --- | --- | --- |
|  |  | Yes | No | Can’t tell | Comments |
| Screening questions (for all types) | S1. Are there clear research questions? | O |  |  |  |
|  | S2. Do the collected data allow to address the research questions? | O |  |  |  |
|  | *Further appraisal may not be feasible or appropriate when the answer is ‘No’ or ‘Can’t tell’ to one or both screening questions.* | | | | |
| 1. Qualitative | 1.1. Is the qualitative approach appropriate to answer the research question? |  |  |  |  |
|  | 1.2. Are the qualitative data collection methods adequate to address the research question? |  |  |  |  |
|  | 1.3. Are the findings adequately derived from the data? |  |  |  |  |
|  | 1.4. Is the interpretation of results sufficiently substantiated by data? |  |  |  |  |
|  | 1.5. Is there coherence between qualitative data sources, collection, analysis and interpretation? |  |  |  |  |
| 2. Quantitative randomized controlled trials | 2.1. Is randomization appropriately performed? |  |  |  |  |
|  | 2.2. Are the groups comparable at baseline? |  |  |  |  |
|  | 2.3. Are there complete outcome data? |  |  |  |  |
|  | 2.4. Are outcome assessors blinded to the intervention provided? |  |  |  |  |
|  | 2.5 Did the participants adhere to the assigned intervention? |  |  |  |  |
| 3. Quantitative non- randomized | 3.1. Are the participants representative of the target population? | O |  |  |  |
|  | 3.2. Are measurements appropriate regarding both the outcome and intervention (or exposure)? | O |  |  |  |
|  | 3.3. Are there complete outcome data? | O |  |  |  |
|  | 3.4. Are the confounders accounted for in the design and analysis? | O |  |  |  |
|  | 3.5. During the study period, is the intervention administered (or exposure occurred) as intended? | O |  |  |  |
| 4. Quantitative descriptive | 4.1. Is the sampling strategy relevant to address the research question? |  |  |  |  |
|  | 4.2. Is the sample representative of the target population? |  |  |  |  |
|  | 4.3. Are the measurements appropriate? |  |  |  |  |
|  | 4.4. Is the risk of nonresponse bias low? |  |  |  |  |
|  | 4.5. Is the statistical analysis appropriate to answer the research question? |  |  |  |  |
| 5. Mixed methods | 5.1. Is there an adequate rationale for using a mixed methods design to address the research question? |  |  |  |  |
|  | 5.2. Are the different components of the study effectively integrated to answer the research question? |  |  |  |  |
|  | 5.3. Are the outputs of the integration of qualitative and quantitative components adequately interpreted? |  |  |  |  |
|  | 5.4. Are divergences and inconsistencies between quantitative and qualitative results adequately addressed? |  |  |  |  |
|  | 5.5. Do the different components of the study adhere to the quality criteria of each tradition of the methods involved? |  |  |  |  |

**Part II: Explanations**

| **3. Quantitative non-randomized studies** | **Methodological quality criteria** |
| --- | --- |
| Non-randomized studies are defined as any quantitative studies estimating the effectiveness of an intervention or studying other exposures that do not use randomization to allocate units to comparison groups (Higgins and Green, 2008).  **Non-randomized controlled trials**  The intervention is assigned by researchers, but there is no randomization, e.g., a pseudo-randomization. A non- random method of allocation is not reliable in producing alone similar groups.  **Cohort study**  Subsets of a defined population are assessed as exposed, not exposed, or exposed at different degrees to factors of interest. Participants are followed over time to determine if an outcome occurs (prospective longitudinal).  **Case-control study**  Cases, e.g., patients, associated with a certain outcome are selected, alongside a corresponding group of controls.  Data is collected on whether cases and controls were exposed to the factor under study (retrospective).  **Cross-sectional analytic study**  At one particular time, the relationship between health- related characteristics (outcome) and other factors (intervention/exposure) is examined. E.g., the frequency of outcomes is compared in different population subgroups according to the presence/absence (or level) of the intervention/exposure. | 3.1. Are the participants representative of the target population?  - Yes  - The study targeted socially vulnerable older adults, and the sample adequately reflects that population group. |
|  | 3.2. Are measurements appropriate regarding both the outcome and intervention (or exposure)?  - Yes  - Validated tools (e.g., PHQ-9, wearable sensor data) were used to ensure reliable and meaningful measurement. |
|  | 3.3. Are there complete outcome data?  - Yes  - The majority of participants completed the follow-up assessments, with missing data handled transparently. |
|  | 3.4. Are the confounders accounted for in the design and analysis?  - Yes  - Multivariable logistic regression was used to adjust for potential confounders such as age, gender, and living situation. |
|  | 3.5 During the study period, is the intervention administered (or exposure occurred) as intended?  - Yes  - The community-based digital monitoring platform was implemented consistently over the study period. |

**Part I: Mixed Methods Appraisal Tool (MMAT), version 2018**

1. **Monitoring Changes in Depression Severity Using Wearable and Mobile Sensors**

| **Category of study designs** | **Methodological quality criteria** | **Responses** | | | |
| --- | --- | --- | --- | --- | --- |
|  |  | Yes | No | Can’t tell | Comments |
| Screening questions (for all types) | S1. Are there clear research questions? | O |  |  |  |
|  | S2. Do the collected data allow to address the research questions? | O |  |  |  |
|  | *Further appraisal may not be feasible or appropriate when the answer is ‘No’ or ‘Can’t tell’ to one or both screening questions.* | | | | |
| 1. Qualitative | 1.1. Is the qualitative approach appropriate to answer the research question? |  |  |  |  |
|  | 1.2. Are the qualitative data collection methods adequate to address the research question? |  |  |  |  |
|  | 1.3. Are the findings adequately derived from the data? |  |  |  |  |
|  | 1.4. Is the interpretation of results sufficiently substantiated by data? |  |  |  |  |
|  | 1.5. Is there coherence between qualitative data sources, collection, analysis and interpretation? |  |  |  |  |
| 2. Quantitative randomized controlled trials | 2.1. Is randomization appropriately performed? |  |  |  |  |
|  | 2.2. Are the groups comparable at baseline? |  |  |  |  |
|  | 2.3. Are there complete outcome data? |  |  |  |  |
|  | 2.4. Are outcome assessors blinded to the intervention provided? |  |  |  |  |
|  | 2.5 Did the participants adhere to the assigned intervention? |  |  |  |  |
| 3. Quantitative non- randomized | 3.1. Are the participants representative of the target population? | O |  |  |  |
|  | 3.2. Are measurements appropriate regarding both the outcome and intervention (or exposure)? | O |  |  |  |
|  | 3.3. Are there complete outcome data? | O |  |  |  |
|  | 3.4. Are the confounders accounted for in the design and analysis? |  |  | O | Does not needed |
|  | 3.5. During the study period, is the intervention administered (or exposure occurred) as intended? | O |  |  |  |
| 4. Quantitative descriptive | 4.1. Is the sampling strategy relevant to address the research question? |  |  |  |  |
|  | 4.2. Is the sample representative of the target population? |  |  |  |  |
|  | 4.3. Are the measurements appropriate? |  |  |  |  |
|  | 4.4. Is the risk of nonresponse bias low? |  |  |  |  |
|  | 4.5. Is the statistical analysis appropriate to answer the research question? |  |  |  |  |
| 5. Mixed methods | 5.1. Is there an adequate rationale for using a mixed methods design to address the research question? |  |  |  |  |
|  | 5.2. Are the different components of the study effectively integrated to answer the research question? |  |  |  |  |
|  | 5.3. Are the outputs of the integration of qualitative and quantitative components adequately interpreted? |  |  |  |  |
|  | 5.4. Are divergences and inconsistencies between quantitative and qualitative results adequately addressed? |  |  |  |  |
|  | 5.5. Do the different components of the study adhere to the quality criteria of each tradition of the methods involved? |  |  |  |  |

**Part II: Explanations**

| **3. Quantitative non-randomized studies** | **Methodological quality criteria** |
| --- | --- |
| Non-randomized studies are defined as any quantitative studies estimating the effectiveness of an intervention or studying other exposures that do not use randomization to allocate units to comparison groups (Higgins and Green, 2008).  **Non-randomized controlled trials**  The intervention is assigned by researchers, but there is no randomization, e.g., a pseudo-randomization. A non- random method of allocation is not reliable in producing alone similar groups.  **Cohort study**  Subsets of a defined population are assessed as exposed, not exposed, or exposed at different degrees to factors of interest. Participants are followed over time to determine if an outcome occurs (prospective longitudinal).  **Case-control study**  Cases, e.g., patients, associated with a certain outcome are selected, alongside a corresponding group of controls.  Data is collected on whether cases and controls were exposed to the factor under study (retrospective).  **Cross-sectional analytic study**  At one particular time, the relationship between health- related characteristics (outcome) and other factors (intervention/exposure) is examined. E.g., the frequency of outcomes is compared in different population subgroups according to the presence/absence (or level) of the intervention/exposure. | 3.1. Are the participants representative of the target population?  - Yes  - Participants were recruited from public advertisements and outpatient clinics, reflecting typical individuals with major depressive disorder. |
|  | 3.2. Are measurements appropriate regarding both the outcome and intervention (or exposure)?  - Yes  - The outcome (PHQ-9) is clinically validated, and exposures (sensor-derived features) are widely accepted in digital mental health research. |
|  | 3.3. Are there complete outcome data?  - Yes  - 66 out of 89 participants completed both baseline and follow-up; missing data are addressed, and response rates are transparently reported. |
|  | 3.4. Are the confounders accounted for in the design and analysis?  - Yes  - No regression analysis or statistical adjustment for confounding variables was included. Only predictive modeling was used. |
|  | 3.5 During the study period, is the intervention administered (or exposure occurred) as intended?  - Yes  - Sensor data were collected as planned with good compliance (>90%), and passive sensing occurred during the study period as expected. |

**Part I: Mixed Methods Appraisal Tool (MMAT), version 2018**

1. **Tracking Depression Dynamics in College Students Using Mobile Phone and Wearable Sensing**

| **Category of study designs** | **Methodological quality criteria** | **Responses** | | | |
| --- | --- | --- | --- | --- | --- |
|  |  | Yes | No | Can’t tell | Comments |
| Screening questions (for all types) | S1. Are there clear research questions? | O |  |  |  |
|  | S2. Do the collected data allow to address the research questions? | O |  |  |  |
|  | *Further appraisal may not be feasible or appropriate when the answer is ‘No’ or ‘Can’t tell’ to one or both screening questions.* | | | | |
| 1. Qualitative | 1.1. Is the qualitative approach appropriate to answer the research question? |  |  |  |  |
|  | 1.2. Are the qualitative data collection methods adequate to address the research question? |  |  |  |  |
|  | 1.3. Are the findings adequately derived from the data? |  |  |  |  |
|  | 1.4. Is the interpretation of results sufficiently substantiated by data? |  |  |  |  |
|  | 1.5. Is there coherence between qualitative data sources, collection, analysis and interpretation? |  |  |  |  |
| 2. Quantitative randomized controlled trials | 2.1. Is randomization appropriately performed? |  |  |  |  |
|  | 2.2. Are the groups comparable at baseline? |  |  |  |  |
|  | 2.3. Are there complete outcome data? |  |  |  |  |
|  | 2.4. Are outcome assessors blinded to the intervention provided? |  |  |  |  |
|  | 2.5 Did the participants adhere to the assigned intervention? |  |  |  |  |
| 3. Quantitative non- randomized | 3.1. Are the participants representative of the target population? | O |  |  |  |
|  | 3.2. Are measurements appropriate regarding both the outcome and intervention (or exposure)? | O |  |  |  |
|  | 3.3. Are there complete outcome data? | O |  |  |  |
|  | 3.4. Are the confounders accounted for in the design and analysis? | O |  |  |  |
|  | 3.5. During the study period, is the intervention administered (or exposure occurred) as intended? | O |  |  |  |
| 4. Quantitative descriptive | 4.1. Is the sampling strategy relevant to address the research question? |  |  |  |  |
|  | 4.2. Is the sample representative of the target population? |  |  |  |  |
|  | 4.3. Are the measurements appropriate? |  |  |  |  |
|  | 4.4. Is the risk of nonresponse bias low? |  |  |  |  |
|  | 4.5. Is the statistical analysis appropriate to answer the research question? |  |  |  |  |
| 5. Mixed methods | 5.1. Is there an adequate rationale for using a mixed methods design to address the research question? |  |  |  |  |
|  | 5.2. Are the different components of the study effectively integrated to answer the research question? |  |  |  |  |
|  | 5.3. Are the outputs of the integration of qualitative and quantitative components adequately interpreted? |  |  |  |  |
|  | 5.4. Are divergences and inconsistencies between quantitative and qualitative results adequately addressed? |  |  |  |  |
|  | 5.5. Do the different components of the study adhere to the quality criteria of each tradition of the methods involved? |  |  |  |  |

**Part II: Explanations**

| **3. Quantitative non-randomized studies** | **Methodological quality criteria** |
| --- | --- |
| Non-randomized studies are defined as any quantitative studies estimating the effectiveness of an intervention or studying other exposures that do not use randomization to allocate units to comparison groups (Higgins and Green, 2008).  **Non-randomized controlled trials**  The intervention is assigned by researchers, but there is no randomization, e.g., a pseudo-randomization. A non- random method of allocation is not reliable in producing alone similar groups.  **Cohort study**  Subsets of a defined population are assessed as exposed, not exposed, or exposed at different degrees to factors of interest. Participants are followed over time to determine if an outcome occurs (prospective longitudinal).  **Case-control study**  Cases, e.g., patients, associated with a certain outcome are selected, alongside a corresponding group of controls.  Data is collected on whether cases and controls were exposed to the factor under study (retrospective).  **Cross-sectional analytic study**  At one particular time, the relationship between health- related characteristics (outcome) and other factors (intervention/exposure) is examined. E.g., the frequency of outcomes is compared in different population subgroups according to the presence/absence (or level) of the intervention/exposure. | 3.1. Are the participants representative of the target population?  - Yes  - The participants were college students, and the sample was described with adequate demographic details to suggest representativeness. |
|  | 3.2. Are measurements appropriate regarding both the outcome and intervention (or exposure)?  - Yes  - The outcome (depressive symptoms using PHQ-9) and exposures (passive sensing data) were measured using validated tools and procedures. |
|  | 3.3. Are there complete outcome data?  - Yes  - While some data were missing, the dataset used for analysis was sufficiently complete and missing data were discussed. |
|  | 3.4. Are the confounders accounted for in the design and analysis?  - Yes  - Potential confounders such as sex and sleep patterns were adjusted for using multivariate analysis in the machine learning models. |
|  | 3.5 During the study period, is the intervention administered (or exposure occurred) as intended?  - Yes  - Passive sensing was implemented as planned, and there were no deviations from the designed data collection protocol. |

**Part I: Mixed Methods Appraisal Tool (MMAT), version 2018**

1. **STDD: Short-Term Depression Detection with Passive Sensing**

| **Category of study designs** | **Methodological quality criteria** | **Responses** | | | |
| --- | --- | --- | --- | --- | --- |
|  |  | Yes | No | Can’t tell | Comments |
| Screening questions (for all types) | S1. Are there clear research questions? |  |  |  |  |
|  | S2. Do the collected data allow to address the research questions? |  |  |  |  |
|  | *Further appraisal may not be feasible or appropriate when the answer is ‘No’ or ‘Can’t tell’ to one or both screening questions.* | | | | |
| 1. Qualitative | 1.1. Is the qualitative approach appropriate to answer the research question? |  |  |  |  |
|  | 1.2. Are the qualitative data collection methods adequate to address the research question? |  |  |  |  |
|  | 1.3. Are the findings adequately derived from the data? |  |  |  |  |
|  | 1.4. Is the interpretation of results sufficiently substantiated by data? |  |  |  |  |
|  | 1.5. Is there coherence between qualitative data sources, collection, analysis and interpretation? |  |  |  |  |
| 2. Quantitative randomized controlled trials | 2.1. Is randomization appropriately performed? |  |  |  |  |
|  | 2.2. Are the groups comparable at baseline? |  |  |  |  |
|  | 2.3. Are there complete outcome data? |  |  |  |  |
|  | 2.4. Are outcome assessors blinded to the intervention provided? |  |  |  |  |
|  | 2.5 Did the participants adhere to the assigned intervention? |  |  |  |  |
| 3. Quantitative non- randomized | 3.1. Are the participants representative of the target population? |  |  | \|  \| \| --- \|   O | \| Generalizability to wider population not clearly addressed. \| \| --- \| |
|  | 3.2. Are measurements appropriate regarding both the outcome and intervention (or exposure)? | O |  |  |  |
|  | 3.3. Are there complete outcome data? |  |  | \|  \| \| --- \|   O | \| Data loss acknowledged, completeness unclear. \| \| --- \| |
|  | 3.4. Are the confounders accounted for in the design and analysis? |  | O | \|  \| \| --- \| | \| No statistical control for confounders. \| \| --- \| |
|  | 3.5. During the study period, is the intervention administered (or exposure occurred) as intended? | O |  |  |  |
| 4. Quantitative descriptive | 4.1. Is the sampling strategy relevant to address the research question? |  |  |  |  |
|  | 4.2. Is the sample representative of the target population? |  |  |  |  |
|  | 4.3. Are the measurements appropriate? |  |  |  |  |
|  | 4.4. Is the risk of nonresponse bias low? |  |  |  |  |
|  | 4.5. Is the statistical analysis appropriate to answer the research question? |  |  |  |  |
| 5. Mixed methods | 5.1. Is there an adequate rationale for using a mixed methods design to address the research question? |  |  |  |  |
|  | 5.2. Are the different components of the study effectively integrated to answer the research question? |  |  |  |  |
|  | 5.3. Are the outputs of the integration of qualitative and quantitative components adequately interpreted? |  |  |  |  |
|  | 5.4. Are divergences and inconsistencies between quantitative and qualitative results adequately addressed? |  |  |  |  |
|  | 5.5. Do the different components of the study adhere to the quality criteria of each tradition of the methods involved? |  |  |  |  |

**Part II: Explanations**

| **3. Quantitative non-randomized studies** | **Methodological quality criteria** |
| --- | --- |
| Non-randomized studies are defined as any quantitative studies estimating the effectiveness of an intervention or studying other exposures that do not use randomization to allocate units to comparison groups (Higgins and Green, 2008).  Common designs include (this list is not exhaustive):  **Non-randomized controlled trials**  The intervention is assigned by researchers, but there is no randomization, e.g., a pseudo-randomization. A non- random method of allocation is not reliable in producing alone similar groups.  **Cohort study**  Subsets of a defined population are assessed as exposed, not exposed, or exposed at different degrees to factors of interest. Participants are followed over time to determine if an outcome occurs (prospective longitudinal).  **Case-control study**  Cases, e.g., patients, associated with a certain outcome are selected, alongside a corresponding group of controls.  Data is collected on whether cases and controls were exposed to the factor under study (retrospective).  **Cross-sectional analytic study**  At one particular time, the relationship between health- related characteristics (outcome) and other factors (intervention/exposure) is examined. E.g., the frequency of outcomes is compared in different population subgroups according to the presence/absence (or level) of the intervention/exposure.  Key references for non-randomized studies: Higgins and Green (2008); Porta et al. (2014); Sterne et al. (2016); Wells et al. (2000) | 3.1. Are the participants representative of the target population?  - Can’t tell  - Participants were college students recruited online and stratified by depression level, but generalizability to broader populations is unclear and not addressed. |
|  | 3.2. Are measurements appropriate regarding both the outcome and intervention (or exposure)?  - Yes  - Validated tools (PHQ-9, BDI-II, STAI, EMA) and sensor-based measures were used based on DSM-5 criteria |
|  | 3.3. Are there complete outcome data?  - Can’t tell  - Data loss and low EMA response (e.g., 38% at 7 a.m.) are reported; while enough data were analyzed (n=2046), completeness across participants is not fully clear. |
|  | 3.4. Are the confounders accounted for in the design and analysis?  - No  - Although some confounders (e.g., STAI) were measured, no statistical adjustment was performed. |
|  | 3.5 During the study period, is the intervention administered (or exposure occurred) as intended?  - Yes  - Protocol adherence was monitored via a dashboard, and participants used the app and devices as planned. |

**Part I: Mixed Methods Appraisal Tool (MMAT), version 2018**

1. **Multimodal Digital Phenotyping Study in Patients With Major Depressive Episodes and Healthy Controls (Mobile Monitoring of Mood): Observational Longitudinal Study**

| **Category of study designs** | **12. Multimodal Digital Phenotyping Study in Patients With Major Depressive Episodes and Healthy Controls (Mobile Monitoring of Mood): Observational Longitudinal Study** | **Responses** | | | |
| --- | --- | --- | --- | --- | --- |
|  |  | Yes | No | Can’t tell | Comments |
| Screening questions (for all types) | S1. Are there clear research questions? | O |  |  |  |
|  | S2. Do the collected data allow to address the research questions? | O |  |  |  |
|  |  | | | | |
| 1. Qualitative | 1.1. Is the qualitative approach appropriate to answer the research question? |  |  |  | Not applicable |
|  | 1.2. Are the qualitative data collection methods adequate to address the research question? |  |  |  | Not applicable |
|  | 1.3. Are the findings adequately derived from the data? |  |  |  | Not applicable |
|  | 1.4. Is the interpretation of results sufficiently substantiated by data? |  |  |  | Not applicable |
|  | 1.5. Is there coherence between qualitative data sources, collection, analysis and interpretation? |  |  |  | Not applicable |
| 2. Quantitative randomized controlled trials | 2.1. Is randomization appropriately performed? |  |  |  | Not RCT |
|  | 2.2. Are the groups comparable at baseline? |  |  |  | Not RCT |
|  | 2.3. Are there complete outcome data? |  |  |  | Not RCT |
|  | 2.4. Are outcome assessors blinded to the intervention provided? |  |  |  | Not RCT |
|  | 2.5 Did the participants adhere to the assigned intervention? |  |  |  | Not RCT |
| 3. Quantitative non- randomized | 3.1. Are the participants representative of the target population? | O |  |  |  |
|  | 3.2. Are measurements appropriate regarding both the outcome and intervention (or exposure)? | O |  |  |  |
|  | 3.3. Are there complete outcome data? |  | O |  |  |
|  | 3.4. Are the confounders accounted for in the design and analysis? |  |  | O |  |
|  | 3.5. During the study period, is the intervention administered (or exposure occurred) as intended? | O |  |  |  |
| 4. Quantitative descriptive | 4.1. Is the sampling strategy relevant to address the research question? |  |  |  | Not applicable |
|  | 4.2. Is the sample representative of the target population? |  |  |  | Not applicable |
|  | 4.3. Are the measurements appropriate? |  |  |  | Not applicable |
|  | 4.4. Is the risk of nonresponse bias low? |  |  |  | Not applicable |
|  | 4.5. Is the statistical analysis appropriate to answer the research question? |  |  |  | Not applicable |
| 5. Mixed methods | 5.1. Is there an adequate rationale for using a mixed methods design to address the research question? |  |  |  | Not applicable |
|  | 5.2. Are the different components of the study effectively integrated to answer the research question? |  |  |  | Not applicable |
|  | 5.3. Are the outputs of the integration of qualitative and quantitative components adequately interpreted? |  |  |  | Not applicable |
|  | 5.4. Are divergences and inconsistencies between quantitative and qualitative results adequately addressed? |  |  |  | Not applicable |
|  | 5.5. Do the different components of the study adhere to the quality criteria of each tradition of the methods involved? |  |  |  | Not applicable |
|  |  |  |  |  |  |

| **3. Quantitative non-randomized studies** | **12. Multimodal Digital Phenotyping Study in Patients With Major Depressive Episodes and Healthy Controls (Mobile Monitoring of Mood): Observational Longitudinal Study** |
| --- | --- |
| Non-randomized studies are defined as any quantitative studies estimating the effectiveness of an intervention or studying other exposures that do not use randomization to allocate units to comparison groups (Higgins and Green, 2008).  Common designs include (this list is not exhaustive):  **Cohort study**  Subsets of a defined population are assessed as exposed, not exposed, or exposed at different degrees to factors of interest. Participants are followed over time to determine if an outcome occurs (prospective longitudinal).  One or more groups (here, patients with various depressive disorders and healthy controls) are observed over time, and data are collected without intervention. An observational and longitudinal design that prospectively tracks participants for up to one year. | 3.1. Are the participants representative of the target population?   - **Yes** - If the target population of the study was more narrowly defined as "outpatient with mild to moderate depression who can and can use a smartphone," the inclusion/exclusion criteria and recruitment approach were in good agreement with that definition, and the sample was considered to be representative of that narrow population |
|  | 3.2. Are measurements appropriate regarding both the outcome and intervention (or exposure)? yes   - **Yes** - We used a proven tool for depression (PHQ-9), and sensor-based measurements are well documented in the digital phenotypic literature |
|  | 3.3. Are there complete outcome data?  - **No**  - "The study is limited by incomplete data, decreased compliance of participants, and increased dropout rates as the study progresses." The paper does not explicitly report the completeness of the resulting data (e.g., the percentage of missing or missing data during the follow-up period).  - Although the results are presented, there is insufficient information to assess whether all participants were included in the analysis or how the missing data was processed. Therefore, it is unclear whether the resulting data are complete. |
|  | 3.4. Are the confounders accounted for in the design and analysis?   - Can’t tell - Using a multivariate approach with key variables (age, gender, etc.) helps to reduce bias, but the paper alone is not entirely clear how thoroughly it captures or statistically adjusts for all relevant confounding factors. - Although some bias has been reduced due to partial adjustments, there is no clear and comprehensive explanation for all confounding being measured and modeled. |
|  | 3.5 During the study period, is the intervention administered (or exposure occurred) as intended?   - Yes - This was observational, so there was no experimental "intervention," but "exposure" (i.e., participants using personal devices and wearing sensors) occurred as the study intended. - The design of this study is to collect actual smartphone/actigraphy/bed sensor data for up to one year in an outpatient setting. This paper does not specify that the data collection protocol itself has been changed or discontinued in a way that invalidates the intended exposure. - Many participants were eventually eliminated, but the remaining participants followed the original data collection procedure. There is no evidence that the data collection approach has changed |

**Part II: Explanations**

**Part I: Mixed Methods Appraisal Tool (MMAT), version 2018**

1. **Exploring actigraphy as a digital phenotyping measure: A study on differentiating psychomotor agitation and retardation in depression**

| **Category of study designs** | **13. Exploring actigraphy as a digital phenotyping measure: A study on differentiating psychomotor agitation and retardation in depression** | **Responses** | | | |
| --- | --- | --- | --- | --- | --- |
|  |  | Yes | No | Can’t tell | Comments |
| Screening questions (for all types) | S1. Are there clear research questions? | O |  |  |  |
|  | S2. Do the collected data allow to address the research questions? | O |  |  |  |
|  |  | | | | |
| 1. Qualitative | 1.1. Is the qualitative approach appropriate to answer the research question? |  |  |  | Not applicable |
|  | 1.2. Are the qualitative data collection methods adequate to address the research question? |  |  |  | Not applicable |
|  | 1.3. Are the findings adequately derived from the data? |  |  |  | Not applicable |
|  | 1.4. Is the interpretation of results sufficiently substantiated by data? |  |  |  | Not applicable |
|  | 1.5. Is there coherence between qualitative data sources, collection, analysis and interpretation? |  |  |  | Not applicable |
| 2. Quantitative randomized controlled trials | 2.1. Is randomization appropriately performed? |  |  |  | Not applicable |
|  | 2.2. Are the groups comparable at baseline? |  |  |  | Not applicable |
|  | 2.3. Are there complete outcome data? |  |  |  | Not applicable |
|  | 2.4. Are outcome assessors blinded to the intervention provided? |  |  |  | Not applicable |
|  | 2.5 Did the participants adhere to the assigned intervention? |  |  |  | Not applicable |
| 3. Quantitative non- randomized | 3.1. Are the participants representative of the target population? | O |  |  |  |
|  | 3.2. Are measurements appropriate regarding both the outcome and intervention (or exposure)? | O |  |  |  |
|  | 3.3. Are there complete outcome data? | O |  |  |  |
|  | 3.4. Are the confounders accounted for in the design and analysis? | O |  |  |  |
|  | 3.5. During the study period, is the intervention administered (or exposure occurred) as intended? | O |  |  |  |
| 4. Quantitative descriptive | 4.1. Is the sampling strategy relevant to address the research question? |  |  |  | Not applicable |
|  | 4.2. Is the sample representative of the target population? |  |  |  | Not applicable |
|  | 4.3. Are the measurements appropriate? |  |  |  | Not applicable |
|  | 4.4. Is the risk of nonresponse bias low? |  |  |  | Not applicable |
|  | 4.5. Is the statistical analysis appropriate to answer the research question? |  |  |  | Not applicable |
| 5. Mixed methods | 5.1. Is there an adequate rationale for using a mixed methods design to address the research question? |  | O |  |  |
|  | 5.2. Are the different components of the study effectively integrated to answer the research question? |  | O |  |  |
|  | 5.3. Are the outputs of the integration of qualitative and quantitative components adequately interpreted? |  | O |  |  |
|  | 5.4. Are divergences and inconsistencies between quantitative and qualitative results adequately addressed? |  | O |  |  |
|  | 5.5. Do the different components of the study adhere to the quality criteria of each tradition of the methods involved? |  | O |  |  |

**Part II: Explanations**

| **3. Quantitative non-randomized studies** | **13. Exploring actigraphy as a digital phenotyping measure: A study on differentiating psychomotor agitation and retardation in depression** |
| --- | --- |
| Non-randomized studies are defined as any quantitative studies estimating the effectiveness of an intervention or studying other exposures that do not use randomization to allocate units to comparison groups (Higgins and Green, 2008).  **Case-control study**  Based on the Methods section (see especially where the authors describe it as a “naturalistic case–control study conducted in routine care”), this paper is a quantitative, non‐randomized study. It compares two existing groups of patients (with psychomotor retardation vs. psychomotor agitation) without any random assignment to interventions or arms, and it uses quantitative outcome measures (questionnaires and actigraphy). | 3.1. Are the participants representative of the target population?   - **Yes** - Participants recruited from regular psychiatric practice, expressing clear inclusion criteria |
|  | 3.2. Are measurements appropriate regarding both the outcome and intervention (or exposure)?   - **Yes** - They used standard DSM‐5 diagnostic criteria, validated questionnaires (e.g., Insomnia Severity Index, Pittsburg Sleep Quality Index) for sleep and depression parameters, and objective actigraphy—which is “noninvasive digital method for collecting objective activity and sleep–wake rhythms.” The only caveat: they did not use specific psychomotor‐rating scales (e.g. MARS), but instead relied on the DSM‐5 criteria for psychomotor agitation/retardation. |
|  | 3.3. Are there complete outcome data?   - **Yes** - The final sample (n=74) all wore the actigraph for ≥14 days; “All patients wore the actiwatch for at least 14 days.” There is no mention of missing or excluded actigraphy data in the results. |
|  | 3.4. Are the confounders accounted for in the design and analysis?   - **Yes** - The authors state: “To account for potential confounding factors, any variables that were significantly different (p < 0.05) between groups were then entered into a binomial logistic regression analysis.” Specifically, they adjusted for differences in current tobacco smoking and current alcohol use disorder. |
|  | 3.5 During the study period, is the intervention administered (or exposure occurred) as intended?   - **Yes** - Although this was an observational comparison (rather than a trial intervention), the “exposure” (i.e., being in the “psychomotor agitation” or “psychomotor retardation” group) did not change during the study; participants were consistently grouped by DSM‐5 criteria, and data collection (actigraphy, questionnaires) proceeded as described. |

**Part I: Mixed Methods Appraisal Tool (MMAT), version 2018**

1. **Using machine learning with intensive longitudinal data to predict depression and suicidal ideation among medical interns over time**

| **Category of study designs** | **14. Using machine learning with intensive longitudinal data to predict depression and suicidal ideation among medical interns over time** | **Responses** | | | |
| --- | --- | --- | --- | --- | --- |
|  |  | Yes | No | Can’t tell | Comments |
| Screening questions (for all types) | S1. Are there clear research questions? | O |  |  |  |
|  | S2. Do the collected data allow to address the research questions? | O |  |  |  |
|  |  | | | | |
| 1. Qualitative | 1.1. Is the qualitative approach appropriate to answer the research question? |  |  |  | Not applicable |
|  | 1.2. Are the qualitative data collection methods adequate to address the research question? |  |  |  | Not applicable |
|  | 1.3. Are the findings adequately derived from the data? |  |  |  | Not applicable |
|  | 1.4. Is the interpretation of results sufficiently substantiated by data? |  |  |  | Not applicable |
|  | 1.5. Is there coherence between qualitative data sources, collection, analysis and interpretation? |  |  |  | Not applicable |
| 2. Quantitative randomized controlled trials | 2.1. Is randomization appropriately performed? |  |  |  | Not applicable |
|  | 2.2. Are the groups comparable at baseline? |  |  |  | Not applicable |
|  | 2.3. Are there complete outcome data? | O |  |  | Not applicable |
|  | 2.4. Are outcome assessors blinded to the intervention provided? |  |  |  | Not applicable |
|  | 2.5 Did the participants adhere to the assigned intervention? |  |  |  | Not applicable |
| 3. Quantitative non- randomized | 3.1. Are the participants representative of the target population? | O |  |  |  |
|  | 3.2. Are measurements appropriate regarding both the outcome and intervention (or exposure)? | O |  |  |  |
|  | 3.3. Are there complete outcome data? | O |  |  |  |
|  | 3.4. Are the confounders accounted for in the design and analysis? |  |  | O |  |
|  | 3.5. During the study period, is the intervention administered (or exposure occurred) as intended? | O |  |  |  |
| 4. Quantitative descriptive | 4.1. Is the sampling strategy relevant to address the research question? |  |  |  |  |
|  | 4.2. Is the sample representative of the target population? |  |  |  |  |
|  | 4.3. Are the measurements appropriate? |  |  |  |  |
|  | 4.4. Is the risk of nonresponse bias low? |  |  |  |  |
|  | 4.5. Is the statistical analysis appropriate to answer the research question? |  |  |  |  |
| 5. Mixed methods | 5.1. Is there an adequate rationale for using a mixed methods design to address the research question? |  | O |  |  |
|  | 5.2. Are the different components of the study effectively integrated to answer the research question? |  | O |  |  |
|  | 5.3. Are the outputs of the integration of qualitative and quantitative components adequately interpreted? |  | O |  |  |
|  | 5.4. Are divergences and inconsistencies between quantitative and qualitative results adequately addressed? |  | O |  |  |
|  | 5.5. Do the different components of the study adhere to the quality criteria of each tradition of the methods involved? |  | O |  |  |

**Part II: Explanations**

| **3. Quantitative non-randomized studies** | **14. Using machine learning with intensive longitudinal data to predict depression and suicidal ideation among medical interns over time** |
| --- | --- |
| Non-randomized studies are defined as any quantitative studies estimating the effectiveness of an intervention or studying other exposures that do not use randomization to allocate units to comparison groups (Higgins and Green, 2008).  **Cohort study**  The authors prospectively collected daily mood ratings and passively sensed data (e.g., Fitbit information), but they did not randomly assign participants to any intervention or control condition (which rules out a randomized controlled trial), nor did they employ qualitative or mixed-methods procedures. Essentially, it is a **prospective cohort study** using quantitative measures, making **“quantitative non-randomized”** the most accurate classification.  The investigators enrolled a large group of first-year medical interns (the “cohort”) and followed them prospectively over time, repeatedly measuring their depressive symptoms, suicidal ideation, and wearable-derived outcomes to see how these measures changed and how well they predicted later outcomes. There was no intervention or control group assignment (ruling out non-randomized trials), there were no matched “case” and “control” groups (ruling out case-control), and the data collection was not limited to one time point (ruling out a cross-sectional study). | 3.1. Are the participants representative of the target population?   - Yes - The study recruited 2,459 first-year medical interns training at over 300 residency institutions throughout the United States, which closely reflects the national intern population in terms of location and diversity. |
|  | 3.2. Are measurements appropriate regarding both the outcome and intervention (or exposure)?   - Yes - The primary outcome measures—depression and suicidal ideation—were assessed using the PHQ-9, a well-validated measure for depressive symptoms and suicidal thoughts. - Daily mood ratings were collected via a simple 1–10 scale, and wearable data (Fitbit) were used to capture sleep and activity patterns. These are widely accepted approaches for assessing mood and behavior. |
|  | 3.3. Are there complete outcome data?   - Yes - The paper indicates that 2,459 interns were included in the analysis and that, at the end-of-quarter follow-up assessment, the authors had PHQ-9 (depression) data for those interns. That allowed them to report the prevalence of depression (18.5%) and suicidal ideation (6.8%) in this same group. - For missing daily (exposure) data, the authors used a well-defined procedure: if there were not enough observations to calculate a participant’s daily feature (e.g., average mood or activity), they substituted the median value from the training set. Therefore, each participant’s quarter-end outcome status (PHQ-9) appears to have been measured and handled consistently, providing complete or fully accounted-for outcome data. |
|  | 3.4. Are the confounders accounted for in the design and analysis?   - Can’t tell - The paper does not explicitly state that investigators identified and statistically controlled for specific confounders (e.g., baseline mood, demographics, or other clinical factors). - The authors used predictive machine-learning models (rather than a traditional causal analysis) and did not clearly describe using any “multivariate adjustment” aimed at reducing confounding in the usual epidemiological sense. - Although they fed multiple variables (daily mood, Fitbit features) into the model, doing so for prediction does not necessarily equate to systematically accounting for confounders in the sense of adjusting for potential biases. |
|  | 3.5 During the study period, is the intervention administered (or exposure occurred) as intended?   - Yes - The “exposure” in this observational cohort study is the typical day-to-day experience of first-year medical internship. The article does not indicate any deviation from that routine internship experience, so it is reasonable to conclude the exposure occurred as intended under real-world residency conditions. |

**Part I: Mixed Methods Appraisal Tool (MMAT), version 2018**

1. **Identifying Objective Physiological Markers and Modifiable Behaviors for Self-Reported Stress and Mental Health Status Using Wearable Sensors and Mobile Phones:**

| **Category of study designs** | **16. Identifying Objective Physiological Markers and Modifiable Behaviors for Self-Reported Stress and Mental Health Status Using Wearable Sensors and Mobile Phones: Observational Study** | **Responses** | | | | |
| --- | --- | --- | --- | --- | --- | --- |
|  |  | Yes | No | Can’t tell | Comments |  |
| Screening questions (for all types) | S1. Are there clear research questions? | O |  |  |  |  |
|  | S2. Do the collected data allow to address the research questions? | O |  |  |  |  |
|  |  | | | | |  |
| 1. Qualitative | 1.1. Is the qualitative approach appropriate to answer the research question? |  |  |  | Not applicable |  |
|  | 1.2. Are the qualitative data collection methods adequate to address the research question? |  |  |  | Not applicable |  |
|  | 1.3. Are the findings adequately derived from the data? |  |  |  | Not applicable |  |
|  | 1.4. Is the interpretation of results sufficiently substantiated by data? |  |  |  | Not applicable |  |
|  | 1.5. Is there coherence between qualitative data sources, collection, analysis and interpretation? |  |  |  | Not applicable |  |
| 2. Quantitative randomized controlled trials | 2.1. Is randomization appropriately performed? |  |  |  | Not applicable |  |
|  | 2.2. Are the groups comparable at baseline? |  |  |  | Not applicable |  |
|  | 2.3. Are there complete outcome data? |  |  |  | Not applicable |  |
|  | 2.4. Are outcome assessors blinded to the intervention provided? |  |  |  | Not applicable |  |
|  | 2.5 Did the participants adhere to the assigned intervention? |  |  |  | Not applicable |  |
| 3. Quantitative non- randomized | 3.1. Are the participants representative of the target population? |  |  | O |  |  |
|  | 3.2. Are measurements appropriate regarding both the outcome and intervention (or exposure)? | O |  |  |  |  |
|  | 3.3. Are there complete outcome data? |  | O |  |  |  |
|  | 3.4. Are the confounders accounted for in the design and analysis? | O |  |  |  |  |
|  | 3.5. During the study period, is the intervention administered (or exposure occurred) as intended? |  |  | O |  |  |
| 4. Quantitative descriptive | 4.1. Is the sampling strategy relevant to address the research question? |  |  |  |  |  |
|  | 4.2. Is the sample representative of the target population? |  |  |  |  |  |
|  | 4.3. Are the measurements appropriate? |  |  |  |  |  |
|  | 4.4. Is the risk of nonresponse bias low? |  |  |  |  |  |
|  | 4.5. Is the statistical analysis appropriate to answer the research question? |  |  |  |  |  |
| 5. Mixed methods | 5.1. Is there an adequate rationale for using a mixed methods design to address the research question? |  |  |  | Not applicable |  |
|  | 5.2. Are the different components of the study effectively integrated to answer the research question? |  |  |  | Not applicable |  |
|  | 5.3. Are the outputs of the integration of qualitative and quantitative components adequately interpreted? |  |  |  | Not applicable |  |
|  | 5.4. Are divergences and inconsistencies between quantitative and qualitative results adequately addressed? |  |  |  | Not applicable |  |
|  | 5.5. Do the different components of the study adhere to the quality criteria of each tradition of the methods involved? |  |  |  | Not applicable |  |

**Part II: Explanations**

| **3. Quantitative non-randomized studies** | **16. Identifying Objective Physiological Markers and Modifiable Behaviors for Self-Reported Stress and Mental Health Status Using Wearable Sensors and Mobile Phones: Observational Study** |
| --- | --- |
| Non-randomized studies are defined as any quantitative studies estimating the effectiveness of an intervention or studying other exposures that do not use randomization to allocate units to comparison groups (Higgins and Green, 2008).  Common designs include (this list is not exhaustive):  **Cohort study**  They enrolled participants and tracked them with wearable sensors and phone data. No trial or random assignment is mentioned. Instead, it is an observational study in which the participants simply go about their normal daily lives:  “During the month, each student completed twice-daily electronic diaries, wore two wrist-based sensors ... and installed an app on their mobile phone... We ... applied machine learning ... to identify factors associated with self-reported poststudy stress and mental health, including behaviors that can possibly be modified...” (Abstract, “Methods”)  Hence, it does not test a therapy or compare groups under different interventions—there is no “treatment” or “control” assignment.  They recruited a group (a “cohort”) of students and observed them prospectively over several weeks, taking continuous measurements (physiology, phone use) plus start/end questionnaires to identify associations with stress and mental health. That is exactly what defines a cohort design. | 3.1. Are the participants representative of the target population?   - Can’t tell - It depends on how you define the target population. If your intended population is literally “college students aged 18–25 at one New England university who are Android users,” then this sample is indeed fairly representative of that narrow group. - However, if your ultimate goal is to generalize to all U.S. college students in that age range, or to a broader set of young adults, then a single-campus sample with predominantly Android users and a skewed male–female ratio isn’t necessarily representative. |
|  | 3.2. Are measurements appropriate regarding both the outcome and intervention (or exposure)?   - Yes - For stress and mental health outcomes, they used validated self-report scales, i.e. Perceived Stress Scale (PSS) and the Mental Component Summary (MCS) from the SF-12. - For exposure/behavior measures (like phone usage, physiology, sleep/wake times), they used well-documented wearable sensors and phone-data logs, with known reliability in capturing physical activity, skin conductance, etc. - Each measurement (e.g., PSS for stress, MCS for mental health, wearable/phone data for behaviors) is recognized as appropriate and validated in the literature. |
|  | 3.3. Are there complete outcome data?   - No - Not All Participants Provided Final Outcome Measures - Partial Sensor Coverage: They mention that phone data were collected on only about 85% of participant-days, and approximately 80% of the collected skin conductance data were usable after cleaning. Even though these are good data-capture rates, they are not 100%. - Authors’ Own Acknowledgments: They explicitly reference participants with missing diaries and missing sensor data. Some diaries were incomplete; some participants dropped out or removed sensors. |
|  | 3.4. Are the confounders accounted for in the design and analysis?   - Can’t tell - They do incorporate some potential confounders (e.g., gender, personality traits) within their machine-learning features. - Although they include a wide range of features, they do not explicitly report full confounder-adjustment in design/analysis. It is unclear whether all key confounders are adequately addressed. |
|  | 3.5 During the study period, is the intervention administered (or exposure occurred) as intended?   - Yes - Although there is no true “intervention” in the experimental sense, the planned “exposure” was that participants would continuously wear the sensors and use their phone with the custom data-collection app for ~1 month. - The paper confirms they periodically checked data and coached participants to keep devices on. They ultimately collected ~145,000 hours of sensor data and ~85% phone data coverage. - The study’s intended “exposure” (ie, continuous sensing from wearable + phone) was indeed carried out for nearly the entire sample, even though some data were missing. Overall, the procedure as designed did occur. |

**Part I: Mixed Methods Appraisal Tool (MMAT), version 2018**

1. **Comprehensive Symptom Prediction in Inpatients With Acute Psychiatric Disorders Using Wearable-Based Deep Learning Models: Development and Validation Study**

| **Category of study designs** | **Methodological quality criteria** | **Responses** | | | |
| --- | --- | --- | --- | --- | --- |
|  |  | Yes | No | Can’t tell | Comments |
| Screening questions (for all types) | S1. Are there clear research questions? | V |  |  |  |
|  | S2. Do the collected data allow to address the research questions? | V |  |  |  |
|  | *Further appraisal may not be feasible or appropriate when the answer is ‘No’ or ‘Can’t tell’ to one or both screening questions.* | | | | |
| 1. Qualitative | 1.1. Is the qualitative approach appropriate to answer the research question? |  |  |  |  |
|  | 1.2. Are the qualitative data collection methods adequate to address the research question? |  |  |  |  |
|  | 1.3. Are the findings adequately derived from the data? |  |  |  |  |
|  | 1.4. Is the interpretation of results sufficiently substantiated by data? |  |  |  |  |
|  | 1.5. Is there coherence between qualitative data sources, collection, analysis and interpretation? |  |  |  |  |
| 2. Quantitative randomized controlled trials | 2.1. Is randomization appropriately performed? |  |  |  |  |
|  | 2.2. Are the groups comparable at baseline? |  |  |  |  |
|  | 2.3. Are there complete outcome data? |  |  |  |  |
|  | 2.4. Are outcome assessors blinded to the intervention provided? |  |  |  |  |
|  | 2.5 Did the participants adhere to the assigned intervention? |  |  |  |  |
| 3. Quantitative non- randomized | 3.1. Are the participants representative of the target population? | V |  |  |  |
|  | 3.2. Are measurements appropriate regarding both the outcome and intervention (or exposure)? | V |  |  |  |
|  | 3.3. Are there complete outcome data? | V |  |  |  |
|  | 3.4. Are the confounders accounted for in the design and analysis? | V |  |  |  |
|  | 3.5. During the study period, is the intervention administered (or exposure occurred) as intended? | V |  |  |  |
| 4. Quantitative descriptive | 4.1. Is the sampling strategy relevant to address the research question? |  |  |  |  |
|  | 4.2. Is the sample representative of the target population? |  |  |  |  |
|  | 4.3. Are the measurements appropriate? |  |  |  |  |
|  | 4.4. Is the risk of nonresponse bias low? |  |  |  |  |
|  | 4.5. Is the statistical analysis appropriate to answer the research question? |  |  |  |  |
| 5. Mixed methods | 5.1. Is there an adequate rationale for using a mixed methods design to address the research question? |  |  |  |  |
|  | 5.2. Are the different components of the study effectively integrated to answer the research question? |  |  |  |  |
|  | 5.3. Are the outputs of the integration of qualitative and quantitative components adequately interpreted? |  |  |  |  |
|  | 5.4. Are divergences and inconsistencies between quantitative and qualitative results adequately addressed? |  |  |  |  |
|  | 5.5. Do the different components of the study adhere to the quality criteria of each tradition of the methods involved? |  |  |  |  |

| **3. Quantitative non-randomized studies** | **Methodological quality criteria** |
| --- | --- |
| Non-randomized studies are defined as any quantitative studies estimating the effectiveness of an intervention or studying other exposures that do not use randomization to allocate units to comparison groups (Higgins and Green, 2008).  Common designs include (this list is not exhaustive):  **Non-randomized controlled trials**  The intervention is assigned by researchers, but there is no randomization, e.g., a pseudo-randomization. A non- random method of allocation is not reliable in producing alone similar groups.  **Cohort study**  Subsets of a defined population are assessed as exposed, not exposed, or exposed at different degrees to factors of interest. Participants are followed over time to determine if an outcome occurs (prospective longitudinal).  **Case-control study**  Cases, e.g., patients, associated with a certain outcome are selected, alongside a corresponding group of controls.  Data is collected on whether cases and controls were exposed to the factor under study (retrospective).  **Cross-sectional analytic study**  At one particular time, the relationship between health- related characteristics (outcome) and other factors (intervention/exposure) is examined. E.g., the frequency of outcomes is compared in different population subgroups according to the presence/absence (or level) of the intervention/exposure. Key references for non-randomized studies: Higgins and Green (2008); Porta et al. (2014); Sterne et al. (2016); Wells et al. (2000) | 3.1. Are the participants representative of the target population?  - Yes  - The study population is clearly defined as inpatients in acute psychiatric wards. - The recruited patients were collected from various types of hospitals (e.g., university hospitals, psychiatric specialty hospitals) and regions, ensuring  diversity. |
|  | 3.2. Are measurements appropriate regarding both the outcome and intervention (or exposure)?  - Yes  - **Outcome**: Standard psychiatric clinical scales were used (BPRS, HAM-A, MADRS, YMRS), all of which are validated and reliable tools. **- Exposure**: Data such as heart rate, accelerometry, sleep index, and location entropy were quantitatively and precisely measured using wearable  sensors. - The variables were clearly defined and appropriately processed as input features for the AI model. |
|  | 3.3. Are there complete outcome data?  - Yes  - Out of a total of 244 participants, 191 (78.3%) were included in the final analysis. - Among the 53 excluded participants, some had more than 50% missing sensor data or did not complete at least one clinical assessment. - The final sample included an average of 4.3 clinical assessments and 20.7 days of observation data, which is considered sufficient. |
|  | 3.4. Are the confounders accounted for in the design and analysis?  - Yes  - In addition to sensor data, variables such as gender and age were included in the model for adjustment. - The study discussed heterogeneity in sensor features across wards and acknowledged potential sources of bias through data visualization and analysis. |
|  | 3.5 During the study period, is the intervention administered (or exposure occurred) as intended?  - Yes  - All participants wore wearable devices continuously during their inpatient stay in a controlled hospital environment, and data were collected in a  consistent manner. - A substantial amount of valid data was collected, and missing data were appropriately handled. - Meaningful AI prediction models were developed using the wearable data, achieving performance levels such as an AUC of 0.82 for BPRS and 0.78  for MADRS. |

**Part II: Explanations**

**Part I: Mixed Methods Appraisal Tool (MMAT), version 2018**

**21.** **Investigating the Feasibility of Assessing Depression Severity and Valence-Arousal with Wearable Sensors Using Discrete Wavelet Transforms and Machine Learning**

| **Category of study designs** | **Methodological quality criteria** | **Responses** | | | |
| --- | --- | --- | --- | --- | --- |
|  |  | Yes | No | Can’t tell | Comments |
| Screening questions (for all types) | S1. Are there clear research questions? | V |  |  |  |
|  | S2. Do the collected data allow to address the research questions? | V |  |  |  |
|  | *Further appraisal may not be feasible or appropriate when the answer is ‘No’ or ‘Can’t tell’ to one or both screening questions.* | | | | |
| 1. Qualitative | 1.1. Is the qualitative approach appropriate to answer the research question? |  |  |  |  |
|  | 1.2. Are the qualitative data collection methods adequate to address the research question? |  |  |  |  |
|  | 1.3. Are the findings adequately derived from the data? |  |  |  |  |
|  | 1.4. Is the interpretation of results sufficiently substantiated by data? |  |  |  |  |
|  | 1.5. Is there coherence between qualitative data sources, collection, analysis and interpretation? |  |  |  |  |
| 2. Quantitative randomized controlled trials | 2.1. Is randomization appropriately performed? |  |  |  |  |
|  | 2.2. Are the groups comparable at baseline? |  |  |  |  |
|  | 2.3. Are there complete outcome data? |  |  |  |  |
|  | 2.4. Are outcome assessors blinded to the intervention provided? |  |  |  |  |
|  | 2.5 Did the participants adhere to the assigned intervention? |  |  |  |  |
| 3. Quantitative non- randomized | 3.1. Are the participants representative of the target population? |  |  |  |  |
|  | 3.2. Are measurements appropriate regarding both the outcome and intervention (or exposure)? |  |  |  |  |
|  | 3.3. Are there complete outcome data? |  |  |  |  |
|  | 3.4. Are the confounders accounted for in the design and analysis? |  |  |  |  |
|  | 3.5. During the study period, is the intervention administered (or exposure occurred) as intended? |  |  |  |  |
| 4. Quantitative descriptive | 4.1. Is the sampling strategy relevant to address the research question? | V |  | Out of the total 142 participants, only 87 provided valid physiological signals, indicating a high rate of nonresponse or invalid data. | |
|  | 4.2. Is the sample representative of the target population? | V |  |  |  |
|  | 4.3. Are the measurements appropriate? | V |  |  |  |
|  | 4.4. Is the risk of nonresponse bias low? |  | V |  |  |
|  | 4.5. Is the statistical analysis appropriate to answer the research question? | V |  |  |  |
| 5. Mixed methods | 5.1. Is there an adequate rationale for using a mixed methods design to address the research question? |  |  |  |  |
|  | 5.2. Are the different components of the study effectively integrated to answer the research question? |  |  |  |  |
|  | 5.3. Are the outputs of the integration of qualitative and quantitative components adequately interpreted? |  |  |  |  |
|  | 5.4. Are divergences and inconsistencies between quantitative and qualitative results adequately addressed? |  |  |  |  |
|  | 5.5. Do the different components of the study adhere to the quality criteria of each tradition of the methods involved? |  |  |  |  |

| **4. Quantitative descriptive studies** | **Methodological quality criteria** |
| --- | --- |
| Quantitative descriptive studies are “concerned with and designed only to describe the existing distribution of variables without much regard to causal relationships or other hypotheses” (Porta et al., 2014, p. 72). They are used to monitoring the population, planning, and generating hypothesis (Grimes and Schulz, 2002).  Common designs include the following single-group studies (this list is not exhaustive):  **Incidence or prevalence study without comparison group**  In a defined population at one particular time, what is happening in a population, e.g., frequencies of factors (importance of problems), is described (portrayed).  **Survey**  “Research method by which information is gathered by asking people questions on a specific topic and the data collection procedure is standardized and well defined.” (Bennett et al., 2011, p. 3).  **Case series**  A collection of individuals with similar characteristics are used to describe an outcome.  **Case report**  An individual or a group with a unique/unusual outcome is described in detail.  Key references: Critical Appraisal Skills Programme (2017); Draugalis et al. (2008) | 4.1. Is the sampling strategy relevant to address the research question?   - **Can’t tell** - There is no clear explanation of how participants were recruited (e.g., random selection or voluntary participation), nor of the inclusion/exclusion criteria. - There is insufficient information to determine whether the sampling strategy was appropriate for the research question. |
|  | 4.2. Is the sample representative of the target population?  **- Yes**   - The target population of the study is clearly defined as individuals with moderate to severe depression. - All participants (BDI-II ≥ 21) met this criterion, aligning with the research objective. - The data were collected in a naturalistic setting, enhancing the real-world representativeness of the sample. |
|  | 4.3. Are the measurements appropriate?  **- Yes**  - Valid and reliable measurement tools were used, including the BDI-II for assessing depression severity, ESM and DRM for measuring emotional states, and wearable devices to collect physiological data such as HR, GSR, and ACC. |
|  | 4.4. Is the risk of nonresponse bias low?  **- Can’t tell**  - Only a brief explanation regarding missing data is provided, and there is no information on the characteristics of non-respondents, differences between respondents and non-respondents, response rates, or any adjustment procedures.  Therefore, it is difficult to assess the risk of nonresponse bias. |
|  | 4.5. Is the statistical analysis appropriate to answer the research question?  **- Yes**  - The analysis strategy was appropriately designed to address the research question, using comparisons between single and multimodal models  along with evaluation metrics such as Accuracy, F1-score, Sensitivity, Specificity, and AUC.  - Appropriate data augmentation techniques, such as SMOTE and ADASYN, were also applied to address class imbalance. |

**Part II: Explanations**

**Part I: Mixed Methods Appraisal Tool (MMAT), version 2018
24. Using digital phenotyping to capture depression symptom variability: detecting naturalistic variability in depression symptoms across one year using passively collected wearable movement and sleep data; Price et al(2023)**

| **Category of study designs** | **Methodological quality criteria** | **Responses** | | | |
| --- | --- | --- | --- | --- | --- |
|  |  | Yes | No | Can’t tell | Comments |
| Screening questions (for all types) | S1. Are there clear research questions? | O |  |  |  |
|  | S2. Do the collected data allow to address the research questions? | O |  |  |  |
|  | *Further appraisal may not be feasible or appropriate when the answer is ‘No’ or ‘Can’t tell’ to one or both screening questions.* | | | | |
| 1. Qualitative | 1.1. Is the qualitative approach appropriate to answer the research question? |  |  |  |  |
|  | 1.2. Are the qualitative data collection methods adequate to address the research question? |  |  |  |  |
|  | 1.3. Are the findings adequately derived from the data? |  |  |  |  |
|  | 1.4. Is the interpretation of results sufficiently substantiated by data? |  |  |  |  |
|  | 1.5. Is there coherence between qualitative data sources, collection, analysis and interpretation? |  |  |  |  |
| 2. Quantitative randomized controlled trials | 2.1. Is randomization appropriately performed? |  |  |  |  |
|  | 2.2. Are the groups comparable at baseline? |  |  |  |  |
|  | 2.3. Are there complete outcome data? |  |  |  |  |
|  | 2.4. Are outcome assessors blinded to the intervention provided? |  |  |  |  |
|  | 2.5 Did the participants adhere to the assigned intervention? |  |  |  |  |
| 3. Quantitative non- randomized | 3.1. Are the participants representative of the target population? |  |  | O |  |
|  | 3.2. Are measurements appropriate regarding both the outcome and intervention (or exposure)? | O |  |  |  |
|  | 3.3. Are there complete outcome data? |  | O |  |  |
|  | 3.4. Are the confounders accounted for in the design and analysis? |  |  | O |  |
|  | 3.5. During the study period, is the intervention administered (or exposure occurred) as intended? | O |  |  |  |
| 4. Quantitative descriptive | 4.1. Is the sampling strategy relevant to address the research question? |  |  |  |  |
|  | 4.2. Is the sample representative of the target population? |  |  |  |  |
|  | 4.3. Are the measurements appropriate? |  |  |  |  |
|  | 4.4. Is the risk of nonresponse bias low? |  |  |  |  |
|  | 4.5. Is the statistical analysis appropriate to answer the research question? |  |  |  |  |
| 5. Mixed methods | 5.1. Is there an adequate rationale for using a mixed methods design to address the research question? |  |  |  |  |
|  | 5.2. Are the different components of the study effectively integrated to answer the research question? |  |  |  |  |
|  | 5.3. Are the outputs of the integration of qualitative and quantitative components adequately interpreted? |  |  |  |  |
|  | 5.4. Are divergences and inconsistencies between quantitative and qualitative results adequately addressed? |  |  |  |  |
|  | 5.5. Do the different components of the study adhere to the quality criteria of each tradition of the methods involved? |  |  |  |  |

**Part II: Explanation**

| **3. Quantitative non-randomized studies** | **Methodological quality criteria** |
| --- | --- |
| Non-randomized studies are defined as any quantitative studies estimating the effectiveness of an intervention or studying other exposures that do not use randomization to allocate units to comparison groups (Higgins and Green, 2008).  Common designs include (this list is not exhaustive):  **Non-randomized controlled trials**  The intervention is assigned by researchers, but there is no randomization, e.g., a pseudo-randomization. A non-random method of allocation is not reliable in producing similar groups.  **Cohort study**  Subsets of a defined population are assessed as exposed, not exposed, or exposed at different degrees to factors of interest. Participants are followed over time to determine if an outcome occurs (prospective longitudinal).  **Case-control study**  Cases, e.g., patients, associated with a certain outcome are selected, alongside a corresponding group of controls.  Data is collected on whether cases and controls were exposed to the factor under study (retrospective).  **Cross-sectional analytic study**  At one particular time, the relationship between health- related characteristics (outcome) and other factors (intervention/exposure) is examined. E.g., the frequency of outcomes is compared in different population subgroups according to the presence/absence (or level) of the intervention/exposure.  Key references for non-randomized studies: Higgins and Green (2008); Porta et al. (2014); Sterne et al. (2016); Wells et al. (2000) | 3.1. Are the participants representative of the target population? Can’t tell  Participants were recruited from the Achievement platform, a commercial health research community, and were required to be users of Fitbit or Garmin devices with 12 months of continuous data and quarterly PHQ-9 assessments. While inclusion and exclusion criteria were clearly stated, the sample was limited to individuals who voluntarily use wearable devices and engage in app-based health tracking. There is no information about how many eligible individuals declined to participate or how the sample compares to the broader population with depression, thus limiting generalizability. |
|  | 3.2. Are measurements appropriate regarding both the outcome and intervention (or exposure)? Yes  The outcome—depression symptom variability—was operationalized using the root mean square of successive differences (RMSSD) in quarterly PHQ-9 scores over 12 months. Exposure data were passively collected from commercial wearable devices (Fitbit and Garmin) and included well-defined measures of mobility, sleep, and circadian rhythms. The variables were described clearly, and data preprocessing was applied to ensure reliability and consistency, including filtering for wear-time and signal quality. These support the appropriateness and validity of both outcome and exposure measurements. |
|  | 3.3. Are there complete outcome data? Yes  The final analytic sample included 939 participants who had at least five PHQ-9 assessments and 12 months of continuous wearable data. Multiple imputation (MICE) was used to address missing values, and strict inclusion criteria ensured that all participants contributed sufficient longitudinal data. There was no mention of attrition, and the consistency between recruited and analyzed participants suggests that outcome data were nearly complete. |
|  | 3.4. Are the confounders accounted for in the design and analysis? No  Although demographic, comorbidity, and behavioral features were included as predictors in the machine learning models, the study did not explicitly control for confounders through regression, matching, stratification, or other conventional methods. The variables were treated as model inputs rather than analytically adjusted for potential bias, limiting the ability to isolate the effects of specific predictors. |
|  | 3.5 During the study period, is the intervention administered (or exposure occurred) as intended? Yes  The exposure—passive data collection via wearable devices—was carried out as planned. Participants were required to have continuous data collection for one year, and only those who met wear-time and data quality thresholds were included. This ensured that the digital phenotyping exposure (i.e., behavioral and physiological tracking) occurred consistently and as intended across the study period. |

**Part I: Mixed Methods Appraisal Tool (MMAT), version 2018**

**26. Tracking and Monitoring Mood Stability of Patients With Major Depressive Disorder by Machine Learning Models Using Passive Digital Data: Prospective Naturalistic Multicenter Study, Ran(2021)**

| **Category of study designs** | **Methodological quality criteria** | **Responses** | | | |
| --- | --- | --- | --- | --- | --- |
|  |  | Yes | No | Can’t tell | Comments |
| Screening questions (for all types) | S1. Are there clear research questions? | O |  |  |  |
|  | S2. Do the collected data allow to address the research questions? | o |  |  |  |
|  | *Further appraisal may not be feasible or appropriate when the answer is ‘No’ or ‘Can’t tell’ to one or both screening questions.* | | | | |
| 1. Qualitative | 1.1. Is the qualitative approach appropriate to answer the research question? |  |  |  |  |
|  | 1.2. Are the qualitative data collection methods adequate to address the research question? |  |  |  |  |
|  | 1.3. Are the findings adequately derived from the data? |  |  |  |  |
|  | 1.4. Is the interpretation of results sufficiently substantiated by data? |  |  |  |  |
|  | 1.5. Is there coherence between qualitative data sources, collection, analysis and interpretation? |  |  |  |  |
| 2. Quantitative randomized controlled trials | 2.1. Is randomization appropriately performed? |  |  |  |  |
|  | 2.2. Are the groups comparable at baseline? |  |  |  |  |
|  | 2.3. Are there complete outcome data? |  |  |  |  |
|  | 2.4. Are outcome assessors blinded to the intervention provided? |  |  |  |  |
|  | 2.5 Did the participants adhere to the assigned intervention? |  |  |  |  |
| 3. Quantitative non- randomized | 3.1. Are the participants representative of the target population? |  |  | O |  |
|  | 3.2. Are measurements appropriate regarding both the outcome and intervention (or exposure)? | O |  |  |  |
|  | 3.3. Are there complete outcome data? | O |  |  |  |
|  | 3.4. Are the confounders accounted for in the design and analysis? |  | O |  |  |
|  | 3.5. During the study period, is the intervention administered (or exposure occurred) as intended? | o |  |  |  |
| 4. Quantitative descriptive | 4.1. Is the sampling strategy relevant to address the research question? |  |  |  |  |
|  | 4.2. Is the sample representative of the target population? |  |  |  |  |
|  | 4.3. Are the measurements appropriate? |  |  |  |  |
|  | 4.4. Is the risk of nonresponse bias low? |  |  |  |  |
|  | 4.5. Is the statistical analysis appropriate to answer the research question? |  |  |  |  |
| 5. Mixed methods | 5.1. Is there an adequate rationale for using a mixed methods design to address the research question? |  |  |  |  |
|  | 5.2. Are the different components of the study effectively integrated to answer the research question? |  |  |  |  |
|  | 5.3. Are the outputs of the integration of qualitative and quantitative components adequately interpreted? |  |  |  |  |
|  | 5.4. Are divergences and inconsistencies between quantitative and qualitative results adequately addressed? |  |  |  |  |
|  | 5.5. Do the different components of the study adhere to the quality criteria of each tradition of the methods involved? |  |  |  |  |

**Part II: Explanation**

| **3. Quantitative non-randomized studies** | **Methodological quality criteria** |
| --- | --- |
| Non-randomized studies are defined as any quantitative studies estimating the effectiveness of an intervention or studying other exposures that do not use randomization to allocate units to comparison groups (Higgins and Green, 2008).  Common designs include (this list is not exhaustive):  **Non-randomized controlled trials**  The intervention is assigned by researchers, but there is no randomization, e.g., a pseudo-randomization. A non- random method of allocation is not reliable in producing alone similar groups.  **Cohort study**  Subsets of a defined population are assessed as exposed, not exposed, or exposed at different degrees to factors of interest. Participants are followed over time to determine if an outcome occurs (prospective longitudinal).  **Case-control study**  Cases, e.g., patients, associated with a certain outcome are selected, alongside a corresponding group of controls.  Data is collected on whether cases and controls were exposed to the factor under study (retrospective).  **Cross-sectional analytic study**  At one particular time, the relationship between health- related characteristics (outcome) and other factors (intervention/exposure) is examined. E.g., the frequency of outcomes is compared in different population subgroups according to the presence/absence (or level) of the intervention/exposure.  Key references for non-randomized studies: Higgins and Green (2008); Porta et al. (2014); Sterne et al. (2016); Wells et al. (2000) | 3.1. Are the participants representative of the target population?  Can’t tell  The study enrolled 334 outpatients with major depressive disorder (MDD) from four psychiatric hospitals in Beijing, China. Although inclusion and exclusion criteria were well-defined (e.g., PHQ-9 ≥ 5, Android phone users, no psychotic symptoms), the recruitment was limited to Android users, which may not reflect the general MDD population. Furthermore, no information is provided regarding how many eligible individuals declined participation or how the sample compares demographically to the wider MDD population. |
|  | 3.2. Are measurements appropriate regarding both the outcome and intervention (or exposure)?  **Yes**  Outcome measures were derived from PHQ-9 assessments conducted every two weeks over a 12-week period, with mood variability categorized based on the range of PHQ-9 scores. Exposure data—including sleep, step count, heart rate, phone usage, and app logs—were collected passively using a wristband and the Mood Mirror app. Variables were clearly defined, and preprocessing included filtering invalid values and computing standardized features. These procedures reflect valid and appropriate measurements for both exposure and outcome. |
|  | 3.3. Are there complete outcome data?  Yes  The analysis included 950 data samples derived from 261 participants. Each sample consisted of at least two PHQ-9 assessments and corresponding wearable/smartphone data for the 3-week period in between. The study applied inclusion rules requiring a minimum data completion rate and excluded participants with excessive missing data. These procedures ensured that nearly all included participants contributed usable outcome data. |
|  | 3.4. Are the confounders accounted for in the design and analysis?  No  Although sociodemographic variables (e.g., age, gender, education) and comorbidity data were collected and included as features in machine learning models, the study did not explicitly control for potential confounding through stratification, regression adjustment, or matching. Thus, while these variables were available, they were not used to statistically account for confounding bias in a conventional sense. |
|  | 3.5 During the study period, is the intervention administered (or exposure occurred) as intended?  Yes  Participants were instructed to use the Mood Mirror app and wear the Mi Band 2 wristband continuously over a 12-week period. The study only included samples that met predefined data quality thresholds (e.g., valid physiological data duration, PHQ-9 completion). Data filtering and sample exclusion rules were rigorously applied, supporting that the passive sensing exposure occurred as intended. |

**Part I: Mixed Methods Appraisal Tool (MMAT), version 2018**

**27. “Sensor-Assisted Weighted Average Ensemble Model for Detecting Major Depressive Disorder” (Sensors, 2019**)

| **Category of study designs** | **Methodological quality criteria** | **Responses** | | | |
| --- | --- | --- | --- | --- | --- |
|  |  | Yes | No | Can’t tell | Comments |
| Screening questions (for all types) | S1. Are there clear research questions? | **V** |  |  |  |
|  | S2. Do the collected data allow to address the research questions? | **V** |  |  |  |
|  | *Further appraisal may not be feasible or appropriate when the answer is ‘No’ or ‘Can’t tell’ to one or both screening questions.* | | | | |
| 1. Qualitative | 1.1. Is the qualitative approach appropriate to answer the research question? |  |  |  |  |
|  | 1.2. Are the qualitative data collection methods adequate to address the research question? |  |  |  |  |
|  | 1.3. Are the findings adequately derived from the data? |  |  |  |  |
|  | 1.4. Is the interpretation of results sufficiently substantiated by data? |  |  |  |  |
|  | 1.5. Is there coherence between qualitative data sources, collection, analysis and interpretation? |  |  |  |  |
| 2. Quantitative randomized controlled trials | 2.1. Is randomization appropriately performed? |  |  |  |  |
|  | 2.2. Are the groups comparable at baseline? |  |  |  |  |
|  | 2.3. Are there complete outcome data? |  |  |  |  |
|  | 2.4. Are outcome assessors blinded to the intervention provided? |  |  |  |  |
|  | 2.5 Did the participants adhere to the assigned intervention? |  |  |  |  |
| 3. Quantitative non- randomized | 3.1. Are the participants representative of the target population? |  |  |  |  |
|  | 3.2. Are measurements appropriate regarding both the outcome and intervention (or exposure)? |  |  |  |  |
|  | 3.3. Are there complete outcome data? |  |  |  |  |
|  | 3.4. Are the confounders accounted for in the design and analysis? |  |  |  |  |
|  | 3.5. During the study period, is the intervention administered (or exposure occurred) as intended? |  |  |  |  |
| 4. Quantitative descriptive | 4.1. Is the sampling strategy relevant to address the research question? |  | **V** |  |  |
|  | 4.2. Is the sample representative of the target population? |  | **V** |  |  |
|  | 4.3. Are the measurements appropriate? | **V** |  |  |  |
|  | 4.4. Is the risk of nonresponse bias low? |  |  | **V** |  |
|  | 4.5. Is the statistical analysis appropriate to answer the research question? | **V** |  |  |  |
| 5. Mixed methods | 5.1. Is there an adequate rationale for using a mixed methods design to address the research question? |  |  |  |  |
|  | 5.2. Are the different components of the study effectively integrated to answer the research question? |  |  |  |  |
|  | 5.3. Are the outputs of the integration of qualitative and quantitative components adequately interpreted? |  |  |  |  |
|  | 5.4. Are divergences and inconsistencies between quantitative and qualitative results adequately addressed? |  |  |  |  |
|  | 5.5 Do the different components of the study adhere to the quality criteria of each tradition of the methods involved? |  |  |  |  |

**Part II: Explanation**

| **4. Quantitative descriptive studies** | **Methodological quality criteria** |
| --- | --- |
| Quantitative descriptive studies are “concerned with and designed only to describe the existing distribution of variables without much regard to causal relationships or other hypotheses” (Porta et al., 2014, p. 72). They are used to monitoring the population, planning, and generating hypothesis (Grimes and Schulz, 2002).  Common designs include the following single-group studies (this list is not exhaustive):  **Incidence or prevalence study without comparison group**  In a defined population at one particular time, what is happening in a population, e.g., frequencies of factors (importance of problems), is described (portrayed).  **Survey**  “Research method by which information is gathered by asking people questions on a specific topic and the data collection procedure is standardized and well defined.” (Bennett et al., 2011, p. 3).  **Case series**  A collection of individuals with similar characteristics are used to describe an outcome.  **Case report**  An individual or a group with a unique/unusual outcome is described in detail.  Key references: Critical Appraisal Skills Programme (2017); Draugalis et al. (2008) | 4.1. Is the sampling strategy relevant to address the research question? **No**.  Explanations  The paper lacks clear information about how participants were recruited or selected. There is no mention of randomization, stratification, or inclusion/exclusion criteria, which limits the generalizability of the results. |
|  | 4.2. Is the sample representative of the target population? **No**.  Explanations  Although age and gender information are presented, the target population is not clearly defined, and the sample may not adequately represent individuals with MDD in broader clinical or community settings. |
|  | 4.3. Are the measurements appropriate? **Yes.**  Explanations  The study employed validated clinical scales (HDRS) and wearable sensor data (heart rate, accelerometry), which are appropriate for assessing depression-related physiological and behavioral patterns. |
|  | 4.4. Is the risk of nonresponse bias low? **Can’t tell**  Explanations  The study excluded 50 participants with data loss but did not provide an analysis of the characteristics of nonrespondents or the potential bias this may introduce. |
|  | 4.5. Is the statistical analysis appropriate to answer the research question? **Yes**  Explanations  The study employed machine learning methods including ensemble models with appropriate performance metrics (AUC, precision, recall) and cross-validation, which are suitable for predictive modeling. |

**Part I: Mixed Methods Appraisal Tool (MMAT), version 2018**

**28.“Effectiveness of a Smartphone App With a Wearable Activity Tracker in Preventing the Recurrence of Mood Disorders” (JMIR Mental Health, 2020)**

| **Category of study designs** | **Methodological quality criteria** | **Responses** | | | |
| --- | --- | --- | --- | --- | --- |
|  |  | Yes | No | Can’t tell | Comments |
| Screening questions (for all types) | S1. Are there clear research questions? | V |  |  |  |
|  | S2. Do the collected data allow to address the research questions? | V |  |  |  |
|  | *Further appraisal may not be feasible or appropriate when the answer is ‘No’ or ‘Can’t tell’ to one or both screening questions.* | | | | |
| 1. Qualitative | 1.1. Is the qualitative approach appropriate to answer the research question? |  |  |  |  |
|  | 1.2. Are the qualitative data collection methods adequate to address the research question? |  |  |  |  |
|  | 1.3. Are the findings adequately derived from the data? |  |  |  |  |
|  | 1.4. Is the interpretation of results sufficiently substantiated by data? |  |  |  |  |
|  | 1.5. Is there coherence between qualitative data sources, collection, analysis and interpretation? |  |  |  |  |
| 2. Quantitative randomized controlled trials | 2.1. Is randomization appropriately performed? |  |  |  |  |
|  | 2.2. Are the groups comparable at baseline? |  |  |  |  |
|  | 2.3. Are there complete outcome data? |  |  |  |  |
|  | 2.4. Are outcome assessors blinded to the intervention provided? |  |  |  |  |
|  | 2.5 Did the participants adhere to the assigned intervention? |  |  |  |  |
| 3. Quantitative non- randomized | 3.1. Are the participants representative of the target population? |  | V |  |  |
|  | 3.2. Are measurements appropriate regarding both the outcome and intervention (or exposure)? | V |  |  |  |
|  | 3.3. Are there complete outcome data? | V |  |  |  |
|  | 3.4. Are the confounders accounted for in the design and analysis? |  |  | V |  |
|  | 3.5. During the study period, is the intervention administered (or exposure occurred) as intended? | V |  |  |  |
| 4. Quantitative descriptive | 4.1. Is the sampling strategy relevant to address the research question? |  |  |  |  |
|  | 4.2. Is the sample representative of the target population? |  |  |  |  |
|  | 4.3. Are the measurements appropriate? |  |  |  |  |
|  | 4.4. Is the risk of nonresponse bias low? |  |  |  |  |
|  | 4.5. Is the statistical analysis appropriate to answer the research question? |  |  |  |  |
| 5. Mixed methods | 5.1. Is there an adequate rationale for using a mixed methods design to address the research question? |  |  |  |  |
|  | 5.2. Are the different components of the study effectively integrated to answer the research question? |  |  |  |  |
|  | 5.3. Are the outputs of the integration of qualitative and quantitative components adequately interpreted? |  |  |  |  |
|  | 5.4. Are divergences and inconsistencies between quantitative and qualitative results adequately addressed? |  |  |  |  |
|  | 5.5. Do the different components of the study adhere to the quality criteria of each tradition of the methods involved? |  |  |  |  |

**Part II: Explanation**

| \| **3. Quantitative non-randomized studies** \| **Methodological quality criteria** \| \| --- \| --- \| \| Non-randomized studies are defined as any quantitative studies estimating the effectiveness of an intervention or studying other exposures that do not use randomization to allocate units to comparison groups (Higgins and Green, 2008).  Common designs include (this list is not exhaustive):  **Non-randomized controlled trials**  The intervention is assigned by researchers, but there is no randomization, e.g., a pseudo-randomization. A non- random method of allocation is not reliable in producing alone similar groups.  **Cohort study**  Subsets of a defined population are assessed as exposed, not exposed, or exposed at different degrees to factors of interest. Participants are followed over time to determine if an outcome occurs (prospective longitudinal).  **Case-control study**  Cases, e.g., patients, associated with a certain outcome are selected, alongside a corresponding group of controls.  Data is collected on whether cases and controls were exposed to the factor under study (retrospective).  **Cross-sectional analytic study**  At one particular time, the relationship between health- related characteristics (outcome) and other factors (intervention/exposure) is examined. E.g., the frequency of outcomes is compared in different population subgroups according to the presence/absence (or level) of the intervention/exposure. \| 3.1. Are the participants representative of the target population? **No.**  Explanations  The intervention and control groups were not matched at baseline, and significant demographic and clinical differences were reported, potentially confounding the results. \| \| 3.2. Are measurements appropriate regarding both the outcome and intervention (or exposure)? **Yes.**  Explanations  The outcome measures (number and duration of mood episodes) were relevant and collected systematically using validated tools and digital logs (e.g., eMoodChart, Fitbit data). \| \| 3.3. Are there complete outcome data? **Yes**.  Explanations  The study defined and excluded participants with insufficient data (e.g., Fitbit adherence <60%), and final analysis included only those with complete data over the intervention period. \| \| 3.4. Are the confounders accounted for in the design and analysis? **Can’t tell.**  Explanations  While some covariates may have been implicitly controlled (e.g., diagnostic categories), there is no clear multivariate adjustment for baseline group differences. \| \| 3.5 During the study period, is the intervention administered (or exposure occurred) as intended? **Yes.**  Explanations  The intervention (CRM app and Fitbit) was used as designed, with real-time feedback and adherence monitoring, ensuring fidelity of implementation. \| |
| --- | --- | --- | --- | --- | --- | --- | --- | --- |

**29. “Mood Prediction of Patients With Mood Disorders by Machine Learning Using Passive Digital Phenotypes Based on the Circadian Rhythm” (JMIR Mental Health, 2019)**

**Part I: Mixed Methods Appraisal Tool (MMAT), version 2018**

| **Category of study designs** | **Methodological quality criteria** | **Responses** | | | |
| --- | --- | --- | --- | --- | --- |
|  |  | Yes | No | Can’t tell | Comments |
| Screening questions (for all types) | S1. Are there clear research questions? | V |  |  |  |
|  | S2. Do the collected data allow to address the research questions? | V |  |  |  |
|  | *Further appraisal may not be feasible or appropriate when the answer is ‘No’ or ‘Can’t tell’ to one or both screening questions.* | | | | |
| 1. Qualitative | 1.1. Is the qualitative approach appropriate to answer the research question? |  |  |  |  |
|  | 1.2. Are the qualitative data collection methods adequate to address the research question? |  |  |  |  |
|  | 1.3. Are the findings adequately derived from the data? |  |  |  |  |
|  | 1.4. Is the interpretation of results sufficiently substantiated by data? |  |  |  |  |
|  | 1.5. Is there coherence between qualitative data sources, collection, analysis and interpretation? |  |  |  |  |
| 2. Quantitative randomized controlled trials | 2.1. Is randomization appropriately performed? |  |  |  |  |
|  | 2.2. Are the groups comparable at baseline? |  |  |  |  |
|  | 2.3. Are there complete outcome data? |  |  |  |  |
|  | 2.4. Are outcome assessors blinded to the intervention provided? |  |  |  |  |
|  | 2.5 Did the participants adhere to the assigned intervention? |  |  |  |  |
| 3. Quantitative non- randomized | 3.1. Are the participants representative of the target population? |  |  |  |  |
|  | 3.2. Are measurements appropriate regarding both the outcome and intervention (or exposure)? |  |  |  |  |
|  | 3.3. Are there complete outcome data? |  |  |  |  |
|  | 3.4. Are the confounders accounted for in the design and analysis? |  |  |  |  |
|  | 3.5. During the study period, is the intervention administered (or exposure occurred) as intended? |  |  |  |  |
| 4. Quantitative descriptive | 4.1. Is the sampling strategy relevant to address the research question? | V |  |  |  |
|  | 4.2. Is the sample representative of the target population? |  |  | V |  |
|  | 4.3. Are the measurements appropriate? | V |  |  |  |
|  | 4.4. Is the risk of nonresponse bias low? |  |  | V |  |
|  | 4.5. Is the statistical analysis appropriate to answer the research question? | V |  |  |  |
| 5. Mixed methods | 5.1. Is there an adequate rationale for using a mixed methods design to address the research question? |  |  |  |  |
|  | 5.2. Are the different components of the study effectively integrated to answer the research question? |  |  |  |  |
|  | 5.3. Are the outputs of the integration of qualitative and quantitative components adequately interpreted? |  |  |  |  |
|  | 5.4. Are divergences and inconsistencies between quantitative and qualitative results adequately addressed? |  |  |  |  |
|  | 5.5. Do the different components of the study adhere to the quality criteria of each tradition of the methods involved? |  |  |  |  |

| **4. Quantitative descriptive studies** | **Methodological quality criteria** |
| --- | --- |
| Quantitative descriptive studies are “concerned with and designed only to describe the existing distribution of variables without much regard to causal relationships or other hypotheses” (Porta et al., 2014, p. 72). They are used to monitoring the population, planning, and generating hypothesis (Grimes and Schulz, 2002).  Common designs include the following single-group studies (this list is not exhaustive):  **Incidence or prevalence study without comparison group**  In a defined population at one particular time, what is happening in a population, e.g., frequencies of factors (importance of problems), is described (portrayed).  **Survey**  “Research method by which information is gathered by asking people questions on a specific topic and the data collection procedure is standardized and well defined.” (Bennett et al., 2011, p. 3).  **Case series**  A collection of individuals with similar characteristics are used to describe an outcome.  **Case report**  An individual or a group with a unique/unusual outcome is described in detail.  Key references: Critical Appraisal Skills Programme (2017); Draugalis et al. (2008) | 4.1. Is the sampling strategy relevant to address the research question? **Yes.**  Explanations  Participants were recruited from a well-defined clinical cohort (MDCRC), and the inclusion criteria were clearly outlined, enhancing relevance. |
|  | 4.2. Is the sample representative of the target population? **Can’t tell.**  Explanations  The sample size (n=55) is relatively small, and while clinical characteristics are described, no formal assessment of representativeness is provided. |
|  | 4.3. Are the measurements appropriate? **Yes.**  Explanations  Data were collected via validated tools (Fitbit, eMoodChart), and the derived digital biomarkers were methodologically sound and relevant to mood disorder prediction. |
|  | 4.4. Is the risk of nonresponse bias low? **Can’t tell.**  Explanations  While only complete data from 19,299 minutes were used, nonadherence or participant exclusion due to missing data is not fully explained.The nonresponse bias is might not be pertinent for case series and case report. This criterion could be adapted. For instance, complete data on the cases might be important to consider in these designs. |
|  | 4.5. Is the statistical analysis appropriate to answer the research question? **Yes.**  Explanations  Advanced machine learning techniques (XGBoost, LOOCV, permutation testing) were employed, supporting the validity of predictive findings.. |

**Part II: Explanation**

**30. "Digital biomarkers of mood disorders and symptom change" (npj Digital Medicine, 2019**)

**Part I: Mixed Methods Appraisal Tool (MMAT), version 2018**

| **Category of study designs** | **Methodological quality criteria** | **Responses** | | | |
| --- | --- | --- | --- | --- | --- |
|  |  | Yes | No | Can’t tell | Comments |
| Screening questions (for all types) | S1. Are there clear research questions? | V |  |  |  |
|  | S2. Do the collected data allow to address the research questions? | V |  |  |  |
|  | *Further appraisal may not be feasible or appropriate when the answer is ‘No’ or ‘Can’t tell’ to one or both screening questions.* | | | | |
| 1. Qualitative | 1.1. Is the qualitative approach appropriate to answer the research question? |  |  |  |  |
|  | 1.2. Are the qualitative data collection methods adequate to address the research question? |  |  |  |  |
|  | 1.3. Are the findings adequately derived from the data? |  |  |  |  |
|  | 1.4. Is the interpretation of results sufficiently substantiated by data? |  |  |  |  |
|  | 1.5. Is there coherence between qualitative data sources, collection, analysis and interpretation? |  |  |  |  |
| 2. Quantitative randomized controlled trials | 2.1. Is randomization appropriately performed? |  |  |  |  |
|  | 2.2. Are the groups comparable at baseline? |  |  |  |  |
|  | 2.3. Are there complete outcome data? |  |  |  |  |
|  | 2.4. Are outcome assessors blinded to the intervention provided? |  |  |  |  |
|  | 2.5 Did the participants adhere to the assigned intervention? |  |  |  |  |
| 3. Quantitative non- randomized | 3.1. Are the participants representative of the target population? | V |  |  |  |
|  | 3.2. Are measurements appropriate regarding both the outcome and intervention (or exposure)? | V |  |  |  |
|  | 3.3. Are there complete outcome data? | V |  |  |  |
|  | 3.4. Are the confounders accounted for in the design and analysis? |  | V |  |  |
|  | 3.5. During the study period, is the intervention administered (or exposure occurred) as intended? | V |  |  |  |
| 4. Quantitative descriptive | 4.1. Is the sampling strategy relevant to address the research question? |  |  |  |  |
|  | 4.2. Is the sample representative of the target population? |  |  |  |  |
|  | 4.3. Are the measurements appropriate? |  |  |  |  |
|  | 4.4. Is the risk of nonresponse bias low? |  |  |  |  |
|  | 4.5. Is the statistical analysis appropriate to answer the research question? | V |  |  |  |
| 5. Mixed methods | 5.1. Is there an adequate rationale for using a mixed methods design to address the research question? |  |  |  |  |
|  | 5.2. Are the different components of the study effectively integrated to answer the research question? |  |  |  |  |
|  | 5.3. Are the outputs of the integration of qualitative and quantitative components adequately interpreted? |  |  |  |  |
|  | 5.4. Are divergences and inconsistencies between quantitative and qualitative results adequately addressed? |  |  |  |  |
|  | 5.5. Do the different components of the study adhere to the quality criteria of each tradition of the methods involved? |  |  |  |  |

| \| **3. Quantitative non-randomized studies** \| **Methodological quality criteria** \| \| --- \| --- \| \| Non-randomized studies are defined as any quantitative studies estimating the effectiveness of an intervention or studying other exposures that do not use randomization to allocate units to comparison groups (Higgins and Green, 2008).  Common designs include (this list is not exhaustive):  **Non-randomized controlled trials**  The intervention is assigned by researchers, but there is no randomization, e.g., a pseudo-randomization. A non- random method of allocation is not reliable in producing alone similar groups.  **Cohort study**  Subsets of a defined population are assessed as exposed, not exposed, or exposed at different degrees to factors of interest. Participants are followed over time to determine if an outcome occurs (prospective longitudinal).  **Case-control study**  Cases, e.g., patients, associated with a certain outcome are selected, alongside a corresponding group of controls.  Data is collected on whether cases and controls were exposed to the factor under study (retrospective).  **Cross-sectional analytic study**  At one particular time, the relationship between health- related characteristics (outcome) and other factors (intervention/exposure) is examined. E.g., the frequency of outcomes is compared in different population subgroups according to the presence/absence (or level) of the intervention/exposure. \| 3.1. Are the participants representative of the target population? **Yes**  Explanations  Participants were drawn from a clinical population with MDD and BD and matched with healthy controls; demographic characteristics (e.g., age, sex) were described, enhancing population relevance.. \| \| 3.2. Are measurements appropriate regarding both the outcome and intervention (or exposure)? **Yes**  Explanations  The outcome (diagnostic group, MADRS score change) and exposure (actigraphy data) were well-aligned with the research objectives and assessed using validated tools and methods. \| \| 3.3. Are there complete outcome data? **Yes**  Explanations  All participants provided the minimum required actigraphy data (19,299 minutes), and no missing data were present in the final feature set. \| \| 3.4. Are the confounders accounted for in the design and analysis? **No**  Explanations  The study did not control for confounders such as medication use or inpatient status in the predictive modeling, which may have influenced results. \| \| 3.5 During the study period, is the intervention administered (or exposure occurred) as intended? **Yes**  Explanations  Data collection through actigraph devices was implemented as planned, and adherence was monitored; all subjects wore devices for the full duration. \| |
| --- | --- | --- | --- | --- | --- | --- | --- | --- |

**Part II: Explanation**

**Part I: Mixed Methods Appraisal Tool (MMAT), version 2018**

**31. depression with multimodal wristband-type wearable device: screening and assessing patient severity utilizing machine-learning. Tazawa, (2020).**

| **Category of study designs** | **Methodological quality criteria** | **Responses** | | | |
| --- | --- | --- | --- | --- | --- |
|  |  | Yes | No | Can’t tell | Comments |
| Screening questions (for all types) | S1. Are there clear research questions? | O |  |  |  |
|  | S2. Do the collected data allow to address the research questions? | o |  |  |  |
|  | *Further appraisal may not be feasible or appropriate when the answer is ‘No’ or ‘Can’t tell’ to one or both screening questions.* | | | | |
| 1. Qualitative | 1.1. Is the qualitative approach appropriate to answer the research question? |  |  |  |  |
|  | 1.2. Are the qualitative data collection methods adequate to address the research question? |  |  |  |  |
|  | 1.3. Are the findings adequately derived from the data? |  |  |  |  |
|  | 1.4. Is the interpretation of results sufficiently substantiated by data? |  |  |  |  |
|  | 1.5. Is there coherence between qualitative data sources, collection, analysis and interpretation? |  |  |  |  |
| 2. Quantitative randomized controlled trials | 2.1. Is randomization appropriately performed? |  |  |  |  |
|  | 2.2. Are the groups comparable at baseline? |  |  |  |  |
|  | 2.3. Are there complete outcome data? |  |  |  |  |
|  | 2.4. Are outcome assessors blinded to the intervention provided? |  |  |  |  |
|  | 2.5 Did the participants adhere to the assigned intervention? |  |  |  |  |
| 3. Quantitative non- randomized | 3.1. Are the participants representative of the target population? |  |  | O |  |
|  | 3.2. Are measurements appropriate regarding both the outcome and intervention (or exposure)? | O |  |  |  |
|  | 3.3. Are there complete outcome data? | O |  |  |  |
|  | 3.4. Are the confounders accounted for in the design and analysis? | O |  |  |  |
|  | 3.5. During the study period, is the intervention administered (or exposure occurred) as intended? | o |  |  |  |
| 4. Quantitative descriptive | 4.1. Is the sampling strategy relevant to address the research question? |  |  |  |  |
|  | 4.2. Is the sample representative of the target population? |  |  |  |  |
|  | 4.3. Are the measurements appropriate? |  |  |  |  |
|  | 4.4. Is the risk of nonresponse bias low? |  |  |  |  |
|  | 4.5. Is the statistical analysis appropriate to answer the research question? |  |  |  |  |
| 5. Mixed methods | 5.1. Is there an adequate rationale for using a mixed methods design to address the research question? |  |  |  |  |
|  | 5.2. Are the different components of the study effectively integrated to answer the research question? |  |  |  |  |
|  | 5.3. Are the outputs of the integration of qualitative and quantitative components adequately interpreted? |  |  |  |  |
|  | 5.4. Are divergences and inconsistencies between quantitative and qualitative results adequately addressed? |  |  |  |  |
|  | 5.5. Do the different components of the study adhere to the quality criteria of each tradition of the methods involved? |  |  |  |  |

**Part II: Explanation**

| **3. Quantitative non-randomized studies** | **Methodological quality criteria** |
| --- | --- |
| Non-randomized studies are defined as any quantitative studies estimating the effectiveness of an intervention or studying other exposures that do not use randomization to allocate units to comparison groups (Higgins and Green, 2008).  Common designs include (this list is not exhaustive):  **Non-randomized controlled trials**  The intervention is assigned by researchers, but there is no randomization, e.g., a pseudo-randomization. A non- random method of allocation is not reliable in producing alone similar groups.  **Cohort study**  Subsets of a defined population are assessed as exposed, not exposed, or exposed at different degrees to factors of interest. Participants are followed over time to determine if an outcome occurs (prospective longitudinal).  **Case-control study**  Cases, e.g., patients, associated with a certain outcome are selected, alongside a corresponding group of controls.  Data is collected on whether cases and controls were exposed to the factor under study (retrospective).  **Cross-sectional analytic study**  At one particular time, the relationship between health- related characteristics (outcome) and other factors (intervention/exposure) is examined. E.g., the frequency of outcomes is compared in different population subgroups according to the presence/absence (or level) of the intervention/exposure.  Key references for non-randomized studies: Higgins and Green (2008); Porta et al. (2014); Sterne et al. (2016); Wells et al. (2000) | 3.1. Are the participants representative of the target population?  Can’t tell.  The study included outpatients and inpatients with MDD or BD from 10 hospitals in Japan, as well as healthy controls confirmed via MINI.  Although inclusion/exclusion criteria and demographics were clearly stated, the sampling method (e.g., voluntary participants from psychiatric clinics) may not fully represent the broader MDD population. |
|  | 3.2. Are measurements appropriate regarding both the outcome and intervention (or exposure)?  **Yes**  Depression severity was assessed using validated clinical scales: HAMD-17, MADRS, BDI-II, and PSQI.  Physiological data (heart rate, sleep, physical activity, temperature, UV) were collected using the Silmee W20 wristband, with justification and technical details provided.  Measurement validity and preprocessing procedures were described. |
|  | 3.3. Are there complete outcome data?  **Yes.**  All participants provided at least one valid data sample, and there is no mention of participant withdrawal or dropout throughout the study. A total of 228 samples were used for the 3-day model and 236 for the 7-day model, with each subject contributing an average of 4.2 assessment sessions. The dataset inclusion criteria (e.g., ≥20 hours/day of wearable data) ensured data quality, and the consistent number of samples relative to the recruited population suggests that loss to follow-up was negligible. Therefore, the outcome data can be considered sufficiently complete according to MMAT standards and published thresholds (e.g., ≥80%). |
|  | 3.4. Are the confounders accounted for in the design and analysis?  **Yes.**  The authors identified a significant age difference between the symptomatic and asymptomatic groups. To address this, they performed multiple linear regression analyses with age included as a covariate, and also conducted subgroup analyses on age-matched samples. These steps demonstrate appropriate statistical adjustment for confounding, increasing confidence in the validity of the group comparisons. |
|  | 3.5 During the study period, is the intervention administered (or exposure occurred) as intended?  **Yes.**  The exposure—wearing the Silmee W20 wristband—was carried out as intended throughout the study period. The authors applied clear inclusion criteria for data quality: only data with ≥20 hours of valid physiological recording per day were used, and heart rate values had to fall within a physiological range (30–200 bpm). Segments with insufficient sensor readings (e.g., fewer than 7 HR measurements per hour) were excluded. These preprocessing steps ensured that exposure to the passive sensing protocol occurred consistently and as intended across participants. |

**References**

1. Hong QN, Pluye P, Fàbregues S, Bartlett G, Boardman F, Cargo M, Dagenais P, Gagnon M-P, Griffiths F, Nicolau B, O’Cathain A, Rousseau M-C, Vedel I. Mixed Methods Appraisal Tool (MMAT), version 2018. Registration of Copyright (#1148552), Canadian Intellectual Property Office, Industry Canada. (chrome-extension://efaidnbmnnnibpcajpcglclefindmkaj/http://mixedmethodsappraisaltoolpublic.pbworks.com/w/file/fetch/127916259/MMAT_2018_criteria-manual_2018-08-01_ENG.pdf)
