# Supplementary material for: Key Features of Digital Phenotyping for Monitoring Mental Disorders: Systematic Review
Source: J Med Internet Res. 2025 Nov 5;27:e77331. doi: 10.2196/77331 (PMC12588392; doi:10.2196/77331)
Supplement: Multimedia Appendix 2 [file jmir-v27-e77331-s002.docx]

# Quality Criteria Checklist: Primary Research

# Exploring Digital Biomarkers of Illness Activity in Mood Episodes: Hypotheses Generating and Model Development Study

Quality Criteria Checklist: Primary Research

| **RELEVANCE QUESTIONS** | | | | |
| --- | --- | --- | --- | --- |
| 1. Would implementing the studied intervention or procedure (if found successful) result in improved outcomes for the patients/clients/population group? (NA for some Epi studies) | Yes | No | Unclear | **N/A** |
| 2. Did the authors study an outcome (dependent variable) or topic that the patients/clients/population group would care about? | **Yes** | No | Unclear | N/A |
| 3. Is the focus of the intervention or procedure (independent variable) or topic of study a common issue of concern to mental health practice? | **Yes** | No | Unclear | N/A |
| 4. Is the intervention or procedure feasible? (NA for some epidemiological studies) | **Yes** | No | Unclear | N/A |
| ***If the answers to all of the above relevance questions are “Yes,” the report is eligible for designation with a plus (+) on the Evidence Quality Worksheet, depending on answers to the following validity questions.*** | | | | |
| **VALIDITY QUESTIONS** | | | | |
| 1. **Was the research question clearly stated?**    1. Was the specific intervention(s) or procedure (independent variable(s)) identified?    2. Was the outcome(s) (dependent variable(s)) clearly indicated?    3. Were the target population and setting specified? | **Yes** | No | **Unclear** | N/A |
| 1. **Was the selection of study subjects/patients free from bias?**    1. Were inclusion/exclusion criteria specified (e.g., risk, point in disease progression, diagnostic or prognosis criteria), and with sufficient detail and without omitting criteria critical to the study?    2. Were criteria applied equally to all study groups?    3. Were health, demographics, and other characteristics of subjects described?    4. Were the subjects/patients a representative sample of the relevant population? | Yes | No | **Unclear** | N/A |
| 1. **Were study groups comparable?**    1. Was the method of assigning subjects/patients to groups described and unbiased? (Method of randomization identified if RCT)    2. Were distribution of disease status, prognostic factors, and other factors (e.g., demographics) similar across study groups at baseline?    3. Were concurrent controls used? (Concurrent preferred over historical controls.)    4. If cohort study or cross-sectional study, were groups comparable on important confounding factors and/or were preexisting differences accounted for by using appropriate adjustments in statistical analysis?    5. If case control study, were potential confounding factors comparable for cases and controls? (If case series or trial with subjects serving as own control, this criterion is not applicable. Criterion may not be applicable in some cross-sectional studies.)    6. If diagnostic test, was there an independent blind comparison with an appropriate reference standard (e.g., “gold standard”)? | Yes | No | Unclear | **N/A** |
| 1. **Was method of handling withdrawals described?**    1. Were follow up methods described and the same for all groups?    2. Was the number, characteristics of withdrawals (i.e., dropouts, lost to follow up, attrition rate) and/or response rate (cross-sectional studies) described for each group? (Follow up goal for a strong study is 80%.)    3. Were all enrolled subjects/patients (in the original sample) accounted for?    4. Were reasons for withdrawals similar across groups?    5. If diagnostic test, was decision to perform reference test not dependent on results of test under study? | **Yes** | No | Unclear | N/A |
| 1. **Was blinding used to prevent introduction of bias?**    1. In intervention study, were subjects, clinicians/practitioners, and investigators blinded to treatment group, as appropriate? | Yes | No | Unclear | **N/A** |

| - 1. Were data collectors blinded for outcomes assessment? (If outcome is measured using an objective test, such as a lab value, this criterion is assumed to be met.)   2. In cohort study or cross-sectional study, were measurements of outcomes and risk factors blinded?   3. In case control study, was case definition explicit and case ascertainment not influenced by exposure status?   4. In diagnostic study, were test results blinded to patient history and other test results? |  | | | |
| --- | --- | --- | --- | --- |
| 1. **Were intervention/therapeutic regimens/exposure factor or procedure and any comparison(s) described in detail? Were intervening factors described?**    1. In RCT or other intervention trial, were protocols described for all regimens studied?    2. In observational study, were interventions, study settings, and clinicians/provider described?    3. Was the intensity and duration of the intervention or exposure factor sufficient to produce a meaningful effect?    4. Was the amount of exposure and, if relevant, subject/patient compliance measured?    5. Were co-interventions (e.g., ancillary treatments, other therapies) described?    6. Were extra or unplanned treatments described?    7. Was the information for 6.4, 6.5, and 6.6 assessed the same way for all groups?    8. In diagnostic study, were details of test administration and replication sufficient? | Yes | No | **Unclear** | N/A |
| 1. **Were outcomes clearly defined and the measurements valid and reliable?**    1. Were primary and secondary endpoints described and relevant to the question?    2. Were mental health measures appropriate to question and outcomes of concern?    3. Was the period of follow-up long enough for important outcome(s) to occur?    4. Were the observations and measurements based on standard, valid, and reliable data collection instruments/tests/procedures?    5. Was the measurement of effect at an appropriate level of precision?    6. Were other factors accounted for (measured) that could affect outcomes?    7. Were the measurements conducted consistently across groups? | **Yes** | No | Unclear | N/A |
| 1. **Was the statistical analysis appropriate for the study design and type of outcome indicators?**    1. Were statistical analyses adequately described the results reported appropriately?    2. Were correct statistical tests used and assumptions of test not violated?    3. Were statistics reported with levels of significance and/or confidence intervals?    4. Was “intent to treat” analysis of outcomes done (and as appropriate, was there an analysis of outcomes for those maximally exposed or a dose-response analysis)?    5. Were adequate adjustments made for effects of confounding factors that might have affected the outcomes (e.g., multivariate analyses)?    6. Was clinical significance as well as statistical significance reported?    7. If negative findings, was a power calculation reported to address type 2 error? | **Yes** | No | Unclear | N/A |
| 1. **Are conclusions supported by results with biases and limitations taken into consideration?**    1. Is there a discussion of findings?    2. Are biases and study limitations identified and discussed? | **Yes** | No | Unclear | N/A |
| 1. **Is bias due to study’s funding or sponsorship unlikely?**    1. Were sources of funding and investigators’ affiliations described?    2. Was there no apparent conflict of interest? | **Yes** | No | Unclear | N/A |
| **MINUS/NEGATIVE (-)**  *If most (six or more) of the answers to the above validity questions are “No,” the report should be designated with a minus (-) symbol on the Evidence Worksheet.* | | | | |
| **NEUTRAL (**∅**)**  *If the answers to validity criteria questions 2, 3, 6, and 7 do not indicate that the study is exceptionally strong, the report should be designated with a neutral* (∅) *symbol on the Evidence Worksheet.* | | | | |
| **PLUS/POSITIVE (+)**  *If most of the answers to the above validity questions are “Yes” (including criteria 2, 3, 6, 7 and at least one additional “Yes”), the report should be designated with a plus symbol (+) on the Evidence Worksheet.* | | | | |

# Final score: NEUTRAL **(∅)**

# Quality Criteria Checklist: Primary Research

1. **Sequence Modeling of Passive Sensing Data for Treatment Response Prediction in Major Depressive Disorder**

Quality Criteria Checklist: Primary Research

| **RELEVANCE QUESTIONS** | | | | |
| --- | --- | --- | --- | --- |
| 1. Would implementing the studied intervention or procedure (if found successful) result in improved outcomes for the patients/clients/population group? (NA for some Epi studies) | **Yes** | No | Unclear | N/A |
| 2. Did the authors study an outcome (dependent variable) or topic that the patients/clients/population group would care about? | **Yes** | No | Unclear | N/A |
| 3. Is the focus of the intervention or procedure (independent variable) or topic of study a common issue of concern to mental health practice? | **Yes** | No | Unclear | N/A |
| 4. Is the intervention or procedure feasible? (NA for some epidemiological studies) | **Yes** | No | Unclear | N/A |
| ***If the answers to all of the above relevance questions are “Yes,” the report is eligible for designation with a plus (+) on the Evidence Quality Worksheet, depending on answers to the following validity questions.*** | | | | |
| **VALIDITY QUESTIONS** | | | | |
| 1. **Was the research question clearly stated?**    1. Was the specific intervention(s) or procedure (independent variable(s)) identified?    2. Was the outcome(s) (dependent variable(s)) clearly indicated?    3. Were the target population and setting specified? | **Yes** | No | Unclear | N/A |
| 1. **Was the selection of study subjects/patients free from bias?**    1. Were inclusion/exclusion criteria specified (e.g., risk, point in disease progression, diagnostic or prognosis criteria), and with sufficient detail and without omitting criteria critical to the study?    2. Were criteria applied equally to all study groups?    3. Were health, demographics, and other characteristics of subjects described?    4. Were the subjects/patients a representative sample of the relevant population? | **Yes** | No | Unclear | N/A |
| 1. **Were study groups comparable?**    1. Was the method of assigning subjects/patients to groups described and unbiased? (Method of randomization identified if RCT)    2. Were distribution of disease status, prognostic factors, and other factors (e.g., demographics) similar across study groups at baseline?    3. Were concurrent controls used? (Concurrent preferred over historical controls.)    4. If cohort study or cross-sectional study, were groups comparable on important confounding factors and/or were preexisting differences accounted for by using appropriate adjustments in statistical analysis?    5. If case control study, were potential confounding factors comparable for cases and controls? (If case series or trial with subjects serving as own control, this criterion is not applicable. Criterion may not be applicable in some cross-sectional studies.)    6. If diagnostic test, was there an independent blind comparison with an appropriate reference standard (e.g., “gold standard”)? | Yes | **No** | Unclear | N/A |
| 1. **Was method of handling withdrawals described?**    1. Were follow up methods described and the same for all groups?    2. Was the number, characteristics of withdrawals (i.e., dropouts, lost to follow up, attrition rate) and/or response rate (cross-sectional studies) described for each group? (Follow up goal for a strong study is 80%.)    3. Were all enrolled subjects/patients (in the original sample) accounted for?    4. Were reasons for withdrawals similar across groups?    5. If diagnostic test, was decision to perform reference test not dependent on results of test under study? | **Yes** | No | Unclear | N/A |
| 1. **Was blinding used to prevent introduction of bias?**    1. In intervention study, were subjects, clinicians/practitioners, and investigators blinded to treatment group, as appropriate? | Yes | No | Unclear | **N/A** |

| - 1. Were data collectors blinded for outcomes assessment? (If outcome is measured using an objective test, such as a lab value, this criterion is assumed to be met.)   2. In cohort study or cross-sectional study, were measurements of outcomes and risk factors blinded?   3. In case control study, was case definition explicit and case ascertainment not influenced by exposure status?   4. In diagnostic study, were test results blinded to patient history and other test results? |  | | | |
| --- | --- | --- | --- | --- |
| 1. **Were intervention/therapeutic regimens/exposure factor or procedure and any comparison(s) described in detail? Were intervening factors described?**    1. In RCT or other intervention trial, were protocols described for all regimens studied?    2. n observational study, were interventions, study settings, and clinicians/provider described?    3. Was the intensity and duration of the intervention or exposure factor sufficient to produce a meaningful effect?    4. Was the amount of exposure and, if relevant, subject/patient compliance measured?    5. Were co-interventions (e.g., ancillary treatments, other therapies) described?    6. Were extra or unplanned treatments described?    7. Was the information for 6.4, 6.5, and 6.6 assessed the same way for all groups?    8. In diagnostic study, were details of test administration and replication sufficient? | **Yes** | No | Unclear | N/A |
| 1. **Were outcomes clearly defined and the measurements valid and reliable?**    1. Were primary and secondary endpoints described and relevant to the question?    2. Were mental health measures appropriate to question and outcomes of concern?    3. Was the period of follow-up long enough for important outcome(s) to occur?    4. Were the observations and measurements based on standard, valid, and reliable data collection instruments/tests/procedures?    5. Was the measurement of effect at an appropriate level of precision?    6. Were other factors accounted for (measured) that could affect outcomes?    7. Were the measurements conducted consistently across groups? | **Yes** | No | Unclear | N/A |
| 1. **Was the statistical analysis appropriate for the study design and type of outcome indicators?**    1. Were statistical analyses adequately described the results reported appropriately?    2. Were correct statistical tests used and assumptions of test not violated?    3. Were statistics reported with levels of significance and/or confidence intervals?    4. Was “intent to treat” analysis of outcomes done (and as appropriate, was there an analysis of outcomes for those maximally exposed or a dose-response analysis)?    5. Were adequate adjustments made for effects of confounding factors that might have affected the outcomes (e.g., multivariate analyses)?    6. Was clinical significance as well as statistical significance reported?    7. If negative findings, was a power calculation reported to address type 2 error? | **Yes** | No | Unclear | N/A |
| 1. **Are conclusions supported by results with biases and limitations taken into consideration?**    1. Is there a discussion of findings?    2. Are biases and study limitations identified and discussed? | **Yes** | No | Unclear | N/A |
| 1. **Is bias due to study’s funding or sponsorship unlikely?**    1. Were sources of funding and investigators’ affiliations described?    2. Was there no apparent conflict of interest? | **Yes** | No | Unclear | N/A |
| **MINUS/NEGATIVE (-)**  *If most (six or more) of the answers to the above validity questions are “No,” the report should be designated with a minus (-) symbol on the Evidence Worksheet.* | | | | |
| **NEUTRAL (**∅**)**  *If the answers to validity criteria questions 2, 3, 6, and 7 do not indicate that the study is exceptionally strong, the report should be designated with a neutral* (∅) *symbol on the Evidence Worksheet.* | | | | |
| **PLUS/POSITIVE (+)**  *If most of the answers to the above validity questions are “Yes” (including criteria 2, 3, 6, 7 and at least one additional “Yes”), the report should be designated with a plus symbol (+) on the Evidence Worksheet.* | | | | |

# Final score: Positive (+)

# Quality Criteria Checklist: Primary Research

**3. Using Digital Phenotyping to Accurately Detect Depression Severity**

Quality Criteria Checklist: Primary Research

| **RELEVANCE QUESTIONS** | | | | |
| --- | --- | --- | --- | --- |
| 1. Would implementing the studied intervention or procedure (if found successful) result in improved outcomes for the patients/clients/population group? (NA for some Epi studies) | **Yes** | No | Unclear | N/A |
| 2. Did the authors study an outcome (dependent variable) or topic that the patients/clients/population group would care about? | **Yes** | No | Unclear | N/A |
| 3. Is the focus of the intervention or procedure (independent variable) or topic of study a common issue of concern to mental health practice? | **Yes** | No | Unclear | N/A |
| 4. Is the intervention or procedure feasible? (NA for some epidemiological studies) | **Yes** | No | Unclear | N/A |
| ***If the answers to all of the above relevance questions are “Yes,” the report is eligible for designation with a plus (+) on the Evidence Quality Worksheet, depending on answers to the following validity questions.*** | | | | |
| **VALIDITY QUESTIONS** | | | | |
| 1. **Was the research question clearly stated?**    1. Was the specific intervention(s) or procedure (independent variable(s)) identified?    2. Was the outcome(s) (dependent variable(s)) clearly indicated?    3. Were the target population and setting specified? | **Yes** | No | Unclear | N/A |
| 1. **Was the selection of study subjects/patients free from bias?**    1. Were inclusion/exclusion criteria specified (e.g., risk, point in disease progression, diagnostic or prognosis criteria), and with sufficient detail and without omitting criteria critical to the study?    2. Were criteria applied equally to all study groups?    3. Were health, demographics, and other characteristics of subjects described?    4. Were the subjects/patients a representative sample of the relevant population? | **Yes** | No | Unclear | N/A |
| 1. **Were study groups comparable?**    1. Was the method of assigning subjects/patients to groups described and unbiased? (Method of randomization identified if RCT)    2. Were distribution of disease status, prognostic factors, and other factors (e.g., demographics) similar across study groups at baseline?    3. Were concurrent controls used? (Concurrent preferred over historical controls.)    4. If cohort study or cross-sectional study, were groups comparable on important confounding factors and/or were preexisting differences accounted for by using appropriate adjustments in statistical analysis?    5. If case control study, were potential confounding factors comparable for cases and controls? (If case series or trial with subjects serving as own control, this criterion is not applicable. Criterion may not be applicable in some cross-sectional studies.)    6. If diagnostic test, was there an independent blind comparison with an appropriate reference standard (e.g., “gold standard”)? | Yes | No | Unclear | **N/A** |
| 1. **Was method of handling withdrawals described?**    1. Were follow up methods described and the same for all groups?    2. Was the number, characteristics of withdrawals (i.e., dropouts, lost to follow up, attrition rate) and/or response rate (cross-sectional studies) described for each group? (Follow up goal for a strong study is 80%.)    3. Were all enrolled subjects/patients (in the original sample) accounted for?    4. Were reasons for withdrawals similar across groups?    5. If diagnostic test, was decision to perform reference test not dependent on results of test under study? | Yes | No | **Unclear** | **N/A** |
| 1. **Was blinding used to prevent introduction of bias?**    1. In intervention study, were subjects, clinicians/practitioners, and investigators blinded to treatment group, as appropriate? | Yes | No | Unclear | **N/A** |

| - 1. Were data collectors blinded for outcomes assessment? (If outcome is measured using an objective test, such as a lab value, this criterion is assumed to be met.)   2. In cohort study or cross-sectional study, were measurements of outcomes and risk factors blinded?   3. In case control study, was case definition explicit and case ascertainment not influenced by exposure status?   4. In diagnostic study, were test results blinded to patient history and other test results? |  | | | |
| --- | --- | --- | --- | --- |
| 1. **Were intervention/therapeutic regimens/exposure factor or procedure and any comparison(s) described in detail? Were intervening factors described?**    1. In RCT or other intervention trial, were protocols described for all regimens studied?    2. n observational study, were interventions, study settings, and clinicians/provider described?    3. Was the intensity and duration of the intervention or exposure factor sufficient to produce a meaningful effect?    4. Was the amount of exposure and, if relevant, subject/patient compliance measured?    5. Were co-interventions (e.g., ancillary treatments, other therapies) described?    6. Were extra or unplanned treatments described?    7. Was the information for 6.4, 6.5, and 6.6 assessed the same way for all groups?    8. In diagnostic study, were details of test administration and replication sufficient? | **Yes** | No | **Unclear** | N/A |
| 1. **Were outcomes clearly defined and the measurements valid and reliable?**    1. Were primary and secondary endpoints described and relevant to the question?    2. Were mental health measures appropriate to question and outcomes of concern?    3. Was the period of follow-up long enough for important outcome(s) to occur?    4. Were the observations and measurements based on standard, valid, and reliable data collection instruments/tests/procedures?    5. Was the measurement of effect at an appropriate level of precision?    6. Were other factors accounted for (measured) that could affect outcomes?    7. Were the measurements conducted consistently across groups? | **Yes** | No | Unclear | N/A |
| 1. **Was the statistical analysis appropriate for the study design and type of outcome indicators?**    1. Were statistical analyses adequately described the results reported appropriately?    2. Were correct statistical tests used and assumptions of test not violated?    3. Were statistics reported with levels of significance and/or confidence intervals?    4. Was “intent to treat” analysis of outcomes done (and as appropriate, was there an analysis of outcomes for those maximally exposed or a dose-response analysis)?    5. Were adequate adjustments made for effects of confounding factors that might have affected the outcomes (e.g., multivariate analyses)?    6. Was clinical significance as well as statistical significance reported?    7. If negative findings, was a power calculation reported to address type 2 error? | **Yes** | No | Unclear | N/A |
| 1. **Are conclusions supported by results with biases and limitations taken into consideration?**    1. Is there a discussion of findings?    2. Are biases and study limitations identified and discussed? | **Yes** | No | Unclear | N/A |
| 1. **Is bias due to study’s funding or sponsorship unlikely?**    1. Were sources of funding and investigators’ affiliations described?    2. Was there no apparent conflict of interest? | **Yes** | No | Unclear | N/A |
| **MINUS/NEGATIVE (-)**  *If most (six or more) of the answers to the above validity questions are “No,” the report should be designated with a minus (-) symbol on the Evidence Worksheet.* | | | | |
| **NEUTRAL (**∅**)**  *If the answers to validity criteria questions 2, 3, 6, and 7 do not indicate that the study is exceptionally strong, the report should be designated with a neutral* (∅) *symbol on the Evidence Worksheet.* | | | | |
| **PLUS/POSITIVE (+)**  *If most of the answers to the above validity questions are “Yes” (including criteria 2, 3, 6, 7 and at least one additional “Yes”), the report should be designated with a plus symbol (+) on the Evidence Worksheet.* | | | | |

# Final score: Positive (+)

# Quality Criteria Checklist: Primary Research

1. **An unsupervised machine learning approach using passive movement data to understand depression and schizophrenia**

Quality Criteria Checklist: Primary Research

| **RELEVANCE QUESTIONS** | | | | |
| --- | --- | --- | --- | --- |
| 1. Would implementing the studied intervention or procedure (if found successful) result in improved outcomes for the patients/clients/population group? (NA for some Epi studies) | **Yes** | No | Unclear | N/A |
| 2. Did the authors study an outcome (dependent variable) or topic that the patients/clients/population group would care about? | **Yes** | No | Unclear | N/A |
| 3. Is the focus of the intervention or procedure (independent variable) or topic of study a common issue of concern to mental health practice? | **Yes** | No | Unclear | N/A |
| 4. Is the intervention or procedure feasible? (NA for some epidemiological studies) | **Yes** | No | Unclear | N/A |
| ***If the answers to all of the above relevance questions are “Yes,” the report is eligible for designation with a plus (+) on the Evidence Quality Worksheet, depending on answers to the following validity questions.*** | | | | |
| **VALIDITY QUESTIONS** | | | | |
| 1. **Was the research question clearly stated?**    1. Was the specific intervention(s) or procedure (independent variable(s)) identified?    2. Was the outcome(s) (dependent variable(s)) clearly indicated?    3. Were the target population and setting specified? | **Yes** | No | Unclear | N/A |
| 1. **Was the selection of study subjects/patients free from bias?**    1. Were inclusion/exclusion criteria specified (e.g., risk, point in disease progression, diagnostic or prognosis criteria), and with sufficient detail and without omitting criteria critical to the study?    2. Were criteria applied equally to all study groups?    3. Were health, demographics, and other characteristics of subjects described?    4. Were the subjects/patients a representative sample of the relevant population? | **Yes** | No | Unclear | N/A |
| 1. **Were study groups comparable?**    1. Was the method of assigning subjects/patients to groups described and unbiased? (Method of randomization identified if RCT)    2. Were distribution of disease status, prognostic factors, and other factors (e.g., demographics) similar across study groups at baseline?    3. Were concurrent controls used? (Concurrent preferred over historical controls.)    4. If cohort study or cross-sectional study, were groups comparable on important confounding factors and/or were preexisting differences accounted for by using appropriate adjustments in statistical analysis?    5. If case control study, were potential confounding factors comparable for cases and controls? (If case series or trial with subjects serving as own control, this criterion is not applicable. Criterion may not be applicable in some cross-sectional studies.)    6. If diagnostic test, was there an independent blind comparison with an appropriate reference standard (e.g., “gold standard”)? | Yes | No | **Unclear** | N/A |
| 1. **Was method of handling withdrawals described?**    1. Were follow up methods described and the same for all groups?    2. Was the number, characteristics of withdrawals (i.e., dropouts, lost to follow up, attrition rate) and/or response rate (cross-sectional studies) described for each group? (Follow up goal for a strong study is 80%.)    3. Were all enrolled subjects/patients (in the original sample) accounted for?    4. Were reasons for withdrawals similar across groups?    5. If diagnostic test, was decision to perform reference test not dependent on results of test under study? | Yes | No | Unclear | **N/A** |
| 1. **Was blinding used to prevent introduction of bias?**    1. In intervention study, were subjects, clinicians/practitioners, and investigators blinded to treatment group, as appropriate? | Yes | No | Unclear | **N/A** |

| - 1. Were data collectors blinded for outcomes assessment? (If outcome is measured using an objective test, such as a lab value, this criterion is assumed to be met.)   2. In cohort study or cross-sectional study, were measurements of outcomes and risk factors blinded?   3. In case control study, was case definition explicit and case ascertainment not influenced by exposure status?   4. In diagnostic study, were test results blinded to patient history and other test results? |  | | | |
| --- | --- | --- | --- | --- |
| 1. **Were intervention/therapeutic regimens/exposure factor or procedure and any comparison(s) described in detail? Were intervening factors described?**    1. In RCT or other intervention trial, were protocols described for all regimens studied?    2. n observational study, were interventions, study settings, and clinicians/provider described?    3. Was the intensity and duration of the intervention or exposure factor sufficient to produce a meaningful effect?    4. Was the amount of exposure and, if relevant, subject/patient compliance measured?    5. Were co-interventions (e.g., ancillary treatments, other therapies) described?    6. Were extra or unplanned treatments described?    7. Was the information for 6.4, 6.5, and 6.6 assessed the same way for all groups?    8. In diagnostic study, were details of test administration and replication sufficient? | **Yes** | No | **Unclear** | N/A |
| 1. **Were outcomes clearly defined and the measurements valid and reliable?**    1. Were primary and secondary endpoints described and relevant to the question?    2. Were mental health measures appropriate to question and outcomes of concern?    3. Was the period of follow-up long enough for important outcome(s) to occur?    4. Were the observations and measurements based on standard, valid, and reliable data collection instruments/tests/procedures?    5. Was the measurement of effect at an appropriate level of precision?    6. Were other factors accounted for (measured) that could affect outcomes?    7. Were the measurements conducted consistently across groups? | **Yes** | No | Unclear | N/A |
| 1. **Was the statistical analysis appropriate for the study design and type of outcome indicators?**    1. Were statistical analyses adequately described the results reported appropriately?    2. Were correct statistical tests used and assumptions of test not violated?    3. Were statistics reported with levels of significance and/or confidence intervals?    4. Was “intent to treat” analysis of outcomes done (and as appropriate, was there an analysis of outcomes for those maximally exposed or a dose-response analysis)?    5. Were adequate adjustments made for effects of confounding factors that might have affected the outcomes (e.g., multivariate analyses)?    6. Was clinical significance as well as statistical significance reported?    7. If negative findings, was a power calculation reported to address type 2 error? | **Yes** | No | Unclear | N/A |
| 1. **Are conclusions supported by results with biases and limitations taken into consideration?**    1. Is there a discussion of findings?    2. Are biases and study limitations identified and discussed? | **Yes** | No | Unclear | N/A |
| 1. **Is bias due to study’s funding or sponsorship unlikely?**    1. Were sources of funding and investigators’ affiliations described?    2. Was there no apparent conflict of interest? | **Yes** | No | Unclear | N/A |
| **MINUS/NEGATIVE (-)**  *If most (six or more) of the answers to the above validity questions are “No,” the report should be designated with a minus (-) symbol on the Evidence Worksheet.* | | | | |
| **NEUTRAL (**∅**)**  *If the answers to validity criteria questions 2, 3, 6, and 7 do not indicate that the study is exceptionally strong, the report should be designated with a neutral* (∅) *symbol on the Evidence Worksheet.* | | | | |
| **PLUS/POSITIVE (+)**  *If most of the answers to the above validity questions are “Yes” (including criteria 2, 3, 6, 7 and at least one additional “Yes”), the report should be designated with a plus symbol (+) on the Evidence Worksheet.* | | | | |

# Final score: Positive (+)

# Quality Criteria Checklist: Primary Research

**5. An Observational Pilot Study using a Digital Phenotyping Approach in Patients with Major Depressive Disorder Treated with Trazodone**

Quality Criteria Checklist: Primary Research

| **RELEVANCE QUESTIONS** | | | | |
| --- | --- | --- | --- | --- |
| 1. Would implementing the studied intervention or procedure (if found successful) result in improved outcomes for the patients/clients/population group? (NA for some Epi studies) | **Yes** | No | Unclear | N/A |
| 2. Did the authors study an outcome (dependent variable) or topic that the patients/clients/population group would care about? | **Yes** | No | Unclear | N/A |
| 3. Is the focus of the intervention or procedure (independent variable) or topic of study a common issue of concern to mental health practice? | **Yes** | No | Unclear | N/A |
| 4. Is the intervention or procedure feasible? (NA for some epidemiological studies) | **Yes** | No | Unclear | N/A |
| ***If the answers to all of the above relevance questions are “Yes,” the report is eligible for designation with a plus (+) on the Evidence Quality Worksheet, depending on answers to the following validity questions.*** | | | | |
| **VALIDITY QUESTIONS** | | | | |
| 1. **Was the research question clearly stated?**    1. Was the specific intervention(s) or procedure (independent variable(s)) identified?    2. Was the outcome(s) (dependent variable(s)) clearly indicated?    3. Were the target population and setting specified? | **Yes** | No | Unclear | N/A |
| 1. **Was the selection of study subjects/patients free from bias?**    1. Were inclusion/exclusion criteria specified (e.g., risk, point in disease progression, diagnostic or prognosis criteria), and with sufficient detail and without omitting criteria critical to the study?    2. Were criteria applied equally to all study groups?    3. Were health, demographics, and other characteristics of subjects described?    4. Were the subjects/patients a representative sample of the relevant population? | **Yes** | No | **Unclear** | N/A |
| 1. **Were study groups comparable?**    1. Was the method of assigning subjects/patients to groups described and unbiased? (Method of randomization identified if RCT)    2. Were distribution of disease status, prognostic factors, and other factors (e.g., demographics) similar across study groups at baseline?    3. Were concurrent controls used? (Concurrent preferred over historical controls.)    4. If cohort study or cross-sectional study, were groups comparable on important confounding factors and/or were preexisting differences accounted for by using appropriate adjustments in statistical analysis?    5. If case control study, were potential confounding factors comparable for cases and controls? (If case series or trial with subjects serving as own control, this criterion is not applicable. Criterion may not be applicable in some cross-sectional studies.)    6. If diagnostic test, was there an independent blind comparison with an appropriate reference standard (e.g., “gold standard”)? | Yes | No | Unclear | **N/A** |
| 1. **Was method of handling withdrawals described?**    1. Were follow up methods described and the same for all groups?    2. Was the number, characteristics of withdrawals (i.e., dropouts, lost to follow up, attrition rate) and/or response rate (cross-sectional studies) described for each group? (Follow up goal for a strong study is 80%.)    3. Were all enrolled subjects/patients (in the original sample) accounted for?    4. Were reasons for withdrawals similar across groups?    5. If diagnostic test, was decision to perform reference test not dependent on results of test under study? | **Yes** | No | Unclear | N/A |
| 1. **Was blinding used to prevent introduction of bias?**    1. In intervention study, were subjects, clinicians/practitioners, and investigators blinded to treatment group, as appropriate? | Yes | No | Unclear | **N/A** |

| - 1. Were data collectors blinded for outcomes assessment? (If outcome is measured using an objective test, such as a lab value, this criterion is assumed to be met.)   2. In cohort study or cross-sectional study, were measurements of outcomes and risk factors blinded?   3. In case control study, was case definition explicit and case ascertainment not influenced by exposure status?   4. In diagnostic study, were test results blinded to patient history and other test results? |  | | | |
| --- | --- | --- | --- | --- |
| 1. **Were intervention/therapeutic regimens/exposure factor or procedure and any comparison(s) described in detail? Were intervening factors described?**    1. In RCT or other intervention trial, were protocols described for all regimens studied?    2. n observational study, were interventions, study settings, and clinicians/provider described?    3. Was the intensity and duration of the intervention or exposure factor sufficient to produce a meaningful effect?    4. Was the amount of exposure and, if relevant, subject/patient compliance measured?    5. Were co-interventions (e.g., ancillary treatments, other therapies) described?    6. Were extra or unplanned treatments described?    7. Was the information for 6.4, 6.5, and 6.6 assessed the same way for all groups?    8. In diagnostic study, were details of test administration and replication sufficient? | **Yes** | No | Unclear | N/A |
| 1. **Were outcomes clearly defined and the measurements valid and reliable?**    1. Were primary and secondary endpoints described and relevant to the question?    2. Were mental health measures appropriate to question and outcomes of concern?    3. Was the period of follow-up long enough for important outcome(s) to occur?    4. Were the observations and measurements based on standard, valid, and reliable data collection instruments/tests/procedures?    5. Was the measurement of effect at an appropriate level of precision?    6. Were other factors accounted for (measured) that could affect outcomes?    7. Were the measurements conducted consistently across groups? | **Yes** | No | Unclear | N/A |
| 1. **Was the statistical analysis appropriate for the study design and type of outcome indicators?**    1. Were statistical analyses adequately described the results reported appropriately?    2. Were correct statistical tests used and assumptions of test not violated?    3. Were statistics reported with levels of significance and/or confidence intervals?    4. Was “intent to treat” analysis of outcomes done (and as appropriate, was there an analysis of outcomes for those maximally exposed or a dose-response analysis)?    5. Were adequate adjustments made for effects of confounding factors that might have affected the outcomes (e.g., multivariate analyses)?    6. Was clinical significance as well as statistical significance reported?    7. If negative findings, was a power calculation reported to address type 2 error? | Yes | No | **Unclear** | N/A |
| 1. **Are conclusions supported by results with biases and limitations taken into consideration?**    1. Is there a discussion of findings?    2. Are biases and study limitations identified and discussed? | **Yes** | No | Unclear | N/A |
| 1. **Is bias due to study’s funding or sponsorship unlikely?**    1. Were sources of funding and investigators’ affiliations described?    2. Was there no apparent conflict of interest? | **Yes** | No | Unclear | N/A |
| **MINUS/NEGATIVE (-)**   1. *If most (six or more) of the answers to the above validity questions are “No,” the report should be designated with a minus (-) symbol on the Evidence Worksheet.* |  |  |  |  |
| **NEUTRAL (**∅**)**  *If the answers to validity criteria questions 2, 3, 6, and 7 do not indicate that the study is exceptionally strong, the report should be designated with a neutral* (∅) *symbol on the Evidence Worksheet.* |  |  |  |  |
| **PLUS/POSITIVE (+)**  *If most of the answers to the above validity questions are “Yes” (including criteria 2, 3, 6, 7 and at least one additional “Yes”), the report should be designated with a plus symbol (+) on the Evidence Worksheet.* |  |  |  |  |

# Final score: Positive (+)

# Quality Criteria Checklist: Primary Research

1. **Large-scale digital phenotyping: Identifying depression and anxiety indicators in a general UK population with over 10,000 participants**

Quality Criteria Checklist: Primary Research

| **RELEVANCE QUESTIONS** | | | | |
| --- | --- | --- | --- | --- |
| 1. Would implementing the studied intervention or procedure (if found successful) result in improved outcomes for the patients/clients/population group? (NA for some Epi studies) | **Yes** | No | Unclear | N/A |
| 2. Did the authors study an outcome (dependent variable) or topic that the patients/clients/population group would care about? | **Yes** | No | Unclear | N/A |
| 3. Is the focus of the intervention or procedure (independent variable) or topic of study a common issue of concern to mental health practice? | **Yes** | No | Unclear | N/A |
| 4. Is the intervention or procedure feasible? (NA for some epidemiological studies) | **Yes** | No | Unclear | N/A |
| ***If the answers to all of the above relevance questions are “Yes,” the report is eligible for designation with a plus (+) on the Evidence Quality Worksheet, depending on answers to the following validity questions.*** | | | | |
| **VALIDITY QUESTIONS** | | | | |
| 1. **Was the research question clearly stated?**    1. Was the specific intervention(s) or procedure (independent variable(s)) identified?    2. Was the outcome(s) (dependent variable(s)) clearly indicated?    3. Were the target population and setting specified? | **Yes** | No | Unclear | N/A |
| 1. **Was the selection of study subjects/patients free from bias?**    1. Were inclusion/exclusion criteria specified (e.g., risk, point in disease progression, diagnostic or prognosis criteria), and with sufficient detail and without omitting criteria critical to the study?    2. Were criteria applied equally to all study groups?    3. Were health, demographics, and other characteristics of subjects described?    4. Were the subjects/patients a representative sample of the relevant population? | **Yes** | No | Unclear | N/A |
| 1. **Were study groups comparable?**    1. Was the method of assigning subjects/patients to groups described and unbiased? (Method of randomization identified if RCT)    2. Were distribution of disease status, prognostic factors, and other factors (e.g., demographics) similar across study groups at baseline?    3. Were concurrent controls used? (Concurrent preferred over historical controls.)    4. If cohort study or cross-sectional study, were groups comparable on important confounding factors and/or were preexisting differences accounted for by using appropriate adjustments in statistical analysis?    5. If case control study, were potential confounding factors comparable for cases and controls? (If case series or trial with subjects serving as own control, this criterion is not applicable. Criterion may not be applicable in some cross-sectional studies.)    6. If diagnostic test, was there an independent blind comparison with an appropriate reference standard (e.g., “gold standard”)? | Yes | No | Unclear | **N/A** |
| 1. **Was method of handling withdrawals described?**    1. Were follow up methods described and the same for all groups?    2. Was the number, characteristics of withdrawals (i.e., dropouts, lost to follow up, attrition rate) and/or response rate (cross-sectional studies) described for each group? (Follow up goal for a strong study is 80%.)    3. Were all enrolled subjects/patients (in the original sample) accounted for?    4. Were reasons for withdrawals similar across groups?    5. If diagnostic test, was decision to perform reference test not dependent on results of test under study? | **Yes** | No | Unclear | N/A |
| 1. **Was blinding used to prevent introduction of bias?**    1. In intervention study, were subjects, clinicians/practitioners, and investigators blinded to treatment group, as appropriate? | Yes | No | Unclear | **N/A** |

| - 1. Were data collectors blinded for outcomes assessment? (If outcome is measured using an objective test, such as a lab value, this criterion is assumed to be met.)   2. In cohort study or cross-sectional study, were measurements of outcomes and risk factors blinded?   3. In case control study, was case definition explicit and case ascertainment not influenced by exposure status?   4. In diagnostic study, were test results blinded to patient history and other test results? |  | | | |
| --- | --- | --- | --- | --- |
| 1. **Were intervention/therapeutic regimens/exposure factor or procedure and any comparison(s) described in detail? Were intervening factors described?**    1. In RCT or other intervention trial, were protocols described for all regimens studied?    2. n observational study, were interventions, study settings, and clinicians/provider described?    3. Was the intensity and duration of the intervention or exposure factor sufficient to produce a meaningful effect?    4. Was the amount of exposure and, if relevant, subject/patient compliance measured?    5. Were co-interventions (e.g., ancillary treatments, other therapies) described?    6. Were extra or unplanned treatments described?    7. Was the information for 6.4, 6.5, and 6.6 assessed the same way for all groups?    8. In diagnostic study, were details of test administration and replication sufficient? | **Yes** | No | Unclear | N/A |
| 1. **Were outcomes clearly defined and the measurements valid and reliable?**    1. Were primary and secondary endpoints described and relevant to the question?    2. Were mental health measures appropriate to question and outcomes of concern?    3. Was the period of follow-up long enough for important outcome(s) to occur?    4. Were the observations and measurements based on standard, valid, and reliable data collection instruments/tests/procedures?    5. Was the measurement of effect at an appropriate level of precision?    6. Were other factors accounted for (measured) that could affect outcomes?    7. Were the measurements conducted consistently across groups? | **Yes** | No | Unclear | N/A |
| 1. **Was the statistical analysis appropriate for the study design and type of outcome indicators?**    1. Were statistical analyses adequately described the results reported appropriately?    2. Were correct statistical tests used and assumptions of test not violated?    3. Were statistics reported with levels of significance and/or confidence intervals?    4. Was “intent to treat” analysis of outcomes done (and as appropriate, was there an analysis of outcomes for those maximally exposed or a dose-response analysis)?    5. Were adequate adjustments made for effects of confounding factors that might have affected the outcomes (e.g., multivariate analyses)?    6. Was clinical significance as well as statistical significance reported?    7. If negative findings, was a power calculation reported to address type 2 error? | **Yes** | No | Unclear | N/A |
| 1. **Are conclusions supported by results with biases and limitations taken into consideration?**    1. Is there a discussion of findings?    2. Are biases and study limitations identified and discussed? | **Yes** | No | Unclear | N/A |
| 1. **Is bias due to study’s funding or sponsorship unlikely?**    1. Were sources of funding and investigators’ affiliations described?    2. Was there no apparent conflict of interest? | **Yes** | No | Unclear | N/A |
| **MINUS/NEGATIVE (-)**   1. *If most (six or more) of the answers to the above validity questions are “No,” the report should be designated with a minus (-) symbol on the Evidence Worksheet.* |  |  |  |  |
| **NEUTRAL (**∅**)**  *If the answers to validity criteria questions 2, 3, 6, and 7 do not indicate that the study is exceptionally strong, the report should be designated with a neutral* (∅) *symbol on the Evidence Worksheet.* |  |  |  |  |
| **PLUS/POSITIVE (+)**  *If most of the answers to the above validity questions are “Yes” (including criteria 2, 3, 6, 7 and at least one additional “Yes”), the report should be designated with a plus symbol (+) on the Evidence Worksheet.* |  |  |  |  |

# Final score: Positive (+)

# Quality Criteria Checklist: Primary Research

**7. Digital Phenotyping of Geriatric Depression Using a Community-Based Digital Mental Health Monitoring Platform for Socially Vulnerable Older Adults and Their Community Caregivers: 6-Week Living Lab Single-Arm Pilot Study**

Quality Criteria Checklist: Primary Research

| **RELEVANCE QUESTIONS** | | | | |
| --- | --- | --- | --- | --- |
| 1. Would implementing the studied intervention or procedure (if found successful) result in improved outcomes for the patients/clients/population group? (NA for some Epi studies) | **Yes** | No | Unclear | N/A |
| 2. Did the authors study an outcome (dependent variable) or topic that the patients/clients/population group would care about? | **Yes** | No | Unclear | N/A |
| 3. Is the focus of the intervention or procedure (independent variable) or topic of study a common issue of concern to mental health practice? | **Yes** | No | Unclear | N/A |
| 4. Is the intervention or procedure feasible? (NA for some epidemiological studies) | **Yes** | No | Unclear | N/A |
| ***If the answers to all of the above relevance questions are “Yes,” the report is eligible for designation with a plus (+) on the Evidence Quality Worksheet, depending on answers to the following validity questions.*** | | | | |
| **VALIDITY QUESTIONS** | | | | |
| 1. **Was the research question clearly stated?**    1. Was the specific intervention(s) or procedure (independent variable(s)) identified?    2. Was the outcome(s) (dependent variable(s)) clearly indicated?    3. Were the target population and setting specified? | **Yes** | No | Unclear | N/A |
| 1. **Was the selection of study subjects/patients free from bias?**    1. Were inclusion/exclusion criteria specified (e.g., risk, point in disease progression, diagnostic or prognosis criteria), and with sufficient detail and without omitting criteria critical to the study?    2. Were criteria applied equally to all study groups?    3. Were health, demographics, and other characteristics of subjects described?    4. Were the subjects/patients a representative sample of the relevant population? | Yes | **No** | Unclear | N/A |
| 1. **Were study groups comparable?**    1. Was the method of assigning subjects/patients to groups described and unbiased? (Method of randomization identified if RCT)    2. Were distribution of disease status, prognostic factors, and other factors (e.g., demographics) similar across study groups at baseline?    3. Were concurrent controls used? (Concurrent preferred over historical controls.)    4. If cohort study or cross-sectional study, were groups comparable on important confounding factors and/or were preexisting differences accounted for by using appropriate adjustments in statistical analysis?    5. If case control study, were potential confounding factors comparable for cases and controls? (If case series or trial with subjects serving as own control, this criterion is not applicable. Criterion may not be applicable in some cross-sectional studies.)    6. If diagnostic test, was there an independent blind comparison with an appropriate reference standard (e.g., “gold standard”)? | Yes | No | Unclear | **N/A** |
| 1. **Was method of handling withdrawals described?**    1. Were follow up methods described and the same for all groups?    2. Was the number, characteristics of withdrawals (i.e., dropouts, lost to follow up, attrition rate) and/or response rate (cross-sectional studies) described for each group? (Follow up goal for a strong study is 80%.)    3. Were all enrolled subjects/patients (in the original sample) accounted for?    4. Were reasons for withdrawals similar across groups?    5. If diagnostic test, was decision to perform reference test not dependent on results of test under study? | **Yes** | No | Unclear | N/A |
| 1. **Was blinding used to prevent introduction of bias?**    1. In intervention study, were subjects, clinicians/practitioners, and investigators blinded to treatment group, as appropriate? | Yes | No | Unclear | **N/A** |

| - 1. Were data collectors blinded for outcomes assessment? (If outcome is measured using an objective test, such as a lab value, this criterion is assumed to be met.)   2. In cohort study or cross-sectional study, were measurements of outcomes and risk factors blinded?   3. In case control study, was case definition explicit and case ascertainment not influenced by exposure status?   4. In diagnostic study, were test results blinded to patient history and other test results? |  | | | |
| --- | --- | --- | --- | --- |
| 1. **Were intervention/therapeutic regimens/exposure factor or procedure and any comparison(s) described in detail? Were intervening factors described?**    1. In RCT or other intervention trial, were protocols described for all regimens studied?    2. n observational study, were interventions, study settings, and clinicians/provider described?    3. Was the intensity and duration of the intervention or exposure factor sufficient to produce a meaningful effect?    4. Was the amount of exposure and, if relevant, subject/patient compliance measured?    5. Were co-interventions (e.g., ancillary treatments, other therapies) described?    6. Were extra or unplanned treatments described?    7. Was the information for 6.4, 6.5, and 6.6 assessed the same way for all groups?    8. In diagnostic study, were details of test administration and replication sufficient? | Yes | No | **Unclear** | N/A |
| 1. **Were outcomes clearly defined and the measurements valid and reliable?**    1. Were primary and secondary endpoints described and relevant to the question?    2. Were mental health measures appropriate to question and outcomes of concern?    3. Was the period of follow-up long enough for important outcome(s) to occur?    4. Were the observations and measurements based on standard, valid, and reliable data collection instruments/tests/procedures?    5. Was the measurement of effect at an appropriate level of precision?    6. Were other factors accounted for (measured) that could affect outcomes?    7. Were the measurements conducted consistently across groups? | **Yes** | No | Unclear | N/A |
| 1. **Was the statistical analysis appropriate for the study design and type of outcome indicators?**    1. Were statistical analyses adequately described the results reported appropriately?    2. Were correct statistical tests used and assumptions of test not violated?    3. Were statistics reported with levels of significance and/or confidence intervals?    4. Was “intent to treat” analysis of outcomes done (and as appropriate, was there an analysis of outcomes for those maximally exposed or a dose-response analysis)?    5. Were adequate adjustments made for effects of confounding factors that might have affected the outcomes (e.g., multivariate analyses)?    6. Was clinical significance as well as statistical significance reported?    7. If negative findings, was a power calculation reported to address type 2 error? | **Yes** | No | Unclear | N/A |
| 1. **Are conclusions supported by results with biases and limitations taken into consideration?**    1. Is there a discussion of findings?    2. Are biases and study limitations identified and discussed? | **Yes** | No | Unclear | N/A |
| 1. **Is bias due to study’s funding or sponsorship unlikely?**    1. Were sources of funding and investigators’ affiliations described?    2. Was there no apparent conflict of interest? | **Yes** | No | Unclear | N/A |
| **MINUS/NEGATIVE (-)**   1. *If most (six or more) of the answers to the above validity questions are “No,” the report should be designated with a minus (-) symbol on the Evidence Worksheet.* |  |  |  |  |
| **NEUTRAL (**∅**)**  *If the answers to validity criteria questions 2, 3, 6, and 7 do not indicate that the study is exceptionally strong, the report should be designated with a neutral* (∅) *symbol on the Evidence Worksheet.* |  |  |  |  |
| **PLUS/POSITIVE (+)**  *If most of the answers to the above validity questions are “Yes” (including criteria 2, 3, 6, 7 and at least one additional “Yes”), the report should be designated with a plus symbol (+) on the Evidence Worksheet.* |  |  |  |  |

# Final score: NEUTRAL (∅)

# Quality Criteria Checklist: Primary Research

1. **Monitoring Changes in Depression Severity Using Wearable and Mobile Sensors**

Quality Criteria Checklist: Primary Research

| **RELEVANCE QUESTIONS** | | | | |
| --- | --- | --- | --- | --- |
| 1. Would implementing the studied intervention or procedure (if found successful) result in improved outcomes for the patients/clients/population group? (NA for some Epi studies) | **Yes** | No | Unclear | N/A |
| 2. Did the authors study an outcome (dependent variable) or topic that the patients/clients/population group would care about? | **Yes** | No | Unclear | N/A |
| 3. Is the focus of the intervention or procedure (independent variable) or topic of study a common issue of concern to mental health practice? | **Yes** | No | Unclear | N/A |
| 4. Is the intervention or procedure feasible? (NA for some epidemiological studies) | Yes | No | Unclear | N/A |
| ***If the answers to all of the above relevance questions are “Yes,” the report is eligible for designation with a plus (+) on the Evidence Quality Worksheet, depending on answers to the following validity questions.*** | | | | |
| **VALIDITY QUESTIONS** | | | | |
| 1. **Was the research question clearly stated?**    1. Was the specific intervention(s) or procedure (independent variable(s)) identified?    2. Was the outcome(s) (dependent variable(s)) clearly indicated?    3. Were the target population and setting specified? | **Yes** | No | Unclear | N/A |
| 1. **Was the selection of study subjects/patients free from bias?**    1. Were inclusion/exclusion criteria specified (e.g., risk, point in disease progression, diagnostic or prognosis criteria), and with sufficient detail and without omitting criteria critical to the study?    2. Were criteria applied equally to all study groups?    3. Were health, demographics, and other characteristics of subjects described?    4. Were the subjects/patients a representative sample of the relevant population? | **Yes** | No | Unclear | N/A |
| 1. **Were study groups comparable?**    1. Was the method of assigning subjects/patients to groups described and unbiased? (Method of randomization identified if RCT)    2. Were distribution of disease status, prognostic factors, and other factors (e.g., demographics) similar across study groups at baseline?    3. Were concurrent controls used? (Concurrent preferred over historical controls.)    4. If cohort study or cross-sectional study, were groups comparable on important confounding factors and/or were preexisting differences accounted for by using appropriate adjustments in statistical analysis?    5. If case control study, were potential confounding factors comparable for cases and controls? (If case series or trial with subjects serving as own control, this criterion is not applicable. Criterion may not be applicable in some cross-sectional studies.)    6. If diagnostic test, was there an independent blind comparison with an appropriate reference standard (e.g., “gold standard”)? | Yes | No | Unclear | **N/A** |
| 1. **Was method of handling withdrawals described?**    1. Were follow up methods described and the same for all groups?    2. Was the number, characteristics of withdrawals (i.e., dropouts, lost to follow up, attrition rate) and/or response rate (cross-sectional studies) described for each group? (Follow up goal for a strong study is 80%.)    3. Were all enrolled subjects/patients (in the original sample) accounted for?    4. Were reasons for withdrawals similar across groups?    5. If diagnostic test, was decision to perform reference test not dependent on results of test under study? | **Yes** | No | Unclear | N/A |
| 1. **Was blinding used to prevent introduction of bias?**    1. In intervention study, were subjects, clinicians/practitioners, and investigators blinded to treatment group, as appropriate? | Yes | No | Unclear | **N/A** |

| - 1. Were data collectors blinded for outcomes assessment? (If outcome is measured using an objective test, such as a lab value, this criterion is assumed to be met.)   2. In cohort study or cross-sectional study, were measurements of outcomes and risk factors blinded?   3. In case control study, was case definition explicit and case ascertainment not influenced by exposure status?   4. In diagnostic study, were test results blinded to patient history and other test results? |  | | | |
| --- | --- | --- | --- | --- |
| 1. **Were intervention/therapeutic regimens/exposure factor or procedure and any comparison(s) described in detail? Were intervening factors described?**    1. In RCT or other intervention trial, were protocols described for all regimens studied?    2. n observational study, were interventions, study settings, and clinicians/provider described?    3. Was the intensity and duration of the intervention or exposure factor sufficient to produce a meaningful effect?    4. Was the amount of exposure and, if relevant, subject/patient compliance measured?    5. Were co-interventions (e.g., ancillary treatments, other therapies) described?    6. Were extra or unplanned treatments described?    7. Was the information for 6.4, 6.5, and 6.6 assessed the same way for all groups?    8. In diagnostic study, were details of test administration and replication sufficient? | **Yes** | No | **Unclear** | N/A |
| 1. **Were outcomes clearly defined and the measurements valid and reliable?**    1. Were primary and secondary endpoints described and relevant to the question?    2. Were mental health measures appropriate to question and outcomes of concern?    3. Was the period of follow-up long enough for important outcome(s) to occur?    4. Were the observations and measurements based on standard, valid, and reliable data collection instruments/tests/procedures?    5. Was the measurement of effect at an appropriate level of precision?    6. Were other factors accounted for (measured) that could affect outcomes?    7. Were the measurements conducted consistently across groups? | **Yes** | No | Unclear | N/A |
| 1. **Was the statistical analysis appropriate for the study design and type of outcome indicators?**    1. Were statistical analyses adequately described the results reported appropriately?    2. Were correct statistical tests used and assumptions of test not violated?    3. Were statistics reported with levels of significance and/or confidence intervals?    4. Was “intent to treat” analysis of outcomes done (and as appropriate, was there an analysis of outcomes for those maximally exposed or a dose-response analysis)?    5. Were adequate adjustments made for effects of confounding factors that might have affected the outcomes (e.g., multivariate analyses)?    6. Was clinical significance as well as statistical significance reported?    7. If negative findings, was a power calculation reported to address type 2 error? | **Yes** | No | Unclear | N/A |
| 1. **Are conclusions supported by results with biases and limitations taken into consideration?**    1. Is there a discussion of findings?    2. Are biases and study limitations identified and discussed? | **Yes** | No | Unclear | N/A |
| 1. **Is bias due to study’s funding or sponsorship unlikely?**    1. Were sources of funding and investigators’ affiliations described?    2. Was there no apparent conflict of interest? | **Yes** | No | Unclear | N/A |
| **MINUS/NEGATIVE (-)**   1. *If most (six or more) of the answers to the above validity questions are “No,” the report should be designated with a minus (-) symbol on the Evidence Worksheet.* |  |  |  |  |
| **NEUTRAL (**∅**)**  *If the answers to validity criteria questions 2, 3, 6, and 7 do not indicate that the study is exceptionally strong, the report should be designated with a neutral* (∅) *symbol on the Evidence Worksheet.* |  |  |  |  |
| **PLUS/POSITIVE (+)**  *If most of the answers to the above validity questions are “Yes” (including criteria 2, 3, 6, 7 and at least one additional “Yes”), the report should be designated with a plus symbol (+) on the Evidence Worksheet.* |  |  |  |  |

# Final score: Positive (+)

# Quality Criteria Checklist: Primary Research

**10.Tracking Depression Dynamics in College Students Using Mobile Phone and Wearable Sensing**

Quality Criteria Checklist: Primary Research

| **RELEVANCE QUESTIONS** | | | | |
| --- | --- | --- | --- | --- |
| 1. Would implementing the studied intervention or procedure (if found successful) result in improved outcomes for the patients/clients/population group? (NA for some Epi studies) | **Yes** | No | Unclear | N/A |
| 2. Did the authors study an outcome (dependent variable) or topic that the patients/clients/population group would care about? | **Yes** | No | Unclear | N/A |
| 3. Is the focus of the intervention or procedure (independent variable) or topic of study a common issue of concern to mental health practice? | **Yes** | No | Unclear | N/A |
| 4. Is the intervention or procedure feasible? (NA for some epidemiological studies) | **Yes** | No | Unclear | N/A |
| ***If the answers to all of the above relevance questions are “Yes,” the report is eligible for designation with a plus (+) on the Evidence Quality Worksheet, depending on answers to the following validity questions.*** | | | | |
| **VALIDITY QUESTIONS** | | | | |
| 1. **Was the research question clearly stated?**    1. Was the specific intervention(s) or procedure (independent variable(s)) identified?    2. Was the outcome(s) (dependent variable(s)) clearly indicated?    3. Were the target population and setting specified? | **Yes** | No | Unclear | N/A |
| 1. **Was the selection of study subjects/patients free from bias?**    1. Were inclusion/exclusion criteria specified (e.g., risk, point in disease progression, diagnostic or prognosis criteria), and with sufficient detail and without omitting criteria critical to the study?    2. Were criteria applied equally to all study groups?    3. Were health, demographics, and other characteristics of subjects described?    4. Were the subjects/patients a representative sample of the relevant population? | **Yes** | No | Unclear | N/A |
| 1. **Were study groups comparable?**    1. Was the method of assigning subjects/patients to groups described and unbiased? (Method of randomization identified if RCT)    2. Were distribution of disease status, prognostic factors, and other factors (e.g., demographics) similar across study groups at baseline?    3. Were concurrent controls used? (Concurrent preferred over historical controls.)    4. If cohort study or cross-sectional study, were groups comparable on important confounding factors and/or were preexisting differences accounted for by using appropriate adjustments in statistical analysis?    5. If case control study, were potential confounding factors comparable for cases and controls? (If case series or trial with subjects serving as own control, this criterion is not applicable. Criterion may not be applicable in some cross-sectional studies.)    6. If diagnostic test, was there an independent blind comparison with an appropriate reference standard (e.g., “gold standard”)? | Yes | No | Unclear | **N/A** |
| 1. **Was method of handling withdrawals described?**    1. Were follow up methods described and the same for all groups?    2. Was the number, characteristics of withdrawals (i.e., dropouts, lost to follow up, attrition rate) and/or response rate (cross-sectional studies) described for each group? (Follow up goal for a strong study is 80%.)    3. Were all enrolled subjects/patients (in the original sample) accounted for?    4. Were reasons for withdrawals similar across groups?    5. If diagnostic test, was decision to perform reference test not dependent on results of test under study? | **Yes** | No | Unclear | N/A |
| 1. **Was blinding used to prevent introduction of bias?**    1. In intervention study, were subjects, clinicians/practitioners, and investigators blinded to treatment group, as appropriate? | Yes | No | Unclear | **N/A** |

| - 1. Were data collectors blinded for outcomes assessment? (If outcome is measured using an objective test, such as a lab value, this criterion is assumed to be met.)   2. In cohort study or cross-sectional study, were measurements of outcomes and risk factors blinded?   3. In case control study, was case definition explicit and case ascertainment not influenced by exposure status?   4. In diagnostic study, were test results blinded to patient history and other test results? |  | | | |
| --- | --- | --- | --- | --- |
| 1. **Were intervention/therapeutic regimens/exposure factor or procedure and any comparison(s) described in detail? Were intervening factors described?**    1. In RCT or other intervention trial, were protocols described for all regimens studied?    2. n observational study, were interventions, study settings, and clinicians/provider described?    3. Was the intensity and duration of the intervention or exposure factor sufficient to produce a meaningful effect?    4. Was the amount of exposure and, if relevant, subject/patient compliance measured?    5. Were co-interventions (e.g., ancillary treatments, other therapies) described?    6. Were extra or unplanned treatments described?    7. Was the information for 6.4, 6.5, and 6.6 assessed the same way for all groups?    8. In diagnostic study, were details of test administration and replication sufficient? | **Yes** | No | Unclear | N/A |
| 1. **Were outcomes clearly defined and the measurements valid and reliable?**    1. Were primary and secondary endpoints described and relevant to the question?    2. Were mental health measures appropriate to question and outcomes of concern?    3. Was the period of follow-up long enough for important outcome(s) to occur?    4. Were the observations and measurements based on standard, valid, and reliable data collection instruments/tests/procedures?    5. Was the measurement of effect at an appropriate level of precision?    6. Were other factors accounted for (measured) that could affect outcomes?    7. Were the measurements conducted consistently across groups? | **Yes** | No | Unclear | N/A |
| 1. **Was the statistical analysis appropriate for the study design and type of outcome indicators?**    1. Were statistical analyses adequately described the results reported appropriately?    2. Were correct statistical tests used and assumptions of test not violated?    3. Were statistics reported with levels of significance and/or confidence intervals?    4. Was “intent to treat” analysis of outcomes done (and as appropriate, was there an analysis of outcomes for those maximally exposed or a dose-response analysis)?    5. Were adequate adjustments made for effects of confounding factors that might have affected the outcomes (e.g., multivariate analyses)?    6. Was clinical significance as well as statistical significance reported?    7. If negative findings, was a power calculation reported to address type 2 error? | **Yes** | No | Unclear | N/A |
| 1. **Are conclusions supported by results with biases and limitations taken into consideration?**    1. Is there a discussion of findings?    2. Are biases and study limitations identified and discussed? | **Yes** | No | Unclear | N/A |
| 1. **Is bias due to study’s funding or sponsorship unlikely?**    1. Were sources of funding and investigators’ affiliations described?    2. Was there no apparent conflict of interest? | **Yes** | No | Unclear | N/A |
| **MINUS/NEGATIVE (-)**   1. *If most (six or more) of the answers to the above validity questions are “No,” the report should be designated with a minus (-) symbol on the Evidence Worksheet.* |  |  |  |  |
| **NEUTRAL (**∅**)**  *If the answers to validity criteria questions 2, 3, 6, and 7 do not indicate that the study is exceptionally strong, the report should be designated with a neutral* (∅) *symbol on the Evidence Worksheet.* |  |  |  |  |
| **PLUS/POSITIVE (+)**  *If most of the answers to the above validity questions are “Yes” (including criteria 2, 3, 6, 7 and at least one additional “Yes”), the report should be designated with a plus symbol (+) on the Evidence Worksheet.* |  |  |  |  |

# Final score: POSITIVE (+)

# Quality Criteria Checklist: Primary Research

**11. STDD: Short-Term Depression Detection with Passive Sensing**

Quality Criteria Checklist: Primary Research

| **RELEVANCE QUESTIONS** | | | | |
| --- | --- | --- | --- | --- |
| 1. Would implementing the studied intervention or procedure (if found successful) result in improved outcomes for the patients/clients/population group? (NA for some Epi studies) | **Yes** | No | Unclear | N/A |
| 2. Did the authors study an outcome (dependent variable) or topic that the patients/clients/population group would care about? | **Yes** | No | Unclear | N/A |
| 3. Is the focus of the intervention or procedure (independent variable) or topic of study a common issue of concern to mental health practice? | **Yes** | No | Unclear | N/A |
| 4. Is the intervention or procedure feasible? (NA for some epidemiological studies) | **Yes** | No | Unclear | N/A |
| ***If the answers to all of the above relevance questions are “Yes,” the report is eligible for designation with a plus (+) on the Evidence Quality Worksheet, depending on answers to the following validity questions.*** | | | | |
| **VALIDITY QUESTIONS** | | | | |
| 1. **Was the research question clearly stated?**    1. Was the specific intervention(s) or procedure (independent variable(s)) identified?    2. Was the outcome(s) (dependent variable(s)) clearly indicated?    3. Were the target population and setting specified? | **Yes** | No | Unclear | N/A |
| 1. **Was the selection of study subjects/patients free from bias?**    1. Were inclusion/exclusion criteria specified (e.g., risk, point in disease progression, diagnostic or prognosis criteria), and with sufficient detail and without omitting criteria critical to the study?    2. Were criteria applied equally to all study groups?    3. Were health, demographics, and other characteristics of subjects described?    4. Were the subjects/patients a representative sample of the relevant population? | **Yes** | No | Unclear | N/A |
| 1. **Were study groups comparable?**    1. Was the method of assigning subjects/patients to groups described and unbiased? (Method of randomization identified if RCT)    2. Were distribution of disease status, prognostic factors, and other factors (e.g., demographics) similar across study groups at baseline?    3. Were concurrent controls used? (Concurrent preferred over historical controls.)    4. If cohort study or cross-sectional study, were groups comparable on important confounding factors and/or were preexisting differences accounted for by using appropriate adjustments in statistical analysis?    5. If case control study, were potential confounding factors comparable for cases and controls? (If case series or trial with subjects serving as own control, this criterion is not applicable. Criterion may not be applicable in some cross-sectional studies.)    6. If diagnostic test, was there an independent blind comparison with an appropriate reference standard (e.g., “gold standard”)? | Yes | **No** | Unclear | N/A |
| 1. **Was method of handling withdrawals described?**    1. Were follow up methods described and the same for all groups?    2. Was the number, characteristics of withdrawals (i.e., dropouts, lost to follow up, attrition rate) and/or response rate (cross-sectional studies) described for each group? (Follow up goal for a strong study is 80%.)    3. Were all enrolled subjects/patients (in the original sample) accounted for?    4. Were reasons for withdrawals similar across groups?    5. If diagnostic test, was decision to perform reference test not dependent on results of test under study? | **Yes** | No | Unclear | N/A |
| 1. **Was blinding used to prevent introduction of bias?**    1. In intervention study, were subjects, clinicians/practitioners, and investigators blinded to treatment group, as appropriate? | Yes | **No** | Unclear | N/A |

| - 1. Were data collectors blinded for outcomes assessment? (If outcome is measured using an objective test, such as a lab value, this criterion is assumed to be met.)   2. In cohort study or cross-sectional study, were measurements of outcomes and risk factors blinded?   3. In case control study, was case definition explicit and case ascertainment not influenced by exposure status?   4. In diagnostic study, were test results blinded to patient history and other test results? |  | | | |
| --- | --- | --- | --- | --- |
| 1. **Were intervention/therapeutic regimens/exposure factor or procedure and any comparison(s) described in detail? Were intervening factors described?**    1. In RCT or other intervention trial, were protocols described for all regimens studied?    2. n observational study, were interventions, study settings, and clinicians/provider described?    3. Was the intensity and duration of the intervention or exposure factor sufficient to produce a meaningful effect?    4. Was the amount of exposure and, if relevant, subject/patient compliance measured?    5. Were co-interventions (e.g., ancillary treatments, other therapies) described?    6. Were extra or unplanned treatments described?    7. Was the information for 6.4, 6.5, and 6.6 assessed the same way for all groups?    8. In diagnostic study, were details of test administration and replication sufficient? | **Yes** | No | Unclear | N/A |
| 1. **Were outcomes clearly defined and the measurements valid and reliable?**    1. Were primary and secondary endpoints described and relevant to the question?    2. Were nutrition measures appropriate to question and outcomes of concern?    3. Was the period of follow-up long enough for important outcome(s) to occur?    4. Were the observations and measurements based on standard, valid, and reliable data collection instruments/tests/procedures?    5. Was the measurement of effect at an appropriate level of precision?    6. Were other factors accounted for (measured) that could affect outcomes?    7. Were the measurements conducted consistently across groups? | **Yes** | No | Unclear | N/A |
| 1. **Was the statistical analysis appropriate for the study design and type of outcome indicators?**    1. Were statistical analyses adequately described the results reported appropriately?    2. Were correct statistical tests used and assumptions of test not violated?    3. Were statistics reported with levels of significance and/or confidence intervals?    4. Was “intent to treat” analysis of outcomes done (and as appropriate, was there an analysis of outcomes for those maximally exposed or a dose-response analysis)?    5. Were adequate adjustments made for effects of confounding factors that might have affected the outcomes (e.g., multivariate analyses)?    6. Was clinical significance as well as statistical significance reported?    7. If negative findings, was a power calculation reported to address type 2 error? | **Yes** | No | Unclear | N/A |
| 1. **Are conclusions supported by results with biases and limitations taken into consideration?**    1. Is there a discussion of findings?    2. Are biases and study limitations identified and discussed? | **Yes** | No | Unclear | N/A |
| 1. **Is bias due to study’s funding or sponsorship unlikely?**    1. Were sources of funding and investigators’ affiliations described?    2. Was there no apparent conflict of interest? | **Yes** | No | Unclear | N/A |

| **MINUS/NEGATIVE (-)**  *If most (six or more) of the answers to the above validity questions are “No,” the report should be designated with a minus (-) symbol on the Evidence Worksheet.* |
| --- |
| **NEUTRAL (**∅**)**  *If the answers to validity criteria questions 2, 3, 6, and 7 do not indicate that the study is exceptionally strong, the report should be designated with a neutral* (∅) *symbol on the Evidence Worksheet.* |
| **PLUS/POSITIVE (+)**  *If most of the answers to the above validity questions are “Yes” (including criteria 2, 3, 6, 7 and at least one additional “Yes”), the report should be designated with a plus symbol (+) on the Evidence Worksheet.* |

# Final score: POSITIVE (+)

# Quality Criteria Checklist: Primary Research

**12. Multimodal Digital Phenotyping Study in Patients With Major Depressive Episodes and Healthy Controls (Mobile Monitoring of Mood): Observational Longitudinal Study**

Quality Criteria Checklist: Primary Research

| **RELEVANCE QUESTIONS** | | | | |
| --- | --- | --- | --- | --- |
| 1. Would implementing the studied intervention or procedure (if found successful) result in improved outcomes for the patients/clients/population group? (NA for some Epi studies) | Yes | No | Unclear | N/A |
| 2. Did the authors study an outcome (dependent variable) or topic that the patients/clients/population group would care about? | Yes | No | Unclear | N/A |
| 3. Is the focus of the intervention or procedure (independent variable) or topic of study a common issue of concern to mental health? | Yes | No | Unclear | N/A |
| 4. Is the intervention or procedure feasible? (NA for some epidemiological studies) | Yes | No | Unclear | N/A |
| ***If the answers to all of the above relevance questions are “Yes,” the report is eligible for designation with a plus (+) on the Evidence Quality Worksheet, depending on answers to the following validity questions.*** | | | | |
| **VALIDITY QUESTIONS** | | | | |
| 1. **Was the research question clearly stated?**    1. Was the specific intervention(s) or procedure (independent variable(s)) identified?    2. Was the outcome(s) (dependent variable(s)) clearly indicated?    3. Were the target population and setting specified? | Yes | No | Unclear | N/A |
| 1. **Was the selection of study subjects/patients free from bias?**    1. Were inclusion/exclusion criteria specified (e.g., risk, point in disease progression, diagnostic or prognosis criteria), and with sufficient detail and without omitting criteria critical to the study?    2. Were criteria applied equally to all study groups?    3. Were health, demographics, and other characteristics of subjects described?    4. Were the subjects/patients a representative sample of the relevant population? | Yes | No | Unclear | N/A |
| 1. **Were study groups comparable?**    1. Was the method of assigning subjects/patients to groups described and unbiased? (Method of randomization identified if RCT) No    2. Were distribution of disease status, prognostic factors, and other factors (e.g., demographics) similar across study groups at baseline? Unclear    3. Were concurrent controls used? (Concurrent preferred over historical controls.) Yes    4. If cohort study or cross-sectional study, were groups comparable on important confounding factors and/or were preexisting differences accounted for by using appropriate adjustments in statistical analysis? Yes    5. If case control study, were potential confounding factors comparable for cases and controls? (If case series or trial with subjects serving as own control, this criterion is not applicable. Criterion may not be applicable in some cross-sectional studies.) N/A    6. If diagnostic test, was there an independent blind comparison with an appropriate reference standard (e.g., “gold standard”)? N/A | Yes | No | Unclear | N/A |
| 1. **Was method of handling withdrawals described?**    1. Were follow up methods described and the same for all groups? Yes    2. Was the number, characteristics of withdrawals (i.e., dropouts, lost to follow up, attrition rate) and/or response rate (cross-sectional studies) described for each group? (Follow up goal for a strong study is 80%.) Yes    3. Were all enrolled subjects/patients (in the original sample) accounted for? Yes    4. Were reasons for withdrawals similar across groups? Yes    5. If diagnostic test, was decision to perform reference test not dependent on results of test under study? N/A | Yes | No | Unclear | N/A |
| 1. **Was blinding used to prevent introduction of bias?**    1. In intervention study, were subjects, clinicians/practitioners, and investigators blinded to treatment group, as appropriate? N/A | Yes | No | Unclear | N/A |

| - 1. Were data collectors blinded for outcomes assessment? (If outcome is measured using an objective test, such as a lab value, this criterion is assumed to be met.) Yes   2. In cohort study or cross-sectional study, were measurements of outcomes and risk factors blinded? Yes   3. In case control study, was case definition explicit and case ascertainment not influenced by exposure status? N/A   4. In diagnostic study, were test results blinded to patient history and other test results? N/A |  | | | |
| --- | --- | --- | --- | --- |
| 1. **Were intervention/therapeutic regimens/exposure factor or procedure and any comparison(s) described in detail? Were intervening factors described?**    1. In RCT or other intervention trial, were protocols described for all regimens studied? N/A    2. n observational study, were interventions, study settings, and clinicians/provider described? Yes    3. Was the intensity and duration of the intervention or exposure factor sufficient to produce a meaningful effect? Yes    4. Was the amount of exposure and, if relevant, subject/patient compliance measured? Yes    5. Were co-interventions (e.g., ancillary treatments, other therapies) described? Unclear    6. Were extra or unplanned treatments described? N/A    7. Was the information for 6.4, 6.5, and 6.6 assessed the same way for all groups? Yes    8. In diagnostic study, were details of test administration and replication sufficient? N/A | Yes | No | Unclear | N/A |
| 1. **Were outcomes clearly defined and the measurements valid and reliable?**    1. Were primary and secondary endpoints described and relevant to the question? Yes    2. Were mental health measures appropriate to question and outcomes of concern? Yes    3. Was the period of follow-up long enough for important outcome(s) to occur? Yes    4. Were the observations and measurements based on standard, valid, and reliable data collection instruments/tests/procedures? Yes    5. Was the measurement of effect at an appropriate level of precision? Yes    6. Were other factors accounted for (measured) that could affect outcomes? Yes    7. Were the measurements conducted consistently across groups? Yes | Yes | No | Unclear | N/A |
| 1. **Was the statistical analysis appropriate for the study design and type of outcome indicators?**    1. Were statistical analyses adequately described the results reported appropriately? Yes    2. Were correct statistical tests used and assumptions of test not violated? Yes    3. Were statistics reported with levels of significance and/or confidence intervals? Yes    4. Was “intent to treat” analysis of outcomes done (and as appropriate, was there an analysis of outcomes for those maximally exposed or a dose-response analysis)? N/A    5. Were adequate adjustments made for effects of confounding factors that might have affected the outcomes (e.g., multivariate analyses)? Yes    6. Was clinical significance as well as statistical significance reported? Unclear    7. If negative findings, was a power calculation reported to address type 2 error? Unclear | Yes | No | Unclear | N/A |
| 1. **Are conclusions supported by results with biases and limitations taken into consideration?**    1. Is there a discussion of findings? Yes    2. Are biases and study limitations identified and discussed? Yes | Yes | No | Unclear | N/A |
| 1. **Is bias due to study’s funding or sponsorship unlikely?**    1. Were sources of funding and investigators’ affiliations described? Yes    2. Was there no apparent conflict of interest? Yes | Yes | No | Unclear | N/A |

# Final score: POSITIVE (+)

# Quality Criteria Checklist: Primary Research

**13. Exploring actigraphy as a digital phenotyping measure: A study on differentiating psychomotor agitation and retardation in depression**

Quality Criteria Checklist: Primary Research

| **RELEVANCE QUESTIONS** | | | | |
| --- | --- | --- | --- | --- |
| 1. Would implementing the studied intervention or procedure (if found successful) result in improved outcomes for the patients/clients/population group? (NA for some Epi studies) | Yes | No | Unclear | N/A |
| 2. Did the authors study an outcome (dependent variable) or topic that the patients/clients/population group would care about? | Yes | No | Unclear | N/A |
| 3. Is the focus of the intervention or procedure (independent variable) or topic of study a common issue of concern to mental health? | Yes | No | Unclear | N/A |
| 4. Is the intervention or procedure feasible? (NA for some epidemiological studies) | Yes | No | Unclear | N/A |
| ***If the answers to all of the above relevance questions are “Yes,” the report is eligible for designation with a plus (+) on the Evidence Quality Worksheet, depending on answers to the following validity questions.*** | | | | |
| **VALIDITY QUESTIONS** | | | | |
| 1. **Was the research question clearly stated?**    1. Was the specific intervention(s) or procedure (independent variable(s)) identified? Yes    2. Was the outcome(s) (dependent variable(s)) clearly indicated? Yes    3. Were the target population and setting specified? Yes | Yes | No | Unclear | N/A |
| 1. **Was the selection of study subjects/patients free from bias?**    1. Were inclusion/exclusion criteria specified (e.g., risk, point in disease progression, diagnostic or prognosis criteria), and with sufficient detail and without omitting criteria critical to the study? Yes    2. Were criteria applied equally to all study groups? Yes    3. Were health, demographics, and other characteristics of subjects described? Yes    4. Were the subjects/patients a representative sample of the relevant population? Yes | Yes | No | Unclear | N/A |
| 1. **Were study groups comparable?**    1. Was the method of assigning subjects/patients to groups described and unbiased? (Method of randomization identified if RCT) Yes    2. Were distribution of disease status, prognostic factors, and other factors (e.g., demographics) similar across study groups at baseline? No    3. Were concurrent controls used? (Concurrent preferred over historical controls.) Yes    4. If cohort study or cross-sectional study, were groups comparable on important confounding factors and/or were preexisting differences accounted for by using appropriate adjustments in statistical analysis? Yes    5. If case control study, were potential confounding factors comparable for cases and controls? (If case series or trial with subjects serving as own control, this criterion is not applicable. Criterion may not be applicable in some cross-sectional studies.) N/A    6. If diagnostic test, was there an independent blind comparison with an appropriate reference standard (e.g., “gold standard”)? N/A | Yes | No | Unclear | N/A |
| 1. **Was method of handling withdrawals described?**    1. Were follow up methods described and the same for all groups? Yes    2. Was the number, characteristics of withdrawals (i.e., dropouts, lost to follow up, attrition rate) and/or response rate (cross-sectional studies) described for each group? (Follow up goal for a strong study is 80%.) Yes    3. Were all enrolled subjects/patients (in the original sample) accounted for? Yes    4. Were reasons for withdrawals similar across groups? N/A    5. If diagnostic test, was decision to perform reference test not dependent on results of test under study? N/A | Yes | No | Unclear | N/A |
| 1. **Was blinding used to prevent introduction of bias?**    1. In intervention study, were subjects, clinicians/practitioners, and investigators blinded to treatment group, as appropriate? N/A | Yes | No | Unclear | N/A |

| - 1. Were data collectors blinded for outcomes assessment? (If outcome is measured using an objective test, such as a lab value, this criterion is assumed to be met.) Yes   2. In cohort study or cross-sectional study, were measurements of outcomes and risk factors blinded? Yes   3. In case control study, was case definition explicit and case ascertainment not influenced by exposure status? N/A   4. In diagnostic study, were test results blinded to patient history and other test results? N/A |  | | | |
| --- | --- | --- | --- | --- |
| 1. **Were intervention/therapeutic regimens/exposure factor or procedure and any comparison(s) described in detail? Were intervening factors described?**    1. In RCT or other intervention trial, were protocols described for all regimens studied? N/A    2. n observational study, were interventions, study settings, and clinicians/provider described? Yes    3. Was the intensity and duration of the intervention or exposure factor sufficient to produce a meaningful effect? Yes    4. Was the amount of exposure and, if relevant, subject/patient compliance measured? Yes    5. Were co-interventions (e.g., ancillary treatments, other therapies) described? Yes    6. Were extra or unplanned treatments described? N/A    7. Was the information for 6.4, 6.5, and 6.6 assessed the same way for all groups? Yes    8. In diagnostic study, were details of test administration and replication sufficient? N/A | Yes | No | Unclear | N/A |
| 1. **Were outcomes clearly defined and the measurements valid and reliable?**    1. Were primary and secondary endpoints described and relevant to the question? Yes    2. Were mental health measures appropriate to question and outcomes of concern? Yes    3. Was the period of follow-up long enough for important outcome(s) to occur? Yes    4. Were the observations and measurements based on standard, valid, and reliable data collection instruments/tests/procedures? Yes    5. Was the measurement of effect at an appropriate level of precision? Yes    6. Were other factors accounted for (measured) that could affect outcomes? Yes    7. Were the measurements conducted consistently across groups? Yes | Yes | No | Unclear | N/A |
| 1. **Was the statistical analysis appropriate for the study design and type of outcome indicators?**    1. Were statistical analyses adequately described the results reported appropriately? Yes    2. Were correct statistical tests used and assumptions of test not violated? Yes    3. Were statistics reported with levels of significance and/or confidence intervals? Yes    4. Was “intent to treat” analysis of outcomes done (and as appropriate, was there an analysis of outcomes for those maximally exposed or a dose-response analysis)? N/A    5. Were adequate adjustments made for effects of confounding factors that might have affected the outcomes (e.g., multivariate analyses)? Yes    6. Was clinical significance as well as statistical significance reported? Unclear    7. If negative findings, was a power calculation reported to address type 2 error? Yes | Yes | No | Unclear | N/A |
| 1. **Are conclusions supported by results with biases and limitations taken into consideration?**    1. Is there a discussion of findings? Yes    2. Are biases and study limitations identified and discussed? Yes | Yes | No | Unclear | N/A |
| 1. **Is bias due to study’s funding or sponsorship unlikely?**     1. Were sources of funding and investigators’ affiliations described? Yes    2. Was there no apparent conflict of interest? Yes | Yes | No | Unclear | N/A |

# Final score: POSITIVE (+)

# Quality Criteria Checklist: Primary Research

**14. Using machine learning with intensive longitudinal data to predict depression and suicidal ideation among medical interns over time**

Quality Criteria Checklist: Primary Research

| **RELEVANCE QUESTIONS** | | | | |
| --- | --- | --- | --- | --- |
| 1. Would implementing the studied intervention or procedure (if found successful) result in improved outcomes for the patients/clients/population group? (NA for some Epi studies) | Yes | No | Unclear | N/A |
| 2. Did the authors study an outcome (dependent variable) or topic that the patients/clients/population group would care about? | Yes | No | Unclear | N/A |
| 3. Is the focus of the intervention or procedure (independent variable) or topic of study a common issue of concern to mental health? | Yes | No | Unclear | N/A |
| 4. Is the intervention or procedure feasible? (NA for some epidemiological studies) | Yes | No | Unclear | N/A |
| ***If the answers to all of the above relevance questions are “Yes,” the report is eligible for designation with a plus (+) on the Evidence Quality Worksheet, depending on answers to the following validity questions.*** | | | | |
| **VALIDITY QUESTIONS** | | | | |
| 1. **Was the research question clearly stated?**    1. Was the specific intervention(s) or procedure (independent variable(s)) identified?    2. Was the outcome(s) (dependent variable(s)) clearly indicated?    3. Were the target population and setting specified? | Yes | No | Unclear | N/A |
| 1. **Was the selection of study subjects/patients free from bias?**    1. Were inclusion/exclusion criteria specified (e.g., risk, point in disease progression, diagnostic or prognosis criteria), and with sufficient detail and without omitting criteria critical to the study?    2. Were criteria applied equally to all study groups?    3. Were health, demographics, and other characteristics of subjects described?    4. Were the subjects/patients a representative sample of the relevant population? | Yes | No | Unclear | N/A |
| 1. **Were study groups comparable?**    1. Was the method of assigning subjects/patients to groups described and unbiased? (Method of randomization identified if RCT) Yes    2. Were distribution of disease status, prognostic factors, and other factors (e.g., demographics) similar across study groups at baseline? Yes    3. Were concurrent controls used? (Concurrent preferred over historical controls.) Yes    4. If cohort study or cross-sectional study, were groups comparable on important confounding factors and/or were preexisting differences accounted for by using appropriate adjustments in statistical analysis? Yes    5. If case control study, were potential confounding factors comparable for cases and controls? (If case series or trial with subjects serving as own control, this criterion is not applicable. Criterion may not be applicable in some cross-sectional studies.) N/A    6. If diagnostic test, was there an independent blind comparison with an appropriate reference standard (e.g., “gold standard”)? N/A | Yes | No | Unclear | N/A |
| 1. **Was method of handling withdrawals described?**    1. Were follow up methods described and the same for all groups? Yes    2. Was the number, characteristics of withdrawals (i.e., dropouts, lost to follow up, attrition rate) and/or response rate (cross-sectional studies) described for each group? (Follow up goal for a strong study is 80%.) Yes    3. Were all enrolled subjects/patients (in the original sample) accounted for? Yes    4. Were reasons for withdrawals similar across groups? Unclear    5. If diagnostic test, was decision to perform reference test not dependent on results of test under study? N/A | Yes | No | Unclear | N/A |
| 1. **Was blinding used to prevent introduction of bias?**    1. In intervention study, were subjects, clinicians/practitioners, and investigators blinded to treatment group, as appropriate? N/A | Yes | No | Unclear | N/A |

| - 1. Were data collectors blinded for outcomes assessment? (If outcome is measured using an objective test, such as a lab value, this criterion is assumed to be met.) Yes   2. In cohort study or cross-sectional study, were measurements of outcomes and risk factors blinded? Yes   3. In case control study, was case definition explicit and case ascertainment not influenced by exposure status? N/A   4. In diagnostic study, were test results blinded to patient history and other test results? N/A |  | | | |
| --- | --- | --- | --- | --- |
| 1. **Were intervention/therapeutic regimens/exposure factor or procedure and any comparison(s) described in detail? Were intervening factors described?**    1. In RCT or other intervention trial, were protocols described for all regimens studied? N/A    2. n observational study, were interventions, study settings, and clinicians/provider described? Yes    3. Was the intensity and duration of the intervention or exposure factor sufficient to produce a meaningful effect? Yes    4. Was the amount of exposure and, if relevant, subject/patient compliance measured? Yes    5. Were co-interventions (e.g., ancillary treatments, other therapies) described? N/A    6. Were extra or unplanned treatments described? N/A    7. Was the information for 6.4, 6.5, and 6.6 assessed the same way for all groups? Yes    8. In diagnostic study, were details of test administration and replication sufficient? N/A | Yes | No | Unclear | N/A |
| 1. **Were outcomes clearly defined and the measurements valid and reliable?**    1. Were primary and secondary endpoints described and relevant to the question? Yes    2. Were mental health measures appropriate to question and outcomes of concern? Yes    3. Was the period of follow-up long enough for important outcome(s) to occur? Yes    4. Were the observations and measurements based on standard, valid, and reliable data collection instruments/tests/procedures? Yes    5. Was the measurement of effect at an appropriate level of precision? Yes    6. Were other factors accounted for (measured) that could affect outcomes? Yes    7. Were the measurements conducted consistently across groups? Yes | Yes | No | Unclear | N/A |
| 1. **Was the statistical analysis appropriate for the study design and type of outcome indicators?**    1. Were statistical analyses adequately described the results reported appropriately? Yes    2. Were correct statistical tests used and assumptions of test not violated? Yes    3. Were statistics reported with levels of significance and/or confidence intervals? Yes    4. Was “intent to treat” analysis of outcomes done (and as appropriate, was there an analysis of outcomes for those maximally exposed or a dose-response analysis)? N/A    5. Were adequate adjustments made for effects of confounding factors that might have affected the outcomes (e.g., multivariate analyses)? Yes    6. Was clinical significance as well as statistical significance reported? Unclear    7. If negative findings, was a power calculation reported to address type 2 error? Unclear | Yes | No | Unclear | N/A |
| 1. **Are conclusions supported by results with biases and limitations taken into consideration?**    1. Is there a discussion of findings?    2. Are biases and study limitations identified and discussed? | Yes | No | Unclear | N/A |
| 1. **Is bias due to study’s funding or sponsorship unlikely?**    1. Were sources of funding and investigators’ affiliations described?    2. Was there no apparent conflict of interest? | Yes | No | Unclear | N/A |

# Final score: POSITIVE (+)

# Quality Criteria Checklist: Primary Research

**16. Identifying Objective Physiological Markers and Modifiable Behaviors for Self-Reported Stress and Mental Health Status Using Wearable Sensors and Mobile Phones: Observational Study**

Quality Criteria Checklist: Primary Research

| **RELEVANCE QUESTIONS** | | | | |
| --- | --- | --- | --- | --- |
| 1. Would implementing the studied intervention or procedure (if found successful) result in improved outcomes for the patients/clients/population group? (NA for some Epi studies) | Yes | No | Unclear | N/A |
| 2. Did the authors study an outcome (dependent variable) or topic that the patients/clients/population group would care about? | Yes | No | Unclear | N/A |
| 3. Is the focus of the intervention or procedure (independent variable) or topic of study a common issue of concern to mental health? | Yes | No | Unclear | N/A |
| 4. Is the intervention or procedure feasible? (NA for some epidemiological studies) | Yes | No | Unclear | N/A |
| ***If the answers to all of the above relevance questions are “Yes,” the report is eligible for designation with a plus (+) on the Evidence Quality Worksheet, depending on answers to the following validity questions.*** | | | | |
| **VALIDITY QUESTIONS** | | | | |
| 1. **Was the research question clearly stated?**    1. Was the specific intervention(s) or procedure (independent variable(s)) identified? Yes    2. Was the outcome(s) (dependent variable(s)) clearly indicated? Yes    3. Were the target population and setting specified? Yes | Yes | No | Unclear | N/A |
| 1. **Was the selection of study subjects/patients free from bias?**    1. Were inclusion/exclusion criteria specified (e.g., risk, point in disease progression, diagnostic or prognosis criteria), and with sufficient detail and without omitting criteria critical to the study? Yes    2. Were criteria applied equally to all study groups? Yes    3. Were health, demographics, and other characteristics of subjects described? Yes    4. Were the subjects/patients a representative sample of the relevant population? Unclear | Yes | No | Unclear | N/A  ?? |
| 1. **Were study groups comparable?**    1. Was the method of assigning subjects/patients to groups described and unbiased? (Method of randomization identified if RCT) N/A    2. Were distribution of disease status, prognostic factors, and other factors (e.g., demographics) similar across study groups at baseline? Yes    3. Were concurrent controls used? (Concurrent preferred over historical controls.) N/A    4. If cohort study or cross-sectional study, were groups comparable on important confounding factors and/or were preexisting differences accounted for by using appropriate adjustments in statistical analysis? Yes/Unclear    5. If case control study, were potential confounding factors comparable for cases and controls? (If case series or trial with subjects serving as own control, this criterion is not applicable. Criterion may not be applicable in some cross-sectional studies.) N/A    6. If diagnostic test, was there an independent blind comparison with an appropriate reference standard (e.g., “gold standard”)? N/A | Yes | No | Unclear | N/A |
| 1. **Was method of handling withdrawals described?**    1. Were follow up methods described and the same for all groups? Yes    2. Was the number, characteristics of withdrawals (i.e., dropouts, lost to follow up, attrition rate) and/or response rate (cross-sectional studies) described for each group? (Follow up goal for a strong study is 80%.) Yes    3. Were all enrolled subjects/patients (in the original sample) accounted for? Yes    4. Were reasons for withdrawals similar across groups? Unclear    5. If diagnostic test, was decision to perform reference test not dependent on results of test under study? N/A | Yes | No | Unclear | N/A |
| 1. **Was blinding used to prevent introduction of bias?**    1. In intervention study, were subjects, clinicians/practitioners, and investigators blinded to treatment group, as appropriate? N/A | Yes | No | Unclear | N/A |

| - 1. Were data collectors blinded for outcomes assessment? (If outcome is measured using an objective test, such as a lab value, this criterion is assumed to be met.) Yes   2. In cohort study or cross-sectional study, were measurements of outcomes and risk factors blinded? Yes   3. In case control study, was case definition explicit and case ascertainment not influenced by exposure status? N/A   4. In diagnostic study, were test results blinded to patient history and other test results? N/A |  | | | |
| --- | --- | --- | --- | --- |
| 1. **Were intervention/therapeutic regimens/exposure factor or procedure and any comparison(s) described in detail? Were intervening factors described?**    1. In RCT or other intervention trial, were protocols described for all regimens studied? N/A    2. n observational study, were interventions, study settings, and clinicians/provider described? Yes    3. Was the intensity and duration of the intervention or exposure factor sufficient to produce a meaningful effect? Yes    4. Was the amount of exposure and, if relevant, subject/patient compliance measured? Yes    5. Were co-interventions (e.g., ancillary treatments, other therapies) described? N/A    6. Were extra or unplanned treatments described? N/A    7. Was the information for 6.4, 6.5, and 6.6 assessed the same way for all groups?Yes    8. In diagnostic study, were details of test administration and replication sufficient? N/A | Yes | No | Unclear | N/A |
| 1. **Were outcomes clearly defined and the measurements valid and reliable?**    1. Were primary and secondary endpoints described and relevant to the question? Yes    2. Were mental health measures appropriate to question and outcomes of concern? Yes    3. Was the period of follow-up long enough for important outcome(s) to occur? Yes    4. Were the observations and measurements based on standard, valid, and reliable data collection instruments/tests/procedures? Yes    5. Was the measurement of effect at an appropriate level of precision? Yes    6. Were other factors accounted for (measured) that could affect outcomes? Yes    7. Were the measurements conducted consistently across groups? Yes | Yes | No | Unclear | N/A |
| 1. **Was the statistical analysis appropriate for the study design and type of outcome indicators?**    1. Were statistical analyses adequately described the results reported appropriately? Yes    2. Were correct statistical tests used and assumptions of test not violated? Yes    3. Were statistics reported with levels of significance and/or confidence intervals? Yes    4. Was “intent to treat” analysis of outcomes done (and as appropriate, was there an analysis of outcomes for those maximally exposed or a dose-response analysis)? N/A    5. Were adequate adjustments made for effects of confounding factors that might have affected the outcomes (e.g., multivariate analyses)? Yes    6. Was clinical significance as well as statistical significance reported? Yes    7. If negative findings, was a power calculation reported to address type 2 error? N/A | Yes | No | Unclear | N/A |
| 1. **Are conclusions supported by results with biases and limitations taken into consideration?**    1. Is there a discussion of findings?    2. Are biases and study limitations identified and discussed? | Yes | No | Unclear | N/A |
| 1. **Is bias due to study’s funding or sponsorship unlikely?**    1. Were sources of funding and investigators’ affiliations described?    2. Was there no apparent conflict of interest? | Yes | No | Unclear | N/A |

# Final score: POSITIVE (+)

# Quality Criteria Checklist: Primary Research

**18. Comprehensive Symptom Prediction in Inpatients With Acute Psychiatric Disorders Using Wearable-Based Deep Learning Models: Development and Validation Study**

Quality Criteria Checklist: Primary Research

| **RELEVANCE QUESTIONS** | | | | |
| --- | --- | --- | --- | --- |
| 1. Would implementing the studied intervention or procedure (if found successful) result in improved outcomes for the patients/clients/population group? (NA for some Epi studies) | **Yes** | No | Unclear | N/A |
| 2. Did the authors study an outcome (dependent variable) or topic that the patients/clients/population group would care about? | **Yes** | No | Unclear | N/A |
| 3. Is the focus of the intervention or procedure (independent variable) or topic of study a common issue of concern to mental health? | **Yes** | No | Unclear | N/A |
| 4. Is the intervention or procedure feasible? (NA for some epidemiological studies) | **Yes** | No | Unclear | N/A |
| ***If the answers to all of the above relevance questions are “Yes,” the report is eligible for designation with a plus (+) on the Evidence Quality Worksheet, depending on answers to the following validity questions.*** | | | | |
| **VALIDITY QUESTIONS** | | | | |
| 1. **Was the research question clearly stated?**    1. Was the specific intervention(s) or procedure (independent variable(s)) identified?    2. Was the outcome(s) (dependent variable(s)) clearly indicated?    3. Were the target population and setting specified? | **Yes** | No | Unclear | N/A |
| 1. **Was the selection of study subjects/patients free from bias?**    1. Were inclusion/exclusion criteria specified (e.g., risk, point in disease progression, diagnostic or prognosis criteria), and with sufficient detail and without omitting criteria critical to the study?    2. Were criteria applied equally to all study groups?    3. Were health, demographics, and other characteristics of subjects described?    4. Were the subjects/patients a representative sample of the relevant population? | **Yes** | No | Unclear | N/A |
| 1. **Were study groups comparable?**    1. Was the method of assigning subjects/patients to groups described and unbiased? (Method of randomization identified if RCT)    2. Were distribution of disease status, prognostic factors, and other factors (e.g., demographics) similar across study groups at baseline?    3. Were concurrent controls used? (Concurrent preferred over historical controls.)    4. If cohort study or cross-sectional study, were groups comparable on important confounding factors and/or were preexisting differences accounted for by using appropriate adjustments in statistical analysis?    5. If case control study, were potential confounding factors comparable for cases and controls? (If case series or trial with subjects serving as own control, this criterion is not applicable. Criterion may not be applicable in some cross-sectional studies.)    6. If diagnostic test, was there an independent blind comparison with an appropriate reference standard (e.g., “gold standard”)? | Yes | No | Unclear | **N/A** |
| 1. **Was method of handling withdrawals described?**    1. Were follow up methods described and the same for all groups?    2. Was the number, characteristics of withdrawals (i.e., dropouts, lost to follow up, attrition rate) and/or response rate (cross-sectional studies) described for each group? (Follow up goal for a strong study is 80%.)    3. Were all enrolled subjects/patients (in the original sample) accounted for?    4. Were reasons for withdrawals similar across groups?    5. If diagnostic test, was decision to perform reference test not dependent on results of test under study? | **Yes** | No | Unclear | N/A |
| 1. **Was blinding used to prevent introduction of bias?**    1. In intervention study, were subjects, clinicians/practitioners, and investigators blinded to treatment group, as appropriate? | Yes | No | Unclear | **N/A** |

| - 1. Were data collectors blinded for outcomes assessment? (If outcome is measured using an objective test, such as a lab value, this criterion is assumed to be met.)   2. In cohort study or cross-sectional study, were measurements of outcomes and risk factors blinded?   3. In case control study, was case definition explicit and case ascertainment not influenced by exposure status?   4. In diagnostic study, were test results blinded to patient history and other test results? |  | | | |
| --- | --- | --- | --- | --- |
| 1. **Were intervention/therapeutic regimens/exposure factor or procedure and any comparison(s) described in detail? Were intervening factors described?**    1. In RCT or other intervention trial, were protocols described for all regimens studied?    2. In observational study, were interventions, study settings, and clinicians/provider described?    3. Was the intensity and duration of the intervention or exposure factor sufficient to produce a meaningful effect?    4. Was the amount of exposure and, if relevant, subject/patient compliance measured?    5. Were co-interventions (e.g., ancillary treatments, other therapies) described?    6. Were extra or unplanned treatments described?    7. Was the information for 6.4, 6.5, and 6.6 assessed the same way for all groups?    8. In diagnostic study, were details of test administration and replication sufficient? | **Yes** | No | Unclear | N/A |
| 1. **Were outcomes clearly defined and the measurements valid and reliable?**    1. Were primary and secondary endpoints described and relevant to the question?    2. Were nutrition measures appropriate to question and outcomes of concern?    3. Was the period of follow-up long enough for important outcome(s) to occur?    4. Were the observations and measurements based on standard, valid, and reliable data collection instruments/tests/procedures?    5. Was the measurement of effect at an appropriate level of precision?    6. Were other factors accounted for (measured) that could affect outcomes?    7. Were the measurements conducted consistently across groups? | **Yes** | No | Unclear | N/A |
| 1. **Was the statistical analysis appropriate for the study design and type of outcome indicators?**    1. Were statistical analyses adequately described the results reported appropriately?    2. Were correct statistical tests used and assumptions of test not violated?    3. Were statistics reported with levels of significance and/or confidence intervals?    4. Was “intent to treat” analysis of outcomes done (and as appropriate, was there an analysis of outcomes for those maximally exposed or a dose-response analysis)?    5. Were adequate adjustments made for effects of confounding factors that might have affected the outcomes (e.g., multivariate analyses)?    6. Was clinical significance as well as statistical significance reported?    7. If negative findings, was a power calculation reported to address type 2 error? | **Yes** | No | Unclear | N/A |
| 1. **Are conclusions supported by results with biases and limitations taken into consideration?**    1. Is there a discussion of findings?    2. Are biases and study limitations identified and discussed? | **Yes** | No | Unclear | N/A |
| 1. **Is bias due to study’s funding or sponsorship unlikely?**    1. Were sources of funding and investigators’ affiliations described?    2. Was there no apparent conflict of interest? | **Yes** | No | Unclear | N/A |

# Final score: POSITIVE (+) **(2,6,7 – Yes / 3 – N/A)**

# Quality Criteria Checklist: Primary Research

**21. Investigating the Feasibility of Assessing Depression Severity and Valence-Arousal with Wearable Sensors Using Discrete Wavelet Transforms and Machine Learning**

Quality Criteria Checklist: Primary Research

| **RELEVANCE QUESTIONS** | | | | |
| --- | --- | --- | --- | --- |
| 1. Would implementing the studied intervention or procedure (if found successful) result in improved outcomes for the patients/clients/population group? (NA for some Epi studies) | **Yes** | No | Unclear | N/A |
| 2. Did the authors study an outcome (dependent variable) or topic that the patients/clients/population group would care about? | **Yes** | No | Unclear | N/A |
| 3. Is the focus of the intervention or procedure (independent variable) or topic of study a common issue of concern to mental health? | **Yes** | No | Unclear | N/A |
| 4. Is the intervention or procedure feasible? (NA for some epidemiological studies) | **Yes** | No | Unclear | N/A |
| ***If the answers to all of the above relevance questions are “Yes,” the report is eligible for designation with a plus (+) on the Evidence Quality Worksheet, depending on answers to the following validity questions.*** | | | | |
| **VALIDITY QUESTIONS** | | | | |
| 1. **Was the research question clearly stated?**    1. Was the specific intervention(s) or procedure (independent variable(s)) identified?    2. Was the outcome(s) (dependent variable(s)) clearly indicated?    3. Were the target population and setting specified? | **Yes** | No | Unclear | N/A |
| 1. **Was the selection of study subjects/patients free from bias?**    1. Were inclusion/exclusion criteria specified (e.g., risk, point in disease progression, diagnostic or prognosis criteria), and with sufficient detail and without omitting criteria critical to the study?    2. Were criteria applied equally to all study groups?    3. Were health, demographics, and other characteristics of subjects described?    4. Were the subjects/patients a representative sample of the relevant population? | Yes | **No** | Unclear | N/A |
| 1. **Were study groups comparable?**    1. Was the method of assigning subjects/patients to groups described and unbiased? (Method of randomization identified if RCT)    2. Were distribution of disease status, prognostic factors, and other factors (e.g., demographics) similar across study groups at baseline?    3. Were concurrent controls used? (Concurrent preferred over historical controls.)    4. If cohort study or cross-sectional study, were groups comparable on important confounding factors and/or were preexisting differences accounted for by using appropriate adjustments in statistical analysis?    5. If case control study, were potential confounding factors comparable for cases and controls? (If case series or trial with subjects serving as own control, this criterion is not applicable. Criterion may not be applicable in some cross-sectional studies.)    6. If diagnostic test, was there an independent blind comparison with an appropriate reference standard (e.g., “gold standard”)? | Yes | No | Unclear | **N/A** |
| 1. **Was method of handling withdrawals described?**    1. Were follow up methods described and the same for all groups?    2. Was the number, characteristics of withdrawals (i.e., dropouts, lost to follow up, attrition rate) and/or response rate (cross-sectional studies) described for each group? (Follow up goal for a strong study is 80%.)    3. Were all enrolled subjects/patients (in the original sample) accounted for?    4. Were reasons for withdrawals similar across groups?    5. If diagnostic test, was decision to perform reference test not dependent on results of test under study? | **Yes** | No | Unclear | N/A |
| 1. **Was blinding used to prevent introduction of bias?**    1. In intervention study, were subjects, clinicians/practitioners, and investigators blinded to treatment group, as appropriate? | Yes | No | Unclear | **N/A** |

| - 1. Were data collectors blinded for outcomes assessment? (If outcome is measured using an objective test, such as a lab value, this criterion is assumed to be met.)   2. In cohort study or cross-sectional study, were measurements of outcomes and risk factors blinded?   3. In case control study, was case definition explicit and case ascertainment not influenced by exposure status?   4. In diagnostic study, were test results blinded to patient history and other test results? |  | | | |
| --- | --- | --- | --- | --- |
| 1. **Were intervention/therapeutic regimens/exposure factor or procedure and any comparison(s) described in detail? Were intervening factors described?**    1. In RCT or other intervention trial, were protocols described for all regimens studied?    2. n observational study, were interventions, study settings, and clinicians/provider described?    3. Was the intensity and duration of the intervention or exposure factor sufficient to produce a meaningful effect?    4. Was the amount of exposure and, if relevant, subject/patient compliance measured?    5. Were co-interventions (e.g., ancillary treatments, other therapies) described?    6. Were extra or unplanned treatments described?    7. Was the information for 6.4, 6.5, and 6.6 assessed the same way for all groups?    8. In diagnostic study, were details of test administration and replication sufficient? | **Yes** | No | Unclear | N/A |
| 1. **Were outcomes clearly defined and the measurements valid and reliable?**    1. Were primary and secondary endpoints described and relevant to the question?    2. Were nutrition measures appropriate to question and outcomes of concern?    3. Was the period of follow-up long enough for important outcome(s) to occur?    4. Were the observations and measurements based on standard, valid, and reliable data collection instruments/tests/procedures?    5. Was the measurement of effect at an appropriate level of precision?    6. Were other factors accounted for (measured) that could affect outcomes?    7. Were the measurements conducted consistently across groups? | **Yes** | No | Unclear | N/A |
| 1. **Was the statistical analysis appropriate for the study design and type of outcome indicators?**    1. Were statistical analyses adequately described the results reported appropriately?    2. Were correct statistical tests used and assumptions of test not violated?    3. Were statistics reported with levels of significance and/or confidence intervals?    4. Was “intent to treat” analysis of outcomes done (and as appropriate, was there an analysis of outcomes for those maximally exposed or a dose-response analysis)?    5. Were adequate adjustments made for effects of confounding factors that might have affected the outcomes (e.g., multivariate analyses)?    6. Was clinical significance as well as statistical significance reported?    7. If negative findings, was a power calculation reported to address type 2 error? | **Yes** | No | Unclear | N/A |
| 1. **Are conclusions supported by results with biases and limitations taken into consideration?**    1. Is there a discussion of findings?    2. Are biases and study limitations identified and discussed? | **Yes** | No | Unclear | N/A |
| 1. **Is bias due to study’s funding or sponsorship unlikely?**    1. Were sources of funding and investigators’ affiliations described?    2. Was there no apparent conflict of interest? | **Yes** | No | Unclear | N/A |

**PLUS/POSITIVE (+) 이지만 NEUTRAL (**∅**)로 볼 수도 있음**

**(2 – No / 3 – N/A / 6, 7 – Yes / 1,4,8,9,10 - Yes)**

# Quality Criteria Checklist: Primary Research

**24. Using digital phenotyping to capture depression symptom variability: detecting naturalistic variability in depression symptoms across one year using passively collected wearable movement and sleep data,
Price et al(2023)**

Quality Criteria Checklist: Primary Research

| **RELEVANCE QUESTIONS** | | | | |
| --- | --- | --- | --- | --- |
| 1. Would implementing the studied intervention or procedure (if found successful) result in improved outcomes for the patients/clients/population group? (NA for some Epi studies) | Yes | No | Unclear | N/A |
| 2. Did the authors study an outcome (dependent variable) or topic that the patients/clients/population group would care about? | Yes | No | Unclear | N/A |
| 3. Is the focus of the intervention or procedure (independent variable) or topic of study a common issue of concern to mental health practice? | Yes | No | Unclear | N/A |
| 4. Is the intervention or procedure feasible? (NA for some epidemiological studies) | Yes | No | Unclear | N/A |
| ***If the answers to all of the above relevance questions are “Yes,” the report is eligible for designation with a plus (+) on the Evidence Quality Worksheet, depending on answers to the following validity questions.*** | | | | |
| **VALIDITY QUESTIONS** | | | | |
| 1. **Was the research question clearly stated?**    1. Was the specific intervention(s) or procedure (independent variable(s)) identified?    2. Was the outcome(s) (dependent variable(s)) clearly indicated?    3. Were the target population and setting specified? | Yes | No | Unclear | N/A |
| 1. **Was the selection of study subjects/patients free from bias?**    1. Were inclusion/exclusion criteria specified (e.g., risk, point in disease progression, diagnostic or prognosis criteria), and with sufficient detail and without omitting criteria critical to the study?    2. Were criteria applied equally to all study groups?    3. Were health, demographics, and other characteristics of subjects described?    4. Were the subjects/patients a representative sample of the relevant population? | Yes | No | Unclear | N/A |
| 1. **Were study groups comparable?**    1. Was the method of assigning subjects/patients to groups described and unbiased? (Method of randomization identified if RCT)    2. Were distribution of disease status, prognostic factors, and other factors (e.g., demographics) similar across study groups at baseline?    3. Were concurrent controls used? (Concurrent preferred over historical controls.)    4. If cohort study or cross-sectional study, were groups comparable on important confounding factors and/or were preexisting differences accounted for by using appropriate adjustments in statistical analysis?    5. If case control study, were potential confounding factors comparable for cases and controls? (If case series or trial with subjects serving as own control, this criterion is not applicable. Criterion may not be applicable in some cross-sectional studies.)    6. If diagnostic test, was there an independent blind comparison with an appropriate reference standard (e.g., “gold standard”)? | Yes | No | Unclear | N/A |
| 1. **Was method of handling withdrawals described?**    1. Were follow up methods described and the same for all groups?    2. Was the number, characteristics of withdrawals (i.e., dropouts, lost to follow up, attrition rate) and/or response rate (cross-sectional studies) described for each group? (Follow up goal for a strong study is 80%.)    3. Were all enrolled subjects/patients (in the original sample) accounted for?    4. Were reasons for withdrawals similar across groups?    5. If diagnostic test, was decision to perform reference test not dependent on results of test under study? | Yes | No | Unclear | N/A |
| 1. **Was blinding used to prevent introduction of bias?**    1. In intervention study, were subjects, clinicians/practitioners, and investigators blinded to treatment group, as appropriate?    2. Were data collectors blinded for outcomes assessment? (If outcome is measured using an objective test, such as a lab value, this criterion is assumed to be met.)    3. In cohort study or cross-sectional study, were measurements of outcomes and risk factors blinded?    4. In case control study, was case definition explicit and case ascertainment not influenced by exposure status?    5. In diagnostic study, were test results blinded to patient history and other test results? | Yes | No | Unclear | N/A |
| 1. **Were intervention/therapeutic regimens/exposure factor or procedure and any comparison(s) described in detail? Were intervening factors described?**    1. In RCT or other intervention trial, were protocols described for all regimens studied?    2. In observational study, were interventions, study settings, and clinicians/provider described?    3. Was the intensity and duration of the intervention or exposure factor sufficient to produce a meaningful effect?    4. Was the amount of exposure and, if relevant, subject/patient compliance measured?    5. Were co-interventions (e.g., ancillary treatments, other therapies) described?    6. Were extra or unplanned treatments described?    7. Was the information for 6.4, 6.5, and 6.6 assessed the same way for all groups?    8. In diagnostic study, were details of test administration and replication sufficient? | Yes | No | Unclear | N/A |
| 1. **Were outcomes clearly defined and the measurements valid and reliable?**    1. Were primary and secondary endpoints described and relevant to the question?    2. Were nutrition measures appropriate to question and outcomes of concern?    3. Was the period of follow-up long enough for important outcome(s) to occur?    4. Were the observations and measurements based on standard, valid, and reliable data collection instruments/tests/procedures?    5. Was the measurement of effect at an appropriate level of precision?    6. Were other factors accounted for (measured) that could affect outcomes?    7. Were the measurements conducted consistently across groups? | Yes | No | Unclear | N/A |
| 1. **Was the statistical analysis appropriate for the study design and type of outcome indicators?**    1. Were statistical analyses adequately described the results reported appropriately?    2. Were correct statistical tests used and assumptions of test not violated?    3. Were statistics reported with levels of significance and/or confidence intervals?    4. Was “intent to treat” analysis of outcomes done (and as appropriate, was there an analysis of outcomes for those maximally exposed or a dose-response analysis)?    5. Were adequate adjustments made for effects of confounding factors that might have affected the outcomes (e.g., multivariate analyses)?    6. Was clinical significance as well as statistical significance reported?    7. If negative findings, was a power calculation reported to address type 2 error? | Yes | No | Unclear | N/A |
| 1. **Are conclusions supported by results with biases and limitations taken into consideration?**    1. Is there a discussion of findings?    2. Are biases and study limitations identified and discussed? | Yes | No | Unclear | N/A |
| 1. **Is bias due to study’s funding or sponsorship unlikely?**    1. Were sources of funding and investigators’ affiliations described?    2. Was there no apparent conflict of interest? | Yes | No | Unclear | N/A |

# Final score: POSITIVE (+)

# Quality Criteria Checklist: Primary Research

**26. Tracking and Monitoring Mood Stability of Patients With Major Depressive Disorder by Machine Learning Models Using Passive Digital Data: Prospective Naturalistic Multicenter Study, Ran(2021)**

Quality Criteria Checklist: Primary Research

| **RELEVANCE QUESTIONS** | | | |  |
| --- | --- | --- | --- | --- |
| 1. Would implementing the studied intervention or procedure (if found successful) result in improved outcomes for the patients/clients/population group? (NA for some Epi studies) | Yes | No | Unclear | N/A |
| 2. Did the authors study an outcome (dependent variable) or topic that the patients/clients/population group would care about? | Yes | No | Unclear | N/A |
| 3. Is the focus of the intervention or procedure (independent variable) or topic of study a common issue of concern to mental health practice? | Yes | No | Unclear | N/A |
| 4. Is the intervention or procedure feasible? (NA for some epidemiological studies) | Yes | No | Unclear | N/A |
| ***If the answers to all of the above relevance questions are “Yes,” the report is eligible for designation with a plus (+) on the Evidence Quality Worksheet, depending on answers to the following validity questions.*** | | | |  |
| **VALIDITY QUESTIONS** | | | |  |
| 1. **Was the research question clearly stated?**    1. Was the specific intervention(s) or procedure (independent variable(s)) identified?    2. Was the outcome(s) (dependent variable(s)) clearly indicated?    3. Were the target population and setting specified? | Yes | No | Unclear | N/A |
| 1. **Was the selection of study subjects/patients free from bias?**    1. Were inclusion/exclusion criteria specified (e.g., risk, point in disease progression, diagnostic or prognosis criteria), and with sufficient detail and without omitting criteria critical to the study?    2. Were criteria applied equally to all study groups?    3. Were health, demographics, and other characteristics of subjects described?    4. Were the subjects/patients a representative sample of the relevant population? | Yes | No | Unclear | N/A |
| 1. **Were study groups comparable?**    1. Was the method of assigning subjects/patients to groups described and unbiased? (Method of randomization identified if RCT)    2. Were distribution of disease status, prognostic factors, and other factors (e.g., demographics) similar across study groups at baseline?    3. Were concurrent controls used? (Concurrent preferred over historical controls.)    4. If cohort study or cross-sectional study, were groups comparable on important confounding factors and/or were preexisting differences accounted for by using appropriate adjustments in statistical analysis?    5. If case control study, were potential confounding factors comparable for cases and controls? (If case series or trial with subjects serving as own control, this criterion is not applicable. Criterion may not be applicable in some cross-sectional studies.)    6. If diagnostic test, was there an independent blind comparison with an appropriate reference standard (e.g., “gold standard”)? | Yes | No | Unclear | N/A |
| 1. **Was method of handling withdrawals described?**    1. Were follow up methods described and the same for all groups?    2. Was the number, characteristics of withdrawals (i.e., dropouts, lost to follow up, attrition rate) and/or response rate (cross-sectional studies) described for each group? (Follow up goal for a strong study is 80%.)    3. Were all enrolled subjects/patients (in the original sample) accounted for?    4. Were reasons for withdrawals similar across groups?    5. If diagnostic test, was decision to perform reference test not dependent on results of test under study? | Yes | No | Unclear | N/A |
| 1. **Was blinding used to prevent introduction of bias?**    1. In intervention study, were subjects, clinicians/practitioners, and investigators blinded to treatment group, as appropriate?    2. Were data collectors blinded for outcomes assessment? (If outcome is measured using an objective test, such as a lab value, this criterion is assumed to be met.)    3. In cohort study or cross-sectional study, were measurements of outcomes and risk factors blinded?    4. In case control study, was case definition explicit and case ascertainment not influenced by exposure status?    5. In diagnostic study, were test results blinded to patient history and other test results? | Yes | No | Unclear | N/A |
| 1. **Were intervention/therapeutic regimens/exposure factor or procedure and any comparison(s) described in detail? Were intervening factors described?**    1. In RCT or other intervention trial, were protocols described for all regimens studied?    2. In observational study, were interventions, study settings, and clinicians/provider described?    3. Was the intensity and duration of the intervention or exposure factor sufficient to produce a meaningful effect?    4. Was the amount of exposure and, if relevant, subject/patient compliance measured?    5. Were co-interventions (e.g., ancillary treatments, other therapies) described?    6. Were extra or unplanned treatments described?    7. Was the information for 6.4, 6.5, and 6.6 assessed the same way for all groups?    8. In diagnostic study, were details of test administration and replication sufficient? | Yes | No | Unclear | N/A |
| 1. **Were outcomes clearly defined and the measurements valid and reliable?**    1. Were primary and secondary endpoints described and relevant to the question?    2. Were nutrition measures appropriate to question and outcomes of concern?    3. Was the period of follow-up long enough for important outcome(s) to occur?    4. Were the observations and measurements based on standard, valid, and reliable data collection instruments/tests/procedures?    5. Was the measurement of effect at an appropriate level of precision?    6. Were other factors accounted for (measured) that could affect outcomes?    7. Were the measurements conducted consistently across groups? | Yes | No | Unclear | N/A |
| 1. **Was the statistical analysis appropriate for the study design and type of outcome indicators?**    1. Were statistical analyses adequately described the results reported appropriately?    2. Were correct statistical tests used and assumptions of test not violated?    3. Were statistics reported with levels of significance and/or confidence intervals?    4. Was “intent to treat” analysis of outcomes done (and as appropriate, was there an analysis of outcomes for those maximally exposed or a dose-response analysis)?    5. Were adequate adjustments made for effects of confounding factors that might have affected the outcomes (e.g., multivariate analyses)?    6. Was clinical significance as well as statistical significance reported?    7. If negative findings, was a power calculation reported to address type 2 error? | Yes | No | Unclear | N/A |
| 1. **Are conclusions supported by results with biases and limitations taken into consideration?**    1. Is there a discussion of findings?    2. Are biases and study limitations identified and discussed? | Yes | No | Unclear | N/A |
| 1. **Is bias due to study’s funding or sponsorship unlikely?**    1. Were sources of funding and investigators’ affiliations described?    2. Was there no apparent conflict of interest? | Yes | No | Unclear | N/A |

# Final score: POSITIVE (+)

# Quality Criteria Checklist: Primary Research

# ***27.* Sensor-Assisted Weighted Average Ensemble Model for Detecting Major Depressive Disorder” (Sensors, 2019)**

Quality Criteria Checklist: Primary Research

| **RELEVANCE QUESTIONS** | | | | |
| --- | --- | --- | --- | --- |
| 1. Would implementing the studied intervention or procedure (if found successful) result in improved outcomes for the patients/clients/population group? (NA for some Epi studies) | **Yes** | No | Unclear | N/A |
| 2. Did the authors study an outcome (dependent variable) or topic that the patients/clients/population group would care about? | **Yes** | No | Unclear | N/A |
| 3. Is the focus of the intervention or procedure (independent variable) or topic of study a common issue of concern to mental healthcare? | **Yes** | No | Unclear | N/A |
| 4. Is the intervention or procedure feasible? (NA for some epidemiological studies) | **Yes** | No | Unclear | N/A |
| ***If the answers to all of the above relevance questions are “Yes,” the report is eligible for designation with a plus (+) on the Evidence Quality Worksheet, depending on answers to the following validity questions.*** | | | | |
| **VALIDITY QUESTIONS** | | | | |
| 1. **Was the research question clearly stated?**    1. Was the specific intervention(s) or procedure (independent variable(s)) identified?    2. Was the outcome(s) (dependent variable(s)) clearly indicated?    3. Were the target population and setting specified? | **Yes** | No | Unclear | N/A |
| 1. **Was the selection of study subjects/patients free from bias?**    1. Were inclusion/exclusion criteria specified (e.g., risk, point in disease progression, diagnostic or prognosis criteria), and with sufficient detail and without omitting criteria critical to the study?    2. Were criteria applied equally to all study groups?    3. Were health, demographics, and other characteristics of subjects described?    4. Were the subjects/patients a representative sample of the relevant population? | Yes | No | **Unclear** | N/A |
| 1. **Were study groups comparable?**    1. Was the method of assigning subjects/patients to groups described and unbiased? (Method of randomization identified if RCT)    2. Were distribution of disease status, prognostic factors, and other factors (e.g., demographics) similar across study groups at baseline?    3. Were concurrent controls used? (Concurrent preferred over historical controls.)    4. If cohort study or cross-sectional study, were groups comparable on important confounding factors and/or were preexisting differences accounted for by using appropriate adjustments in statistical analysis?    5. If case control study, were potential confounding factors comparable for cases and controls? (If case series or trial with subjects serving as own control, this criterion is not applicable. Criterion may not be applicable in some cross-sectional studies.)    6. If diagnostic test, was there an independent blind comparison with an appropriate reference standard (e.g., “gold standard”)? | Yes | No | Unclear | **N/A** |
| 1. **Was method of handling withdrawals described?**    1. Were follow up methods described and the same for all groups?    2. Was the number, characteristics of withdrawals (i.e., dropouts, lost to follow up, attrition rate) and/or response rate (cross-sectional studies) described for each group? (Follow up goal for a strong study is 80%.)    3. Were all enrolled subjects/patients (in the original sample) accounted for?    4. Were reasons for withdrawals similar across groups?    5. If diagnostic test, was decision to perform reference test not dependent on results of test under study? | Yes | **No** | Unclear | N/A |
| 1. **Was blinding used to prevent introduction of bias?**    1. In intervention study, were subjects, clinicians/practitioners, and investigators blinded to treatment group, as appropriate? | Yes | No | Unclear | **N/A** |

| - 1. Were data collectors blinded for outcomes assessment? (If outcome is measured using an objective test, such as a lab value, this criterion is assumed to be met.)   2. In cohort study or cross-sectional study, were measurements of outcomes and risk factors blinded?   3. In case control study, was case definition explicit and case ascertainment not influenced by exposure status?   4. In diagnostic study, were test results blinded to patient history and other test results? |  | | | |
| --- | --- | --- | --- | --- |
| 1. **Were intervention/therapeutic regimens/exposure factor or procedure and any comparison(s) described in detail? Were intervening factors described?**    1. In RCT or other intervention trial, were protocols described for all regimens studied?    2. In observational study, were interventions, study settings, and clinicians/provider described?    3. Was the intensity and duration of the intervention or exposure factor sufficient to produce a meaningful effect?    4. Was the amount of exposure and, if relevant, subject/patient compliance measured?    5. Were co-interventions (e.g., ancillary treatments, other therapies) described?    6. Were extra or unplanned treatments described?    7. Was the information for 6.4, 6.5, and 6.6 assessed the same way for all groups?    8. In diagnostic study, were details of test administration and replication sufficient? | **Yes** | No | Unclear | N/A |
| 1. **Were outcomes clearly defined and the measurements valid and reliable?**    1. Were primary and secondary endpoints described and relevant to the question?    2. Were nutrition measures appropriate to question and outcomes of concern?    3. Was the period of follow-up long enough for important outcome(s) to occur?    4. Were the observations and measurements based on standard, valid, and reliable data collection instruments/tests/procedures?    5. Was the measurement of effect at an appropriate level of precision?    6. Were other factors accounted for (measured) that could affect outcomes?    7. Were the measurements conducted consistently across groups? | **Yes** | No | Unclear | N/A |
| 1. **Was the statistical analysis appropriate for the study design and type of outcome indicators?**    1. Were statistical analyses adequately described the results reported appropriately?    2. Were correct statistical tests used and assumptions of test not violated?    3. Were statistics reported with levels of significance and/or confidence intervals?    4. Was “intent to treat” analysis of outcomes done (and as appropriate, was there an analysis of outcomes for those maximally exposed or a dose-response analysis)?    5. Were adequate adjustments made for effects of confounding factors that might have affected the outcomes (e.g., multivariate analyses)?    6. Was clinical significance as well as statistical significance reported?    7. If negative findings, was a power calculation reported to address type 2 error? | **Yes** | No | Unclear | N/A |
| 1. **Are conclusions supported by results with biases and limitations taken into consideration?**    1. Is there a discussion of findings?    2. Are biases and study limitations identified and discussed? | **Yes** | No | Unclear | N/A |
| 1. **Is bias due to study’s funding or sponsorship unlikely?**    1. Were sources of funding and investigators’ affiliations described?    2. Was there no apparent conflict of interest? | **Yes** | No | Unclear | N/A |

# Final score: POSITIVE (+)

# Quality Criteria Checklist: Primary Research

**28. “Effectiveness of a Smartphone App With a Wearable Activity Tracker in Preventing the Recurrence of Mood Disorders” (JMIR Mental Health, 2020)**

Quality Criteria Checklist: Primary Research

| **RELEVANCE QUESTIONS** | | | | |
| --- | --- | --- | --- | --- |
| 1. Would implementing the studied intervention or procedure (if found successful) result in improved outcomes for the patients/clients/population group? (NA for some Epi studies) | **Yes** | No | Unclear | N/A |
| 2. Did the authors study an outcome (dependent variable) or topic that the patients/clients/population group would care about? | **Yes** | No | Unclear | N/A |
| 3. Is the focus of the intervention or procedure (independent variable) or topic of study a common issue of concern to mental health practice? | **Yes** | No | Unclear | N/A |
| 4. Is the intervention or procedure feasible? (NA for some epidemiological studies) | **Yes** | No | Unclear | N/A |
| ***If the answers to all of the above relevance questions are “Yes,” the report is eligible for designation with a plus (+) on the Evidence Quality Worksheet, depending on answers to the following validity questions.*** | | | | |
| **VALIDITY QUESTIONS** | | | | |
| 1. **Was the research question clearly stated?**    1. Was the specific intervention(s) or procedure (independent variable(s)) identified?    2. Was the outcome(s) (dependent variable(s)) clearly indicated?    3. Were the target population and setting specified? | **Yes** | No | Unclear | N/A |
| 1. **Was the selection of study subjects/patients free from bias?**    1. Were inclusion/exclusion criteria specified (e.g., risk, point in disease progression, diagnostic or prognosis criteria), and with sufficient detail and without omitting criteria critical to the study?    2. Were criteria applied equally to all study groups?    3. Were health, demographics, and other characteristics of subjects described?    4. Were the subjects/patients a representative sample of the relevant population? | Yes | No | **Unclear** | N/A |
| 1. **Were study groups comparable?**    1. Was the method of assigning subjects/patients to groups described and unbiased? (Method of randomization identified if RCT)    2. Were distribution of disease status, prognostic factors, and other factors (e.g., demographics) similar across study groups at baseline?    3. Were concurrent controls used? (Concurrent preferred over historical controls.)    4. If cohort study or cross-sectional study, were groups comparable on important confounding factors and/or were preexisting differences accounted for by using appropriate adjustments in statistical analysis?    5. If case control study, were potential confounding factors comparable for cases and controls? (If case series or trial with subjects serving as own control, this criterion is not applicable. Criterion may not be applicable in some cross-sectional studies.)    6. If diagnostic test, was there an independent blind comparison with an appropriate reference standard (e.g., “gold standard”)? | Yes | **No** | Unclear | N/A |
| 1. **Was method of handling withdrawals described?**    1. Were follow up methods described and the same for all groups?    2. Was the number, characteristics of withdrawals (i.e., dropouts, lost to follow up, attrition rate) and/or response rate (cross-sectional studies) described for each group? (Follow up goal for a strong study is 80%.)    3. Were all enrolled subjects/patients (in the original sample) accounted for?    4. Were reasons for withdrawals similar across groups?    5. If diagnostic test, was decision to perform reference test not dependent on results of test under study? | **Yes** | No | Unclear | N/A |
| 1. **Was blinding used to prevent introduction of bias?**    1. In intervention study, were subjects, clinicians/practitioners, and investigators blinded to treatment group, as appropriate? | Yes | No | Unclear | **N/A** |

| - 1. Were data collectors blinded for outcomes assessment? (If outcome is measured using an objective test, such as a lab value, this criterion is assumed to be met.)   2. In cohort study or cross-sectional study, were measurements of outcomes and risk factors blinded?   3. In case control study, was case definition explicit and case ascertainment not influenced by exposure status?   4. In diagnostic study, were test results blinded to patient history and other test results? |  | | | |
| --- | --- | --- | --- | --- |
| 1. **Were intervention/therapeutic regimens/exposure factor or procedure and any comparison(s) described in detail? Were intervening factors described?**    1. In RCT or other intervention trial, were protocols described for all regimens studied?    2. In observational study, were interventions, study settings, and clinicians/provider described?    3. Was the intensity and duration of the intervention or exposure factor sufficient to produce a meaningful effect?    4. Was the amount of exposure and, if relevant, subject/patient compliance measured?    5. Were co-interventions (e.g., ancillary treatments, other therapies) described?    6. Were extra or unplanned treatments described?    7. Was the information for 6.4, 6.5, and 6.6 assessed the same way for all groups?    8. In diagnostic study, were details of test administration and replication sufficient? | **Yes** | No | Unclear | N/A |
| 1. **Were outcomes clearly defined and the measurements valid and reliable?**    1. Were primary and secondary endpoints described and relevant to the question?    2. Were nutrition measures appropriate to question and outcomes of concern?    3. Was the period of follow-up long enough for important outcome(s) to occur?    4. Were the observations and measurements based on standard, valid, and reliable data collection instruments/tests/procedures?    5. Was the measurement of effect at an appropriate level of precision?    6. Were other factors accounted for (measured) that could affect outcomes?    7. Were the measurements conducted consistently across groups? | **Yes** | No | Unclear | N/A |
| 1. **Was the statistical analysis appropriate for the study design and type of outcome indicators?**    1. Were statistical analyses adequately described the results reported appropriately?    2. Were correct statistical tests used and assumptions of test not violated?    3. Were statistics reported with levels of significance and/or confidence intervals?    4. Was “intent to treat” analysis of outcomes done (and as appropriate, was there an analysis of outcomes for those maximally exposed or a dose-response analysis)?    5. Were adequate adjustments made for effects of confounding factors that might have affected the outcomes (e.g., multivariate analyses)?    6. Was clinical significance as well as statistical significance reported?    7. If negative findings, was a power calculation reported to address type 2 error? | **Yes** | No | Unclear | N/A |
| 1. **Are conclusions supported by results with biases and limitations taken into consideration?**    1. Is there a discussion of findings?    2. Are biases and study limitations identified and discussed? | **Yes** | No | Unclear | N/A |
| 1. **Is bias due to study’s funding or sponsorship unlikely?**    1. Were sources of funding and investigators’ affiliations described?    2. Was there no apparent conflict of interest? | **Yes** | No | Unclear | N/A |

# Final score: POSITIVE (+)

# Quality Criteria Checklist: Primary Research

**29. “Mood Prediction of Patients With Mood Disorders by Machine Learning Using Passive Digital Phenotypes Based on the Circadian Rhythm” (JMIR Mental Health, 2019)**

Quality Criteria Checklist: Primary Research

| **RELEVANCE QUESTIONS** | | | | |
| --- | --- | --- | --- | --- |
| 1. Would implementing the studied intervention or procedure (if found successful) result in improved outcomes for the patients/clients/population group? (NA for some Epi studies) | **Yes** | No | Unclear | N/A |
| 2. Did the authors study an outcome (dependent variable) or topic that the patients/clients/population group would care about? | **Yes** | No | Unclear | N/A |
| 3. Is the focus of the intervention or procedure (independent variable) or topic of study a common issue of concern to mental health practice? | **Yes** | No | Unclear | N/A |
| 4. Is the intervention or procedure feasible? (NA for some epidemiological studies) | **Yes** | No | Unclear | N/A |
| ***If the answers to all of the above relevance questions are “Yes,” the report is eligible for designation with a plus (+) on the Evidence Quality Worksheet, depending on answers to the following validity questions.*** | | | | |
| **VALIDITY QUESTIONS** | | | | |
| 1. **Was the research question clearly stated?**    1. Was the specific intervention(s) or procedure (independent variable(s)) identified?    2. Was the outcome(s) (dependent variable(s)) clearly indicated?    3. Were the target population and setting specified? | **Yes** | No | Unclear | N/A |
| 1. **Was the selection of study subjects/patients free from bias?**    1. Were inclusion/exclusion criteria specified (e.g., risk, point in disease progression, diagnostic or prognosis criteria), and with sufficient detail and without omitting criteria critical to the study?    2. Were criteria applied equally to all study groups?    3. Were health, demographics, and other characteristics of subjects described?    4. Were the subjects/patients a representative sample of the relevant population? | **Yes** | No | Unclear | N/A |
| 1. **Were study groups comparable?**    1. Was the method of assigning subjects/patients to groups described and unbiased? (Method of randomization identified if RCT)    2. Were distribution of disease status, prognostic factors, and other factors (e.g., demographics) similar across study groups at baseline?    3. Were concurrent controls used? (Concurrent preferred over historical controls.)    4. If cohort study or cross-sectional study, were groups comparable on important confounding factors and/or were preexisting differences accounted for by using appropriate adjustments in statistical analysis?    5. If case control study, were potential confounding factors comparable for cases and controls? (If case series or trial with subjects serving as own control, this criterion is not applicable. Criterion may not be applicable in some cross-sectional studies.)    6. If diagnostic test, was there an independent blind comparison with an appropriate reference standard (e.g., “gold standard”)? | Yes | No | Unclear | **N/A** |
| 1. **Was method of handling withdrawals described?**    1. Were follow up methods described and the same for all groups?    2. Was the number, characteristics of withdrawals (i.e., dropouts, lost to follow up, attrition rate) and/or response rate (cross-sectional studies) described for each group? (Follow up goal for a strong study is 80%.)    3. Were all enrolled subjects/patients (in the original sample) accounted for?    4. Were reasons for withdrawals similar across groups?    5. If diagnostic test, was decision to perform reference test not dependent on results of test under study? | **Yes** | No | Unclear | N/A |
| 1. **Was blinding used to prevent introduction of bias?**    1. In intervention study, were subjects, clinicians/practitioners, and investigators blinded to treatment group, as appropriate? | Yes | No | Unclear | **N/A** |

| - 1. Were data collectors blinded for outcomes assessment? (If outcome is measured using an objective test, such as a lab value, this criterion is assumed to be met.)   2. In cohort study or cross-sectional study, were measurements of outcomes and risk factors blinded?   3. In case control study, was case definition explicit and case ascertainment not influenced by exposure status?   4. In diagnostic study, were test results blinded to patient history and other test results? |  | | | |
| --- | --- | --- | --- | --- |
| 1. **Were intervention/therapeutic regimens/exposure factor or procedure and any comparison(s) described in detail? Were intervening factors described?**    1. In RCT or other intervention trial, were protocols described for all regimens studied?    2. In observational study, were interventions, study settings, and clinicians/provider described?    3. Was the intensity and duration of the intervention or exposure factor sufficient to produce a meaningful effect?    4. Was the amount of exposure and, if relevant, subject/patient compliance measured?    5. Were co-interventions (e.g., ancillary treatments, other therapies) described?    6. Were extra or unplanned treatments described?    7. Was the information for 6.4, 6.5, and 6.6 assessed the same way for all groups?    8. In diagnostic study, were details of test administration and replication sufficient? | **Yes** | No | Unclear | N/A |
| 1. **Were outcomes clearly defined and the measurements valid and reliable?**    1. Were primary and secondary endpoints described and relevant to the question?    2. Were nutrition measures appropriate to question and outcomes of concern?    3. Was the period of follow-up long enough for important outcome(s) to occur?    4. Were the observations and measurements based on standard, valid, and reliable data collection instruments/tests/procedures?    5. Was the measurement of effect at an appropriate level of precision?    6. Were other factors accounted for (measured) that could affect outcomes?    7. Were the measurements conducted consistently across groups? | **Yes** | No | Unclear | N/A |
| 1. **Was the statistical analysis appropriate for the study design and type of outcome indicators?**    1. Were statistical analyses adequately described the results reported appropriately?    2. Were correct statistical tests used and assumptions of test not violated?    3. Were statistics reported with levels of significance and/or confidence intervals?    4. Was “intent to treat” analysis of outcomes done (and as appropriate, was there an analysis of outcomes for those maximally exposed or a dose-response analysis)?    5. Were adequate adjustments made for effects of confounding factors that might have affected the outcomes (e.g., multivariate analyses)?    6. Was clinical significance as well as statistical significance reported?    7. If negative findings, was a power calculation reported to address type 2 error? | **Yes** | No | Unclear | N/A |
| 1. **Are conclusions supported by results with biases and limitations taken into consideration?**    1. Is there a discussion of findings?    2. Are biases and study limitations identified and discussed? | **Yes** | No | Unclear | N/A |
| 1. **Is bias due to study’s funding or sponsorship unlikely?**    1. Were sources of funding and investigators’ affiliations described?    2. Was there no apparent conflict of interest? | **Yes** | No | Unclear | N/A |

# Final score: POSITIVE (+)

# Quality Criteria Checklist: Primary Research

# **30. "Digital biomarkers of mood disorders and symptom change" (npj Digital Medicine, 2019)**

Quality Criteria Checklist: Primary Research

| **RELEVANCE QUESTIONS** | | | | |
| --- | --- | --- | --- | --- |
| 1. Would implementing the studied intervention or procedure (if found successful) result in improved outcomes for the patients/clients/population group? (NA for some Epi studies) | **Yes** | No | Unclear | N/A |
| 2. Did the authors study an outcome (dependent variable) or topic that the patients/clients/population group would care about? | **Yes** | No | Unclear | N/A |
| 3. Is the focus of the intervention or procedure (independent variable) or topic of study a common issue of concern to mental healthcare? | **Yes** | No | Unclear | N/A |
| 4. Is the intervention or procedure feasible? (NA for some epidemiological studies) | **Yes** | No | Unclear | N/A |
| ***If the answers to all of the above relevance questions are “Yes,” the report is eligible for designation with a plus (+) on the Evidence Quality Worksheet, depending on answers to the following validity questions.*** | | | | |
| **VALIDITY QUESTIONS** | | | | |
| 1. **Was the research question clearly stated?**    1. Was the specific intervention(s) or procedure (independent variable(s)) identified?    2. Was the outcome(s) (dependent variable(s)) clearly indicated?    3. Were the target population and setting specified? | **Yes** | No | Unclear | N/A |
| 1. **Was the selection of study subjects/patients free from bias?**    1. Were inclusion/exclusion criteria specified (e.g., risk, point in disease progression, diagnostic or prognosis criteria), and with sufficient detail and without omitting criteria critical to the study?    2. Were criteria applied equally to all study groups?    3. Were health, demographics, and other characteristics of subjects described?    4. Were the subjects/patients a representative sample of the relevant population? | **Yes** | No | Unclear | N/A |
| 1. **Were study groups comparable?**    1. Was the method of assigning subjects/patients to groups described and unbiased? (Method of randomization identified if RCT)    2. Were distribution of disease status, prognostic factors, and other factors (e.g., demographics) similar across study groups at baseline?    3. Were concurrent controls used? (Concurrent preferred over historical controls.)    4. If cohort study or cross-sectional study, were groups comparable on important confounding factors and/or were preexisting differences accounted for by using appropriate adjustments in statistical analysis?    5. If case control study, were potential confounding factors comparable for cases and controls? (If case series or trial with subjects serving as own control, this criterion is not applicable. Criterion may not be applicable in some cross-sectional studies.)    6. If diagnostic test, was there an independent blind comparison with an appropriate reference standard (e.g., “gold standard”)? | **Yes** | No | Unclear | N/A |
| 1. **Was method of handling withdrawals described?**    1. Were follow up methods described and the same for all groups?    2. Was the number, characteristics of withdrawals (i.e., dropouts, lost to follow up, attrition rate) and/or response rate (cross-sectional studies) described for each group? (Follow up goal for a strong study is 80%.)    3. Were all enrolled subjects/patients (in the original sample) accounted for?    4. Were reasons for withdrawals similar across groups?    5. If diagnostic test, was decision to perform reference test not dependent on results of test under study? | **Yes** | No | Unclear | N/A |
| 1. **Was blinding used to prevent introduction of bias?**    1. In intervention study, were subjects, clinicians/practitioners, and investigators blinded to treatment group, as appropriate? | Yes | No | Unclear | **N/A** |

| - 1. Were data collectors blinded for outcomes assessment? (If outcome is measured using an objective test, such as a lab value, this criterion is assumed to be met.)   2. In cohort study or cross-sectional study, were measurements of outcomes and risk factors blinded?   3. In case control study, was case definition explicit and case ascertainment not influenced by exposure status?   4. In diagnostic study, were test results blinded to patient history and other test results? |  | | | |
| --- | --- | --- | --- | --- |
| 1. **Were intervention/therapeutic regimens/exposure factor or procedure and any comparison(s) described in detail? Were intervening factors described?**    1. In RCT or other intervention trial, were protocols described for all regimens studied?    2. In observational study, were interventions, study settings, and clinicians/provider described?    3. Was the intensity and duration of the intervention or exposure factor sufficient to produce a meaningful effect?    4. Was the amount of exposure and, if relevant, subject/patient compliance measured?    5. Were co-interventions (e.g., ancillary treatments, other therapies) described?    6. Were extra or unplanned treatments described?    7. Was the information for 6.4, 6.5, and 6.6 assessed the same way for all groups?    8. In diagnostic study, were details of test administration and replication sufficient? | **Yes** | No | Unclear | N/A |
| 1. **Were outcomes clearly defined and the measurements valid and reliable?**    1. Were primary and secondary endpoints described and relevant to the question?    2. Were nutrition measures appropriate to question and outcomes of concern?    3. Was the period of follow-up long enough for important outcome(s) to occur?    4. Were the observations and measurements based on standard, valid, and reliable data collection instruments/tests/procedures?    5. Was the measurement of effect at an appropriate level of precision?    6. Were other factors accounted for (measured) that could affect outcomes?    7. Were the measurements conducted consistently across groups? | **Yes** | No | Unclear | N/A |
| 1. **Was the statistical analysis appropriate for the study design and type of outcome indicators?**    1. Were statistical analyses adequately described the results reported appropriately?    2. Were correct statistical tests used and assumptions of test not violated?    3. Were statistics reported with levels of significance and/or confidence intervals?    4. Was “intent to treat” analysis of outcomes done (and as appropriate, was there an analysis of outcomes for those maximally exposed or a dose-response analysis)?    5. Were adequate adjustments made for effects of confounding factors that might have affected the outcomes (e.g., multivariate analyses)?    6. Was clinical significance as well as statistical significance reported?    7. If negative findings, was a power calculation reported to address type 2 error? | **Yes** | No | Unclear | N/A |
| 1. **Are conclusions supported by results with biases and limitations taken into consideration?**    1. Is there a discussion of findings?    2. Are biases and study limitations identified and discussed? | **Yes** | No | Unclear | N/A |
| 1. **Is bias due to study’s funding or sponsorship unlikely?**    1. Were sources of funding and investigators’ affiliations described?    2. Was there no apparent conflict of interest? | **Yes** | No | Unclear | N/A |

# Final score: POSITIVE (+)

# Quality Criteria Checklist: Primary Research

**31. Evaluating depression with multimodal wristband-type wearable device: screening and assessing patient severity utilizing machine-learning, Tazawa(2020)**

Quality Criteria Checklist: Primary Research

| **RELEVANCE QUESTIONS** | | | | |
| --- | --- | --- | --- | --- |
| 1. Would implementing the studied intervention or procedure (if found successful) result in improved outcomes for the patients/clients/population group? (NA for some Epi studies) | Yes | No | Unclear | N/A |
| 2. Did the authors study an outcome (dependent variable) or topic that the patients/clients/population group would care about? | Yes | No | Unclear | N/A |
| 3. Is the focus of the intervention or procedure (independent variable) or topic of study a common issue of concern to mental health practice? | Yes | No | Unclear | N/A |
| 4. Is the intervention or procedure feasible? (NA for some epidemiological studies) | Yes | No | Unclear | N/A |
| ***If the answers to all of the above relevance questions are “Yes,” the report is eligible for designation with a plus (+) on the Evidence Quality Worksheet, depending on answers to the following validity questions.*** | | | | |
| **VALIDITY QUESTIONS** | | | | |
| 1. **Was the research question clearly stated?**    1. Was the specific intervention(s) or procedure (independent variable(s)) identified?    2. Was the outcome(s) (dependent variable(s)) clearly indicated?    3. Were the target population and setting specified? | Yes | No | Unclear | N/A |
| 1. **Was the selection of study subjects/patients free from bias?**    1. Were inclusion/exclusion criteria specified (e.g., risk, point in disease progression, diagnostic or prognosis criteria), and with sufficient detail and without omitting criteria critical to the study?    2. Were criteria applied equally to all study groups?    3. Were health, demographics, and other characteristics of subjects described?    4. Were the subjects/patients a representative sample of the relevant population? | Yes | No | Unclear | N/A |
| 1. **Were study groups comparable?**    1. Was the method of assigning subjects/patients to groups described and unbiased? (Method of randomization identified if RCT)    2. Were distribution of disease status, prognostic factors, and other factors (e.g., demographics) similar across study groups at baseline?    3. Were concurrent controls used? (Concurrent preferred over historical controls.)    4. If cohort study or cross-sectional study, were groups comparable on important confounding factors and/or were preexisting differences accounted for by using appropriate adjustments in statistical analysis?    5. If case control study, were potential confounding factors comparable for cases and controls? (If case series or trial with subjects serving as own control, this criterion is not applicable. Criterion may not be applicable in some cross-sectional studies.)    6. If diagnostic test, was there an independent blind comparison with an appropriate reference standard (e.g., “gold standard”)? | Yes | No | Unclear | N/A |
| 1. **Was method of handling withdrawals described?**    1. Were follow up methods described and the same for all groups?    2. Was the number, characteristics of withdrawals (i.e., dropouts, lost to follow up, attrition rate) and/or response rate (cross-sectional studies) described for each group? (Follow up goal for a strong study is 80%.)    3. Were all enrolled subjects/patients (in the original sample) accounted for?    4. Were reasons for withdrawals similar across groups?    5. If diagnostic test, was decision to perform reference test not dependent on results of test under study? | Yes | No | Unclear | N/A |
| 1. **Was blinding used to prevent introduction of bias?**    1. In intervention study, were subjects, clinicians/practitioners, and investigators blinded to treatment group, as appropriate?    2. Were data collectors blinded for outcomes assessment? (If outcome is measured using an objective test, such as a lab value, this criterion is assumed to be met.)    3. In cohort study or cross-sectional study, were measurements of outcomes and risk factors blinded?    4. In case control study, was case definition explicit and case ascertainment not influenced by exposure status?    5. In diagnostic study, were test results blinded to patient history and other test results? | Yes | No | Unclear | N/A |
| 1. **Were intervention/therapeutic regimens/exposure factor or procedure and any comparison(s) described in detail? Were intervening factors described?**    1. In RCT or other intervention trial, were protocols described for all regimens studied?    2. In observational study, were interventions, study settings, and clinicians/provider described?    3. Was the intensity and duration of the intervention or exposure factor sufficient to produce a meaningful effect?    4. Was the amount of exposure and, if relevant, subject/patient compliance measured?    5. Were co-interventions (e.g., ancillary treatments, other therapies) described?    6. Were extra or unplanned treatments described?    7. Was the information for 6.4, 6.5, and 6.6 assessed the same way for all groups?    8. In diagnostic study, were details of test administration and replication sufficient? | Yes | No | Unclear | N/A |
| 1. **Were outcomes clearly defined and the measurements valid and reliable?**    1. Were primary and secondary endpoints described and relevant to the question?    2. Were nutrition measures appropriate to question and outcomes of concern?    3. Was the period of follow-up long enough for important outcome(s) to occur?    4. Were the observations and measurements based on standard, valid, and reliable data collection instruments/tests/procedures?    5. Was the measurement of effect at an appropriate level of precision?    6. Were other factors accounted for (measured) that could affect outcomes?    7. Were the measurements conducted consistently across groups? | Yes | No | Unclear | N/A |
| 1. **Was the statistical analysis appropriate for the study design and type of outcome indicators?**    1. Were statistical analyses adequately described the results reported appropriately?    2. Were correct statistical tests used and assumptions of test not violated?    3. Were statistics reported with levels of significance and/or confidence intervals?    4. Was “intent to treat” analysis of outcomes done (and as appropriate, was there an analysis of outcomes for those maximally exposed or a dose-response analysis)?    5. Were adequate adjustments made for effects of confounding factors that might have affected the outcomes (e.g., multivariate analyses)?    6. Was clinical significance as well as statistical significance reported?    7. If negative findings, was a power calculation reported to address type 2 error? | Yes | No | Unclear | N/A |
| 1. **Are conclusions supported by results with biases and limitations taken into consideration?**    1. Is there a discussion of findings?    2. Are biases and study limitations identified and discussed? | Yes | No | Unclear | N/A |
| 1. **Is bias due to study’s funding or sponsorship unlikely?**    1. Were sources of funding and investigators’ affiliations described?    2. Was there no apparent conflict of interest? | Yes | No | Unclear | N/A |

# Final score: POSITIVE (+)

**References**

1. Research IaSBDT. Evidence Analysis Manual: Steps in the Academy Evidence Analysis Process: Academy of Nutrition and Dietetics; 2016. ISBN: 978-0-88091-429-1.
2. Handu D, Moloney L, Wolfram T, Ziegler P, Acosta A, Steiber A. Academy of Nutrition and Dietetics methodology for conducting systematic reviews for the Evidence Analysis Library. Journal of the Academy of Nutrition and Dietetics. 2016;116(2):311-8.
