# Supplementary material for: Key Features of Digital Phenotyping for Monitoring Mental Disorders: Systematic Review
Source: J Med Internet Res. 2025 Nov 5;27:e77331. doi: 10.2196/77331 (PMC12588392; doi:10.2196/77331)
Supplement: Multimedia Appendix 3 [file jmir-v27-e77331-s003.docx]

1. **Stratified by target population**

This supplementary material presents a stratified analysis of the findings from the main manuscript (Tables 5-7) by target population type (clinical vs community samples), as summarized in Tables S1 and S2. Table S1 presents values from the synthesized feature summary in Table 5 of the main manuscript, stratified by target population type. Table S2 integrates the device-specific results from Table S1 and reorganizes them by population type, providing a consolidated overview of feature usage across clinical and community samples. In total, 14 studies were based on clinical samples and 8 studies were based on community samples. The Actiwatch studies included only clinical samples and were therefore not stratified.

Due to this imbalance in study numbers, direct comparisons should be interpreted with caution. Nevertheless, several tentative trends can be observed. Features derived from BVP signals (eg, heart rate and interbeat intervals) and ACC-based features tended to be identified more frequently as important in clinical sample studies than in community-based studies, although the differences were modest. In contrast, sleep and phone usage appeared more prominent in community sample studies. Some features—such as app usage, light exposure, and GPS—were generally expected to be more prominent in community-based sensing contexts due to their passive and ubiquitous nature. However, in this analysis, these features were more frequently used and rated as important in clinical sample studies.
This counter-directional pattern limits the interpretability of stratification by population type, as the observed differences in feature importance did not consistently align with expected distinctions between clinical and community contexts. Overall, the stratified analysis did not reveal consistent patterns attributable to sample type alone, suggesting that other factors may have had a greater influence on feature selection and importance.

Table S1. Features used across studies stratified by clinical versus community samples.

|  | | | **All devices** | | | | | | |
| --- | --- | --- | --- | --- | --- | --- | --- | --- | --- |
|  |  |  | **Clinical sample study (n=14)** | | | | **Community sample study (n=8)** | | |
|  |  |  | **SFA** | **SFB**  **(clinical sample)** | **SFW (community sample)** | **TSF (clinical sample)** | **SFB**  **(community sample)** | **SFW (community sample)** | **TSF (community sample)** |
| Feature list | blood volume pulse (BVP) | | 0/0 | 0/1 | 0/0 | 0/1 | 0/0 | 0/0 | 0/0 |
|  |  | heart rate | 0/0 | 6/8 | 0/0 | 6/8 | 1/2 | 2/4 | 3/6 |
|  |  | Interbeat intervals | 0/0 | 1/2 | 0/0 | 1/2 | 0/0 | 0/0 | 0/0 |
|  |  | SpO₂ | 0/0 | 0/0 | 0/0 | 0/0 | 0/1 | 0/0 | 0/1 |
|  | Accelerometer | | 3/3 | 2/4 | 1/1 | 6/8 | 2/2 | 0/2 | 2/4 |
|  |  | Caloric consumption | 0/0 | 1/2 | 1/1 | 2/3 | 0/1 | 0/1 | 0/2 |
|  |  | Sedentary minutes | 0/0 | 0/0 | 0/0 | 0/0 | 0/0 | 0/1 | 0/1 |
|  |  | Activity | 1/2 | 1/3 | 0/1 | 2/6 | 1/2 | 0/2 | 1/4 |
|  |  | Steps | 0/0 | 4/6 | 0/1 | 4/7 | 1/1 | 2/4 | 3/5 |
|  |  | Motion magnitude | 0/0 | 1/1 | 0/0 | 1/1 | 0/0 | 1/1 | 1/1 |
|  | EDA (electrodermal activity) | | 0/0 | 3/3 | 0/0 | 3/3 | 1/1 | 0/0 | 1/1 |
|  | TEMP | | 0/0 | 1/2 | 0/0 | 1/2 | 1/1 | 0/0 | 1/1 |
|  | Sleep | | 0/1 | 2/7 | 1/1 | 3/9 | 3/3 | 3/4 | 6/7 |
|  | Call log | | 1/1 | 1/3 | 0/0 | 2/4 | 1/1 | 0/1 | 1/2 |
|  | Short Message Service (SMS) | | 0/1 | 0/1 | 0/0 | 0/2 | 0/1 | 0/0 | 0/1 |
|  | Phone usage | | 0/1 | 2/3 | 0/0 | 2/4 | 2/2 | 0/0 | 2/2 |
|  | App usage | | 0/1 | 1/3 | 0/0 | 1/4 | 0/0 | 0/1 | 0/1 |
|  | Light exposure | | 1/1 | 1/3 | 0/0 | 2/4 | 0/0 | 0/0 | 0/0 |
|  | GPS | | 0/1 | 2/2 | 0/0 | 2/3 | 0/0 | 0/0 | 0/0 |

Table S2. Proportion of studies identifying each feature as important: clinical versus community samples.

|  | | | **All devices** | | | |  |
| --- | --- | --- | --- | --- | --- | --- | --- |
|  |  |  | **Clinical sample study (n=14)** | | **Community sample study (n=8)** | |  |
|  |  |  | **TSF**  **(clinical sample)** | **% of TSF (clinical)** | **TSF**  **(community sample)** | **% of TSF (community)** | **TSF** |
| Feature list | blood volume pulse (BVP, 64 Hz) | | 0/1 | 0% | 0/0 | 0% | 0/1 |
|  |  | heart rate | 6/8 | 75% | 3/6 | 50% | 9/14 |
|  |  | Interbeat intervals | 1/2 | 50% | 0/0 | 0% | 1/2 |
|  |  | SpO₂ | 0/0 | 0% | 0/1 | 0% | 0/1 |
|  | Accelerometer | | 6/8 | 75% | 2/4 | 50% | 8/12 |
|  |  | Caloric consumption | 2/3 | 66.66% | 0/2 | 0% | 2/5 |
|  |  | Sedentary minutes | 0/0 | 0% | 0/1 | 0% | 0/1 |
|  |  | Activity | 2/6 | 33.33% | 1/4 | 25% | 3/10 |
|  |  | Steps | 4/7 | 57.14% | 3/5 | 60% | 7/12 |
|  |  | Motion magnitude | 1/1 | 100% | 1/1 | 100% | 1/2 |
|  | EDA (electrodermal activity) | | 3/3 | 100% | 1/1 | 100% | 4/4 |
|  | TEMP | | 1/2 | 50% | 1/1 | 100% | 2/3 |
|  | Sleep | | 3/9 | 33.33% | 6/7 | 85.71% | 9/15 |
|  | Call log | | 2/4 | 50% | 1/2 | 50% | 2/5 |
|  | Short Message Service (SMS) | | 0/2 | 0% | 0/1 | 0% | 0/3 |
|  | Phone usage | | 2/4 | 50% | 2/2 | 100% | 4/6 |
|  | App usage | | 1/4 | 25% | 0/1 | 0% | 1/5 |
|  | Light exposure | | 2/4 | 50% | 0/0 | 0% | 2/4 |
|  | GPS | | 2/3 | 66.66% | 0/0 | 0% | 2/3 |

1. **Stratified by analytic approach**

This supplementary material further presents a stratified analysis based on an analytic approach—machine learning versus traditional statistics—as summarized in Tables S3 and S4. Table S3 presents values from the synthesized feature summary in Table 5 of the main manuscript, stratified by analytic groups, while Table S4 summarizes the proportion of studies within each group that identified a given feature as important. Of the included studies, 17 employed machine learning techniques, whereas only 5 used traditional statistical methods. Given this substantial imbalance, the stratified analysis should be interpreted with caution.

In conclusion, while some differences in feature prioritization were observed between the two analytic approaches, the limited number of studies using traditional statistics renders the comparison largely inconclusive. These findings suggest that analytic approach may influence feature selection; however, firmer conclusions will require a more balanced distribution of analytic methods across the literature.

Table S3. Features used across studies stratified by machine learning versus traditional statistics.

|  | | | **Stratification** | | | | | | | |
| --- | --- | --- | --- | --- | --- | --- | --- | --- | --- | --- |
|  |  |  | **Studies using machine learning (n=17)** | | | | **Studies using traditional statistics (n=5)** | | | |
|  |  |  | **Actiwatch** | **Smart band** | **Smart watch** | **All devices** | **Actiwatch** | **Smart band** | **Smart watch** | **All devices** |
|  |  |  | **SFA** | **SFB** | **SFW** | **TSF** | **SFA** | **SFB** | **SFW** | **TSF** |
| Feature list | blood volume pulse (BVP) | | 0/0 | 0/1 | 0/0 | 0/1 | 0/0 | 0/0 | 0/0 | 0/0 |
|  |  | heart rate | 0/0 | 7/8 | 2/3 | 9/11 | 0/0 | 0/2 | 0/1 | 0/3 |
|  |  | Interbeat intervals | 0/0 | 1/2 | 0/0 | 1/2 | 0/0 | 0/0 | 0/0 | 0/0 |
|  |  | SpO₂ | 0/0 | 0/1 | 0/0 | 0/1 | 0/0 | 0/0 | 0/0 | 0/0 |
|  | Accelerometer | | 2/2 | 4/6 | 0/1 | 6/9 | 1/1 | 0/0 | 1/2 | 2/3 |
|  |  | Caloric consumption | 0/0 | 1/3 | 0/1 | 1/4 | 0/0 | 0/0 | 1/1 | 1/1 |
|  |  | Sedentary minutes | 0/0 | 0/0 | 0/1 | 0/1 | 0/0 | 0/0 | 0/0 | 0/0 |
|  |  | Activity | 1/1 | 1/3 | 0/2 | 2/6 | 0/1 | 1/2 | 0/1 | 1/4 |
|  |  | Steps | 0/0 | 5/6 | 2/3 | 7/9 | 0/0 | 0/1 | 0/2 | 0/3 |
|  |  | Motion magnitude | 0/0 | 0/1 | 1/1 | 1/2 | 0/0 | 0/0 | 0/0 | 0/0 |
|  | EDA (electrodermal activity) | | 0/0 | 4/4 | 0/0 | 4/4 | 0/0 | 0/0 | 0/0 | 0/0 |
|  | TEMP | | 0/0 | 2/3 | 0/0 | 2/3 | 0/0 | 0/0 | 0/0 | 0/0 |
|  | Sleep | | 0/0 | 4/8 | 2/3 | 6/11 | 0/1 | 1/2 | 2/2 | 3/5 |
|  | Call log | | 0/0 | 2/4 | 0/1 | 2/5 | 1/1 | 0/0 | 0/0 | 1/1 |
|  | Short Message Service (SMS) | | 0/0 | 0/2 | 0/0 | 0/2 | 0/1 | 0/0 | 0/0 | 0/1 |
|  | Phone usage | | 0/0 | 3/4 | 0/0 | 3/4 | 0/1 | 1/1 | 0/0 | 1/2 |
|  | App usage | | 0/0 | 1/3 | 0/1 | 1/4 | 0/1 | 0/0 | 0/0 | 0/1 |
|  | Light exposure | | 1/1 | 1/2 | 0/0 | 2/3 | 0/0 | 0/1 | 0/0 | 0/1 |
|  | GPS | | 0/0 | 2/2 | 0/0 | 2/2 | 0/1 | 0/0 | 0/0 | 0/1 |

Table S4. Proportion of studies identifying each feature as important: machine learning versus traditional statistics.

|  | | | **Stratification** | | | |  |
| --- | --- | --- | --- | --- | --- | --- | --- |
|  |  |  | **Machine learning (n=17)** | | **Traditional statistics (n=5)** | |  |
|  |  |  | **TSF stratified by analytic approach-machine learning** | **Percentage of TSF among studies using machine learning** | **TSF stratified by analytic approach-traditional statistics** | **Percentage of TSF among studies using traditional statistics** | **TSF** |
| Feature list | blood volume pulse (BVP, 64 Hz) | | 0/1 | 0% | 0/0 | 0% | 0/1 |
|  |  | heart rate | 9/11 | 81.81% | 0/3 | 0% | 9/14 |
|  |  | Interbeat intervals | 1/2 | 50% | 0/0 | 0% | 1/2 |
|  |  | SpO₂ | 0/1 | 0% | 0/0 | 0% | 0/1 |
|  | Accelerometer | | 6/9 | 66.66% | 2/3 | 66.66% | 8/12 |
|  |  | Caloric consumption | 1/4 | 25% | 1/1 | 100% | 2/5 |
|  |  | Sedentary minutes | 0/1 | 0% | 0/0 | 0% | 0/1 |
|  |  | Activity | 2/6 | 33.33% | 1/4 | 25% | 3/10 |
|  |  | Steps | 7/9 | 77.77% | 0/3 | 0% | 7/12 |
|  |  | Motion magnitude | 1/2 | 50% | 0/0 | 0% | 1/2 |
|  | EDA (electrodermal activity) | | 4/4 | 100% | 0/0 | 0% | 4/4 |
|  | TEMP | | 2/3 | 66.66% | 0/0 | 0% | 2/3 |
|  | Sleep | | 6/11 | 54.54% | 3/5 | 60% | 9/15 |
|  | Call log | | 2/5 | 40% | 1/1 | 100% | 2/5 |
|  | Short Message Service (SMS) | | 0/2 | 0% | 0/1 | 0% | 0/3 |
|  | Phone usage | | 3/4 | 75% | 1/2 | 50% | 4/6 |
|  | App usage | | 1/4 | 25% | 0/1 | 0% | 1/5 |
|  | Light exposure | | 2/3 | 66.66% | 0/1 | 0% | 2/4 |
|  | GPS | | 2/2 | 100% | 0/1 | 0% | 2/3 |
