# Supplementary material for: Key Features of Digital Phenotyping for Monitoring Mental Disorders: Systematic Review
Source: J Med Internet Res. 2025 Nov 5;27:e77331. doi: 10.2196/77331 (PMC12588392; doi:10.2196/77331)
Supplement: Multimedia Appendix 4 [file jmir-v27-e77331-s004.docx]

**1. Classification of Data Extraction Methods and Feature Importance Determination Criteria Across Included Studies**

Table S5. Extraction method codes and descriptions of feature importance criteria used in each included study.

| Study No. | Author | Extraction Method Code | Description of Feature Importance Criteria |
| --- | --- | --- | --- |
| 1 | Jacobson et al.[1] | PR (Predictive Performance Emphasis) | Important features were identified based on the authors' selective emphasis within the study's presentation and analysis focus. This study used only two features (movement and light exposure) as predictors. No explicit feature importance metrics (e.g., SHAP, permutation importance) were reported. However, given the limited feature set and the high predictive performance of the XGBoost model (r = 0.855 for BDI-II; r = 0.604 for HAM-D), both were considered important by the authors as sufficient for accurate depression detection. |
| 2 | Jacobson et al.[2] | AE  (Author's Emphasis) | Important features were identified based on the authors' selective emphasis within the study's presentation and analysis focus. A large number of features (n = 9,929) were extracted from actigraphy data, including movement intensity, variability, and temporal patterns. Although explicit feature importance rankings were not reported, movement-related features—such as movement intensity, variability, autoregressive lags, and oscillatory patterns—were consistently emphasized through figures and narrative descriptions in the results and discussion as core predictors. Therefore, ACC-derived activity features were marked as important. |
| 3 | Price et al.[3] | AE  (Author's Emphasis) | Important features were identified based on the authors' selective emphasis within the study's presentation and analysis focus. This study utilized a large set of passive actigraphy features to identify distinct movement patterns through an unsupervised machine learning approach (clustering). However, even though SHAP values were used for model explanation, explicit feature importance rankings or statistically significant individual features were not reported. The identified movement patterns—such as "low activity and high repetitiveness" for depression and "bursts of activity with abnormally long intervals" for schizophrenia—were emphasized as key findings due to their significant association with mental health outcomes. |
| 4 | Aledavood et al.[4] | P  (p-value of traditional statistics) | The important features were extracted by statistical significancy of group differences using traditional statistical tests (e.g., Mann-Whitney U tests). The revealed important features included: location variance, entropy of location, and temporal patterns of communication and smartphone use. |
| 5 | Anmella et al.[5] | PR (Predictive Performance Emphasis) | We extracted important features based on permutation feature importance analysis and referred to the description of the results in the article: "ACC was the most relevant channel for predicting mania, whereas EDA and HR, followed by TEMP, were the most relevant channels for predicting both BD and unipolar depression. The BVP channel did not change performance for either better or worse" |
| 6 | Zou et al.[6] | AE  (Author's Emphasis) | Important features were identified based on the authors’ emphasis in the study’s analysis and presentation. The study employed a comprehensive set of passive sensing features (as categorized in Table 2) with deep learning sequence models (e.g., RNNs, LSTMs, Transformers) to predict treatment response. Although explicit feature importance rankings or statistical significance tests were not reported, the authors consistently highlighted longitudinal patterns—particularly related to phone and app usage—as key contributors to predictive performance, as evidenced by the results shown in Figures 1, 3, and 4 and the accompanying narrative discussion. |
| 7 | Pedrelli et al.[7] | PR (Predictive Performance Emphasis) | Important features were identified based on a Boruta algorithm–based feature importance analysis, which selected the most relevant features for predicting treatment response. Out of 877 initial features, 39 were specifically selected by the Boruta algorithm for inclusion in deep learning sequence models (e.g., RNNs, LSTMs, Transformers). While technically more features could have been selected, it appears that the authors' judgment—likely considering factors such as interpretability—influenced the decision to focus on these 39. For the purpose of this review, the selected features were reclassified into categories such as phone usage, app usage, ACC, and call log–related features. For instance, the study reported feature importance rankings such as: *average time phone screen was on over 24 h* and *average duration phone screen was on from 8 a.m. to 6 p.m.* These were therefore categorized as phone usage. |
| 8 | Wang et al.[8] | P  (p-value of traditional statistics) | The important features were extracted by statistical significance as determined by generalized linear mixed models (p-value < 0.05). |
| 9 | Sano et al.[9] | PR (Predictive Performance Emphasis) | Important features were identified based on a machine learning–based feature importance analysis, from a total of 1,260 candidate features. We reclassified all features reported as important in the study into the categories used in our analysis. |
| 10 | Hong et al.[10] | PR (Predictive Performance Emphasis) | Important features were identified using permutation feature importance based on deep learning models, from a total of 31 input features. We reclassified all important features presented in the study into the categories used in our framework. |
| 11 | Ahmed et al.[11] | PR (Predictive Performance Emphasis) | Important features were identified based on the authors' selective emphasis within the study's presentation and analysis focus. This study evaluated the predictive performance of individual modalities (GSR, HR, ACC) for valence and arousal classification using machine learning models. Although no explicit feature importance metrics were reported, the authors highlighted certain modalities as more feasible predictors based on higher accuracy or F1-scores across different depression severity groups. Accordingly, we included GSR, HR, and ACC as important features in our analysis. |
| 12 | Price et al.[12] | PR (Predictive Performance Emphasis) | As the study presented only five important features derived from passive data using Shapley Additive exPlanations (SHAP), we extracted and reclassified these features according to our framework. |
| 13 | Bai et al.[13] | AE  (Author's Emphasis) | We simply adopted the feature categorization used in this study. The authors classified all device-derived features into four predefined categories—phone usage, sleep data, step count, and heart rate data—without applying any feature selection or feature importance analysis to determine these groups. Therefore, it can be said that the researchers arbitrarily selected feature types based on the types of data available from their devices. Within each category, however, feature selection methods—such as L1-based selection and tree-based algorithms—were applied to a large set of sub-features. For example, in the case of step count, 230 features from the steady group and 382 features from the swing group were screened, and between 5 and 48 features were selected per model to develop prediction algorithms. This procedure was conducted separately for each data category to allow for model comparisons. |
| 14 | Mahendran et al.[14] | PR (Predictive Performance Emphasis) | We extracted important features as they described in the Methodology section. All the features derived from smartwatch sensors were included in the machine learning odels, and conducted feature selection procedure; though, there were no reports on important features. The predefined features were narrowly set, Accelerometer, Gyroscope, and Heart-rate. In the heart-rate feature, calories burned, blood pressure, and oxygen levels were included. |
| 15 | Cho et al.[15] | P  (p-value of traditional statistics) | Total features were presented in the Multimedia Appendix. Important features were extracted based on those reported as statistically significant. It appears that the authors conducted a series of internal trials and statistical tests (Kolmogorov–Smirnov and Mann–Whitney U tests), and among various candidate features, only a subset showing significant behavioral changes after feedback was reported. Specifically, heart rate, light exposure, and step count were highlighted as key features demonstrating significant post-feedback improvement. In contrast, none of the sleep-related features reached statistical significance. Therefore, we confirmed important features based on those identified as statistically significant. |
| 16 | Cho et al.[16] | PR (Predictive Performance Emphasis) | The included features were identical to those used in Cho et al. [15], and the full list of features was provided in the Multimedia Appendix. Rather than relying on traditional statistical methods, this study identified important features through machine learning-based procedures. Given the large number of generated features (e.g., mean, variance, entropy), most features corresponding to our predefined categories were included among the selected ones. In summary, we extracted important features based on those reported or presented in the study, regardless of their rank. |
| 17 | Tazawa et al.[17] | PR (Predictive Performance Emphasis) | We referred to the machine learning–driven feature importance reported in the study. Due to the large number of subfeatures generated under each representative feature type (e.g., various percentiles and time-windowed measures of step counts), the total number of distinct feature types was relatively limited. Therefore, we extracted all features presented as important, as they could be appropriately mapped to our predefined feature categories. |
| 18 | Čermák et al.[18] | AE  (Author's Emphasis) | Since this study conducted only a descriptive correlation analysis—with a predefined criterion considering a Spearman’s correlation of ≥0.7 as indicative of a strong relationship—we had no choice but to rely on the authors’ emphasis. We extracted all features presented in the tables and figures, but determined importance based on whether the reported correlation scores met the predefined threshold. |
| 19 | Zhang et al.[19] | PR (Predictive Performance Emphasis) | We referred to the SHAP plots to identify important features. The study presented the top 15 features, which included a combination of EMA (Ecological Momentary Assessment) data, demographic variables, and passive digital phenotyping features. Although the predictive contributions of digital phenotyping features were much lower than those of EMA and demographic features, we extracted all features that appeared in the SHAP plots or were described as important in the main text. Although digital phenotyping features contributed less to prediction than EMA or demographic variables, we treated those appearing in the SHAP plots or highlighted in the text as important. This is because other digital phenotyping features showed even lower contributions and were not presented at all. |
| 20 | Song et al.[20] | P  (p-value of traditional statistics) | We referred to the features that showed statistical significance (p-values) in the multilevel modeling analysis, which was based on traditional statistical methods. |
| 21 | Narziev et al.[21] | AE  (Author's Emphasis) | We referred to the features emphasized by the authors as important. Although the study did not present standardized statistical metrics (e.g., p-values or SHAP plots), it proposed five DSM-5-based symptom clusters (mood, physical activity, sleep, social activity, and food intake) as core domains. Feature contributions were not ranked but were incorporated into machine learning models (SVM, RF), and classification performance was reported. Hence, we extracted all features used for model training and interpretation based on the authors’ theoretical emphasis on symptom clusters. |
| 22 | Horwitz et al.[22] | N/A | We did not extract any important features from this study, as no features were considered important by the authors. Although all utilized input features were described, the study did not apply any statistical test (e.g., p-value, SHAP, or permutation) to assess feature importance. Instead, the authors compared predictive performance between models using only daily mood diary data and those combining mood with Fitbit sensor data. Interestingly, the inclusion of sensor data rather lowered prediction performance. Therefore, no specific feature was emphasized or statistically validated. |

**2. Example of coding process**

This section outlines the protocol for the “○/●” coding procedure in detail. We illustrate the process using Study No. 4 by Aledavood et al [4] as an example. This study describes the extracted features in the Data Preprocessing section, with subtitles such as “Communication and Smartphone Screen Use,” “Apps,” “Accelerometer,” “Sleep and Activity Detection,” and “Location and Mobility” (see Table S6). Table S6 summarizes how these features were described in the original manuscript and how we mapped them into the feature categories used in our review (Table 5-7 of the main texts). We directly cited relevant words or phrases from Aledavood et al [4].

For instance, Aledavood et al [4] includes the subtitle Apps and describes the corresponding feature as “frequency and duration of smartphone apps.” Based on this description, we classified it under the App Usage category. In the first round of coding (“○”), we assigned a “○” to any feature that was mentioned at least once in the methods section. In the second round (“●”), features were assigned a “●” if they were highlighted as important in the main results, especially in statistical tables or visualizations.

For example, CallLog was rated as “●” based on the following evidence: while the initial unadjusted univariate analysis (Mann-Whitney U test, Table 2; of Aledavood et al [4]) showed no statistically significant differences for any call-related features (e.g., number/duration of incoming and outgoing calls), Figure 9 (of Aledavood et al [4]) provided a more intuitive visualization suggesting minor differences. Moreover, the primary analysis—linear mixed models adjusted for covariates—identified both incoming and outgoing call duration as statistically significant predictors (Table 3 of Aledavood et al [4]). These findings were also highlighted in the abstract. In cases where the results were inconsistent across analyses, we based our final coding on the findings of the primary analysis and the emphasis placed by the authors in the manuscript.

Please note that the sleep-related features in Aledavood et al [4] were derived from a combination of two devices: the Actiwatch and a non-wearable bed sensor. However, these variables were not prominently reported in the results section through figures or tables, likely due to their low statistical significance.

Table S6. An example of coding process.

| **Aledavood et al.(26)** | |  | **Main text of this study** | | | **Aledavood et al.(26)** |  | **Main text of this study** |
| --- | --- | --- | --- | --- | --- | --- | --- | --- |
| **Subtitle** | **Description in the manuscript** |  | **Feature list (Table 5)** |  | **1^st^ round of rating** | **Evidence** |  | **2^nd^ round of rating** |
| Communication and Smartphone Screen Use | “Incoming or outgoing calls” | 🡺 | CallLog | 🡺 | ○ | Figure 9, Table 3 showed all the features were statistically significant. | 🡺 | **●** |
|  | “SMS text messages” | 🡺 | SMS | 🡺 | ○ | Tables 2 and 3 showed all the features were not statistically significant. | 🡺 | ○ |
|  | “Screen use” | 🡺 | Phone Usage | 🡺 | ○ | Table 1 and Figure 9 showed no statistically significant difference in screen use | 🡺 | ○ |
| Apps | “Frequency and duration of smartphone apps (by function types: sports, communication, leisure, social media, etc.)” | 🡺 | App Usage | 🡺 | ○ | Table 1 showed all the features were not statistically significant. | 🡺 | ○ |
| Accelerometer | “Euclidean norm of the acceleration vectors” | 🡺 | ACC | 🡺 | ○ | Figure 9 showed meaningful differences in distributions, and Table 3 presented significant differences in ‘Magnitude SD morning’ of accelerometer (*p*-value=0.02). | 🡺 | ● |
| Sleep and Physical Activity | “Sleep variables were derived from actigraph and bed sensor data” | 🡺 | N/A | 🡺 | ○ | No figure or table reported. | 🡺 | ○ |
|  | “Activity count and status (active, rest, and sleep)” | 🡺 | Activity | 🡺 | ○ | Figure 8 showed all the related features—activity, percentage of time at home, number of significant places, distance traveled, average speed—were not statistically significant. | 🡺 | ○ |
| Location and Mobility | Several mobility features (distance traveled, significant places, normalized entropy, proportion of time spent at home, speed mean, location variance) were extracted from the GPS sensor | 🡺 | GPS | 🡺 | ○ | Figure 8 showed only normalized entropy and location variance were significant; however, Table 3 showed no significant results in either feature. | 🡺 | ○ |

**Reference**

1. Jacobson NC, Weingarden H, Wilhelm S. Digital biomarkers of mood disorders and symptom change. npj Digit Med. 2019;2(1):3.

2. Jacobson NC, Weingarden H, Wilhelm S. Using digital phenotyping to accurately detect depression severity. The Journal of nervous and mental disease. 2019;207(10):893-6.

3. Price GD, Heinz MV, Zhao D, Nemesure M, Ruan F, Jacobson NC. An unsupervised machine learning approach using passive movement data to understand depression and schizophrenia. Journal of affective disorders. 2022;316:132-9.

4. Aledavood T, Luong N, Baryshnikov I, Darst R, Heikkilä R, Holmén J, et al. Multimodal digital phenotyping study in patients with major depressive episodes and healthy controls (mobile monitoring of mood): Observational longitudinal study. JMIR Ment Health. 2025;12:e63622.

5. Anmella G, Corponi F, Li BM, Mas A, Sanabra M, Pacchiarotti I, et al. Exploring digital biomarkers of illness activity in mood episodes: hypotheses generating and model development study. JMIR mHealth and uHealth. 2023;11(1):e45405.

6. Zou B, Zhang X, Xiao L, Bai R, Li X, Liang H, et al. Sequence modeling of passive sensing data for treatment response prediction in major depressive disorder. IEEE Trans Neural Syst Rehabil Eng. 2023;31:1786-95.

7. Pedrelli P, Fedor S, Ghandeharioun A, Howe E, Ionescu DF, Bhathena D, et al. Monitoring changes in depression severity using wearable and mobile sensors. Front Psychiatry. 2020;11:584711.

8. Wang R, Wang W, DaSilva A, Huckins JF, Kelley WM, Heatherton TF, et al. Tracking depression dynamics in college students using mobile phone and wearable sensing. Proceedings of the ACM on Interactive, Mobile, Wearable and Ubiquitous Technologies. 2018;2(1):1-26.

9. Sano A, Taylor S, McHill AW, Phillips AJ, Barger LK, Klerman E, et al. Identifying objective physiological markers and modifiable behaviors for self-reported stress and mental health status using wearable sensors and mobile phones: observational study. Journal of medical Internet research. 2018;20(6):e210.

10. Hong M, Kang R-R, Yang JH, Rhee SJ, Lee H, Kim Y-g, et al. Comprehensive Symptom Prediction in Inpatients With Acute Psychiatric Disorders Using Wearable-Based Deep Learning Models: Development and Validation Study. Journal of medical Internet research. 2024;26:e65994.

11. Ahmed A, Ramesh J, Ganguly S, Aburukba R, Sagahyroon A, Aloul F. Investigating the feasibility of assessing depression severity and valence-arousal with wearable sensors using discrete wavelet transforms and machine learning. Information. 2022;13(9):406.

12. Price GD, Heinz MV, Song SH, Nemesure MD, Jacobson NC. Using digital phenotyping to capture depression symptom variability: detecting naturalistic variability in depression symptoms across one year using passively collected wearable movement and sleep data. Translational Psychiatry. 2023;13(1):381.

13. Bai R, Xiao L, Guo Y, Zhu X, Li N, Wang Y, et al. Tracking and monitoring mood stability of patients with major depressive disorder by machine learning models using passive digital data: prospective naturalistic multicenter study. JMIR mHealth and uHealth. 2021;9(3):e24365.

14. Mahendran N, Vincent DR, Srinivasan K, Chang C-Y, Garg A, Gao L, et al. Sensor-assisted weighted average ensemble model for detecting major depressive disorder. Sensors. 2019;19(22):4822.

15. Cho C-H, Lee T, Kim M-G, In HP, Kim L, Lee H-J. Mood prediction of patients with mood disorders by machine learning using passive digital phenotypes based on the circadian rhythm: prospective observational cohort study. Journal of medical Internet research. 2019;21(4):e11029.

16. Cho C-H, Lee T, Lee J-B, Seo JY, Jee H-J, Son S, et al. Effectiveness of a smartphone app with a wearable activity tracker in preventing the recurrence of mood disorders: prospective case-control study. JMIR Ment Health. 2020;7(8):e21283.

17. Tazawa Y, Liang K-c, Yoshimura M, Kitazawa M, Kaise Y, Takamiya A, et al. Evaluating depression with multimodal wristband-type wearable device: screening and assessing patient severity utilizing machine-learning. Heliyon. 2020;6(2).

18. Čermák J, Pietrucha S, Nawka A, Lipone P, Ruggieri A, Bonelli A, et al. An Observational Pilot Study using a Digital Phenotyping Approach in Patients with Major Depressive Disorder Treated with Trazodone. Front Psychiatry. 2023;14:1127511.

19. Zhang Y, Stewart C, Ranjan Y, Conde P, Sankesara H, Rashid Z, et al. Large-scale digital phenotyping: identifying depression and anxiety indicators in a general UK population with over 10,000 participants. Journal of Affective Disorders. 2025;375:412-22.

20. Song S, Seo Y, Hwang S, Kim H-Y, Kim J. Digital phenotyping of geriatric depression using a community-based digital mental health monitoring platform for socially vulnerable older adults and their community caregivers: 6-week living lab single-arm pilot study. JMIR mHealth and uHealth. 2024;12(1):e55842.

21. Narziev N, Goh H, Toshnazarov K, Lee SA, Chung K-M, Noh Y. STDD: short-term depression detection with passive sensing. Sensors. 2020;20(5):1396.

22. Horwitz AG, Kentopp SD, Cleary J, Ross K, Wu Z, Sen S, et al. Using machine learning with intensive longitudinal data to predict depression and suicidal ideation among medical interns over time. Psychological medicine. 2023;53(12):5778-85.
