# Supplementary material for: Key Features of Digital Phenotyping for Monitoring Mental Disorders: Systematic Review
Source: J Med Internet Res. 2025 Nov 5;27:e77331. doi: 10.2196/77331 (PMC12588392; doi:10.2196/77331)
Supplement: Multimedia Appendix 5 [file jmir-v27-e77331-s005.docx]

1. **Summary of Included Studies and Description of Used Features by Device Type**

This subsection details the evidence sources underlying the synthesized findings, including information on each study and its employed features. The primary distinction among the studies is the type of device used. The following section summarizes each study, with particular emphasis on features identified as important or contributing substantially to mental health prediction.

***Actiwatch***

Among the 22 studies included in this systematic review, four employed smartphones in combination with Actiwatch devices (Tables 4-5). Notably, Jacobson was involved in three of these studies [1-3]. The first study was conducted in Brazil, where 15 outpatients were recruited [1]. The study aimed to examine the predictive power of digital phenotyping for depression severity, given the limited evidence at the time regarding the prediction of severity rather than the mere detection of depression. In this study, participants wore Actiwatch-L devices for one week. As with other Actiwatch-based studies, the researchers did not report a detailed list of extracted features by name or format. Instead, the feature set was described in broad terms such as “continuous movement,” “ambient light exposure,” and “digital biomarkers created using the square root and square of light and actigraphy data.” Only two feature types—ACC and light exposure—were explicitly confirmed (Table 5-7). Using these limited digital phenotyping features, the researchers reported strong predictive accuracy for depression severity, with correlation coefficients of r = 0.855 for self-reported and r = 0.604 for clinician-rated outcomes [2]

The second research, Jacobson et al [2], conducted a follow-up study using public-use actigraphy data collected from patients with mood disorders and healthy controls in Norway. The purpose of the study was to identify generalizable digital biomarkers capable of predicting both diagnostic status and changes in depressive symptoms over two weeks. Unlike their other work [1], which utilized only 2 basic features—accelerometry and light exposure—this study focused on the development and validation of an extensive set of digital features derived from raw Actiwatch data. Participants wore Actiwatch-L devices continuously for up to two weeks. This study demonstrated that Actiwatch is capable of producing a much broader range of data features than previously utilized. While the features of the study were marked under only the “Activity” feature in Table 5-7, this classification reflects the fact that all 9,929 features were derived from movement-based signals. Thus, although the feature set was extensive and multidimensional in nature, its exclusive reliance on ACC data justifies its representation under the “Activity” category. Importantly, using these activity-based features alone, the model achieved a diagnostic accuracy of 89.1% (κ=0.773) and a strong correlation (r=0.782) in predicting changes in depressive symptoms over the two weeks.

The third study was led by Price, with Jacobson participating as a co-author [3]. This study was carried out within the same medical and research infrastructure as Jacobson et al. [2]. The main goal of the study was to explore whether natural patterns in passively collected movement data—when analyzed using unsupervised machine learning—could effectively reflect and distinguish the clinical categories of major depressive disorder, schizophrenia, and healthy controls, as defined by the Diagnostic and Statistical Manual of Mental Disorders (DSM). Additionally, the study sought to characterize the unique movement patterns associated with each group. The researchers exclusively collected raw minute-level activity counts, derived from ACC data. A key strength of this study lies in its detailed explanation of the data preprocessing procedures. It described methods including Uniform Manifold Approximation and Projection (UMAP), UMAP-based Euclidean distance calculation, and the computation of normalization scores. The results showed significant differences in activity patterns among the three groups. Consequently, the study demonstrated that ACC data can serve as a valuable predictor for DSM-based diagnostic classification.

Aledavood et al [4] conducted a multimodal digital phenotyping study in Finland, targeting individuals experiencing major depressive episodes, including those diagnosed with MDD, bipolar disorder (BD), or borderline personality disorder (BPD), alongside healthy controls. The primary aim of the study was to investigate whether behavioral features passively collected through personal digital devices could distinguish patients from controls and predict the severity of depression symptoms, as measured by the PHQ-9. During the two-week active phase, participants wore Philips Actiwatch 2 devices, which recorded 30-second activity counts and sleep-wake states. In addition, the study employed ballistocardiography-based bed sensors, which measured HR, heart rate variability (HRV), respiration rate, and stroke volume. Despite this wide range of biosignals, no variables emerged as significant predictors of depression severity. Furthermore, sleep duration and bed occupancy patterns, derived from both actigraphy and bed sensors, showed no significant between-group differences or associations with PHQ-9 scores. Instead, significant predictors were limited to smartphone- and wrist-derived behavioral features. Notably, shorter durations of incoming and outgoing calls (β=–0.08, *P*<.001; β=0.05, *P*<.02, respectively) and reduced variability in morning physical activity captured via phone ACC (β=–2.05, *P*=.02) were associated with higher depression severity.

***Smartband***

Anmella et al [5] conducted a prospective observational study in Spain aimed at identifying physiological digital biomarkers capable of predicting mood episode severity and polarity among individuals with BD, MDD, and Healthy controls. The study addressed two primary research questions: (1) whether wearable-derived physiological data could predict intra-individual transitions from acute to response and remission states, and (2) whether such data could distinguish mood episode polarity (manic, depressive, mixed, euthymic) among different individuals. Participants wore Empatica E4 wristbands, which collected ACC, HR, EDA, TEMP, and BVP over 48 hours. Permutation importance analysis revealed that ACC was the strongest predictor for manic states, while EDA and HR were most predictive for both unipolar and bipolar depression. In contrast, BVP showed near-zero predictive contribution, indicating its limited utility in mental health symptom detection. Associations between physiological data and symptom-specific scale items further confirmed that ACC was linked to motor activity, insomnia, and psychomotor retardation, while EDA and HR were associated with aggressive behavior, psychic anxiety, and stress-related symptoms.

Zou et al [6] conducted a prospective cohort study in China to examine whether passively collected behavioral data during the first 2 weeks of treatment could be used to predict treatment response at the end of a 12-week course in patients with MDD. Participants were recruited from outpatient clinics at four psychiatric sites and wore a digital wristband while also installing a custom-built smartphone app. These tools passively recorded data on call logs, screen and app usage, sleep patterns, step count, and HR. Of the 358 initially enrolled participants, 245 completed the follow-up protocol, and of those, only 113–175 participants had sufficient valid sensor data (≥10 days of data in the first 2 weeks) to be included in the final analyses, depending on the modality. A total of 71 behavioral features were extracted daily from both smartphone and wearable devices. To model the longitudinal data with high missingness, the authors employed Gated Recurrent Units (GRU)-D, a variant of GRU that incorporates decay mechanisms to account for the timing and informativeness of missing values. Evaluation results demonstrated that the GRU-D model outperformed traditional machine learning and standard recurrent models (eg, LSTM and GRU) across recall, F1 score, and AUROC metrics. Smartphone-based features such as screen usage, app usage, and phone activity were found to be more predictive than call logs or wearable-only features.

Pedrelli et al [7] conducted a longitudinal study in the United States to investigate whether passively collected behavioral and physiological data could be used to estimate changes in depressive symptom severity among individuals diagnosed with MDD. A total of 31 participants with MDD were enrolled and monitored over an eight-week period, during which depressive symptoms were assessed at six time points using the HDRS-17. Passive data were continuously collected via Empatica E4 wristbands and Android smartphones. The wristbands recorded EDA, HRV, TEMP, ACC, and sleep, while the smartphones captured screen time, app usage, communication behavior, and GPS-derived mobility patterns. Unexpectedly, the machine learning models showed that using smartphone features alone yielded the highest predictive accuracy (MAE=3.88; r=0.70), outperforming both the wearable-only and combined-feature models. However, Boruta feature selection revealed that several wearable-derived features—particularly EDA and HRV—remained among the most informative predictors, alongside indicators of mobile engagement and location variability. This apparent inconsistency between model-level performance and feature-level importance may reflect issues such as data quality, feature redundancy, or overfitting risks associated with multimodal integration.

Wang et al [8] conducted a longitudinal study at Dartmouth College to investigate whether passive sensing data from smartphones and wearable devices could track and predict depressive symptoms in college students. A total of 83 undergraduate students were monitored over two 9-week academic terms, during which depressive symptoms were assessed using the PHQ-8 (pre/post) and the PHQ-4 (weekly). Each participant wore a Microsoft Band 2 device, which captured physiological measures such as heart rate. This research introduced a set of symptom-specific features that aligned with the Diagnostic and Statistical Manual of Mental Disorders (DSM-5) criteria. These features reflected sleep changes through variation in sleep duration and timing; difficulty concentrating based on phone usage patterns in study-related contexts; anhedonia, or loss of interest and pleasure, inferred from decreased mobility, reduced conversational engagement, and prolonged stationary behavior; and low mood or fatigue, estimated from wearable-based HR signals. The suggested key predictors of higher symptom severity included longer phone unlock duration in academic settings, irregular sleep-wake patterns, and lower mobility (e.g., fewer visited locations and more stationary time). Although HR did not show a strong individual correlation with depression scores, it was still retained as a relevant predictor in the modeling process.

Sano et al [9] uniquely employed two wearable sensors concurrently in a large-scale observational study aimed at identifying physiological and behavioral markers associated with self-reported stress and mental health among college students. A total of 201 participants were recruited and completed pre- and post-study questionnaires. They wore two types of wrist-worn sensors: the Q-Sensor (Affectiva) and the Motion Logger (Ambulatory Monitoring Inc.). The Q-Sensor collected skin conductance (SC), TEMP, ACC, and ambient light, primarily capturing physiological arousal and stress responses. In parallel, the Motion Logger, a validated actigraphy device, measured activity levels and sleep patterns, functioning similarly to standard Actiwatches. Additionally, a custom smartphone app captured calls, SMS, screen-on duration, and GPS-based mobility patterns. Machine learning models were applied to classify participants into high vs. low stress and mental health. Sensor-derived features, particularly SC and TEMP, achieved the highest classification accuracy for both stress (up to 78%) and mental health (87%). Modifiable behavioral features such as napping frequency, study duration, and phone usage also contributed meaningfully (accuracy up to 74–79%), while mobile phone features alone performed less well overall.

Hong et al [10] is a prospective, longitudinal, multicenter study conducted in South Korea. The aim was to develop and validate deep learning models that predict multidimensional psychiatric symptoms in inpatients with acute psychiatric disorders. A total of 191 participants with mood disorders or schizophrenia spectrum disorders were recruited from four psychiatric wards across three hospitals, and observed for an average of 20.7 days. Participants wore a wrist-worn device, the URBAN HR (Partron Co, Ltd.), which continuously collected HR, ACC, and location data throughout the hospitalization period. In addition to raw sensor signals, the device also generated derived features such as calories burned, step count, distance traveled, and a sleep index, which were aggregated and included in the model input. Deep learning models were designed to predict either within-subject symptom deterioration (ie, score increases from the previous assessment) or between-subject symptom severity scores, and to predict each scale either individually (Single) or simultaneously (Multi) using a multitask learning approach. Feature importance analysis indicated that variables such as location entropy, number of unique heart rate values, and time spent outside patient rooms were influential across several models.

Ahmed et al [11] conducted a feasibility study to evaluate whether physiological signals collected from wrist-worn wearable sensors could be used to classify depression severity and affective states in daily life settings. The study utilized the Data Acquisition, Preprocessing, and Parameter Estimation for Predictive Evaluation and Reporting dataset, which included five days of continuous data from 87 participants, all of whom had moderate to severe depressive symptoms based on the BDI-II. Participants wore the Psychorus wristband, which recorded HR, galvanic skin response (GSR), and three-axis ACC. All signals were analyzed using the Discrete Wavelet Transform (DWT). From the wavelet coefficients, the authors extracted a total of 12 statistical features, including entropy, zero and mean crossing rates, mean, standard deviation (SD), median, variance, root mean square (RMS), and four percentile values (5th, 25th, 75th, and 95th). These features were calculated separately for each signal type. The classification tasks included distinguishing between moderate vs. severe depression and classifying valence and arousal states (low vs. high), using various machine learning models such as CatBoost, Random Forest (RF), support vector machine (SVM), and XGBoost. Although the study did not explicitly rank individual feature importance, the results showed that GSR and ACC features were more useful in classifying emotional valence and arousal in moderately depressed individuals, while HR and GSR were more relevant among those with severe depression.

Price et al [12] investigated whether passively collected movement and sleep data from wearable devices could predict long-term variability in depression symptoms over one year. A total participants were 939 adults and 12 passive behavioral features were measured, including average activity levels, active and sedentary day counts, recent step count, sleep durations, sleep onset time and its variability, total sleep duration range, and weekly frequencies of hyposomnia (nights with <5 hours of sleep) and hypersomnia (>9 hours). These were combined with eight biodemographic variables, such as sex, Body Mass Index (BMI), migraine diagnosis, and financial difficulty. A stacked ensemble machine learning model revealed that multimodal models incorporating both behavioral and biodemographic features outperformed unimodal ones. Shapley Additive exPlanations (SHAP) analysis identified longer weekday sleep duration, more frequent hyposomnia episodes, lower step count, wider variability in total sleep time, and shorter weekend sleep as the most influential predictors of symptom variability. The findings highlight the predictive value of instability in sleep and PA patterns for identifying individuals at risk for fluctuating depressive symptoms.

Bai et al [13] examined whether variations in passively collected data from smartphones and wearable sensors could be used to classify mood stability among patients with MDD. Data were collected from 334 participants using the Mi Band 2 wristband over a 12-week period. From this, the researchers extracted a total of 252 features across several modalities: phone call logs (eg, call frequency, duration, and entropy of contacts), phone usage patterns (screen time and app usage by category and time of day), sleep metrics (light/deep/total sleep duration, sleep onset/wake time, and sleep quality ratios), step counts (daily totals and hourly patterns), and HR data (cosinor parameters such as amplitude and acrophase during sleep). Among all combinations tested, the model that combined call logs, sleep, step count, and HR data yielded the highest performance in classifying mood stability, achieving up to 76.7% accuracy and 90.4% recall. Feature selection analyses showed that the most predictive features were those related to sleep quality and structure (e.g., ratios of light and deep sleep, total sleep variability), social behavior (number of contacts, call entropy), PA (step count distributions), and circadian rhythm of HR. In contrast, app usage features had minimal predictive value and reduced model performance when included.

Mahendran et al [14] developed a machine learning model to detect MDD using a combination of questionnaire-based data and sensor-derived data from the Mi Band 3 smart band. Although the authors referred to the device as a smartwatch, it would be more accurate to classify it as a smart band, given its functionality and hardware limitations relative to full-featured smartwatches. The study used data from 450 individuals, including responses to HDRS and objective signals from the device’s accelerometer and heart rate sensor. After preprocessing and mean imputation, correlation-based feature selection reduced the initial 35 variables to a final set of 16: nine questionnaire-derived features (e.g., sadness, irritability, suicidal thoughts, bodily symptoms), six ACC-derived statistical features (SD, root mean square, root sum square, upper and lower quartiles, kurtosis), and mean HR. While individual feature importance scores were not reported, the study emphasized that combining subjective symptom data with passively collected movement and physiological signals led to superior classification accuracy.

Cho et al [15] and Cho et al [16] were both conducted as part of a continuous digital mental health research initiative funded by the Korea Health 21 Research and Development (R&D) Project, with the shared aim of utilizing passive digital phenotyping and wearable sensor data to support the management of mood disorders. While both studies originated from the same project and employed similar sensing modalities, they differed in their research objectives, analytical approaches, and most importantly, in how predictive features were selected and applied. Firstly, Cho et al [16] focused on developing a machine learning–based model to forecast mood states and episodes. A total of 55 patients with MDD or BD I/II were monitored using the Fitbit Charge HR, which collected 13 core features related to circadian behavior and physiology: light exposure, sleep, activity levels, HR, and derived variables (e.g., wake after sleep onset, sleep start time). These were expanded into 130 features by applying statistical summarization (mean, variance, entropy) across 3-time windows. Using random forest classifiers, the study found that personalized models yielded higher accuracy than generalized ones. The most predictive features were those representing circadian rhythm stability, such as: circadian rhythm amplitude, light exposure during daytime, and steps during daytime. Secondly, Cho et al [15] applied the previously developed prediction framework in a real-world feedback intervention context. It used the same 130 features extracted from the Fitbit Charge HR, including sleep, activity, light, and heart rate, to generate an H-score representing circadian rhythm integrity. Unlike the 2019 study, this one did not rank feature importance directly through machine learning.

Tazawa et al [17] conducted a multicenter study in Japan to develop a machine learning model capable of screening for depression and assessing symptom severity using multimodal data collected from a wristband-type wearable device (Silmee W20, TDK, Tokyo). A total of 45 patients with mood disorders and 41 healthy controls participated, generating over 5,250 days of continuous data. The Silmee W20 recorded step count, caloric consumption, body movement, sleep time, HR, ST, and ultraviolet (UV) light exposure. From these modalities, the authors extracted 63 engineered features, including percentiles, SD, and inter-modal correlations. A machine learning framework was used to train and evaluate both depression screening and severity prediction models. Among all features, those derived from TEMP, sleep patterns, and particularly the correlation between sleep and TEMP, consistently emerged as the most predictive. Features related to PA—such as body movement and energy expenditure—also showed notable predictive value. In contrast, while HR and UV exposure exhibited statistically significant group differences, their contributions to model performance were relatively limited.

***Smartwatch***

Čermák et al [18] conducted an 8-week observational pilot study to examine whether passively collected data from a smartwatch could complement clinical evaluations of depression severity in patients treated with trazodone once daily. Eleven patients were enrolled, and passive data were collected using the Withings Move ECG (smartwatch, which tracked a range of features including distance traveled, step count, calories burned, sleep duration, deep/light sleep phases, awake time, wake-up count, and an aggregated sleep score derived from these variables. The sleep score was calculated nightly based on total sleep time, depth, regularity, and interruptions, yielding a 0–100 scale. Although the study did not aim to build predictive models, it explored Spearman correlations between passive features and the MADRS depression scores. The most consistent findings were that higher sleep scores and longer deep sleep durations were moderately correlated with lower MADRS scores, suggesting better sleep quality aligned with symptom improvement. Conversely, longer light sleep, total sleep time, and more time awake showed positive correlations with depression severity. While physical activity features such as step count and calories burned showed weak or inconsistent correlations, the findings overall suggest that sleep-related features may offer valuable passive indicators of depression status, though larger samples are needed to validate these patterns.

Zhang et al [19] attempted one of the largest digital phenotyping studies to date, analyzing data from over 10,000 participants in the UK general population to identify behavioral and physiological indicators of depression and anxiety. Using Fitbit wearables, they extracted 46 biweekly aggregated features, including sleep metrics (duration, onset/offset times, variability), activity measures (duration and timing of active/sedentary periods, caloric expenditure), step metrics (total step count, cadence, and timing), and HR features (mean, variability, and time-specific patterns). These wearable-derived features were combined with demographic, health, and mood variables (valence and arousal) and analyzed using XGBoost models. Feature importance was assessed using SHAP analysis. For depression (PHQ-8), the most influential features included sleep variability, wake time, step count, step timing and cadence, and evening HR. For anxiety Generalized Anxiety Disorder 7-item scale (GAD-7), HR features played a more prominent role, particularly nighttime and minimum HR, along with step cadence and sleep regularity. Mood scores (valence and arousal) and demographic variables like age, gender, and BMI were also strong predictors. The study found that models using only wearable features explained less than 15% of the variance in depression and anxiety, whereas combining wearable, mood, and baseline variables increased predictive power substantially (R² = 0.41 for depression and 0.31 for anxiety).

Song et al [20] conducted a 6-week living lab pilot study in South Korea to explore the feasibility of digital phenotyping for detecting depressive symptoms among socially vulnerable older adults using a community-based monitoring platform. Twenty-five participants (aged 76.4 on average) wore the Fitbit Sense smartwatch and engaged with a smartphone chatbot for daily verbal PHQ-9 symptom screening. Passive data were collected on three domains: sleep, HRV, and PA. From the wearable, the following features were extracted: Sleep (total sleep time, sleep fragmentation index, sleep efficiency), HRV, and PA (including step count). The adopted multilevel modeling revealed that daily increases in sleep fragmentation and decreases in sleep efficiency predicted higher odds of depressive symptoms on the following day, even after adjusting for age, sex, comorbidities, and baseline depression. In contrast, HRV and PA indicators were not significantly associated with same- or next-day depressive symptoms.

Narziev et al [21] developed the Short-Term Depression Detector system to classify individuals into depression severity using passive sensing and Ecological Momentary Assessment (EMA) over 4 weeks. A total of 20 participants were monitored using a Samsung Gear S3 smartwatch and an Android smartphone, with data collected across five DSM-5-based symptom clusters: mood, PA, sleep, social activity, and food intake. To capture these, they extracted features as follows: PA (102 features from smartphone and smartwatch accelerometers, such as step count, significant motion triggers, in forms of mean, SD, RMS, energy, and kurtosis), mood (same as PA but including HR features such as mean and SD), sleep (inferred via non-use periods, phone immobility, and darkness between 10 PM and 10 AM), social activity (number and duration of calls and social app usage), and food intake (self-reported only via EMA). In terms of feature importance, the sleep cluster was the most predictive, contributing 34% to the model. For PA, step count and significant motion were the most important. For mood, HR features were dominant. The study demonstrated strong alignment between passive sensing and EMA data, especially for sleep and mood, while social activity and food intake were less reliably captured via sensors.

Horwitz et al [22] examined whether a combination of daily mood data and wearable data could predict depression among medical interns over 3 months. A total of 2,459 participants were enrolled and provided with Fitbit Charge 4 devices, while also completing a one-item daily mood diary via a smartphone app. Features were derived from two sources: self-reported mood diary metrics (mean mood, SD, and completion rate) and Fitbit-based passively collected signals, including total sleep time, sleep onset and offset, sleep efficiency, step count, minutes spent in activity by intensity level (sedentary to very active), and resting HR. Using both elastic net regression (ENR) and RF, the authors tested eight predictive models of depression and suicidal ideation (SI) × linear and nonlinear × mood-only vs. mood + Fitbit features. Results showed that models using only mood diary features outperformed or matched those incorporating Fitbit data. For depression, ENR models using mood data alone reached acceptable predictive accuracy (AUC>0.70) within just 14 days and plateaued by around week 7. Passive Fitbit features did not improve performance and sometimes degraded it, especially in RF models.

**References**

1. Jacobson NC, Weingarden H, Wilhelm S. Digital biomarkers of mood disorders and symptom change. npj Digit Med. 2019;2(1):3.

2. Jacobson NC, Weingarden H, Wilhelm S. Using digital phenotyping to accurately detect depression severity. The Journal of nervous and mental disease. 2019;207(10):893-6.

3. Price GD, Heinz MV, Zhao D, Nemesure M, Ruan F, Jacobson NC. An unsupervised machine learning approach using passive movement data to understand depression and schizophrenia. Journal of affective disorders. 2022;316:132-9.

4. Aledavood T, Luong N, Baryshnikov I, Darst R, Heikkilä R, Holmén J, et al. Multimodal digital phenotyping study in patients with major depressive episodes and healthy controls (mobile monitoring of mood): Observational longitudinal study. JMIR Ment Health. 2025;12:e63622.

5. Anmella G, Corponi F, Li BM, Mas A, Sanabra M, Pacchiarotti I, et al. Exploring digital biomarkers of illness activity in mood episodes: hypotheses generating and model development study. JMIR mHealth and uHealth. 2023;11(1):e45405.

6. Zou B, Zhang X, Xiao L, Bai R, Li X, Liang H, et al. Sequence modeling of passive sensing data for treatment response prediction in major depressive disorder. IEEE Trans Neural Syst Rehabil Eng. 2023;31:1786-95.

7. Pedrelli P, Fedor S, Ghandeharioun A, Howe E, Ionescu DF, Bhathena D, et al. Monitoring changes in depression severity using wearable and mobile sensors. Front Psychiatry. 2020;11:584711.

8. Wang R, Wang W, DaSilva A, Huckins JF, Kelley WM, Heatherton TF, et al. Tracking depression dynamics in college students using mobile phone and wearable sensing. Proceedings of the ACM on Interactive, Mobile, Wearable and Ubiquitous Technologies. 2018;2(1):1-26.

9. Sano A, Taylor S, McHill AW, Phillips AJ, Barger LK, Klerman E, et al. Identifying objective physiological markers and modifiable behaviors for self-reported stress and mental health status using wearable sensors and mobile phones: observational study. Journal of medical Internet research. 2018;20(6):e210.

10. Hong M, Kang R-R, Yang JH, Rhee SJ, Lee H, Kim Y-g, et al. Comprehensive Symptom Prediction in Inpatients With Acute Psychiatric Disorders Using Wearable-Based Deep Learning Models: Development and Validation Study. Journal of medical Internet research. 2024;26:e65994.

11. Ahmed A, Ramesh J, Ganguly S, Aburukba R, Sagahyroon A, Aloul F. Investigating the feasibility of assessing depression severity and valence-arousal with wearable sensors using discrete wavelet transforms and machine learning. Information. 2022;13(9):406.

12. Price GD, Heinz MV, Song SH, Nemesure MD, Jacobson NC. Using digital phenotyping to capture depression symptom variability: detecting naturalistic variability in depression symptoms across one year using passively collected wearable movement and sleep data. Translational Psychiatry. 2023;13(1):381.

13. Bai R, Xiao L, Guo Y, Zhu X, Li N, Wang Y, et al. Tracking and monitoring mood stability of patients with major depressive disorder by machine learning models using passive digital data: prospective naturalistic multicenter study. JMIR mHealth and uHealth. 2021;9(3):e24365.

14. Mahendran N, Vincent DR, Srinivasan K, Chang C-Y, Garg A, Gao L, et al. Sensor-assisted weighted average ensemble model for detecting major depressive disorder. Sensors. 2019;19(22):4822.

15. Cho C-H, Lee T, Kim M-G, In HP, Kim L, Lee H-J. Mood prediction of patients with mood disorders by machine learning using passive digital phenotypes based on the circadian rhythm: prospective observational cohort study. Journal of medical Internet research. 2019;21(4):e11029.

16. Cho C-H, Lee T, Lee J-B, Seo JY, Jee H-J, Son S, et al. Effectiveness of a smartphone app with a wearable activity tracker in preventing the recurrence of mood disorders: prospective case-control study. JMIR Ment Health. 2020;7(8):e21283.

17. Tazawa Y, Liang K-c, Yoshimura M, Kitazawa M, Kaise Y, Takamiya A, et al. Evaluating depression with multimodal wristband-type wearable device: screening and assessing patient severity utilizing machine-learning. Heliyon. 2020;6(2).

18. Čermák J, Pietrucha S, Nawka A, Lipone P, Ruggieri A, Bonelli A, et al. An Observational Pilot Study using a Digital Phenotyping Approach in Patients with Major Depressive Disorder Treated with Trazodone. Front Psychiatry. 2023;14:1127511.

19. Zhang Y, Stewart C, Ranjan Y, Conde P, Sankesara H, Rashid Z, et al. Large-scale digital phenotyping: identifying depression and anxiety indicators in a general UK population with over 10,000 participants. Journal of Affective Disorders. 2025;375:412-22.

20. Song S, Seo Y, Hwang S, Kim H-Y, Kim J. Digital phenotyping of geriatric depression using a community-based digital mental health monitoring platform for socially vulnerable older adults and their community caregivers: 6-week living lab single-arm pilot study. JMIR mHealth and uHealth. 2024;12(1):e55842.

21. Narziev N, Goh H, Toshnazarov K, Lee SA, Chung K-M, Noh Y. STDD: short-term depression detection with passive sensing. Sensors. 2020;20(5):1396.

22. Horwitz AG, Kentopp SD, Cleary J, Ross K, Wu Z, Sen S, et al. Using machine learning with intensive longitudinal data to predict depression and suicidal ideation among medical interns over time. Psychological medicine. 2023;53(12):5778-85.
