# Supplementary material for: Key Features of Digital Phenotyping for Monitoring Mental Disorders: Systematic Review
Source: J Med Internet Res. 2025 Nov 5;27:e77331. doi: 10.2196/77331 (PMC12588392; doi:10.2196/77331)
Supplement: Multimedia Appendix 6 [file jmir-v27-e77331-s006.docx]

Table S7. Features used across studies on actiwatch-based mood monitoring.

| Feature lists | | Actiwatch | | | |
| --- | --- | --- | --- | --- | --- |
|  |  | SFA | Coverage (%) | Importance among used (%) | Overall importance (%) |
| BVP | | 0/0 | 0.0 | - | 0.0 |
|  | HR | 0/0 | 0.0 | - | 0.0 |
|  | Interbeat intervals | 0/0 | 0.0 | - | 0.0 |
| SpO₂ | | 0/0 | 0.0 | - | 0.0 |
| ACC | | 3/3 | 75.0 | 100.0 | 75.0 |
|  | Caloric consumption | 0/0 | 0.0 | - | 0.0 |
|  | Sedentary minutes | 0/0 | 0.0 | - | 0.0 |
|  | Activity | 1/2 | 50.0 | 50.0 | 25.0 |
|  | Steps | 0/0 | 0.0 | - | 0.0 |
|  | Motion magnitude | 0/0 | 0.0 | - | 0.0 |
| EDA | | 0/0 | 0.0 | - | 0.0 |
| TEMP | | 0/0 | 0.0 | - | 0.0 |
| Sleep | | 0/1 | 25.0 | 0.0 | 0.0 |
| Call log | | 1/1 | 25.0 | 100.0 | 25.0 |
| SMS | | 0/1 | 25.0 | 0.0 | 0.0 |
| Phone usage | | 0/1 | 25.0 | 0.0 | 0.0 |
| App usage | | 0/1 | 25.0 | 0.0 | 0.0 |
| Light exposure | | 1/1 | 25.0 | 100.0 | 25.0 |
| GPS | | 0/1 | 25.0 | 0.0 | 0.0 |

Note: BVP stands for Blood Volume Pulse; ACC for Accelerometer; SpO₂ for Peripheral Capillary Oxygen Saturation; EDA for Electrodermal Activity; and TEMP for Skin Temperature. SFA refers to the synthesized feature summary for Actiwatch. Coverage represents the percentage of studies using a given feature out of the total number of studies using Actiwatch. Importance among used refers to the percentage of studies in which the feature was identified as important among those that used it. Overall importance refers to the percentage of studies in which the feature was identified as important out of the total number of studies using Actiwatch.

Table S8. Features used across studies on smart band–based mood monitoring.

| Feature lists | | Smart band | | | |
| --- | --- | --- | --- | --- | --- |
|  |  | SFB | Coverage (%) | Importance among used (%) | Overall importance (%) |
| BVP | | 0/1 | 7.7 | 0.0 | 0.0 |
|  | HR | 7/10 | 76.9 | 70.0 | 53.8 |
|  | Interbeat intervals | 1/2 | 15.4 | 50.0 | 7.7 |
| SpO₂ | | 0/1 | 7.7 | 0.0 | 0.0 |
| ACC | | 4/6 | 46.2 | 66.7 | 30.8 |
|  | Caloric consumption | 1/3 | 23.1 | 33.3 | 7.7 |
|  | Sedentary minutes | 0/0 | 0.0 | - | 0.0 |
|  | Activity | 2/5 | 38.5 | 40.0 | 15.4 |
|  | Steps | 5/7 | 53.8 | 71.4 | 38.5 |
|  | Motion magnitude | 0/1 | 7.7 | 0.0 | 0.0 |
| EDA | | 4/4 | 30.8 | 100.0 | 30.8 |
| TEMP | | 2/3 | 23.1 | 66.7 | 15.4 |
| Sleep | | 5/10 | 76.9 | 50.0 | 38.5 |
| Call log | | 2/4 | 30.8 | 50.0 | 15.4 |
| SMS | | 0/2 | 15.4 | 0.0 | 0.0 |
| Phone usage | | 4/5 | 80.0 | 80.0 | 61.5 |
| App usage | | 1/3 | 23.1 | 33.3 | 7.7 |
| Light exposure | | 1/3 | 23.1 | 33.3 | 7.7 |
| GPS | | 2/2 | 15.4 | 100.0 | 15.4 |

Note: BVP stands for Blood Volume Pulse; ACC for Accelerometer; SpO₂ for Peripheral Capillary Oxygen Saturation; EDA for Electrodermal Activity; and TEMP for Skin Temperature. SFB refers to the synthesized feature summary for smart bands. Coverage represents the percentage of studies using a given feature out of the total number of studies using smart bands. Importance among used refers to the percentage of studies in which the feature was identified as important among those that used it. Overall importance refers to the percentage of studies in which the feature was identified as important out of the total number of studies using smart bands.

Table S9. Features used across studies on smartwatch-based mood monitoring.

| Feature lists | | Smartwatch | | | |
| --- | --- | --- | --- | --- | --- |
|  |  | SFW | Coverage (%) | Importance among used (%) | Overall importance (%) |
| BVP | | 0/0 | 0.0 | - | 0.0 |
|  | HR | 2/4 | 80.0 | 50.0 | 40.0 |
|  | Interbeat intervals | 0/0 | 0.0 | - | 0.0 |
| SpO₂ | | 0/0 | - | 0.0 | - |
| ACC | | 1/3 | 60.0 | 33.3 | 20.0 |
|  | Caloric consumption | 1/2 | 40.0 | 50.0 | 20.0 |
|  | Sedentary minutes | 0/1 | 20.0 | 0.0 | 0.0 |
|  | Activity | 0/3 | 60.0 | 0.0 | 0.0 |
|  | Steps | 2/5 | 100.0 | 40.0 | 40.0 |
|  | Motion magnitude | 1/1 | 20.0 | 100.0 | 20.0 |
| EDA | | 0/0 | 0.0 | - | 0.0 |
| TEMP | | 0/0 | 0.0 | - | 0.0 |
| Sleep | | 4/5 | 100.0 | 80.0 | 80.0 |
| CallLog | | 0/1 | 20.0 | 0.0 | 0.0 |
| SMS | | 0/0 | 0.0 | - | 0.0 |
| Phone usage | | 0/0 | 20.0 | 0.0 | 0.0 |
| App usage | | 0/1 | 20.0 | 0.0 | 0.0 |
| Light exposure | | 0/0 | 0.0 | - | 0.0 |
| GPS | | 0/0 | 0.0 | - | 0.0 |

Note: SFW refers to the synthesized feature summary for smartwatches; BVP stands for Blood Volume Pulse; ACC for Accelerometer; SpO₂ for Peripheral Capillary Oxygen Saturation; EDA for Electrodermal Activity; and TEMP for Skin Temperature. Coverage represents the percentage of studies using a given feature out of the total number of studies using smart bands. Importance among used refers to the percentage of studies in which the feature was identified as important among those that used it. Overall importance refers to the percentage of studies in which the feature was identified as important out of the total number of studies using smartwatches.

Table S10. Features used across studies on all the wearable-based mood monitoring.

| Feature lists | | All devices | | | |
| --- | --- | --- | --- | --- | --- |
|  |  | TSF | Coverage (%) | Importance among used (%) | Overall importance (%) |
| BVP | | 0/1 | 4.5 | 0.0 | 0.0 |
|  | HR | 9/14 | 63.6 | 64.3 | 40.9 |
|  | Interbeat intervals | 1/2 | 9.1 | 50.0 | 4.5 |
| SpO₂ | | 0/1 | 4.5 | 0.0 | 0.0 |
| ACC | | 8/12 | 54.5 | 66.7 | 36.4 |
|  | Caloric consumption | 2/5 | 22.7 | 40.0 | 9.1 |
|  | Sedentary minutes | 0/1 | 4.5 | 0.0 | 0.0 |
|  | Activity | 3/10 | 45.5 | 30.0 | 13.6 |
|  | Steps | 7/12 | 54.5 | 58.3 | 31.8 |
|  | Motion magnitude | 1/2 | 9.1 | 50.0 | 4.5 |
| EDA | | 4/4 | 18.2 | 100.0 | 18.2 |
| TEMP | | 2/3 | 13.6 | 66.7 | 9.1 |
| Sleep | | 9/15 | 68.2 | 60.0 | 40.9 |
| Call log | | 2/5 | 22.7 | 40.0 | 9.1 |
| SMS | | 0/3 | 13.6 | 0.0 | 0.0 |
| Phone usage | | 4/6 | 27.3 | 66.7 | 18.2 |
| App usage | | 1/5 | 22.7 | 20.0 | 4.5 |
| Light exposure | | 2/4 | 18.2 | 50.0 | 9.1 |
| GPS | | 2/3 | 13.6 | 66.7 | 9.1 |

Note: TSF refers to the total synthesized feature across all devices. BVP stands for Blood Volume Pulse; ACC for Accelerometer; SpO₂ for Peripheral Capillary Oxygen Saturation; EDA for Electrodermal Activity; and TEMP for Skin Temperature. Coverage represents the percentage of studies using a given feature out of the total number of studies using smart bands. Importance among used refers to the percentage of studies in which the feature was identified as important among those that used it. Overall importance refers to the percentage of studies in which the feature was identified as important out of the total number of studies using smartwatches.

- Although we calculated overall importance, this metric can be misleading because it does not differentiate between features that are rarely used but highly important and those that are frequently used but of low importance.
- For example, in the case of the Actiwatch, Activity has a coverage of 50% and an importance among users of 50%, whereas call log has a coverage of 25% but an importance among used of 100% (Table S7). This indicates that call log is used less frequently than Activity but is considered highly important in the studies where it is included. However, when interpreted solely based on overall importance, both Activity and call log score 25%.
- Thus, overall importance may be better understood as reflecting the proportion of importance assigned in prior studies, but it cannot distinguish whether a feature is underused despite its high importance or widely used despite its low importance. For this reason, we present the overall importance results only in Multimedia Appendix 8.
