# Supplementary material for: Key Features of Digital Phenotyping for Monitoring Mental Disorders: Systematic Review
Source: J Med Internet Res. 2025 Nov 5;27:e77331. doi: 10.2196/77331 (PMC12588392; doi:10.2196/77331)
Supplement: Multimedia Appendix 7 [file jmir-v27-e77331-s007.docx]

1. **Smartphone-exclusive sensing study**

In the main review process (Figure 1 in the main text; Flow Chart), a total of 235 articles underwent full-text screening. Among them, 100 studies were excluded due to the absence of wearable devices, which was one of our predefined eligibility criteria. Notably, this excluded subset largely consisted of studies employing smartphone-exclusive sensing. Given their conceptual relevance to digital phenotyping, we retained these studies separately for a subsequent sensitivity analysis. From this pool of 100 smartphone-only studies, we conducted an additional screening to assess the potential impact of including such studies on feature ranking. Based on the original eligibility criteria, we first screened titles and abstracts, narrowing the pool to 25 potentially relevant articles. In the second round, full-text review was conducted to exclude studies that: (1) involved participants under the age of 19; (2) were conceptually unrelated to our research focus (eg, studies that surveyed implementation challenges of smartphone sensing rather than developing predictive models); or (3) had fewer than 10 citations. After applying these criteria, 13 studies were included in the final sensitivity analysis.

Table S11 presents the basic information of smartphone-exclusive studies, the included features, and the coding process for rating feature importance (“○” or “●”). All the additional references are presented in the last chapter of this documen

Table S11. Summary of smartphone-exclusive studies, included features, and criteria for feature importance determination.

| **N** | **Device** | **Nation** | **Author** | **Target population (sample size)** | **Statistical methodology** | **Data collection period** | **Feature description** |
| --- | --- | --- | --- | --- | --- | --- | --- |
| 1 | Smartphone (Android) | Multi-country | McNamara et al [1] | 229 Global adults | Machine learning | Up to 2 weeks | App usage behavior (34 features): session length, gap time, app opens, usage by app category (social, passive info, commerce); All features were presented as important variables. |
| 2 | Smartphone (Android) | U.S. | Place et al [2] | 73 adults with PTSD or depression symptoms | Hybrid (statistical models and machine learning) | 12 weeks | Six digital trace categories were collected: Activity (accelerometer and gyroscope data including angle, velocity, and rotation), Social (call/text log metadata), Location (GPS, cell tower triangulation, and WiFi-based location), Device interaction (screen on/off timestamps), Device information (device model, battery status, OS version), and Vocal cues (voice prosody, intonation, speaking rate; lexical content not analyzed). Device information was used solely for quality assurance purposes and was therefore excluded from our analysis. These categories were reclassified into our study framework as follows: Activity → Accelerometer, Social → CallLog/SMS, Location → GPS, Device interaction → Phone Usage, and Vocal cues → Audio. While feature importance was not formally computed using techniques such as SHAP, we identified key predictors based on Table 2 and accompanying narrative descriptions. Audio features (e.g., MeanPitchVar, VocalEffort), GPS-based indicators (e.g., travel distance), and communication metrics (e.g., call.out.sum, sms.address.count) were consistently selected in symptom-specific models. In contrast, screen usage and light exposure were included but explicitly noted as having minimal influence or no interpretation. Notably, Place et al.[2] excluded activity data derived from the accelerometer, citing a lack of meaningful patterns observed during exploratory analysis. |
| 3 | Smartphone (Android) | Japan | Fukazawa et al [3] | 20 healthy adults | Machine learning | 1 month | The smartphone-sensed data introduced in the Methods section included four features: illuminance, acceleration (motion magnitude), rotation/orientation (angle to the ground: parallel, diagonal, perpendicular), and app usage (four types: Social Network, Browser, Mail, and Entertainment). These were grouped into our framework as Light exposure, Accelerometer, Gyroscope, and App Usage. The final results of this study presented the top 20 important feature combinations. Although Table 5 of Fukazawa et al. [5] highlighted that the combination of Light exposure, Accelerometer, and Gyroscope showed the highest importance in the feature importance analysis, the model including all features demonstrated the highest predictive performance, as reported in Table 4. Therefore, we considered all features to be important and rated them as “●”. |
| 4 | Smartphone (Android) | Canada | Di Matteo et al [4] | 112 adults | Machine learning | 2-weeks | The smartphone-sensed data (8 features) introduced in the Methods section included audio (volume, speech presence, death-related words), GPS (number of locations visited, number of exits from home), screen use, light sensor (time in darkness), daily similarity, and weeknight sleep disturbance. We categorized these features into our framework of sleep, phone usage, light exposure, and GPS. Audio was added as a distinct new category representing a smartphone-exclusive feature frequently highlighted in prior literature. Key features identified in the figure and discussed in the main text include the audio features (death-related words and speech presence), GPS features (number of locations visited and number of exits from home), and sleep features (weeknight sleep disturbance and daily similarity). In contrast, screen use (phone usage) was explicitly described as having minimal influence, and time in darkness (light exposure) was reported without interpretation. Accordingly, only Sleep, GPS and Audio were rated as important domains (●). |
| 5 | Smartphone (Android) | Canada | Di-Matteo et al [5] | 112 adults | Traditional statistics (Pearson, Bonferroni) | 2-weeks | This study exclusively utilized the smartphone’s audio recording function to capture ambient speech. No other feature types were collected or analyzed. All linguistic data were processed through automatic speech recognition and categorized using the LIWC (Linguistic Inquiry and Word Count) framework. Therefore, based on our classification scheme, this study was assigned to the Audio category and received a full importance rating (●), as it focused solely on this single feature type. |
| 6 | Smartphone (Android) | Denmark | Faurholt-Jepsen et al [6] | 46 patients with bipolar disorders, 31 healthy controls | Hybrid (statistical models and machine learning) | up to 9 months | This study exclusively utilized passively collected smartphone-based location data to analyze mobility patterns (e.g., number of stops, routine index, radius of gyration, location entropy). No other smartphone sensor data such as accelerometer, gyroscope, or audio was used. Based on our classification framework, this study was assigned to the GPS category and received a full importance rating (●) as it focused solely on this single feature type. |
| 7 | Smartphone (Android) | England | Gillett et al [7] | 17 patients with bipolar disorder, 17 with borderline personality disorder, and 21 healthy controls | Traditional statistics (linear mixed-effects regression models) | 3 months + up to 1 year | This study employed only two types of smartphone-derived features: **call-related features** (total call frequency, outgoing call frequency, mean total call duration, mean incoming call duration, mean outgoing call duration, cumulative incoming call duration, and cumulative outgoing call duration) and **SMS-related features** (total SMS frequency, outgoing SMS frequency, mean total SMS length, mean outgoing SMS length, cumulative total SMS length, cumulative outgoing SMS length, ratio of calls to SMS, and ratio of call duration to SMS length). Accordingly, we mapped these features to **CallLog** and **SMS** categories in our framework. As the study exclusively focused on these two feature types, both of which demonstrated significant associations with mental health status, it was not necessary to identify individual feature importance. |
| 8 | Smartphone (Android) | U.S. | Meyerhoff et al [8] | 282 adults | Traditional statistics (repeated measures correlation, etc) | 16 weeks | The smartphone-sensed data introduced in the Methods section included three categories of features: GPS-derived movement and location features (e.g., location cluster, entropy, velocity), communication data (calls and SMS), and app usage (active, passive, and social apps). Semantic location data (e.g., home, work, exercise) were also collected, but because they were based on self-reported daily labeling by participants, they were excluded from our framework focused on passively sensed features. GPS-derived features (location clusters, entropy, transitions) were mapped to GPS; app usage (active, passive, social) to AppUsage; and call/SMS data (number, duration, length) to CallLog and SMS in our framework. Important features were determined based on the main results presented in Table 1 of Meyerhoff et al. [8]. GPS showed consistently significant associations with changes in depression, whereas App usage was significant only in subgroup analyses. However, we rated both as important (●) features. |
| 9 | Smartphone (Android) | Multi-country | Opoku et al [9] | 629 adults | Hybrid (statistical models and machine learning) | 6 months | The smartphone-sensed data introduced in the Methods section included three types of behavioral markers: screen status (on/off state logs), internet connectivity (connected/disconnected), and foreground app usage (interacted apps). These were grouped into our framework as Phone Usage and App Usage. The final results of this study showed high-performing machine learning models based on these features. Feature importance analysis identified the internet regularity index, screen on/off count, screen status entropy, and app usage count as the most influential. Therefore, we considered both Phone Usage and App Usage features to be important and rated them as "●". |
| 10 | Smartphone (Android) | Multi-country | Choudhary et al [10] | 229 adults | Machine learning | 4 months | The study reported a total of 34 extracted features (detailed in Multimedia Appendix 1), which were broadly grouped into three categories: session-level interaction metrics (e.g., mean session duration, number of app launches), app-specific usage patterns (e.g., time spent and frequency across 11 categorized app types), and inactivity periods (e.g., the longest interval of non-use within a 24-hour window). These were mapped onto our framework as Phone Usage, App Usage, and Sleep. While the Multimedia Appendix clearly included a sleep-related feature (i.e., the longest duration without phone interaction), this variable was not examined or discussed in the main body of the paper. In contrast, the final analysis—particularly the binary XGBoost model—highlighted features related to Phone Usage (e.g., session duration) and App Usage (e.g., use of passive information and health-related apps) as the most predictive of generalized anxiety disorder. As such, we rated Phone Usage and App Usage as important (“●”), whereas Sleep was included but not rated. |
| 11 | Smartphone (Android) | Denmark | Faurholt-Jepsen et al [11] | 65 patients with bipolar disorder, 75 with unipolar disorder | Hybrid (statistical models and machine learning) | up to 6 months | This study exclusively focused on location data derived from GPS, WiFi, and mobile cell tower signals. The extracted features included the number of stops, total duration of stops, number of places, number of moves, total distance of moves, and others. Accordingly, we mapped all features to the GPS category in our framework. Since only one category of features was used, identifying important individual features was not necessary. |
| 12 | Smartphone (Android) | U.S. | Stamatis et al [12] | 335 adults | Traditional statistics (multilevel regression models) | 16 weeks | This study uniquely utilized linguistic features extracted from outgoing text messages using LIWC 2015. Through this method, text features were categorized into dimensions such as personal pronouns, negations, cognitive and biological process words, as well as features derived from the NRC Emotion Lexicon (eg, *anger, anticipation, and trust*) and depression/stress lexica. |
| 13 | Smartphone (Android) | U.S. | Stamatis et al [13] | 1,013 adults | Traditional statistics (multilevel linear mixed models) | 16 weeks | The smartphone-sensed data introduced in the Methods section included four main categories of passively collected features: GPS-based location data (e.g., home duration, location variability, venue frequency, circadian movement), communication logs (e.g., call/text frequency, app-based messaging), screen and launcher use (e.g., screen-on time, launcher use), and app use across types (e.g., social media, games, email, browser). These features were reclassified into our framework as follows: Location → GPS, Communication logs → CallLog / SMS / Messaging, Phone use behavior → Phone Usage, and App use → App Usage. Feature importance was not formally computed using techniques such as SHAP. However, multilevel linear mixed models were used to estimate within- and between-person associations across three time lags (distal, medial, proximal), as shown in Tables 2 and 3. Based on these results and narrative discussions, we considered features to be important if any subcomponent of a feature category was statistically significant in any prediction window. |

Table S12 lists the features used in each smartphone-exclusive sensing study, allowing for a side-by-side comparison. One key finding from this analysis is the emergence of Audio and Gyroscope features—neither of which were observed in studies that used smartphone–wearable packages. Although Gyroscope may have been collected in those multimodal studies, it was never highlighted as an important feature. In contrast, one of the smartphone-exclusive studies identified Gyroscope as a key predictor.

Overall, a distinguishing characteristic of smartphone-exclusive sensing studies is the complete absence of features based on wearable sensors, such as BVP-derived measures (heart rate, interbeat intervals, and SpO₂), Accelerometer (eg, caloric consumption, sedentary minutes, activity, steps, motion magnitude), EDA, and TEMP—except Study 2 and Study 3. Instead, these studies rely heavily on built-in smartphone functionalities, including calls, SMS, phone usage, app usage, light exposure, and GPS. A few studies also uniquely emphasized audio and gyroscope features.

Among the 13 studies, 4 utilized call-related features, and 3 of them identified these features as important. Similarly, SMS-related features were included in 5 studies, with 4 of them reported as important. Phone usage was used in 6 studies, 4 of which found it to be important. App usage appeared in all 6 relevant studies and was consistently identified as important. Light exposure features were included in 2 studies, both highlighting their importance. GPS data were also used in 6 studies, all of which reported them as important features. Notably, Audio features, newly introduced in smartphone-exclusive studies, were included in 3 studies, and all of them identified these features as important. A gyroscope was used in 1 study and was also rated as important.

**Table S12.** Features used across smartphone-exclusive studies on mood prediction and criteria for feature importance assignment.

|  | | | **Smartphone exclusive sensing studies** | | | | | | | | | | | | | |
| --- | --- | --- | --- | --- | --- | --- | --- | --- | --- | --- | --- | --- | --- | --- | --- | --- |
|  |  |  | (1) | (2) | (3) | (4) | (5) | (6) | (7) | (8) | (9) | (10) | (11) | (12) | (13) | SFP* |
| Feature list | blood volume pulse (BVP) | |  |  |  |  |  |  |  |  |  |  |  |  |  | 0/0 |
|  |  | heart rate |  |  |  |  |  |  |  |  |  |  |  |  |  | 0/0 |
|  |  | Interbeat intervals |  |  |  |  |  |  |  |  |  |  |  |  |  | 0/0 |
|  |  | SpO₂ |  |  |  |  |  |  |  |  |  |  |  |  |  | 0/0 |
|  | Accelerometer | |  | ○ | ● |  |  |  |  |  |  |  |  |  |  | 1/2 |
|  |  | Caloric consumption |  |  |  |  |  |  |  |  |  |  |  |  |  | 0/0 |
|  |  | Sedentary minutes |  |  |  |  |  |  |  |  |  |  |  |  |  | 0/0 |
|  |  | Activity |  |  |  |  |  |  |  |  |  |  |  |  |  | 0/0 |
|  |  | Steps |  |  |  |  |  |  |  |  |  |  |  |  |  | 0/0 |
|  |  | Motion magnitude |  |  |  |  |  |  |  |  |  |  |  |  |  | 0/0 |
|  | EDA (electrodermal activity) | |  |  |  |  |  |  |  |  |  |  |  |  |  | 0/0 |
|  | TEMP | |  |  |  |  |  |  |  |  |  |  |  |  |  | 0/0 |
|  | Sleep | |  |  |  | ● |  |  |  |  |  | ○ |  |  |  | 1/2 |
|  | CallLog | |  | ● |  |  |  |  | ● | ○ |  |  |  |  | ● | 3/4 |
|  | Short Message Service (SMS) | |  | ● |  |  |  |  | ● | ○ |  |  |  | ● | ● | 4/5 |
|  | PhoneUsage | | ● | ○ |  | ○ |  |  |  |  | ● | ● |  |  | ● | 4/6 |
|  | AppUsage | | ● |  | ● |  |  |  |  | ● | ● | ● |  |  | ● | 6/6 |
|  | Light exposure | |  |  | ● | ○ |  |  |  |  |  |  |  |  |  | 1/2 |
|  | GPS | |  | ● |  | ● |  | ● |  | ● |  |  | ● |  | ● | 6/6 |
|  | ***Audio (newly added)*** | |  | ● |  | ● | ● |  |  |  |  |  |  |  |  | 3/3 |
|  | ***Gyroscope (newly added)*** | |  |  | ● |  |  |  |  |  |  |  |  |  |  | 1/1 |

Note: *SFP refers to the synthesized feature summary for smartphones

**2. Impact of Including Smartphone-Exclusive Sensing Studies on Feature Rankings**

Table S5 presents the results of a sensitivity analysis conducted to address potential bias stemming from the exclusion of smartphone-exclusive sensing studies. This analysis was performed in response to concerns that excluding such studies might skew feature rankings—particularly by underrepresenting features such as GPS and app usage—and contradict the review’s stated aim of informing smart-package design.

To examine this analytically, we compared the Total Synthesized Feature set (TSF) derived from studies using smart–wearable packages only, with the extended set (TSFSA) that additionally includes the Synthesized Feature Profile (SFP) from smartphone-exclusive studies. For each feature, we calculated the percentage representation in both TSF and TSFSA and conducted a two-sided t-test to determine whether any significant differences existed. While this approach is somewhat arbitrary and was not part of the original analytic framework, it was employed to test the robustness of the synthesized findings. The results indicated no statistically significant difference between TSF and TSFSA (*P*=.446), suggesting that including smartphone-exclusive studies did not meaningfully alter the overall feature profile of the review.

Although four features—SMS (0.00→0.50), App usage (0.20→0.64), GPS (0.67→0.89), and Audio (0.00→1.00)—showed noticeable increases in percentage representation, these features were drawn from studies with highly limited sensing scopes that did not consider other critical variables such as BVP, Accelerometer, EDA, TEMP, or Sleep (as shown in Table S13). Therefore, while localized feature bias may exist within these narrowly scoped studies, their inclusion does not appear to systematically bias the overall synthesis of the review.

Table S13. Sensitivity analysis comparing studies using smart-wearable packages and smartphone-exclusive sensing approaches.

|  | | | Smart–wearable packages | | | | Smartphone-exclusive | Comprehensive model | Percentage of TSF (Existing results) | Percentage of TSFSA (Comprehensive model) |
| --- | --- | --- | --- | --- | --- | --- | --- | --- | --- | --- |
|  |  |  | SFA | SFB | SFW | TSF | SFP* | TSFSA** | TSF | TSFSA** |
| Feature list | blood volume pulse (BVP) | | 0/0 | 0/1 | 0/0 | 0/1 | 0/0 | 0/1 | 0.00 | 0.00 |
|  |  | heart rate | 0/0 | 7/10 | 2/4 | 9/14 | 0/0 | 9/14 | 0.64 | 0.64 |
|  |  | Interbeat intervals | 0/0 | 1/2 | 0/0 | 1/2 | 0/0 | 1/2 | 0.50 | 0.50 |
|  |  | SpO₂ | 0/0 | 0/1 | 0/0 | 0/1 | 0/0 | 0/1 | 0.00 | 0.00 |
|  | Accelerometer | | 3/3 | 4/6 | 1/3 | 8/12 | 1/2 | 9/13 | 0.67 | 0.69 |
|  |  | Caloric consumption | 0/0 | 1/3 | 1/2 | 2/5 | 0/0 | 2/5 | 0.40 | 0.40 |
|  |  | Sedentary minutes | 0/0 | 0/0 | 0/1 | 0/1 | 0/0 | 0/1 | 0.00 | 0.00 |
|  |  | Activity | 1/2 | 2/5 | 0/3 | 3/10 | 0/0 | 3/10 | 0.30 | 0.30 |
|  |  | Steps | 0/0 | 5/7 | 2/5 | 7/12 | 0/0 | 7/12 | 0.58 | 0.58 |
|  |  | Motion magnitude | 0/0 | 0/1 | 1/1 | 1/2 | 0/0 | 1/2 | 0.50 | 0.50 |
|  | EDA (electrodermal activity) | | 0/0 | 4/4 | 0/0 | 4/4 | 0/0 | 4/4 | 1.00 | 1.00 |
|  | TEMP | | 0/0 | 2/3 | 0/0 | 2/3 | 0/0 | 2/3 | 0.67 | 0.67 |
|  | Sleep | | 0/1 | 5/10 | 4/5 | 9/15 | 1/2 | 10/17 | 0.60 | 0.59 |
|  | Call log | | 1/1 | 2/4 | 0/1 | 2/5 | 3/4 | 5/9 | 0.40 | 0.56 |
|  | Short Message Service (SMS) | | 0/1 | 0/2 | 0/0 | 0/3 | 4/5 | 4/8 | 0.00 | 0.50 |
|  | Phone usage | | 0/1 | 4/5 | 0/0 | 4/6 | 4/6 | 8/12 | 0.67 | 0.67 |
|  | App usage | | 0/1 | 1/3 | 0/1 | 1/5 | 6/6 | 7/11 | 0.20 | 0.64 |
|  | Light exposure | | 1/1 | 1/3 | 0/0 | 2/4 | 1/2 | 3/6 | 0.50 | 0.50 |
|  | GPS | | 0/1 | 2/2 | 0/0 | 2/3 | 6/6 | 8/9 | 0.67 | 0.89 |
|  | ***Audio*** | | - | - | - | - | 3/3 | 3/3 | 0.00 | 1.00 |
|  | ***Gyroscope*** | | - | - | - | - | 1/1 | 1/1 | 0.00 | 1.00 |
| Two-sided t-test | | | | | | | *p*-value=0.446 | | | |

Note: *SFP refers to the synthesized feature summary for smartphones; **TSFSA refers to the total synthesized feature summary for Sensitivity Analysis

**3. References**

1. McNamara L, Ngai E. SADHealth: a personal mobile sensing system for seasonal health monitoring. IEEE Syst J. 2016;12(1):30-40.

2. Place S, Blanch-Hartigan D, Rubin C, Gorrostieta C, Mead C, Kane J, et al. Behavioral indicators on a mobile sensing platform predict clinically validated psychiatric symptoms of mood and anxiety disorders. Journal of medical Internet research. 2017;19(3):e75.

3. Fukazawa Y, Ito T, Okimura T, Yamashita Y, Maeda T, Ota J. Predicting anxiety state using smartphone-based passive sensing. J Biomed Inform. 2019;93:103151.

4. Di Matteo D, Fotinos K, Lokuge S, Mason G, Sternat T, Katzman MA, et al. Automated screening for social anxiety, generalized anxiety, and depression from objective smartphone-collected data: cross-sectional study. Journal of medical Internet research. 2021;23(8):e28918.

5. Di Matteo D, Wang W, Fotinos K, Lokuge S, Yu J, Sternat T, et al. Smartphone-detected ambient speech and self-reported measures of anxiety and depression: exploratory observational study. JMIR Form Res. 2021;5(1):e22723.

6. Faurholt-Jepsen M, Busk J, Vinberg M, Christensen EM, Þórarinsdóttir H, Frost M, et al. Daily mobility patterns in patients with bipolar disorder and healthy individuals. Journal of Affective Disorders. 2021;278:413-22.

7. Gillett G, McGowan NM, Palmius N, Bilderbeck AC, Goodwin GM, Saunders KE. Digital communication biomarkers of mood and diagnosis in borderline personality disorder, bipolar disorder, and healthy control populations. Front Psychiatry. 2021;12:610457.

8. Meyerhoff J, Liu T, Kording KP, Ungar LH, Kaiser SM, Karr CJ, et al. Evaluation of changes in depression, anxiety, and social anxiety using smartphone sensor features: longitudinal cohort study. Journal of medical Internet research. 2021;23(9):e22844.

9. Opoku Asare K, Terhorst Y, Vega J, Peltonen E, Lagerspetz E, Ferreira D. Predicting depression from smartphone behavioral markers using machine learning methods, hyperparameter optimization, and feature importance analysis: exploratory study. JMIR mHealth and uHealth. 2021;9(7):e26540.

10. Choudhary S, Thomas N, Alshamrani S, Srinivasan G, Ellenberger J, Nawaz U, et al. A machine learning approach for continuous mining of nonidentifiable smartphone data to create a novel digital biomarker detecting generalized anxiety disorder: prospective cohort study. JMIR medical informatics. 2022;10(8):e38943.

11. Faurholt-Jepsen M, Busk J, Rohani DA, Frost M, Tønning ML, Bardram JE, et al. Differences in mobility patterns according to machine learning models in patients with bipolar disorder and patients with unipolar disorder. Journal of Affective Disorders. 2022;306:246-53.

12. Stamatis CA, Meyerhoff J, Liu T, Sherman G, Wang H, Liu T, et al. Prospective associations of text‐message‐based sentiment with symptoms of depression, generalized anxiety, and social anxiety. Depress Anxiety. 2022;39(12):794-804.

13. Stamatis CA, Meyerhoff J, Meng Y, Lin ZCC, Cho YM, Liu T, et al. Differential temporal utility of passively sensed smartphone features for depression and anxiety symptom prediction: a longitudinal cohort study. npj Mental Health Research 3, 1 (Jan. 2024), 1–8. 2024.
